# Flying cars economically favor battery electric over fuel cell and internal combustion engine

**DOI:** 10.1093/pnasnexus/pgad019

**Published:** 2023-03-14

**Authors:** Ming Liu, Han Hao, Zhenhong Lin, Xin He, Yuping Qian, Xin Sun, Jingxuan Geng, Zongwei Liu, Fuquan Zhao

**Affiliations:** State Key Laboratory of Automotive Safety and Energy, Tsinghua University, 30 Shuangqing Road, Haidian District, Beijing 100084, China; Tsinghua-Rio Tinto Joint Research Center for Resources Energy and Sustainable Development, Tsinghua University, 30 Shuangqing Road, Haidian District, Beijing 100084, China; State Key Laboratory of Automotive Safety and Energy, Tsinghua University, 30 Shuangqing Road, Haidian District, Beijing 100084, China; Tsinghua-Rio Tinto Joint Research Center for Resources Energy and Sustainable Development, Tsinghua University, 30 Shuangqing Road, Haidian District, Beijing 100084, China; Oak Ridge National Laboratory, National Transportation Research Center, 2360 Cherahala Blvd, Knoxville, TN 37932, USA; Aramco Americas: Aramco Research Center—Detroit, 46535 Peary Ct., Novi, MI 48377, USA; State Key Laboratory of Automotive Safety and Energy, Tsinghua University, 30 Shuangqing Road, Haidian District, Beijing 100084, China; State Key Laboratory of Automotive Safety and Energy, Tsinghua University, 30 Shuangqing Road, Haidian District, Beijing 100084, China; Tsinghua-Rio Tinto Joint Research Center for Resources Energy and Sustainable Development, Tsinghua University, 30 Shuangqing Road, Haidian District, Beijing 100084, China; State Key Laboratory of Automotive Safety and Energy, Tsinghua University, 30 Shuangqing Road, Haidian District, Beijing 100084, China; Tsinghua-Rio Tinto Joint Research Center for Resources Energy and Sustainable Development, Tsinghua University, 30 Shuangqing Road, Haidian District, Beijing 100084, China; State Key Laboratory of Automotive Safety and Energy, Tsinghua University, 30 Shuangqing Road, Haidian District, Beijing 100084, China; State Key Laboratory of Automotive Safety and Energy, Tsinghua University, 30 Shuangqing Road, Haidian District, Beijing 100084, China

**Keywords:** flying car, total cost of ownership, battery, fuel cell, internal combustion engine

## Abstract

Flying cars, essentially vertical takeoff and landing aircraft (VTOL), are an emerging, disruptive technology that is expected to reshape future transportation. VTOLs can be powered by battery electric, fuel cell, or internal combustion engine, which point to entirely different needs for industry expertise, research & development, supply chain, and infrastructure supports. A pre-analysis of the propulsion technology competition is crucial to avoid potential wrong directions of research, investment, and policy making efforts. In this study, we comprehensively examined the cost competitiveness of the three propulsion technologies. Here we show that battery electric has already become the lowest-cost option for below-200-km VTOL applications, covering intra-city and short-range inter-city travels. This cost advantage can be robustly strengthened in the long term under various technology development scenarios. Battery energy density improvement is the key to reducing cost. In particular, a 600 Wh/kg battery energy density provides battery electric with all-range cost advantage, and promises high return in business. Fuel cell and internal combustion engine, under certain technology development scenarios, can obtain cost advantage in long-range applications, but face intense competition from ground transportation such as high-speed rail. The findings suggest a battery-electric-prioritized VTOL development strategy, and the necessity of developing VTOL-customized high-energy-density batteries.

**Significance Statement**

Flying cars lie at the heart of future transportation. We comprehensively examined the cost competitiveness of the three major competing propulsion technologies for VTOLs, which are battery electric, fuel cell, or internal combustion engine. The findings suggest a battery-electric-prioritized VTOL development strategy, and the necessity of developing VTOL-customized high-energy-density batteries. This sends strong messages to researchers, entrepreneurs, policy makers, and the general public, that future research, investment and policy making efforts in VTOL development should be mainly directed at battery electric, rather than fuel cell and internal combustion engine.

## Introduction

Flying cars, essentially vertical takeoff and landing aircraft (VTOL), are an emerging, disruptive technology that is expected to substantially reshape future transportation. VTOLs provide high-speed point-to-point transportation services that are faster than any other competing transport mode (1). For example, VTOL can complete a 90-km trip from San Francisco to San Jose, a 2-hour drive by on-road vehicle, in only 15 minutes (2). An advantage of using VTOL is that it can travel the straight-line distance between two places, which is much more convenient and time-saving than on-road vehicles, considering that road networks do not have straight-line distance. Adopting VTOLs for providing transportation services from one place to another can also improve the performance of the overall transportation system. Replacing a portion of on-road vehicle travel with travel by flying car is estimated to reduce traffic accidents (3, 4). Given these advantages of using VTOLs, significant industrial and commercial potential is expected from VTOLs (5–7).

Despite the widely recognized potential for their commercialization, there is less consensus on the optimal propulsion technology for VTOLs. In recent years, battery-electric (BE) propulsion systems for VTOLs are gaining interest (8–10), much like they are for on-road transport, owing to the advancement in battery and distributed electric propulsion technologies (11–14). Numerous concepts and prototypes of BE VTOLs have been proposed by Airbus, Boeing, Bell, EHang, and others. With the consistent development of fuel cell and hydrogen storage technologies, another alternative technology, fuel cell (FC) propulsion, has also attracted increased research and industrial interests. Companies such as IO Aircraft, LuftCar, and Metro Skyways have also developed prototypes of FC VTOLs (15–17). Another potential candidate is a conventional technology called internal combustion engine (ICE) propulsion. This technology, when coupled with renewable-based sustainable aviation fuel (18), could provide the same low-carbon benefits as BE VTOLs and FC VTOLs. Further complexity is added to the technology competition when considering the wide range of VTOL applications, such as one-seater private flying cars, four-seater air taxis, high-capacity air vans, and airport shuttles, that might favor different propulsion technologies. These knowledge gaps point to the necessity of conducting a comprehensive economic analysis of VTOLs.

Total cost of ownership (TCO) analysis is an effective method for investigating the economic performance of competing technologies. It calculates all costs occurring in the life cycle of research objects (e.g. manufacturing cost, operation cost, maintenance cost, etc.) and is widely employed in forming technology roadmaps in the transport sector (19–23). By using TCO analysis, future competition among candidate propulsion technologies can be observed, impediments to the commercialization of VTOLs can be identified, and application scenarios best suited for VTOL utilization can be further explored. Although a few studies have estimated the TCO of VTOLs (2, 15), no comprehensive TCO analysis covering the full spectrum of competing technologies and typical applications has been published. Thus, the present study bridges the research gap by developing a VTOL operation model and calculating the TCO of VTOLs under various technologies and applications. Here, we show that BE VTOLs are likely to prevail in intra-city and short-range inter-city transport that are likely to become the main application scenario for VTOLs. Moreover, it should be noted that the future market competitiveness of BE VTOLs is shaped by battery energy density.

## Results

### Competition among propulsion technologies

As mentioned in the Materials and methods section, this study calculates the TCO of VTOLs under different technologies and applications using (1) the VTOL technological model that simulates flying processes and estimates component sizes, and (2) the cost model that systematically calculates all aspects of VTOL operation costs. We first observed the competition among different propulsion technologies by using TCO estimates. For this, six competing technologies were defined based on propulsion technology (BE, FC, ICE) and the fuel type used (fossil energy [FE] based and renewable energy [RE] based). FE includes natural gas-powered electricity, natural gas-derived hydrogen, and gasoline for BE, FC, and ICE, respectively. RE includes renewable-based electricity, renewable-based green hydrogen, and sustainable aviation fuel for BE, FC, and ICE, respectively. Besides the assumed FE and RE scenarios, the results under more realistic electricity energy scenarios (e.g. the US grid) are provided in Figure S72.

**Fig. 1.**
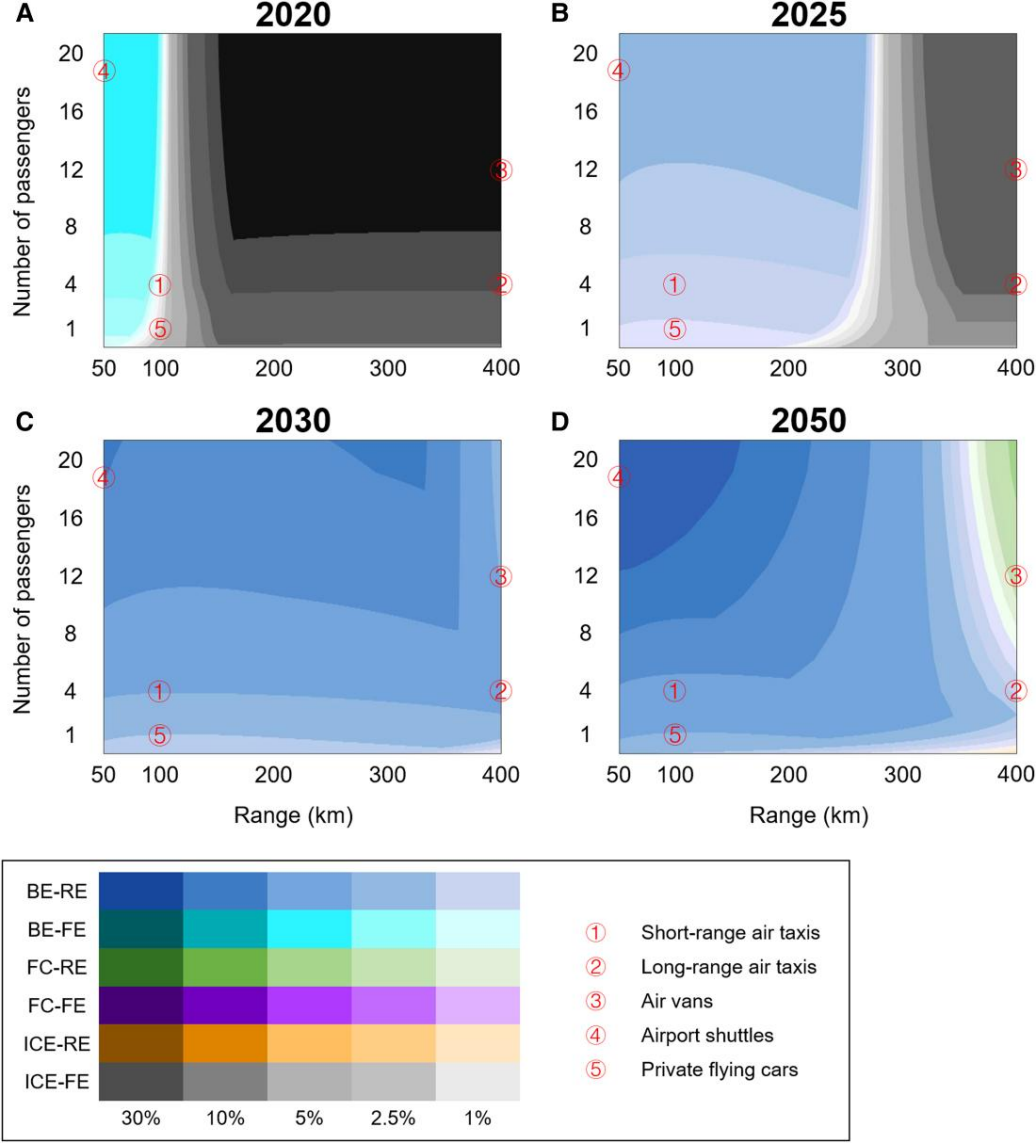
TCO-advantageous technologies for different VTOL applications under the baseline scenario. The results are presented under assumed technology improvements for the years of 2020 (A), 2025 (B), 2030 (C), and 2050 (D), respectively. The TCO-advantageous technology for a given application domain is indicated by the color of the area. Color shading indicates the relative advantage of the leading technology against the second-best technology. A darker color indicates a stronger advantage and vice versa. In each subfigure, typical applications of VTOLs, including air taxis, air vans, airport shuttles, and private flying cars, are indicated using circled numbers. BE, battery electric; FC, fuel cell; ICE, internal combustion engine; FE, fossil energy-based; RE, renewable energy-based.

Figure 1 shows the most TCO-advantageous technologies for different VTOL applications in terms of travel range and number of passengers. These results are provided under assumed technology improvements under the baseline scenario for the years of 2020, 2025, 2030, and 2050 (see Segment 3 of Table S1). Figure 7 shows the key assumptions for the baseline scenario, with their justifications in Supplementary material text. In the 2020 scenario, BE-FE and ICE-FE are the most competitive technologies in terms of TCO, as indicated by the cyan and gray colors in Fig. 1A. In particular, BE-FE shows strong competitiveness for short-range transport within 100 km, while ICE-FE dominates the rest of the application domain, especially with more than eight passengers and a range of 200 km or more, as shown by the dark gray color in Fig. 1. Regarding the five typical applications shown in Fig. 1, BE-FE is preferred for airport shuttles, ICE-FE is preferred for long-range applications, and either BE-FE or ICE-FE is preferred for short-range air taxis and private flying cars.

The 2025 scenario represents technology competition under near-term, predictable technology improvements. Significant changes can be expected under this scenario due to improvements in battery energy density and reductions in RE cost. As indicated by the blue color in Fig. 1B, BE-RE is the most promising technology for short- and medium-range transport up to 250 km, and is preferred for short-range air taxis, airport shuttles, and private flying cars. In addition, the TCO-advantageous domain of ICE-FE (gray color) in this scenario contracts significantly, maintaining an advantage in long-range transport only.

In the 2030 scenario, further technology improvements are expected. Accordingly, BE-RE (blue color) consolidates its TCO advantage in short- and medium-range transport and further expands its advantage into the long-range application domain, replacing the role of ICE-FE entirely.

Fully developed powertrain and energy technologies are assumed in the 2050 scenario. Here, FC-RE emerges as a TCO-competitive technology in the long-range transport domain, covering the typical application of air vans, as indicated by the green color in Fig. 1D. Nevertheless, BE-RE dominates the majority of the application domain. Notably, neither FC-FE nor ICE-RE emerges as a TCO-advantageous technology in the baseline scenario.

The sensitivity of TCO-advantageous technology results to the changes in battery life, VTOL life, FE energy price, RE energy price, etc., is provided in Figures S60–S71. These figures show an overall technology competition landscape similar to that in Fig. 1. Notably, longer battery life, lower RE energy price, higher FE energy price settings, etc., can further expand the TCO-advantageous domain of BE-RE. In addition, FC-FE will become a TCO-competitive technology in the long-range transport domain, with lower battery energy density or FE energy price settings, as shown by Figures S62 and S64.

In the baseline scenario with reference technology improvements, both battery and fuel cell technologies are undergoing rapid technology changes, making technology projection extremely difficult and, under some circumstances, misleading. While high confidence can be attached to the current and near-term analyses (2020 and 2025 scenarios), there is high uncertainty in the long-term analysis (2050 scenario), especially when considering that the prevalence of BE-RE VTOL only rests on a marginal TCO advantage over other competing technologies, as indicated by the light shading in Fig. 1D. Therefore, technology competition under four sets of alternative battery and FC technology assumptions (see Segment 4 of Table S1) is further analyzed in the 2050 context to address the above-mentioned uncertainty. This analysis can reveal a full spectrum of future technology competition possibilities. Figure 1 shows the key assumptions for different technology improvement possibilities in the long term (2050).

**Fig. 2.**
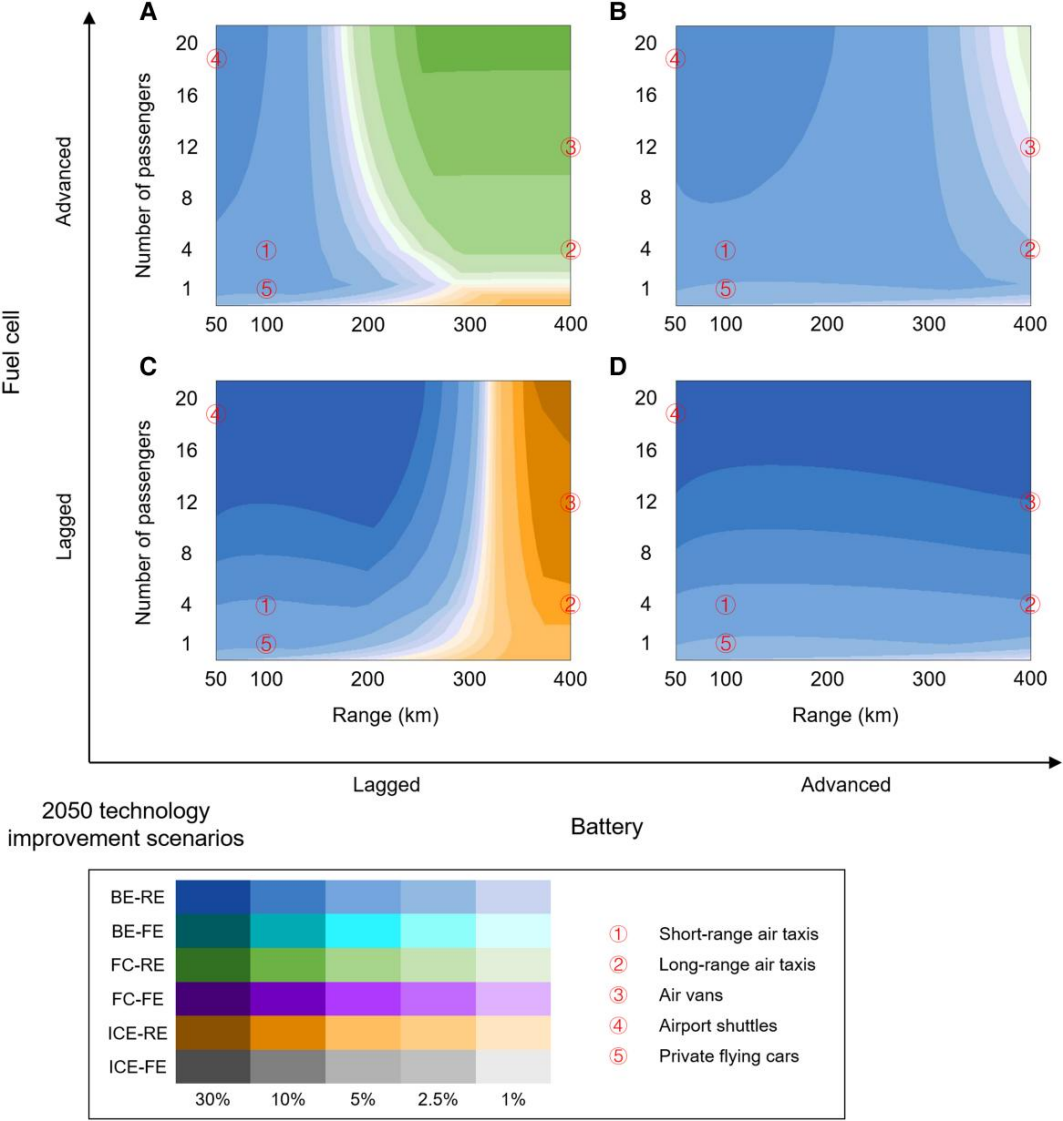
TCO-advantageous technologies under alternative battery and fuel cell technology improvement scenarios in 2050. The four subfigures are organized by the technology improvement levels of batteries (horizontal dimension) and fuel cells (vertical dimension): lagged battery vs. advanced fuel cell (A); both advanced (B); both lagged (C); advanced battery vs. lagged fuel cell (D). BE, battery electric; FC, fuel cell; ICE, internal combustion engine; FE, fossil energy-based; RE, renewable energy-based.

Figure 2 presents technology competition under different degrees of technology improvements for batteries and fuel cells in 2050. As shown by the green color with larger area in Fig. 2A, the TCO-advantageous domain of FC-RE extends to short-range applications under advanced fuel cell and lagged battery technology assumptions. Meanwhile, the TCO-advantageous domain of BE-RE contracts significantly, with a weak advantage in trips up to 200 km and low-capacity trips. In contrast, as indicated by the blue color in the whole subfigure in Fig. 2D, BE-RE dominates the whole application domain with lagged fuel cell and advanced battery technology improvements. In particular, the TCO of BE-RE is more than 20% lower than that of the other technologies for trips with more than 12 passengers, which is shown by the dark blue color in the figure.

In the context of advanced development for both battery and fuel cell technologies (Fig. 2B), the competition landscape between BE-RE and FC-RE is similar to the baseline scenario. However, the differences between the TCO of BE-RE and FC-RE are less than 7.5% in most applications, as shown by the light shading in the whole subfigure. Thus, this indicates intense competition between these two technologies. When advancements in battery and fuel cell technologies lag behind expectations (Fig. 2C), BE-RE remains advantageous in most of the application domain, as shown by blue color. Meanwhile, ICE-RE emerges as the most TCO-competitive technology in long-range transport, covering the typical applications of long-range air taxis and air vans, as shown by the orange-brown color.

### Driving factors behind technology competition

**Fig. 3.**
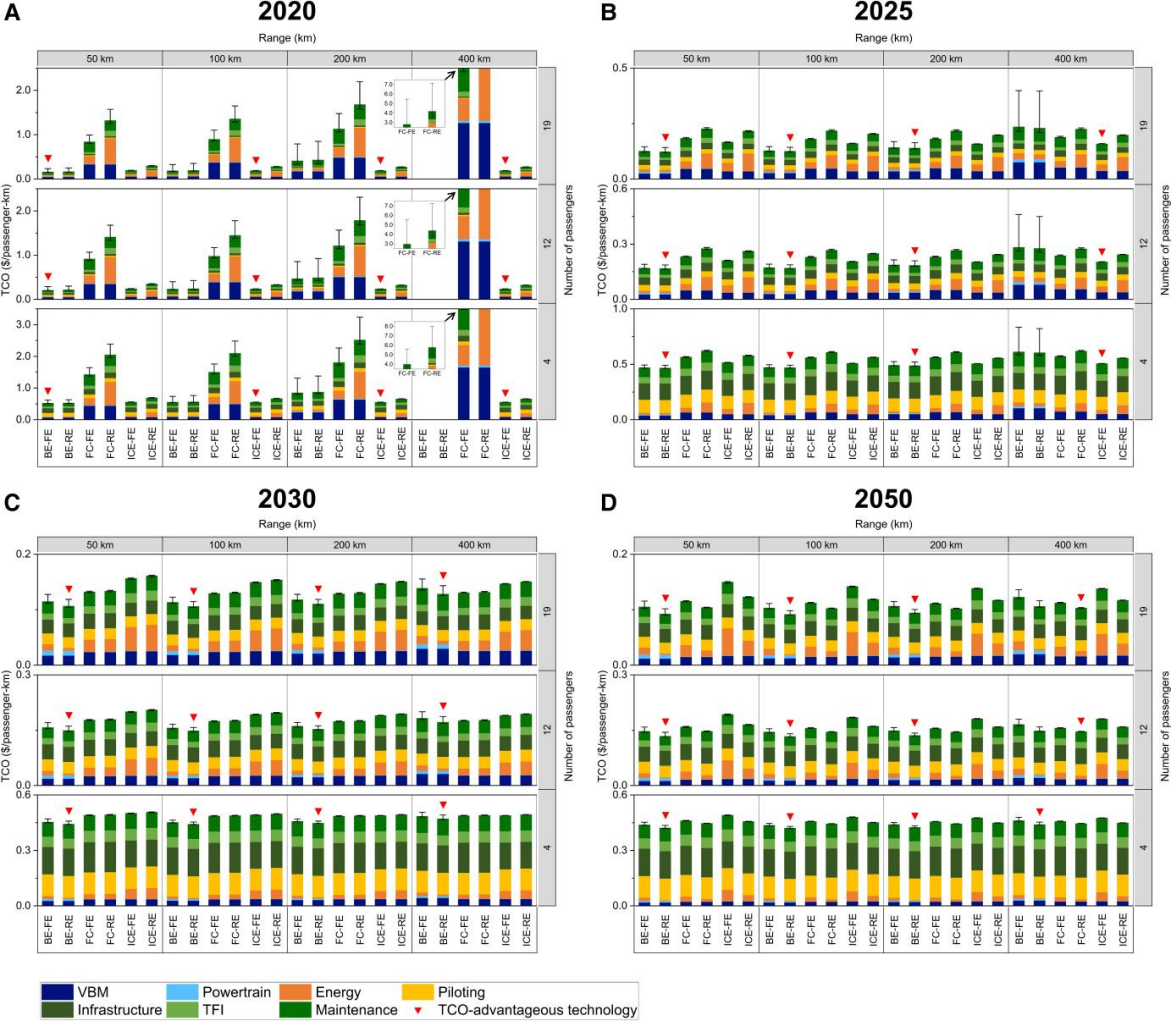
TCO breakdown under baseline technology improvements in 2020 (A), 2025 (B), 2030 (C), and 2050 (D). Each subfigure is divided into twelve application domains distinguished by the number of passengers (vertical dimension) and the range (horizontal dimension). For each application domain, the TCO estimates for six propulsion technologies are shown, with different colors representing different cost components. The absence of estimates for some circumstances indicates technological infeasibility. Salvage value is not shown due to its marginal influence on TCO. BE, battery electric; FC, fuel cell; ICE, internal combustion engine; FE, fossil energy-based; RE, renewable energy-based; TCO, total cost of ownership; VBM, VTOL body manufacturing; TFI, taxes, fees, and insurance. The TCO breakdown under alternative scenarios for 2050 is provided in Figure S1. Specific results are provided in Tables S2–S11.

**Fig. 4.**
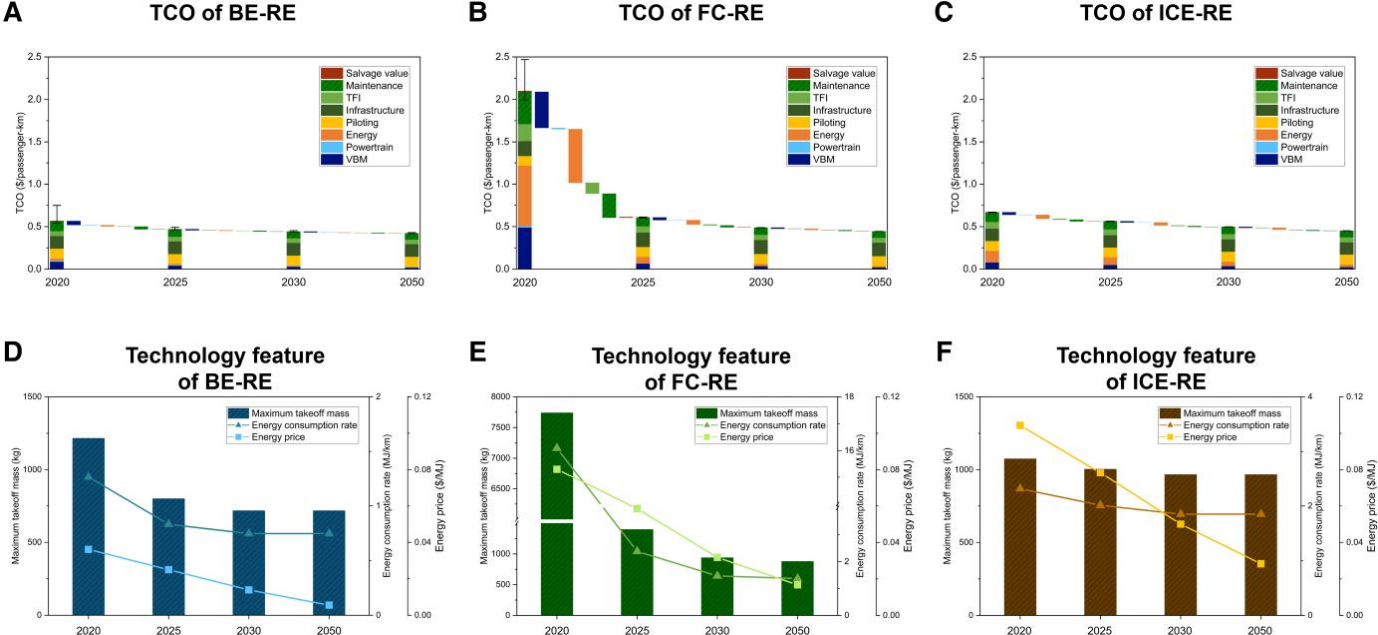
TCO changes and the corresponding contributing factors for three technology and energy source combinations (BE-RE, FC-RE, and ICE-RE) under the baseline scenario for short-range air taxis. Subfigures (A)–(C) show the changes in the TCO, with the changes broken down into contributions from different cost components. Subfigures (D)–(F) show the changes in key technology parameters (i.e. maximum takeoff mass, energy consumption rate, and energy price). BE, battery electric; FC, fuel cell; ICE, internal combustion engine; RE, renewable energy-based; TCO, total cost of ownership; VBM, VTOL body manufacturing; TFI, taxes, fees, and insurance. TCO changes and the corresponding contributing factors for other applications are provided in Figures S2–S18.

As shown in Figs. 3 and 4, the driving factors behind the changes in the technology competition landscape are further analyzed by looking into the TCO breakdown. The VTOL body manufacturing (VBM) and maintenance costs—maintenance cost is primarily determined by VBM cost, as explained in the Materials and methods section—are the major differentiating factors among technologies in the early stage of VTOL development. For example, the VBM and maintenance costs of 200-km-range air taxis under the current conditions are $0.23/passenger-km and $0.64/passenger-km for BE and FC, respectively, and only $0.08/passenger-km for ICE. Such cost differences become more significant for long-range applications. Thus, BE and FC are currently not TCO-competitive against ICE in most applications. The high VBM costs of BE and FC can be primarily attributed to the low energy density of batteries and the low energy efficiency and power density of fuel cells, which lead to high VBM weight and the consequent high cost. The VBM and maintenance costs of BE and FC can be substantially reduced to a similar level as those of ICE with the expected technology improvements in 2025 and beyond. With such changes, the impact of VBM and maintenance costs on TCO differences among technologies gradually becomes marginal. The reason behind such changes is the iterative calculation, technology improvements, with reductions in energy prices, etc., which can bring significant TCO reductions, especially from 2020 to 2025 (e.g. a 70% TCO decrease of FC-RE VTOLs from 2020 to 2025).

As another part of VTOL manufacturing cost, powertrain cost (light blue color) imposes a far smaller impact than the VBM cost. Powertrain costs account for less than 1% of their respective total costs for most applications for FC and ICE technologies. The powertrain cost of BE plays a similar role under short-range and low-capacity applications. However, the powertrain cost of BE increases significantly for long-range and high-capacity applications, primarily due to the large battery capacity required to sustain the flight. For example, for air vans, the powertrain cost of BE is $0.01/passenger-km in 2025 and $0.007/passenger-km in 2050, about 10 times higher than for FC and ICE. This explains why BE shows a lower TCO competitiveness in such applications.

Energy cost (orange color) is the key factor in determining the market competitiveness of emerging technologies in road transport (21, 24). It reflects the energy efficiency and energy price differences among technologies. For VTOLs, the impact of energy cost on TCO can be different under different technology development and application contexts. For example, for short-range air taxis (Fig. 4), the per-passenger-km energy costs of BE-RE, FC-RE, and ICE-RE in 2025 are $0.01, $0.08, and $0.09, respectively, which significantly distinguish the TCO competitiveness among technologies. However, in 2050, the per-passenger-km energy costs of BE-RE, FC-RE, and ICE-RE will decline to $0.002, $0.01, and $0.03, respectively, accounting for 0.5%, 3%, and 6% of their respective total costs. This is because of the technology developments that leads to substantial energy consumption reductions, as well as the expected reductions in RE prices. Under such circumstances, energy cost has little impact and contributes to the convergence of TCO among technologies. For high-capacity applications such as airport shuttles, energy cost tends to consistently play an important role in total cost. The shares of energy costs for airport shuttle applications are 2%, 11%, and 22% for BE-RE, FC-RE, and ICE-RE, respectively, in 2050. In such applications, BE technology exhibits a consistent energy cost advantage due to the inherent high-energy efficiency and low energy price.

Piloting and infrastructure costs (yellow and dark green colors) are relatively insensitive to VTOL technology development. For example, for short-range air taxis, these two cost components add up to approximately $0.25/passenger-km to $0.30/passenger-km for BE, FC, and ICE in 2020, accounting for approximately 14–45% of total costs. In the long run, piloting and infrastructure costs gradually become the dominant share of TCO due to the substantial reductions in VBM, maintenance, and energy costs. In 2050, these two cost components account for around 60% of the TCO for short-range air taxis, implying substantial changes in the relative importance of these costs.

This analysis also shows the sensitivity of the TCO estimates to the changes in VTOL technical parameters, including cruising speed, cruising altitude, and energy reserve amount. TCO estimates tend to be sensitive to parameter changes under immature BE and FC technologies. The upper bounds of the TCO uncertainty range of BE and FC short-range air taxis in 2020 are $0.76/passenger-km (35% higher than baseline estimate) and $2.48/passenger-km (18% higher than baseline estimate), respectively. In contrast, the change is only 0.2% for ICE. This uncertainty can be mitigated with increased technical maturity. In 2050, the TCO uncertainty ranges for both BE and FC are within 2% of their respective baseline estimates.

Notably, VTOL technical parameters are highly related to regulations. In this research, the baseline assumptions of VTOL technical parameters are mainly based on current Federal Aviation Administration regulations (see Supplementary material text). The sensitivity of the TCO estimates to the changes in regulations (lax or strict regulations considering different operation situations) is provided in Figure S74 so as to further investigate the influence of regulations. The results show that TCO estimates of short-range or low-capacity applications are less sensitive to regulation changes. However, long-range or high-capacity applications show much higher TCO uncertainty with different regulation scenarios, especially in the near term when technologies are still immature, demonstrating the importance of setting proper regulations for these applications.

The sensitivity of TCO estimates to a wider range of parameter changes is provided in Figures S30–S59, with Fig. 5 summarizing the sensitivity of TCO estimates of short-range air taxis as an example. In the case of BE VTOLs, the TCO results are more sensitive to battery energy density, base VBM cost, piloting cost, and TFI cost, while they are less sensitive to energy price, battery cost, etc. In the near term, the TCO results of FC VTOLs are highly sensitive to fuel cell power density and hydrogen price. In the long term, the TCO results of FC VTOLs are more sensitive to piloting cost and TFI cost, compared with other parameters. Given the high technical maturity, ICE VTOLs show higher sensitivity to piloting cost, base VBM cost, and TFI cost than other parameters. In addition, the TCO results under different discount rate scenarios are provided in Figure S73.

**Fig. 5.**
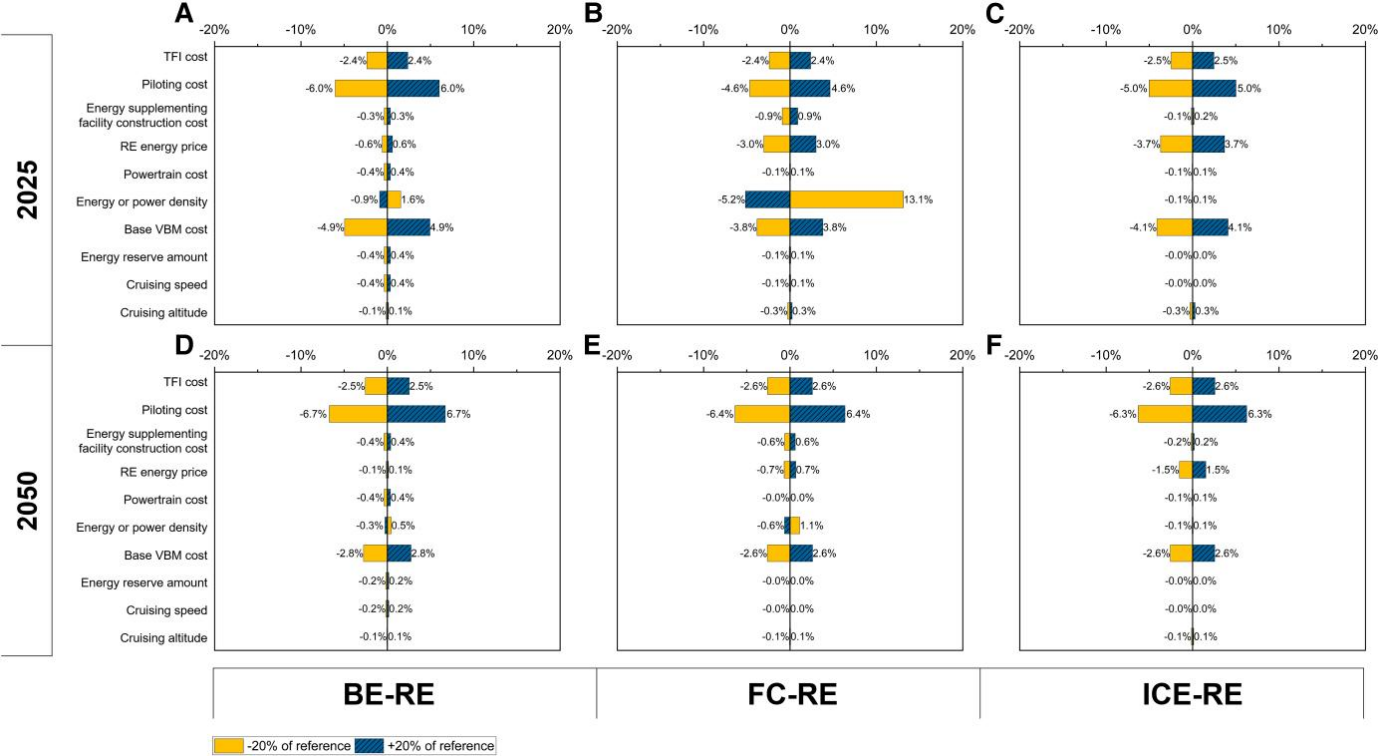
Sensitivity analysis on the TCO of short-range air taxis with RE technologies in 2025 and 2050. The subfigures present the sensitivity of the TCO estimates to changes in VTOL technical parameters (i.e. cruising speed, cruising altitude, energy reserve amount, energy density for BE, and power density for FC and ICE) and cost parameters (i.e. base VBM cost, powertrain cost, RE energy price, energy supplementing facility construction cost, piloting cost, and TFI cost). Subfigures (A)–(C) show the sensitivity of the TCO estimates of BE-RE, FC-RE, and ICE-RE short-range air taxis, respectively, in 2025 scenario (near term). Subfigures (D)–(F) show the sensitivity of the TCO estimates of BE-RE, FC-RE, and ICE-RE short-range air taxis, respectively, in 2050 scenario (long term). BE, battery electric; FC, fuel cell; ICE, internal combustion engine; RE, renewable energy-based; TCO, total cost of ownership; VBM, VTOL body manufacturing; TFI, taxes, fees, and insurance.

### Application priorities

**Fig. 6.**
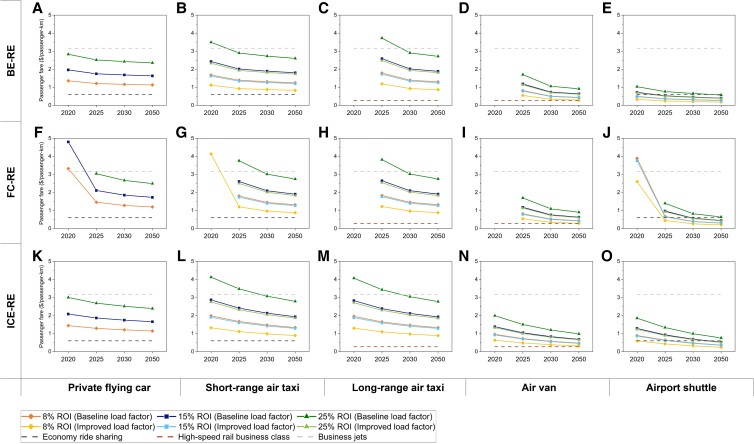
Required passenger fares for achieving target ROIs. The subfigures represent results under different applications (horizontal dimension) and technologies (vertical dimension): BE-RE for private flying cars (A), short-range air taxis (B), long-range air taxis (C), air vans (D), and airport shuttles (E); FC-RE for private flying cars (F), short-range air taxis (G), long-range air taxis (H), air vans (I), and airport shuttles (J); ICE-RE for private flying cars (K), short-range air taxis (L), long-range air taxis (M), air vans (N), and airport shuttles (O). The required fares are provided for three ROIs (10%, 15%, and 25%) and two load factor cases (baseline vs. improved). For some cases, where required fares are too high to be realistic, the fares are not plotted. Average passenger fares for economy ride-sharing, high-speed rail business class, and business jets are provided as benchmarks to be compared with the results. BE, battery electric; FC, fuel cell; ICE, internal combustion engine; RE, renewable energy-based; ROI, return on investment. Required passenger fares under other conditions are provided in Figures S19–S23, with all specific data provided in Tables S12–S21. ROI of VTOL operation is provided in Figures S24–S29.

Although VTOL has a wide range of applications, these applications are not equally important in commercial terms. Thus, it is crucial to identify the commercially prioritized applications so as to further differentiate the relative importance of the propulsion technologies. Thus, in this study, we estimated the passenger fares required to achieve the target return on investment (ROI) so as to evaluate the commercialization priorities of VTOL applications (Fig. 6). An ROI of 10% was set as the benchmark, which is about the average ROI in the airline and the economy ride-sharing business (25, 26). Based on this benchmark, the required passenger fares for BE-RE in 2020 are $1.53/passenger-km for private flying cars, $1.89/passenger-km for short-range air taxis, and $0.56/passenger-km for airport shuttles. As shown, the required fare for airport shuttles is already lower than that for economy ride-sharing ($0.60/passenger-km) (5), demonstrating its strong potential for commercialization. The required fares for private flying cars and short-range air taxis are lower than the equivalent passenger fare for business jets that offer similar transport services ($3.16/passenger-km) (27), although they are much higher than for airport shuttles. Nevertheless, the required fares for all applications are reduced with further technology development in 2025 and onwards. In 2050, the required passenger fares for airport shuttle services decline to $0.31/passenger-km, 49% lower than for economy ride-sharing. The fares for private flying cars and short-range air taxis decline to $1.27/passenger-km and $1.41/passenger-km, respectively, both of which are about double the fare of economy ride-sharing. The required fares for ICE VTOLs and FC VTOLs for achieving the 10% ROI are higher than those for BE VTOLs under most circumstances, due to the higher TCO, as shown in the previous sections. Considering that early,stage investors might require much higher ROI, the passenger fares required to achieve high ROI (40 and 60%) are provided in Figures S75 and S76.

The long-range applications in the near term are only economically viable with ICE propulsion technology, with passenger fares of $2.20/passenger-km for long-range air taxis and $1.08/passenger-km for air vans required to reach 10% ROI. In the long term, BE, FC, and ICE show similar profitability. In order to achieve 10% ROI, the required passenger fare for BE-RE air vans in 2050 is $0.50/passenger-km, 83% higher than that for business class high-speed rail (28). The development of high-capacity and long-range applications will face intense competition from high-speed rail, considering the fact that air vans do not show an obvious advantage in transportation efficiency and privacy. However, long-range air taxis require a significantly higher passenger fare ($1.47/passenger-km) to reach the 10% ROI in 2050 than the competing transport modes. Nevertheless, they provide differentiated service in terms of privacy and comfort and can thus meet the needs of the niche market.

As expected, an improved load factor—an increase in the number of passengers that can be carried per unit flight—reduces the passenger fare required to reach the same ROI. When the load factor of air taxis is increased from 50% (baseline load factor, 1.5 passengers carried on average) to 75% (improved load factor, 2.25 passengers carried on average), the passenger fares required to meet 10% ROI in 2025 are lowered to $1.05/passenger-km for BE-RE short-range air taxis and $1.34/passenger-km for BE-RE long-range air taxis. These reductions are more than 30% lower than the fares required with the baseline load factor. In addition, these fares are even lower than the required passenger fare for private flying cars ($1.36/passenger-km), which provides air taxis with priority for commercialization. In the early stage of VTOL commercialization, the reduction in required passenger fare through improved load factor is important, since it lowers the economic bar for consumer participation and could induce an earlier commercial breakthrough.

While an ROI of 10% is sufficiently profitable for conventional transport business, VTOLs can achieve a much higher ROI with passenger fares set at reasonable levels, especially under short-range applications. The ROI of BE VTOLs in 2030 can reach 13% for private flying cars, 10% for short-range air taxis, and 67% for airport shuttles when the passenger fare is set at 2.5 times the fare of economy ride-sharing, which is acceptable from the consumer perspective considering the substantial improvement in commuting efficiency (29). The values for short-range air taxis and airport shuttles could further increase to 19 and 104%, respectively, with an improved load factor. This reveals the potential of short-range VTOL transport as a high-profitability business.

## Discussion

Our comprehensive TCO analysis of VTOLs shows that both the development of VTOL business as a whole and the internal competition among different propulsion technologies rely critically on technology development and application scenario. Below we discuss some of the most important implications.

In this analysis, we demonstrated the high-return potential for short-range VTOL applications. We confirmed the potential of VTOLs to reshape future short-range transport, particularly intra-city and short-range inter-city transport. BE is a compelling propulsion technology with a TCO advantage for short-range applications under most of the scenarios in this study. FC and ICE are preferred for long-range VTOL applications, although they face competition from high-speed rail and show neither service nor economic advantages. Therefore, our findings show that BE will be the dominant propulsion technology for future VTOL applications providing short-range services, while FC and ICE could be used for long-range VTOL applications.

As per the results of the TCO analysis, battery technology is an essential aspect of future BE VTOL development and determines the evolution of technology competition. Currently, battery technology development is mainly driven by the large-scale deployment in on-road vehicles. Central to the development of on-road vehicle batteries is a low battery cost that can make the price of electric vehicles acceptable to consumers. However, battery energy density is a relatively lower priority for on-road vehicles, as demonstrated by the prevalence of lithium iron phosphate batteries. In the case of VTOLs, the tradeoff among battery attributes can develop in a different way. Although safety is the main priority (30), battery energy density is equally important as it determines battery weight and substantially affects the weight and associated costs of other VTOL components. BE VTOLs can achieve much lower TCO and higher profitability with high-energy-density batteries, as shown in Figs. 3, 4 and 6, Figures S2–S12, and S19. Thus, for example, BE VTOLs require a 600 Wh/kg battery, which is far beyond the energy density of on-road vehicle batteries, to make economic sense. However, battery cost can be compromised in battery design as it becomes far less important, considering its limited share in the total cost. This suggests new perspectives for the development of 600 Wh/kg and even higher-energy density batteries, which represent next-generation battery technologies, such as solid-state lithium-metal anode batteries, lithium-sulfur batteries, and others (31). This finding also shows the importance of developing VTOL batteries with their own technology roadmap, rather than following the same technology development path as on-road vehicles.

The economic feasibility of VTOLs, especially in the near term, can be increased by reducing VBM cost. On the one hand, the VBM cost is highly sensitive to VTOL weight due to the utilization of high-cost materials such as titanium alloy (32), which again demonstrates the significance of high-energy-density battery technology for the VTOL industry. On the other hand, increasing production volume is essential to achieving economies of scale, which can significantly reduce the VBM cost through lowered material, assembly, and R&D costs. Therefore, it is essential to stimulate market growth in the early stage of VTOL commercialization. This will require economic incentives for VTOL transport operation, accelerated construction of infrastructures such as vertiports and VTOL energy supplementing facilities, and public education to improve social acceptance.

Unmanned autonomous driving could also boost VTOL development. As shown in the analysis, piloting cost is expected to become the largest share of operating cost in the long term. Thus, adopting advanced unmanned autonomous driving systems for VTOLs, which would eliminate the need for pilots in most cases (except for rough weather conditions), could significantly reduce piloting costs. In addition, eliminating the pilot need leaves another seat available for passenger transport, bringing additional profits to operators. An additional passenger seat could substantially increase passenger capacity (e.g. a one-third increase for a four-seater air taxi), and thus, eliminating the pilot need is particularly important for low-capacity VTOL operation.

High-capacity applications could be considered a priority for VTOL commercialization due to the low passenger fare required to sustain a profit. In 2021, airlines worldwide carried more than 2.2 billion passengers (25, 33), most of whom had to spend considerable time traveling to the airport by taxi, bus, or subway. Most of this travel to the airport could be carried out by VTOL airport shuttles in less than half of the time required by conventional ground transportation. This is only one of the many expected intra-city applications for VTOLs (34). Aggressive strategies (such as low fares) can be used to attract consumers from other competing transport modes as high-capacity VTOLs provide operators with substantial profit margins (35).

High profitability of high-capacity applications demonstrates the great potential of VTOLs for large-scale deployment. In addition, additional congestion in VTOL transportation is less likely to occur with the large-scale deployment of VTOLs. On the one hand, there is more room for VTOL operation as VTOLs can fly at different altitudes without road network constraints in a three-dimension transportation system. On the other hand, VTOLs are expected to get more regulatory supports on their operation, with VTOL development strategies being proposed by many countries such as China, considering that a three-dimension transportation system could improve the transportation efficiency of road traffic (4).

Low-capacity VTOL applications could further enhance privacy and comfort, serving as a one-of-a-kind transport service that none of the existing transport modes can provide. Intra-city or short-range inter-city trips could be suitable for air taxis, considering that airlines hardly run such short-range routes, high-speed rails lack privacy, and on-road taxis and buses have much lower transport efficiency. This provides an opportunity for a high-fare, differentiated-service strategy. Ensuring high load factors is also essential for these low-capacity applications. In addition, user behavior strategies, such as encouraging passengers to carpool, can be considered to lower fares.

## Materials and methods

### Flying process modeling

For VTOLs, a flight can be divided into five sub-phases: hovering, climbing, cruising, descending, and landing. While hovering and landing, VTOLs take off and land vertically. While climbing and descending, VTOLs fly to or descend from the cruising altitude at the angle of attack (AOA). While cruising, VTOLs fly horizontally with a certain cruising speed at the cruising altitude. Based on each sub-phase's kinematic and dynamic characteristics, the power demand in each sub-phase can be obtained (Eqs. 1–3).


(1) $$P_{hover}=\frac{m_{TO}g}{\eta_{hover}}\sqrt{\frac{\delta }{2\rho }}$$

(2) $$P_{climb}=\frac{m_{TO}g}{\eta_{climb}}\left(ROC+\frac{ROC}{sin\alpha \times L/D_{climb}\;}\right)$$

(3) $$P_{cruise}=\frac{m_{TO}g}{\eta_{cruise}}\frac{V}{L/D_{cruise}}$$

where *P*_*hover*_, *P*_*climb*_, and *P*_*cruise*_ are the power demand of VTOLs while hovering/landing, climbing/descending, and cruising, respectively (kW);

*m*_*TO*_ is the maximum takeoff mass (kg);

*η*_*hover*_, *η*_*climb*_, and *η*_*cruise*_ are the propulsion system efficiencies while hovering/landing, climbing/descending, and cruising, respectively;

*δ* is the disk loading (N/m^2^);

*ρ* is the air density (kg/m^3^);

*ROC* is the rate of climb (m/s);

*L*/*D*_*climb*_ and *L*/*D*_*cruise*_ are the lift-to-drag ratios while climbing/descending and cruising, respectively;

*α* is the AOA (degrees);

*g* is the acceleration due to gravity (m/s^2^);

*V* is the cruising speed (m/s).

Total energy consumption is the sum of the energy consumption in each sub-phase, which is obtained by multiplying the power demand with the duration of time in each sub-phase (Eq. 4). For hovering and landing, the hover time is set as a fixed value. In other sub-phases, the duration of time is calculated by dividing the flying distance by the velocity (Eq. 5). The energy reserve is the product of the power demand while cruising and the additional cruising time required by regulations (Eq. 6). The parameters used for this part of the calculation are provided in Table S1.


(4) $$E_{c}=\sum P_{i}t_{i}$$

(5) $$t_{i}=\frac{d_{i}}{V_{i}}$$

(6) $$E_{r}=P_{crusie}\times t_{add}$$

where *E*_*c*_ and *E*_*r*_ are total energy consumption and the energy reserve, respectively (MJ);

*P*_*i*_ is the power demand of sub-phase *i* (kW);

*t*_*i*_ and *t*_*add*_ are the duration of time in sub-phase *i* and the additional cruising time, respectively (s);

*d*_*i*_ is the flying distance in sub-phase *i* (m);

*V*_*i*_ is the velocity in sub-phase *i* (m/s).

### VTOL component sizing

In this study, the components of structure, energy storage device, and drive system are sized to meet the requirements of range, payload, and power capacities, as calculated in the flying process modeling part. For BE VTOLs, batteries serve as the energy storage device, providing electricity for electric motors to drive the tilt-rotors. For FC VTOLs, fuel cells convert hydrogen stored in tanks into electricity to drive electric motors and tilt-rotors. For ICE VTOLs, fuel from fuel tanks is combusted in internal combustion engines to generate kinetic energy, which is transmitted through mechanical transmission systems to drive tilt-rotors.

**Fig. 7.**
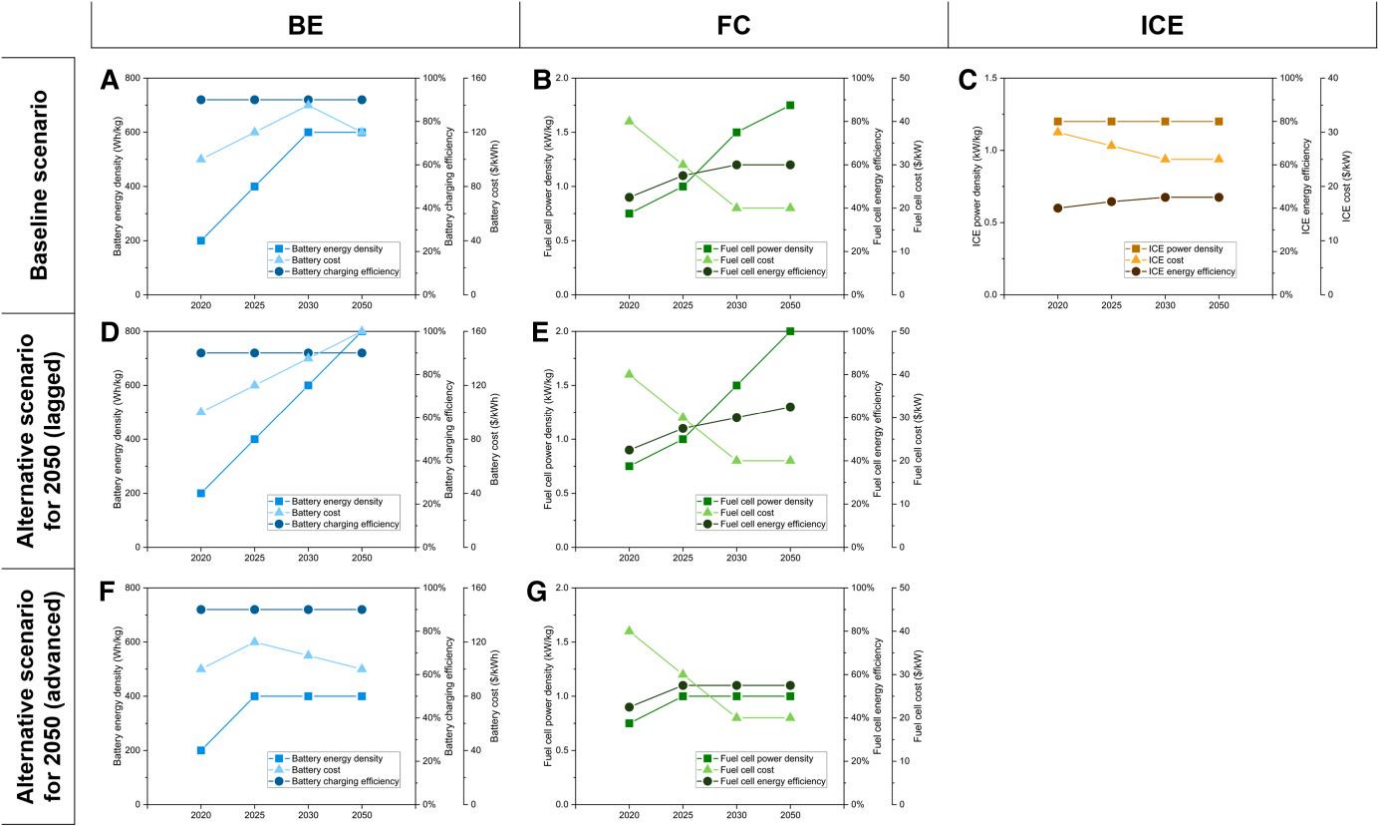
Key assumptions of baseline scenario and alternative scenario for 2050. Subfigures (A)–(C) show the key assumptions for BE, FC, and ICE technologies, respectively, under the baseline scenario. Subfigures (D)–(E) show the key assumptions for BE and FC technologies, respectively, under the alternative scenario for 2050 (lagged case). Subfigures (F)–(G) show the key assumptions for BE and FC technologies, respectively, under the alternative scenario for 2050 (advanced case). BE, battery electric; FC, fuel cell; ICE, internal combustion engine.

The takeoff mass of VTOLs is divided into four parts: structural mass, payload mass, energy storage device mass, and drive system mass (Eq. 7). To meet range requirements, the energy storage device needs to provide adequate energy for both energy consumption and energy reserve (Eq. 8). To meet power capacity requirements, the drive system needs to provide sufficient power for VTOLs to achieve hovering (Eq. 9). The structure of VTOLs mainly refers to body frame, which is assumed to be proportional to the maximum takeoff mass (Eq. 10). For BE VTOLs, battery protection also puts requirements on structure, the mass of which is assumed to be proportional to battery mass (Eq. 10). All parameters used for this part of the calculation are provided in Table S1, with key parameters being provided in Fig. 7. The influences of increased component volume on VTOL performance are not considered in this study, given that a large amount of mechanical structure is eliminated from BE VTOLs and FC VTOLs, leaving extra space.


(7) $$m_{TO}=m_{ES}+m_{DS}+m_{S}+m_{P}$$

(8) $$m_{ES}\times \rho_{energy}=E_{c}+E_{r}$$

(9) $$m_{DS}=\frac{P_{hover}}{\rho_{\;power}}+m_{0_{DS}}$$

(10) $$m_{S}=\{\begin{array}{c} Ratio_{ST}\times m_{TO,}\;\;\;\;\;\;\;\;\;\;\;\;\;\;\;\;\;\;\;\;(FC\;or\;ICE)\\ Ratio_{ST}\times m_{TO}+Ratio_{SB}\times m_{ES},\;\;(BE) \end{array}$$

where *m*_*ES*_ is the energy storage device mass (kg);

*m*_*DS*_ is the drive system mass (kg);

*m*_*S*_ is the structural mass (kg);

*m*_*P*_ is the payload (kg);

$m_{0_{DS}}$ is the initial mass of the drive system (kg);

*ρ*_*energy*_ is the energy density of energy storage devices or fuels (Wh/kg or MJ/kg);

*ρ*_*power*_ is the power density of the drive system (kW/kg);

*Ratio*_*ST*_ is the ratio of the mass of structure for VTOL body frame to the takeoff mass;

*Ratio*_*SB*_ is the ratio of the mass of the structure for battery protection to the battery mass.

### TCO and ROI calculation

For operators, the TCO of VTOLs consists of VBM; powertrain; energy; piloting; infrastructure; taxes, fees, and insurance (TFI); maintenance costs; and the salvage value. For every year in a VTOL's life, the yearly cost (i.e. the sum of all costs occurring in one year) is discounted to the present value using the discount rate (Eq. 11). The TCO is further levelized by dividing the sum of the present values of yearly costs by the number of passengers carried and the total flying distance (Eq. 12).


(11) $$PYC_{t}=\frac{\sum C_{\;jt}}{{\left(1+DR\right)}^{t}}$$

(12) $$TCO=\{\begin{array}{c} \frac{\sum_{t=1}^{VL}\;PYC_{t}}{(N_{P}-1)\times Ratio_{P}\times V\times T\times VL},\;\;(N_{p}>1)\\ \frac{\sum_{t=1}^{VL}\;PYC_{t}}{N_{P}\times Ratio_{P}\times V\times T\times VL},\;\;\;\;\;\;\;\;(N_{p}=1) \end{array}$$

where *C*_*jt*_ is the cost of item *j* that occurs in year *t* ($);

*PYC*_*t*_ is the present value of the yearly cost in year *t* ($);

*DR* is the discount rate;

*TCO* is the total cost of ownership ($/passenger-km);

*VL* is the VTOL life (yr);

*N*_*P*_ is the number of passengers (including the pilot, except for one-seater VTOLs);

*Ratio*_*P*_ is the load factor;

*T* is the yearly operation time (h).

The VBM cost, which occurs in the first year of VTOL life, is calculated using an empirical formula (Eq. 13) (15). The powertrain cost, which occurs when VTOLs are manufactured or the powertrain system needs to be replaced (e.g. when batteries reach their life limit), is the sum of the energy storage device cost and the drive system cost (Eq. 14). The energy cost is the product of the energy price and the total energy consumption (Eq. 15). The TFI cost is assumed to be proportional to the direct operation cost, which comprises energy, piloting, and infrastructure costs (Eq. 16). In this study, the air traffic management cost is considered to be included in the TFI cost, as the air traffic management services are assumed to be provided by the government. Considering that the air traffic management is essential to the safety of VTOLs, reduction in this cost is not expected in this study. The maintenance cost consists of two parts: a fixed maintenance cost related to the VBM cost and a variable maintenance cost that is the product of flight time and variable maintenance cost (Eq. 17). The salvage value, which occurs in the last year of VTOL life, is calculated by multiplying the VBM cost by a fixed ratio (Eq. 18). The methods for estimating infrastructure cost and fuel price are introduced in subsequent sections.


(13) $$C_{VBM_{1}}=C_{VBM-B}+C_{VBM-V}\times (m_{TO}-m_{0_{VBM}})$$

(14) $$C_{P_{t}}=\{\begin{array}{c} m_{ES}\times UC_{ES}+P_{DS}\times UC_{DS},\;\;\;\;\;\;(FC\;or\;ICE)\\ \underset{k}{\sum }\;(Q\times UC_{ES})+P_{DS}\times UC_{DS},\;\;(BE) \end{array}$$

(15) $$C_{E_{t}}=p_{E}\times \frac{E_{c}}{R}\times V\times T\times (1+Ratio_{DE})$$

(16) $$C_{TFI_{t}}=Ratio_{TFI}\times (C_{E_{t}}+C_{Plt_{t}}+C_{I_{t}})$$

(17) $$C_{M_{t}}=Ratio_{M}\times C_{VBM_{1}}+T\times UC_{VM}$$

(18) $$C_{SV_{VL}}=-Ratio_{SV}\times C_{VBM_{1}}$$

where $C_{VBM_{1}}$, $C_{P_{t}}$, $C_{E_{t}}$, $C_{Plt_{t}}$, $C_{I_{t}}$, $C_{TFI_{t}}$, $C_{M_{t}}$, and $C_{SV_{VL}}$ are VBM cost, powertrain cost, energy cost, piloting cost, infrastructure cost, TFI cost, maintenance cost, and the salvage value, respectively, in year *t* ($);

*C*_*VBM*−*B*_ and *C*_*VBM*−*V*_ are base and variable VBM costs ($ and $/kg);

$m_{0_{VBM}}$ is the baseline VTOL structural mass (kg);

*P*_*DS*_ is the power of the drive system (kW);

*UC*_*ES*_ is the energy storage device cost ($/kg or $/kWh);

*UC*_*DS*_ is the drive system cost ($/kW);

*Q* is the battery capacity (kWh);

*p*_*E*_ is the energy price ($/kWh for electricity, $/kg for hydrogen, and $/L for gasoline and sustainable aviation fuel);

*R* is the range traveled per mission (km);

*Ratio*_*DE*_ is the dead-end ratio;

*Ratio*_*TFI*_ is the ratio of the TFI cost to the direct operation cost;

*Ratio*_*M*_ is the ratio of the fixed maintenance cost to the VBM cost;

*UC*_*VM*_ is the variable maintenance cost ($/h);

*Ratio*_*SV*_ is the ratio of the salvage value to the VBM cost.

The ROI is calculated by dividing the average yearly profit by the total cost (Eq. 19). The yearly profit is obtained by subtracting the yearly cost from the yearly revenue (Eq. 20). The parameters used for this part of the calculation are provided in Table S1.


(19) $$ROI=\frac{\frac{1}{VL}\times \sum_{t=1}^{VL}\;PYP_{t}}{\sum_{t=1}^{VL}\;PYC_{t}}$$

(20) $$PYP_{t}=\{\begin{array}{c} \frac{\;p_{P}\times (N_{P}-1)\times Ratio_{P}\times V\times T}{{\left(1+DR\right)}^{t}}-PYC_{t},\;\;(N_{P}>1)\\ \frac{\;p_{P}\times N_{P}\times Ratio_{P}\times V\times T}{{\left(1+DR\right)}^{t}}-PYC_{t},\;\;\;\;\;\;\;\;(N_{P}=1) \end{array}$$

where *ROI* is the return on investment;

*PYP*_*t*_ is the present value of yearly profit ($);

*p*_*P*_ is the passenger fare ($/passenger-km).

### Infrastructure cost

The infrastructure cost consists of vertiport construction, vertiport operation, refueling facility construction, and refueling facility operation costs (Eq. 21). Vertiport and refueling facility construction costs are amortized over their lifetimes. The refueling facility operation cost is calculated by summing up maintenance, labor, and electricity use costs (Eq. 22). The maintenance cost is assumed to be proportional to the refueling facility construction cost (Eq. 23). The labor cost is calculated by multiplying the number of staff, yearly operation time, and unit labor cost (Eq. 24). The electricity use cost is assumed to be higher for a hydrogen refueling facility, due to the higher electricity demand from its devices. The detailed assumptions are provided in Table S1.


(21) $$C_{I_{t}}=\frac{C_{VPC_{0}}}{VPL}+C_{VPO_{t}}+\frac{C_{RFC_{0}}}{RFL}+C_{RFO_{t}}$$

(22) $$C_{RFO_{t}}=C_{RFM_{t}}+C_{LBR_{t}}+C_{EU_{t}}$$

(23) $$C_{RFM_{t}}=C_{VPC_{0}}\times Ratio_{RFM}$$

(24) $$C_{LBR_{t}}=N_{LBR}\times T\times UC_{LBR}$$

Where $C_{VPC_{0}}$ and $C_{RFC_{0}}$ are vertiport and refueling facility construction costs, respectively ($);

$C_{VPO_{t}}$ , $C_{RFO_{t}}$, $C_{RFM_{t}}$, $C_{LBR_{t}}$, and $C_{EU_{t}}$ are vertiport operation cost, refueling facility operation cost, refueling facility maintenance cost, refueling facility labor cost, and refueling facility electricity use cost, respectively, in year *t* ($);

*VPL* and *RFL* are vertiport and refueling facility life, respectively (yr);

*Ratio*_*RFM*_ is the ratio of refueling facility maintenance cost to refueling facility construction cost;

*N*_*LBR*_ is the number of staff;

*UC*_*LBR*_ is the unit labor cost ($/h).

### Energy price

This study estimates energy price by using a levelized cost approach, which is obtained by dividing the present value of total cost by energy production volume (Eq. 25). The total cost of energy production consists of total capital cost, operation and maintenance cost, feedstock cost, decommissioning and waste management cost, and storage and distribution cost. The costs of natural gas and renewable electricity, which are the common basis for FE and RE price calculations, are assumed based on the authors’ estimates and tested with sensitivity analysis. The costs of other items are based on existing estimates, as explained in Table S1.


(25) $$p_{k}=\frac{\sum (C_{CAPEX_{t}}+C_{OPEX_{t}}+C_{F_{t}}+C_{DW_{t}}+C_{SD_{t}})\times {\left(1+DR\right)}^{-t}}{\sum PV_{k}\times {\left(1+DR\right)}^{-t}}$$

where *p*_*k*_ is the price of energy *k* ($/kWh for electricity, $/kg for hydrogen, and $/L for gasoline and sustainable aviation fuel);

$C_{CAPEX_{t}}$ , $C_{OPEX_{t}}$, $C_{F_{t}}$, $C_{DW_{t}}$, and $C_{SD_{t}}$ are total capital cost, operation and maintenance cost, feedstock cost, decommissioning and waste management cost, and storage and distribution cost, respectively, in year *t* ($);

*PV*_*k*_ is the annual production volume of energy *k* (MWh for electricity, *t* for hydrogen, and L for gasoline and sustainable aviation fuel).

### Sensitivity analysis

In this study, the sensitivity of the TCO estimates to changes in VTOL technical parameters (i.e. cruising speed, cruising altitude, energy reserve amount, energy density for BE, and power density for FC and ICE) and cost parameters (i.e. base VBM cost, energy price, energy supplementing facility construction cost, piloting cost, TFI cost, battery cost for BE, fuel cell cost for FC, and internal combustion engine cost for ICE) is analyzed using Monte Carlo simulation. The sensitivity of the TCO estimates to changes in cruising speed, cruising altitude, and energy reserve amount is used in Figs. 3 and 4, for their considerable uncertainties in VTOL design. Sensitivity of the TCO estimates to changes in the rest of the selected parameters is provided in the Supplementary material. A total of 50,000 simulations are conducted and, in every simulation, random values varying between the lower and upper bounds of the parameters are generated using a triangular distribution. The TCO and ROI are correspondingly recalculated for each simulation. The results of the sensitivity analysis are in the 90% confidence interval.

## Supplementary Material

pgad019_Supplementary_Data

## Data Availability

The authors declare that all data supporting the findings of the present study are available within the paper and its Supplementary material.
