# Supplementary material for: Flying cars economically favor battery electric over fuel cell and internal combustion engine
Source: PNAS Nexus. 2023 Mar 14;2(3):pgad019. doi: 10.1093/pnasnexus/pgad019 (PMC10013336; doi:10.1093/pnasnexus/pgad019)
Supplement: pgad019_Supplementary_Data [file pgad019_supplementary_data.doc]

**
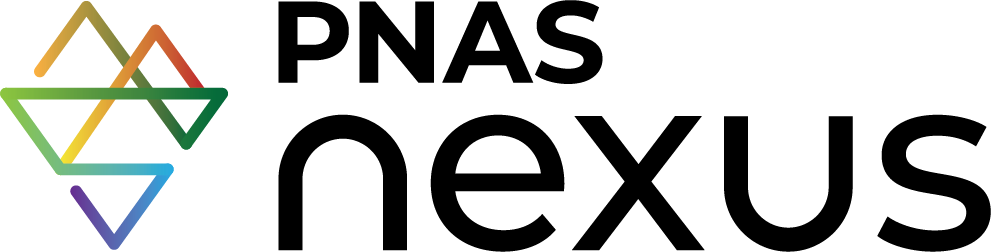
**

**Supplementary Information for**

Flying Cars Economically Favor Battery Electric over Fuel Cell and Internal Combustion Engine

Ming Liu, Han Hao, Zhenhong Lin, Xin He, Yuping Qian, Xin Sun, Jingxuan Geng, Zongwei Liu, Fuquan Zhao

Corresponding author: Han Hao

Email: [hao@tsinghua.edu.cn](mailto:hao@tsinghua.edu.cn)

**This PDF file includes:**

Supplementary text

Figures S1 to S76

Tables S1 to S21

SI References

**Supplementary Information Text**

**Justifications of FE and RE Scenarios**

In this study, the FE and RE scenarios are artificial hypothetical scenarios, which don’t represent any realistic situations but only for theoretical calculation. The reason for making such assumptions is that by using the same source of primary energy as the starting point of energy supply, either renewable or fossil, all technology pathways can be compared on the same basis. In reality, however, the electricity used by VTOLs is likely to be a mix of renewable-based and fossil-based electricity. This is also the case for hydrogen, which can be a mix of renewable-based green hydrogen and fossil-based gray hydrogen; and for liquid fuels, which can be a mix of conventional petroleum-based liquid fuels and renewable-based power-to-liquids. Incorporating these mixes into the analysis, not only for electricity, but also for hydrogen and liquid fuels, could make the analysis too complicated. Instead, by comparing these options using the same starting point of primary energy, the comparison can be more transparent and easier to follow.

**Justifications of Assumptions in Baseline Scenario**

In baseline scenario, assumptions on the technology development in particular years (*i.e.*, 2020, 2025, 2030, and 2050) are made by the authors, with references providing key data. Here we assume that VTOLs will utilize the battery or fuel cell technology with the highest achievable energy density or power density in particular years. For battery technology, in 2020 and 2025, VTOLs are expected to use Nickel Cobalt Manganese (NCM) battery with 200 and 400 Wh/kg energy density, respectively. In 2030 and 2050, solid-state battery with 600 Wh/kg energy density is expected to be used in VTOLs. For fuel cell technology, Proton Exchange Membrane Fuel Cell (PEMFC) is expected to be used in VTOLs. The power density and fuel cell efficiency are assumed to be gradually increased with the development of light-weighting technologies, such as utilizing aluminum as bipolar plate material, and high-temperature PEMFC technology.

**Justifications of Assumptions in Alternative Scenario for 2050**

In this research, the “lagged” and “advanced” scenarios for 2050 are assumed by the authors, in order to show possible technology development situations other than the baseline scenario in the long term. The reason why we set these scenarios for 2050 is that the technology development situation in the long term is hard to predict, with high uncertainty; thus, showing the results under baseline scenario only might be misleading.

The “lagged” scenario for 2050 represents the situation that (1) high-energy-density battery technology (e.g., solid-state battery) or advanced fuel cell technology (*e.g.*, high-temperature PEMFC) are not realized or (2) high-energy-density battery technology and high-power-density fuel cell technology can’t achieve large-scale utilization, due to potentially low battery or fuel cell life, high cost, etc. Therefore, in this scenario, we assume that the technological level of BE or FC in 2030 and 2050 shows no improvements compared with that in 2025 (2020, 2025 scenarios remain the same as baseline scenario). Battery cost is assumed to be further reduced, while fuel cell cost remains the same as the baseline assumptions.

The “advanced” scenario for 2050 represents the situation that disruptive technological innovation, such as lithium-sulfur battery with 800 Wh/kg energy density and high-temperature PEMFC technology, makes breakthroughs and can achieve large-scale utilization. Therefore, in this scenario, the technological level of BE and FC in 2050 is higher than that in baseline scenario. Battery cost is assumed to be higher with higher energy density, while fuel cell cost remains the same as the baseline assumptions.

**Justifications of Assumptions of Lax and Strict Regulation Scenarios**

VTOL technical parameters in this research are assumed mainly based on FAA regulations. The cruising speed is assumed according to FAA regulation Part 91.117. The cruising altitude is assumed based on FAA regulations Part 91.119 and Part 135.203. The energy reserve amount is assumed according to both FAA regulation Part 135.209 and the references.

To further investigate the influence of regulations on the TCO results, different regulation scenarios are assumed. A lax regulation scenario represents the case that laxer regulations are set for VTOLs (*i.e.*, higher cruising speed, lower cruising altitude, less energy reserve amount), which might potentially be applied to inter-city VTOL operation. Considering intra-city VTOL operation, which has more restraints such as buildings, speed control, etc., a strict regulation scenario is assumed, in which lower cruising speed, higher cruising altitude, and larger energy reserve amount are required. Besides, all assumptions in lax and strict regulation scenarios compile with FAA regulations.

**Supplementary Figures**


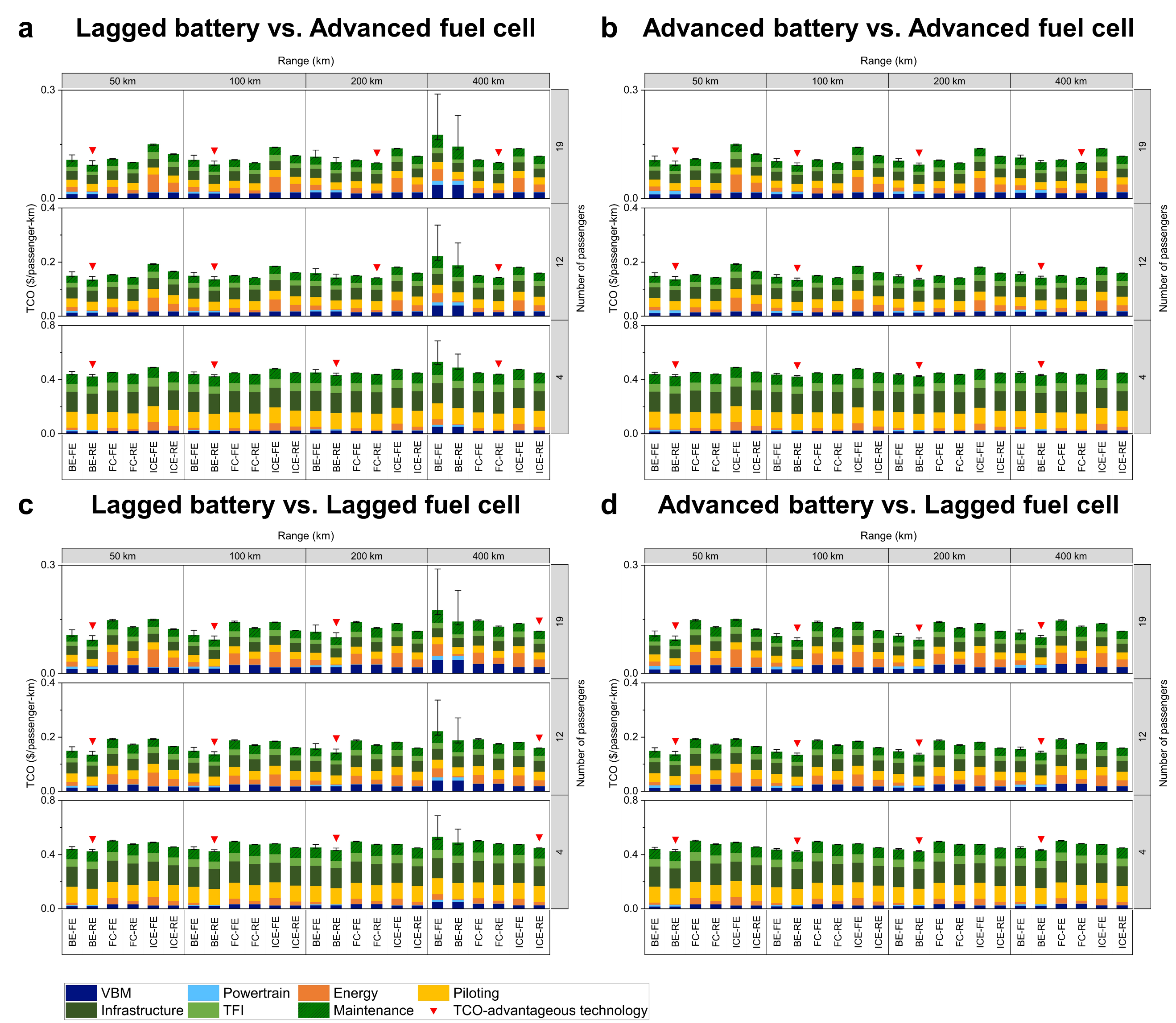


**Fig. S1.** TCO breakdown under alternative battery and fuel cell technology improvement scenarios for 2050. The four subfigures are presented under different technology improvement levels of batteries (horizontal dimension) and fuel cells (vertical dimension): lagged battery vs. advanced fuel cell (a); both advanced (b); both lagged (c); advanced battery vs. lagged fuel cell (d). Each subfigure is divided into twelve application domains distinguished by the number of passengers (vertical dimension) and the range (horizontal dimension). For each application domain, The TCO estimates of six propulsion technologies are shown, with different colors representing different cost components. Salvage value is not shown due to its marginal influence on TCO. BE: Battery Electric; FC: Fuel Cell; ICE: Internal Combustion Engine; FE: Fossil Energy-based; RE: Renewable Energy-based; TCO: Total Cost of Ownership; VBM: VTOL Body Manufacturing; TFI: Taxes, Fees, and Insurance.


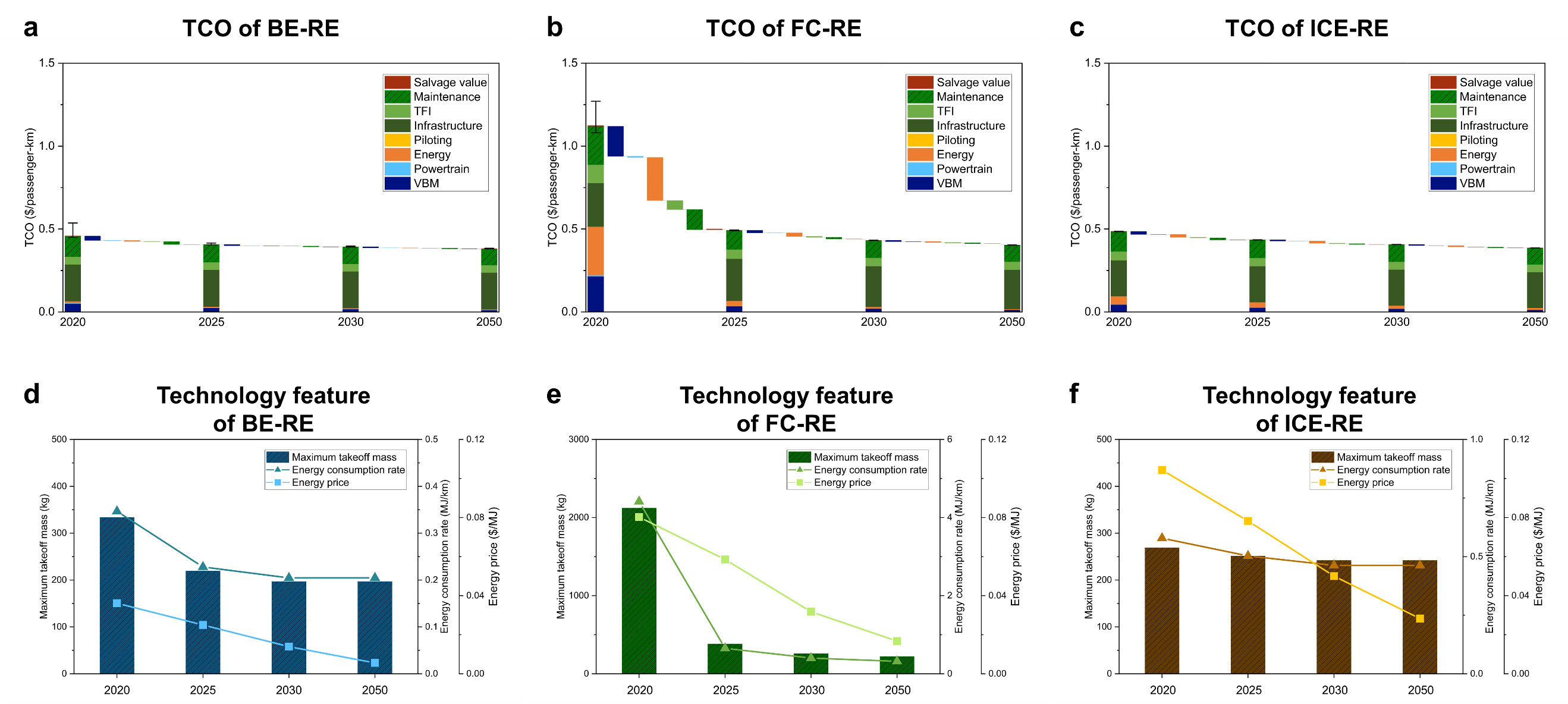


**Fig. S2.** TCO changes and the corresponding contributing factors under the baseline scenario for private flying cars based on RE technologies. Subfigures (a)–(c) show the changes in TCO of BE-RE, FC-RE, and ICE-RE, with the changes broken down into contributions from cost components; subfigures (d)–(f) show the changes in key technology parameters (*i.e.*, maximum takeoff mass, energy consumption rate, and energy price) of BE-RE, FC-RE, and ICE-RE. BE: Battery Electric; FC: Fuel Cell; ICE: Internal Combustion Engine; RE: Renewable Energy-based; TCO: Total Cost of Ownership; VBM: VTOL Body Manufacturing; TFI: Taxes, Fees, and Insurance.


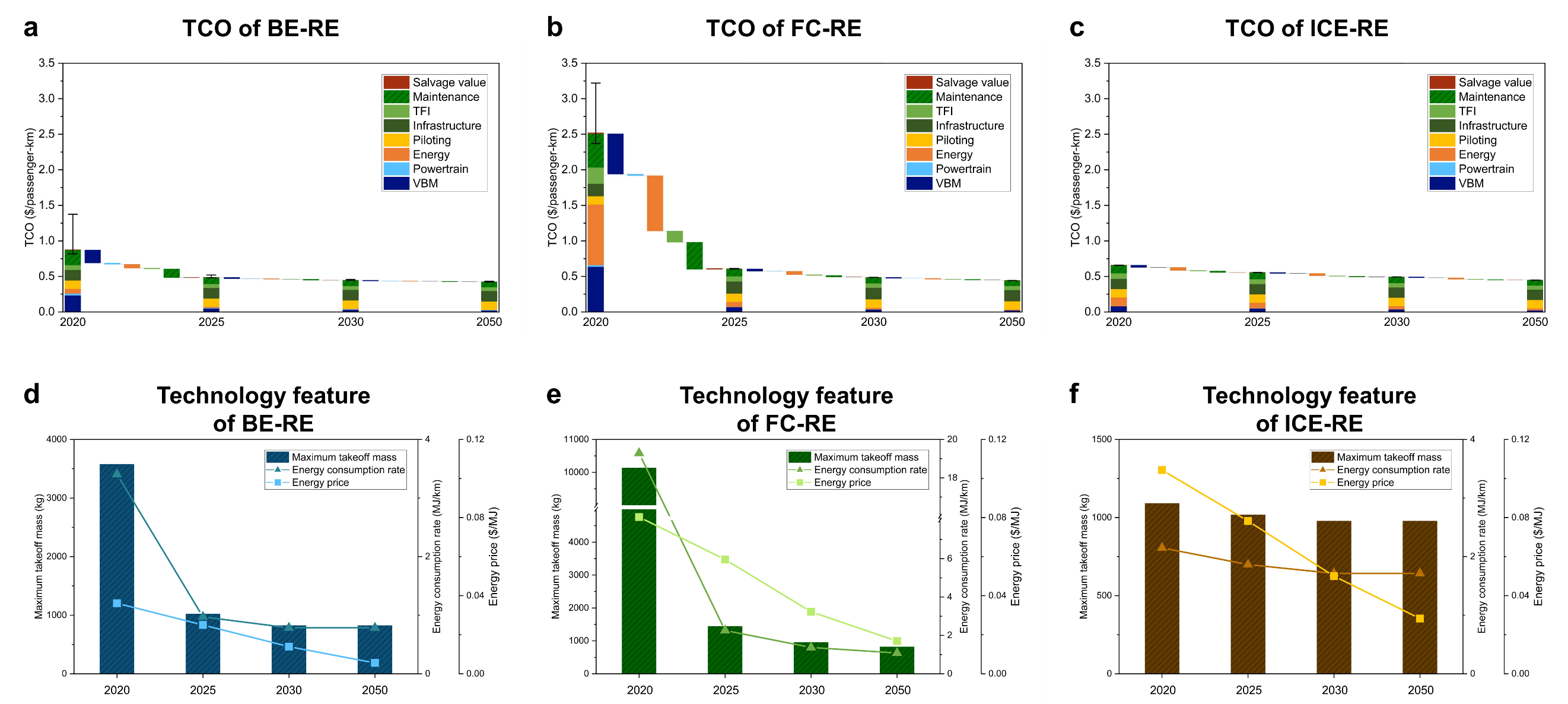


**Fig. S3.** TCO changes and the corresponding contributing factors under the baseline scenario for 200-km-range air taxis based on RE technologies. Subfigures (a)–(c) show the changes in TCO of BE-RE, FC-RE, and ICE-RE, with the changes broken down into contributions from cost components; subfigures (d)–(f) show the changes in key technology parameters (*i.e.*, maximum takeoff mass, energy consumption rate, and energy price) of BE-RE, FC-RE, and ICE-RE. BE: Battery Electric; FC: Fuel Cell; ICE: Internal Combustion Engine; RE: Renewable Energy-based; TCO: Total Cost of Ownership; VBM: VTOL Body Manufacturing; TFI: Taxes, Fees, and Insurance.


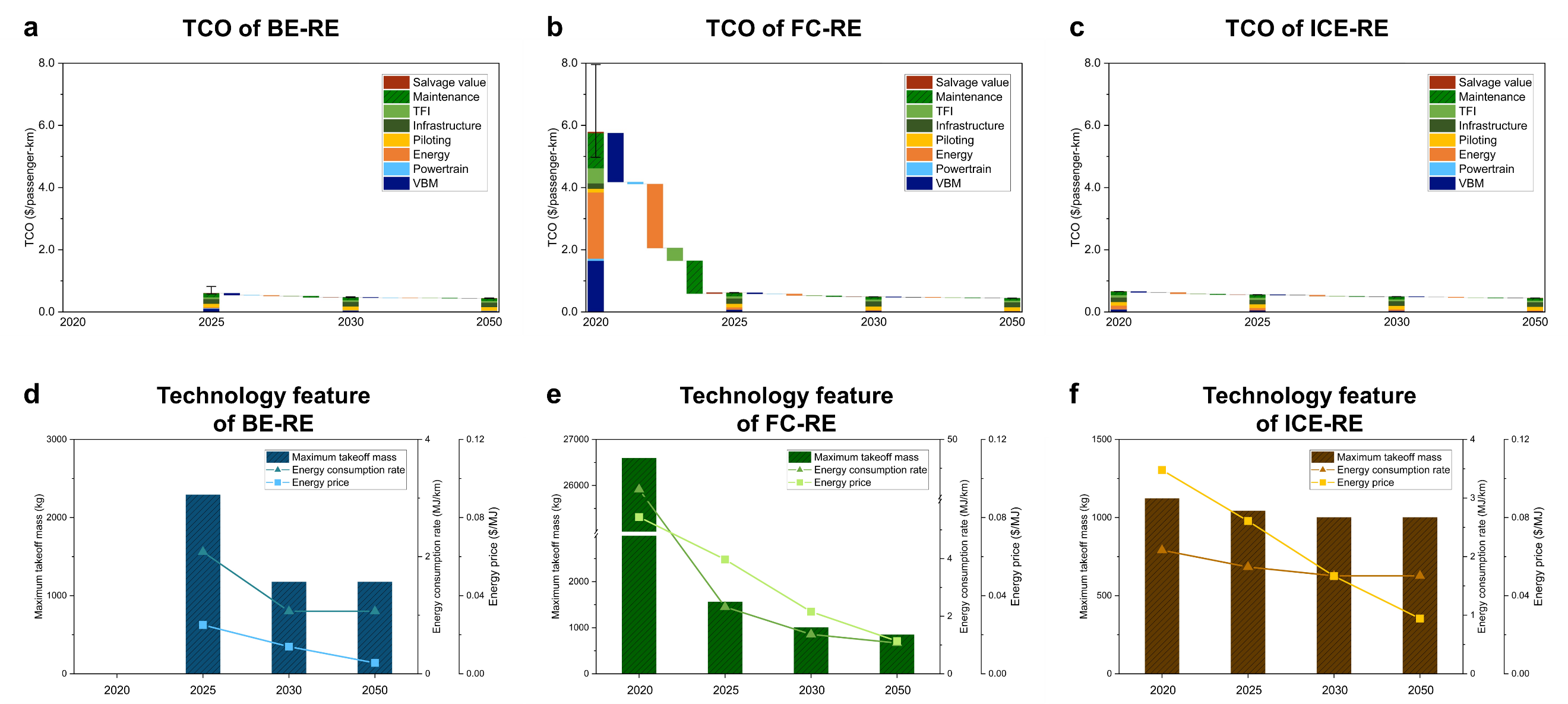


**Fig. S4.** TCO changes and the corresponding contributing factors under the baseline scenario for long-range air taxis based on RE technologies. Subfigures (a)–(c) show the changes in TCO of BE-RE, FC-RE, and ICE-RE, with the changes broken down into contributions from cost components; subfigures (d)–(f) show the changes in key technology parameters (*i.e.*, maximum takeoff mass, energy consumption rate, and energy price) of BE-RE, FC-RE, and ICE-RE. The absence of TCO estimates under certain circumstances indicates technological infeasibility. BE: Battery Electric; FC: Fuel Cell; ICE: Internal Combustion Engine; RE: Renewable Energy-based; TCO: Total Cost of Ownership; VBM: VTOL Body Manufacturing; TFI: Taxes, Fees, and Insurance.


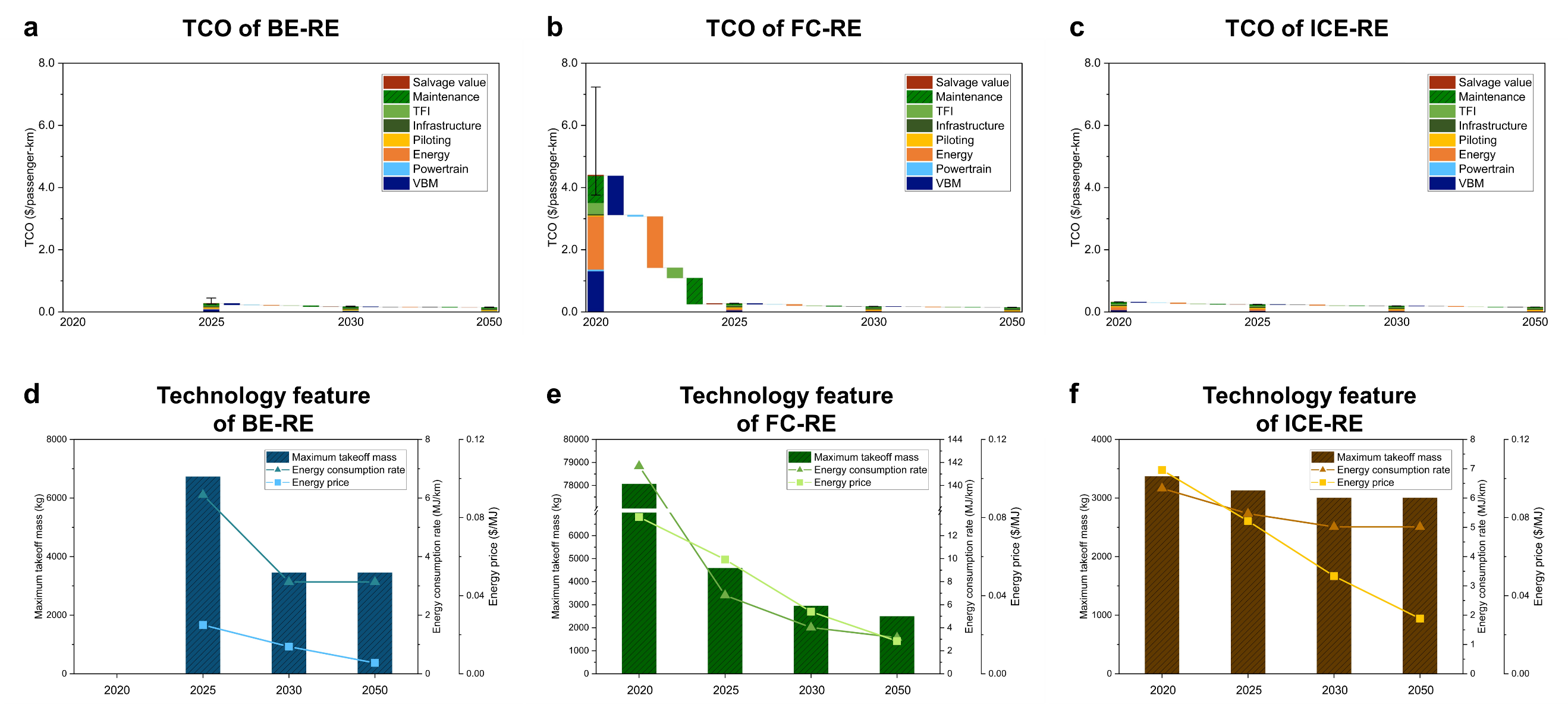


**Fig. S5.** TCO changes and the corresponding contributing factors under the baseline scenario for air vans based on RE technologies. Subfigures (a)–(c) show the changes in TCO of BE-RE, FC-RE, and ICE-RE, with the changes broken down into contributions from cost components; subfigures (d)–(f) show the changes in key technology parameters (*i.e.*, maximum takeoff mass, energy consumption rate, and energy price) of BE-RE, FC-RE, and ICE-RE. The absence of TCO estimates under certain circumstances indicates technological infeasibility. BE: Battery Electric; FC: Fuel Cell; ICE: Internal Combustion Engine; RE: Renewable Energy-based; TCO: Total Cost of Ownership; VBM: VTOL Body Manufacturing; TFI: Taxes, Fees, and Insurance.


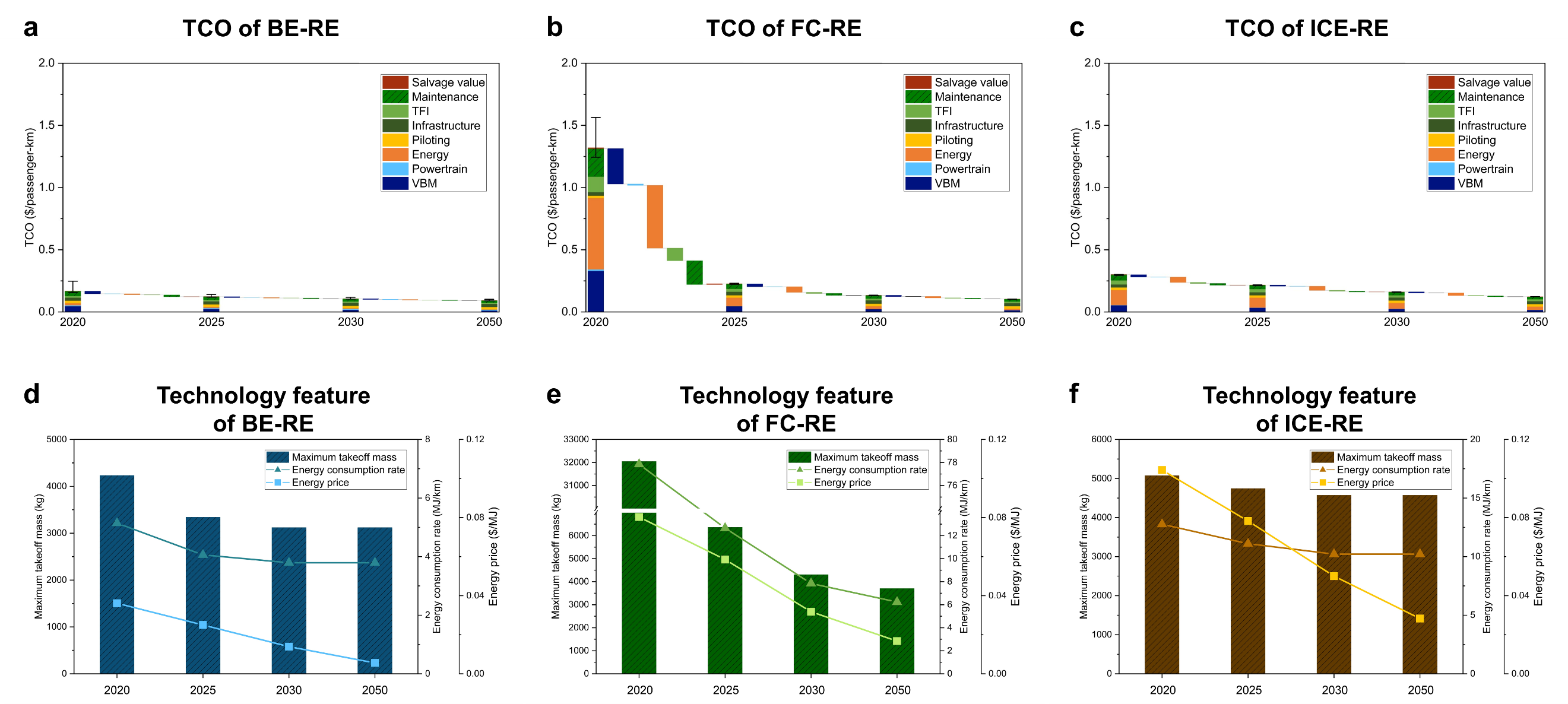


**Fig. S6.** TCO changes and the corresponding contributing factors under the baseline scenario for airport shuttles based on RE technologies. Subfigures (a)–(c) show the changes in TCO of BE-RE, FC-RE, and ICE-RE, with the changes broken down into contributions from cost components; subfigures (d)–(f) show the changes in key technology parameters (*i.e.*, maximum takeoff mass, energy consumption rate, and energy price) of BE-RE, FC-RE, and ICE-RE. BE: Battery Electric; FC: Fuel Cell; ICE: Internal Combustion Engine; RE: Renewable Energy-based; TCO: Total Cost of Ownership; VBM: VTOL Body Manufacturing; TFI: Taxes, Fees, and Insurance.


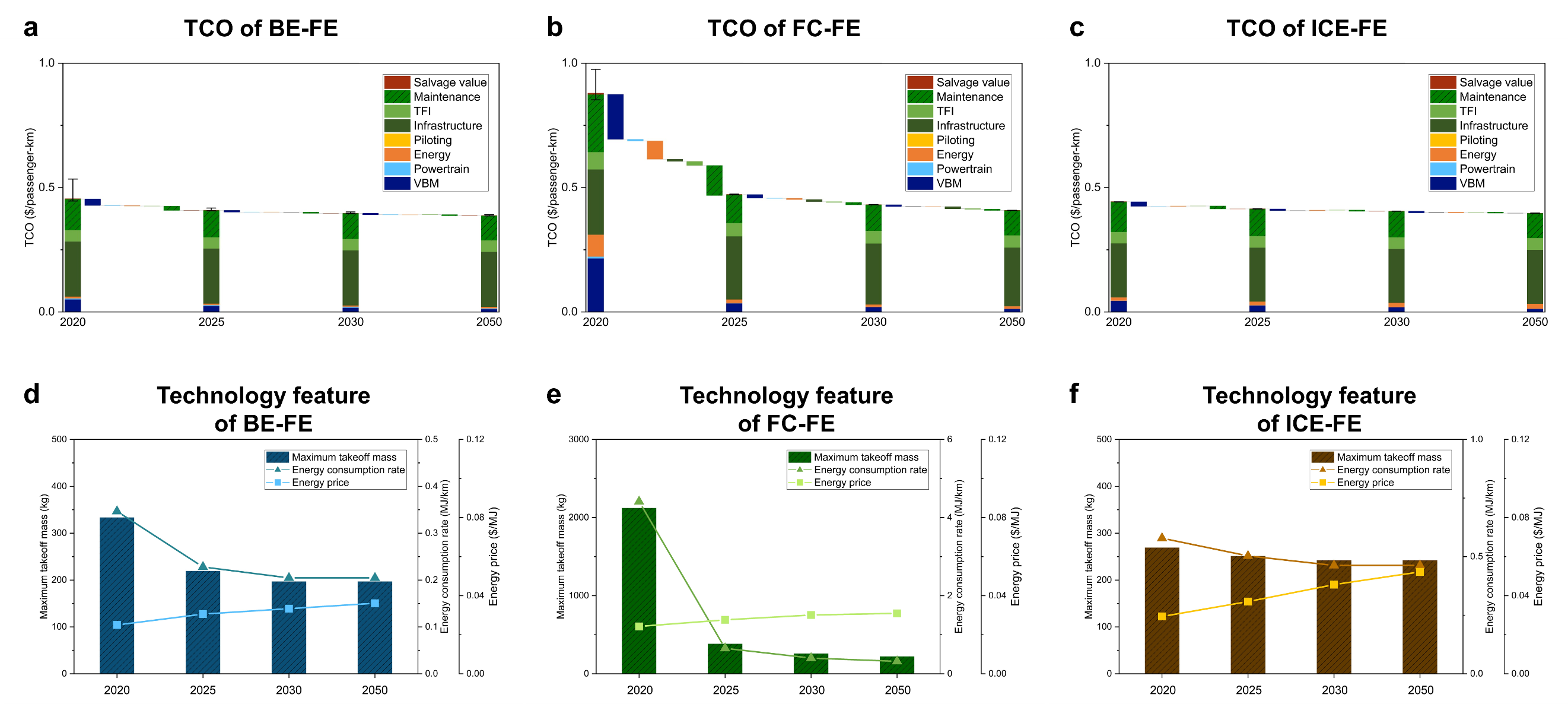


**Fig. S7.** TCO changes and the corresponding contributing factors under the baseline scenario for private flying cars based on FE technologies. Subfigures (a)–(c) show the changes in TCO of BE-FE, FC-FE, and ICE-FE, with the changes broken down into contributions from cost components; subfigures (d)–(f) show the changes in key technology parameters (*i.e.*, maximum takeoff mass, energy consumption rate, and energy price) of BE-FE, FC-FE, and ICE-FE. BE: Battery Electric; FC: Fuel Cell; ICE: Internal Combustion Engine; FE: Fossil Energy-based; TCO: Total Cost of Ownership; VBM: VTOL Body Manufacturing; TFI: Taxes, Fees, and Insurance.


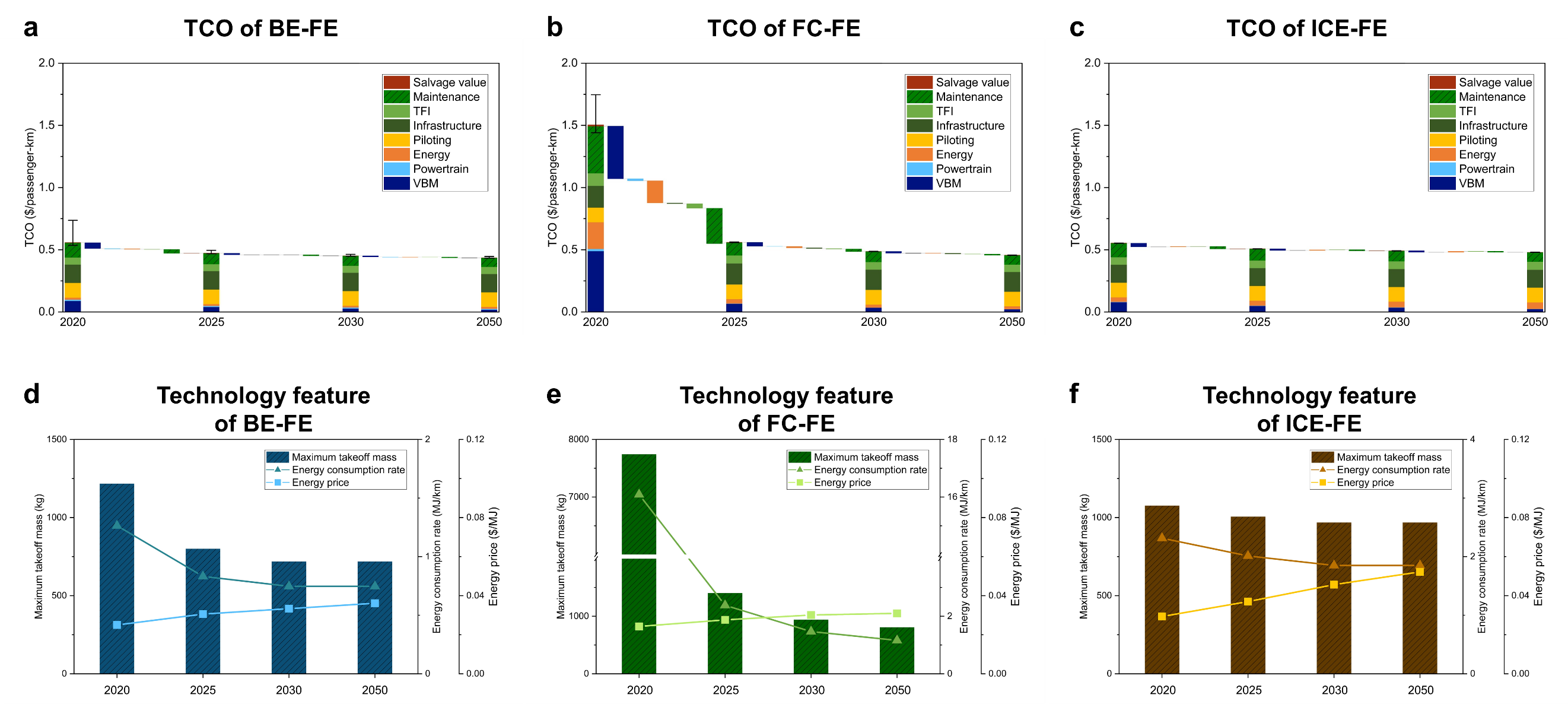


**Fig. S8.** TCO changes and the corresponding contributing factors under the baseline scenario for short-range air taxis based on FE technologies. Subfigures (a)–(c) show the changes in TCO of BE-FE, FC-FE, and ICE-FE, with the changes broken down into contributions from cost components; subfigures (d)–(f) show the changes in key technology parameters (*i.e.*, maximum takeoff mass, energy consumption rate, and energy price) of BE-FE, FC-FE, and ICE-FE. BE: Battery Electric; FC: Fuel Cell; ICE: Internal Combustion Engine; FE: Fossil Energy-based; TCO: Total Cost of Ownership; VBM: VTOL Body Manufacturing; TFI: Taxes, Fees, and Insurance.


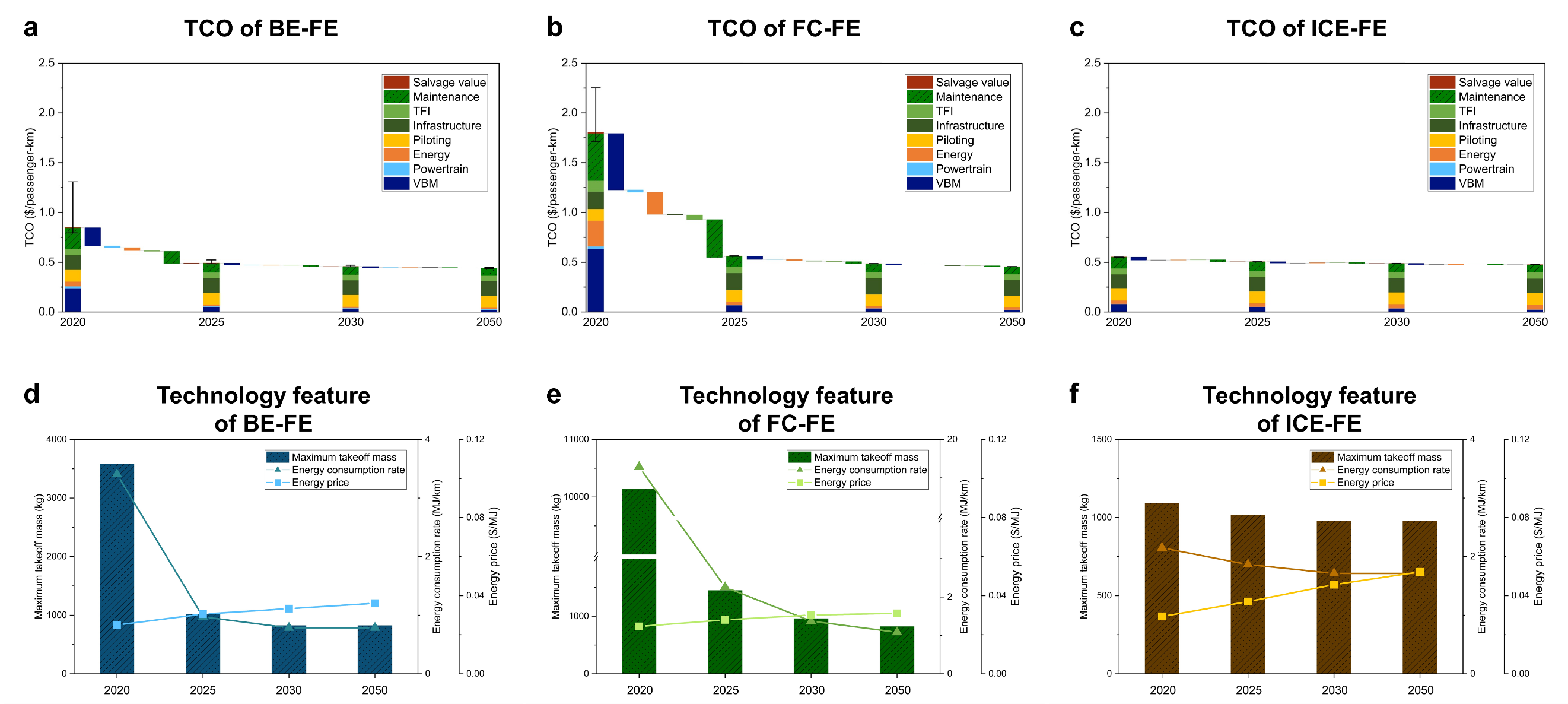


**Fig. S9.** TCO changes and the corresponding contributing factors under the baseline scenario for 200-km-range air taxis based on FE technologies. Subfigures (a)–(c) show the changes in TCO of BE-FE, FC-FE, and ICE-FE, with the changes broken down into contributions from cost components; subfigures (d)–(f) show the changes in key technology parameters (*i.e.*, maximum takeoff mass, energy consumption rate, and energy price) of BE-FE, FC-FE, and ICE-FE. BE: Battery Electric; FC: Fuel Cell; ICE: Internal Combustion Engine; FE: Fossil Energy-based; TCO: Total Cost of Ownership; VBM: VTOL Body Manufacturing; TFI: Taxes, Fees, and Insurance.


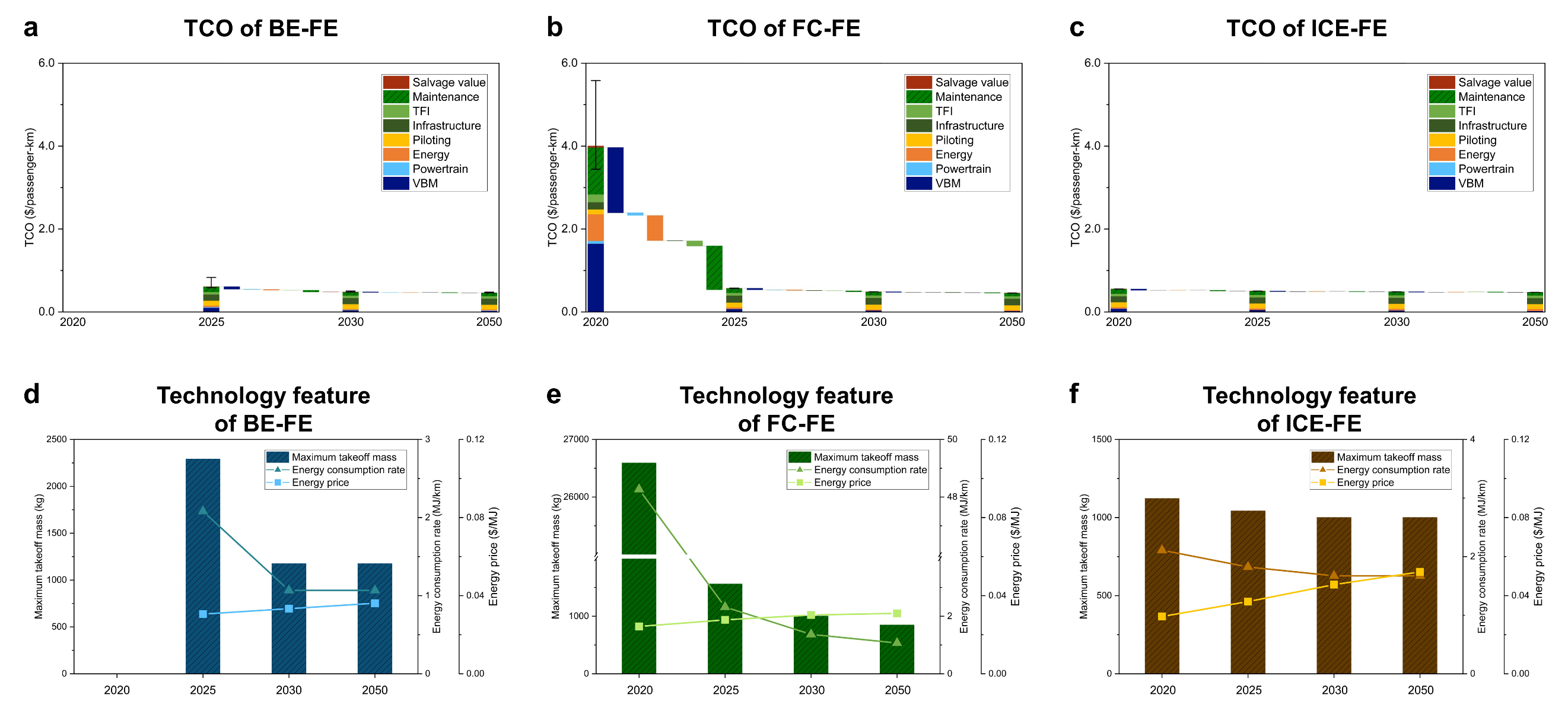


**Fig. S10.** TCO changes and the corresponding contributing factors under the baseline scenario for long-range air taxis based on FE technologies. Subfigures (a)–(c) show the changes in TCO of BE-FE, FC-FE, and ICE-FE, with the changes broken down into contributions from cost components; subfigures (d)–(f) show the changes in key technology parameters (*i.e.*, maximum takeoff mass, energy consumption rate, and energy price) of BE-FE, FC-FE, and ICE-FE. The absence of TCO estimates under certain circumstances indicates technological infeasibility. BE: Battery Electric; FC: Fuel Cell; ICE: Internal Combustion Engine; FE: Fossil Energy-based; TCO: Total Cost of Ownership; VBM: VTOL Body Manufacturing; TFI: Taxes, Fees, and Insurance.


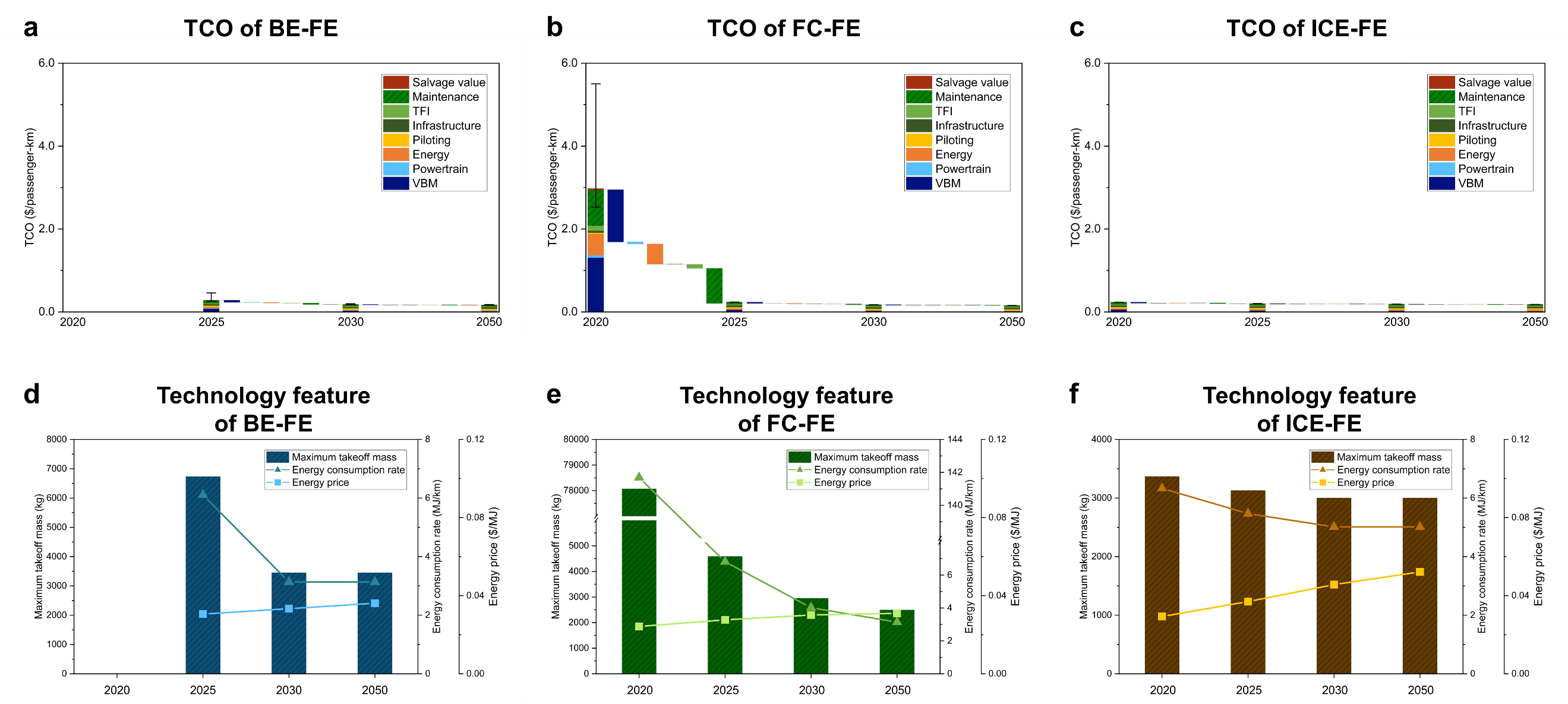


**Fig. S11.** TCO changes and the corresponding contributing factors under the baseline scenario for air vans based on FE technologies. Subfigures (a)–(c) show the changes in TCO of BE-FE, FC-FE, and ICE-FE, with the changes broken down into contributions from cost components; subfigures (d)–(f) show the changes in key technology parameters (*i.e.*, maximum takeoff mass, energy consumption rate, and energy price) of BE-FE, FC-FE, and ICE-FE. The absence of TCO estimates under certain circumstances indicates technological infeasibility. BE: Battery Electric; FC: Fuel Cell; ICE: Internal Combustion Engine; FE: Fossil Energy-based; TCO: Total Cost of Ownership; VBM: VTOL Body Manufacturing; TFI: Taxes, Fees, and Insurance.


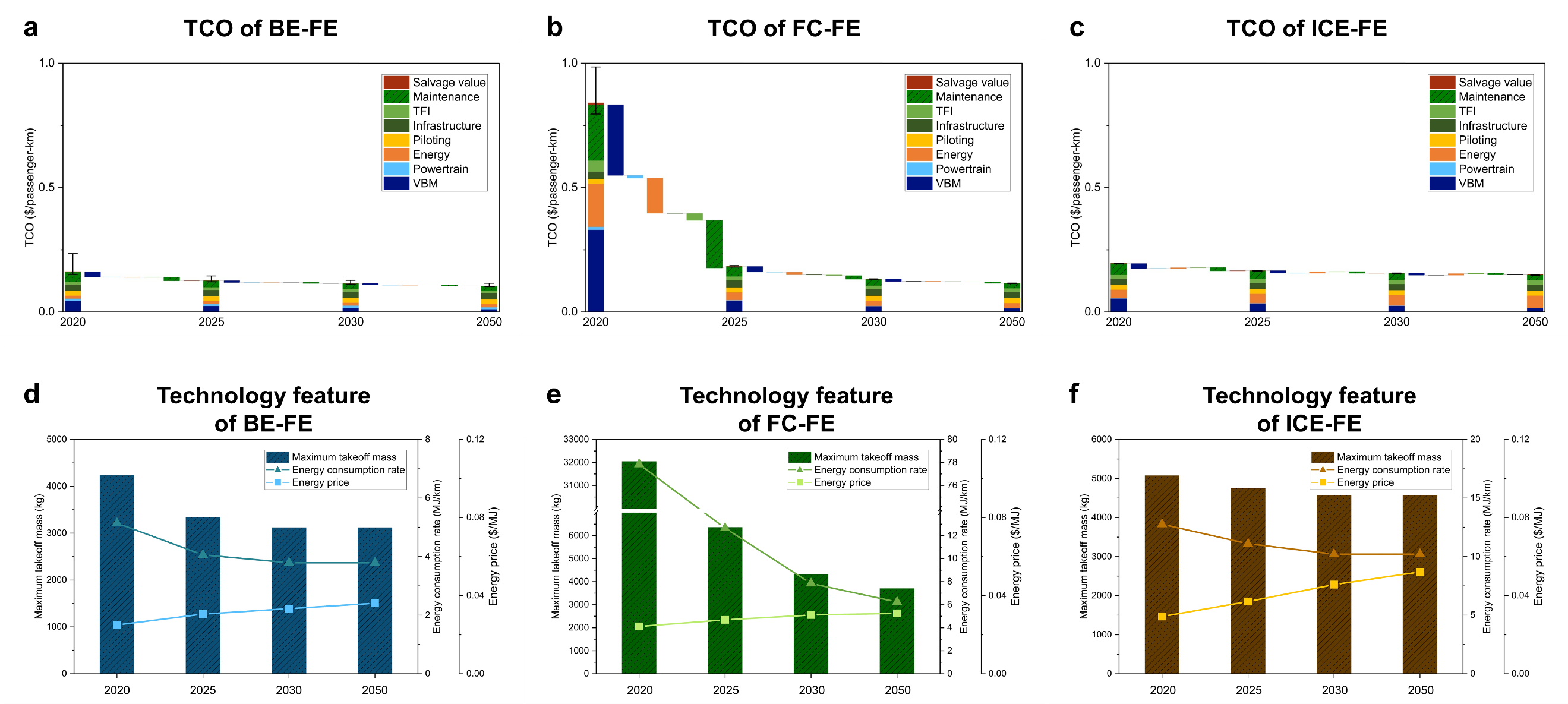


**Fig. S12.** TCO changes and the corresponding contributing factors under the baseline scenario for airport shuttles based on FE technologies. Subfigures (a)–(c) show the changes in TCO of BE-FE, FC-FE, and ICE-FE, with the changes broken down into contributions from cost components; subfigures (d)–(f) show the changes in key technology parameters (*i.e.*, maximum takeoff mass, energy consumption rate, and energy price) of BE-FE, FC-FE, and ICE-FE. BE: Battery Electric; FC: Fuel Cell; ICE: Internal Combustion Engine; FE: Fossil Energy-based; TCO: Total Cost of Ownership; VBM: VTOL Body Manufacturing; TFI: Taxes, Fees, and Insurance.


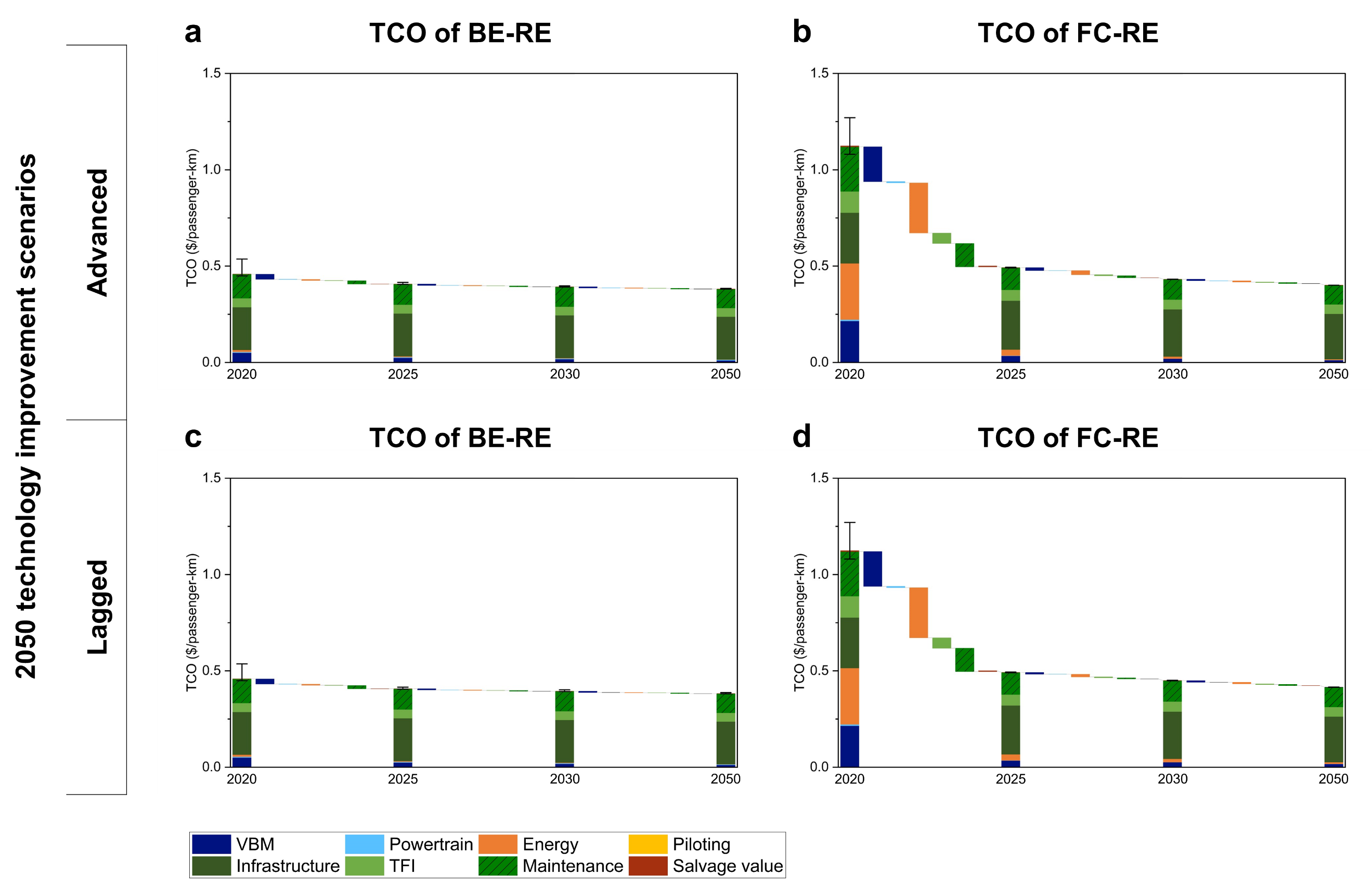


**Fig. S13.** TCO changes and the corresponding contributing factors under the alternative scenarios for private flying cars based on RE technologies. Subfigures (a) and (c) show the changes in TCO of BE-RE with advanced and lagged battery technology development for 2050, with the changes broken down into contributions from cost components. Subfigures (b) and (d) show the changes in TCO of FC-RE with advanced and lagged fuel cell technology development for 2050, with the changes broken down into contributions from cost components. BE: Battery Electric; FC: Fuel Cell; RE: Renewable Energy-based; TCO: Total Cost of Ownership; VBM: VTOL Body Manufacturing; TFI: Taxes, Fees, and Insurance.


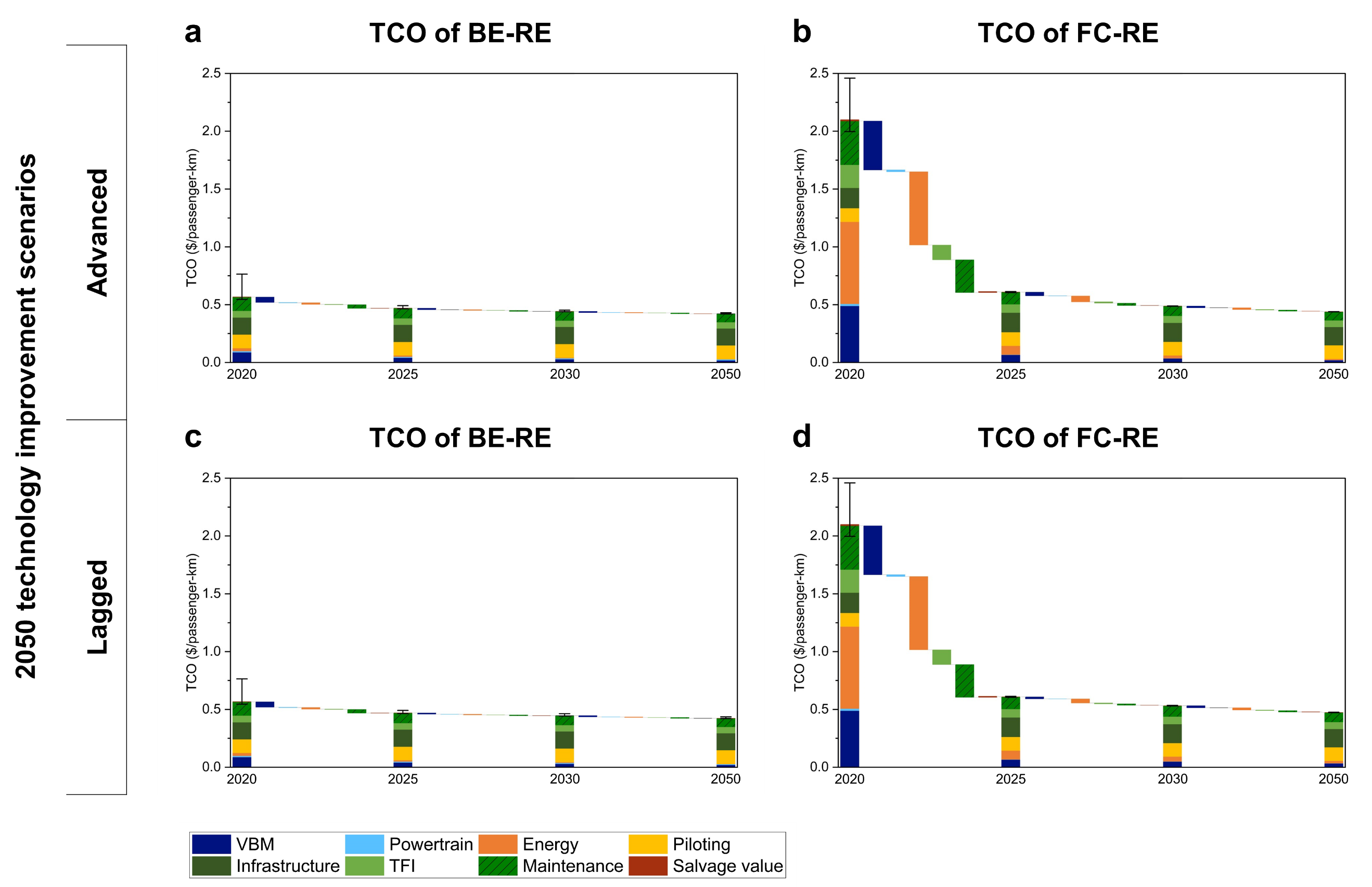


**Fig. S14.** TCO changes and the corresponding contributing factors under the alternative scenarios for short-range air taxis based on RE technologies. Subfigures (a) and (c) show the changes in TCO of BE-RE with advanced and lagged battery technology development for 2050, with the changes broken down into contributions from cost components. Subfigures (b) and (d) show the changes in TCO of FC-RE with advanced and lagged fuel cell technology development for 2050, with the changes broken down into contributions from cost components. BE: Battery Electric; FC: Fuel Cell; RE: Renewable Energy-based; TCO: Total Cost of Ownership; VBM: VTOL Body Manufacturing; TFI: Taxes, Fees, and Insurance.


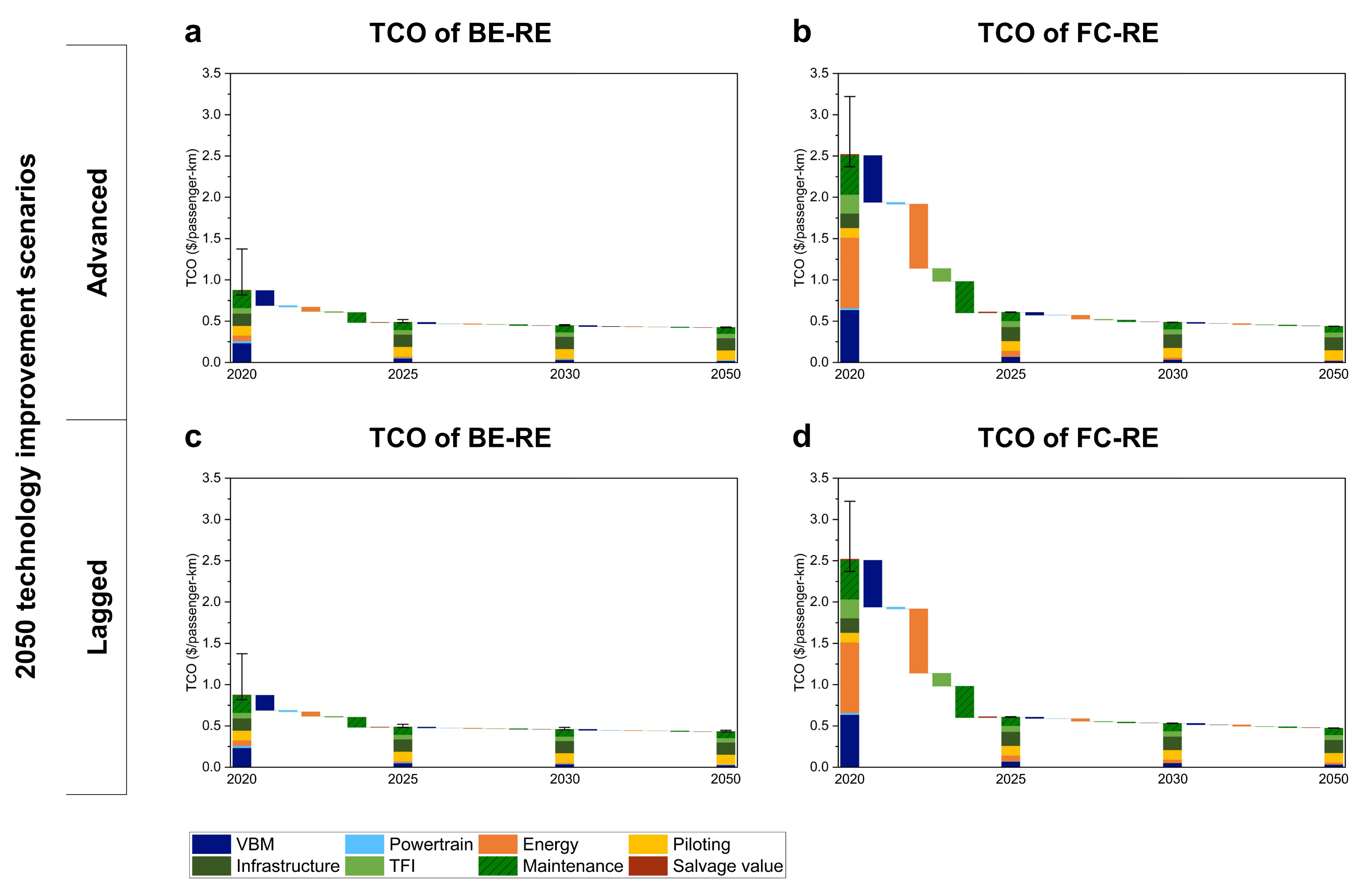


**Fig. S15.** TCO changes and the corresponding contributing factors under the alternative scenarios for 200-km-range air taxis based on RE technologies. Subfigures (a) and (c) show the changes in TCO of BE-RE with advanced and lagged battery technology development for 2050, with the changes broken down into contributions from cost components. Subfigures (b) and (d) show the changes in TCO of FC-RE with advanced and lagged fuel cell technology development for 2050, with the changes broken down into contributions from cost components. BE: Battery Electric; FC: Fuel Cell; RE: Renewable Energy-based; TCO: Total Cost of Ownership; VBM: VTOL Body Manufacturing; TFI: Taxes, Fees, and Insurance.


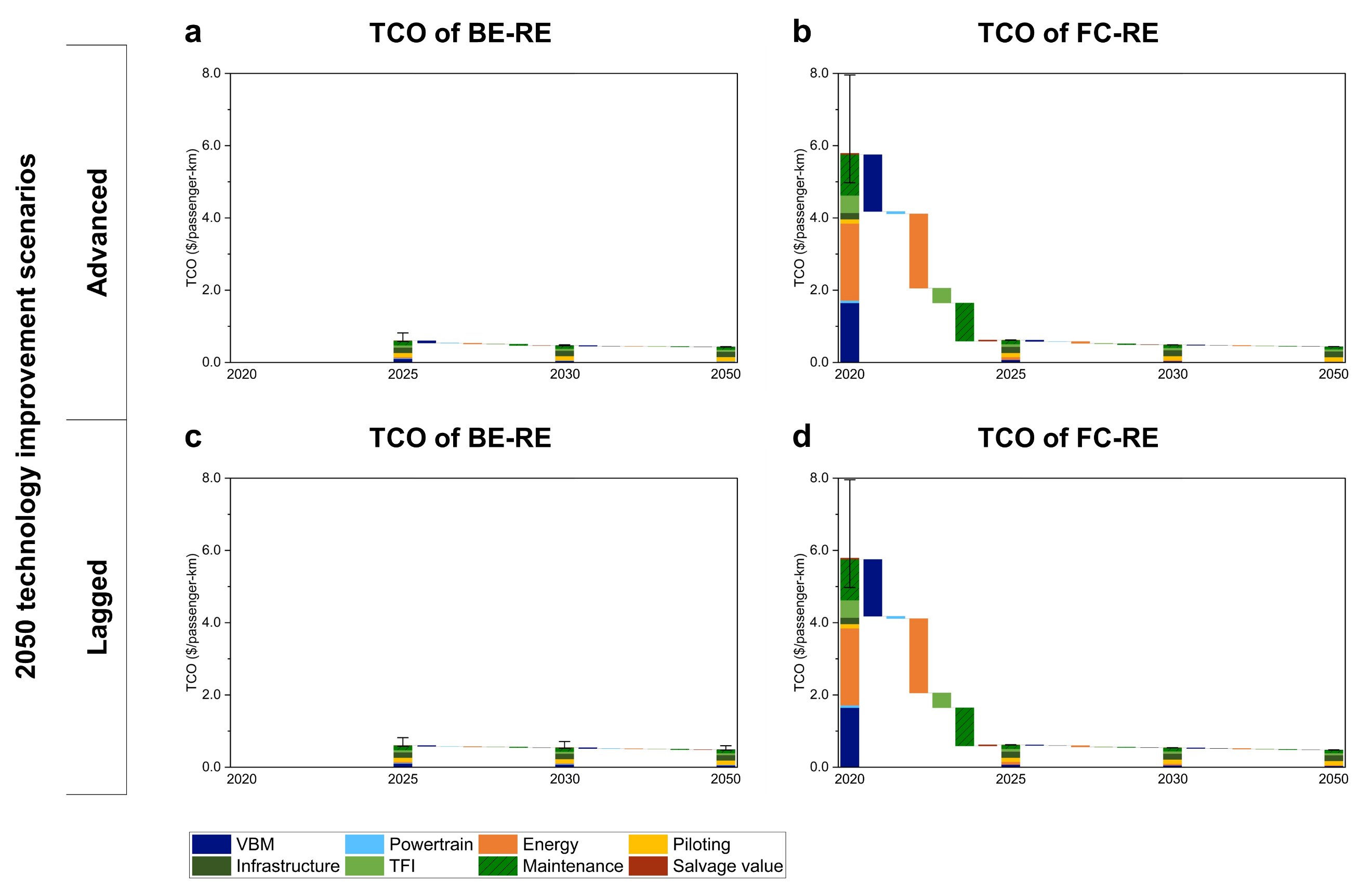


**Fig. S16.** TCO changes and the corresponding contributing factors under the alternative scenarios for long-range air taxis based on RE technologies. Subfigures (a) and (c) show the changes in TCO of BE-RE with advanced and lagged battery technology development for 2050, with the changes broken down into contributions from cost components. Subfigures (b) and (d) show the changes in TCO of FC-RE with advanced and lagged fuel cell technology development for 2050, with the changes broken down into contributions from cost components. BE: Battery Electric; FC: Fuel Cell; RE: Renewable Energy-based; TCO: Total Cost of Ownership; VBM: VTOL Body Manufacturing; TFI: Taxes, Fees, and Insurance.


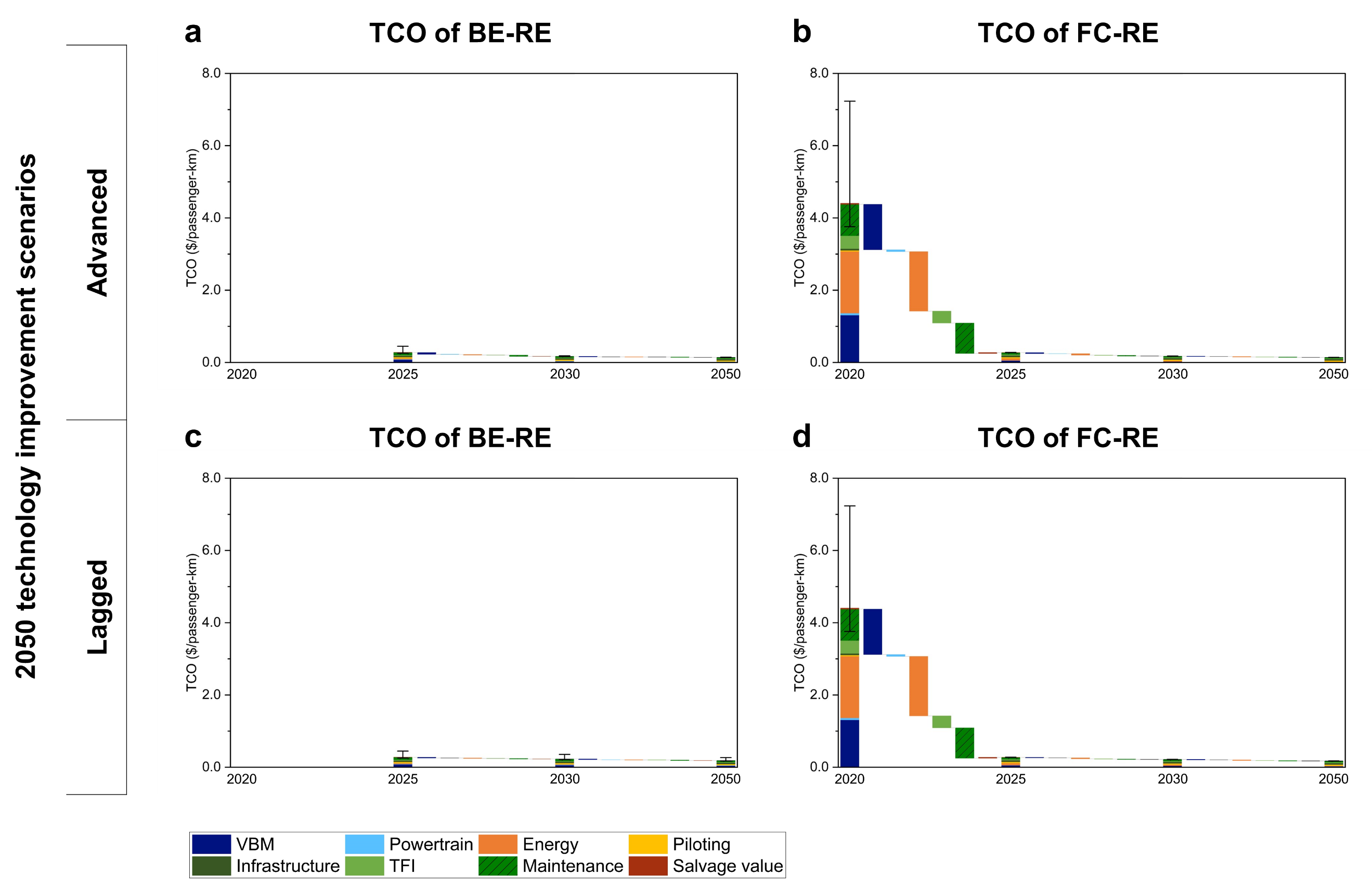


**Fig. S17.** TCO changes and the corresponding contributing factors under the alternative scenarios for air vans based on RE technologies. Subfigures (a) and (c) show the changes in TCO of BE-RE with advanced and lagged battery technology development for 2050, with the changes broken down into contributions from cost components. Subfigures (b) and (d) show the changes in TCO of FC-RE with advanced and lagged fuel cell technology development for 2050, with the changes broken down into contributions from cost components. BE: Battery Electric; FC: Fuel Cell; RE: Renewable Energy-based; TCO: Total Cost of Ownership; VBM: VTOL Body Manufacturing; TFI: Taxes, Fees, and Insurance.


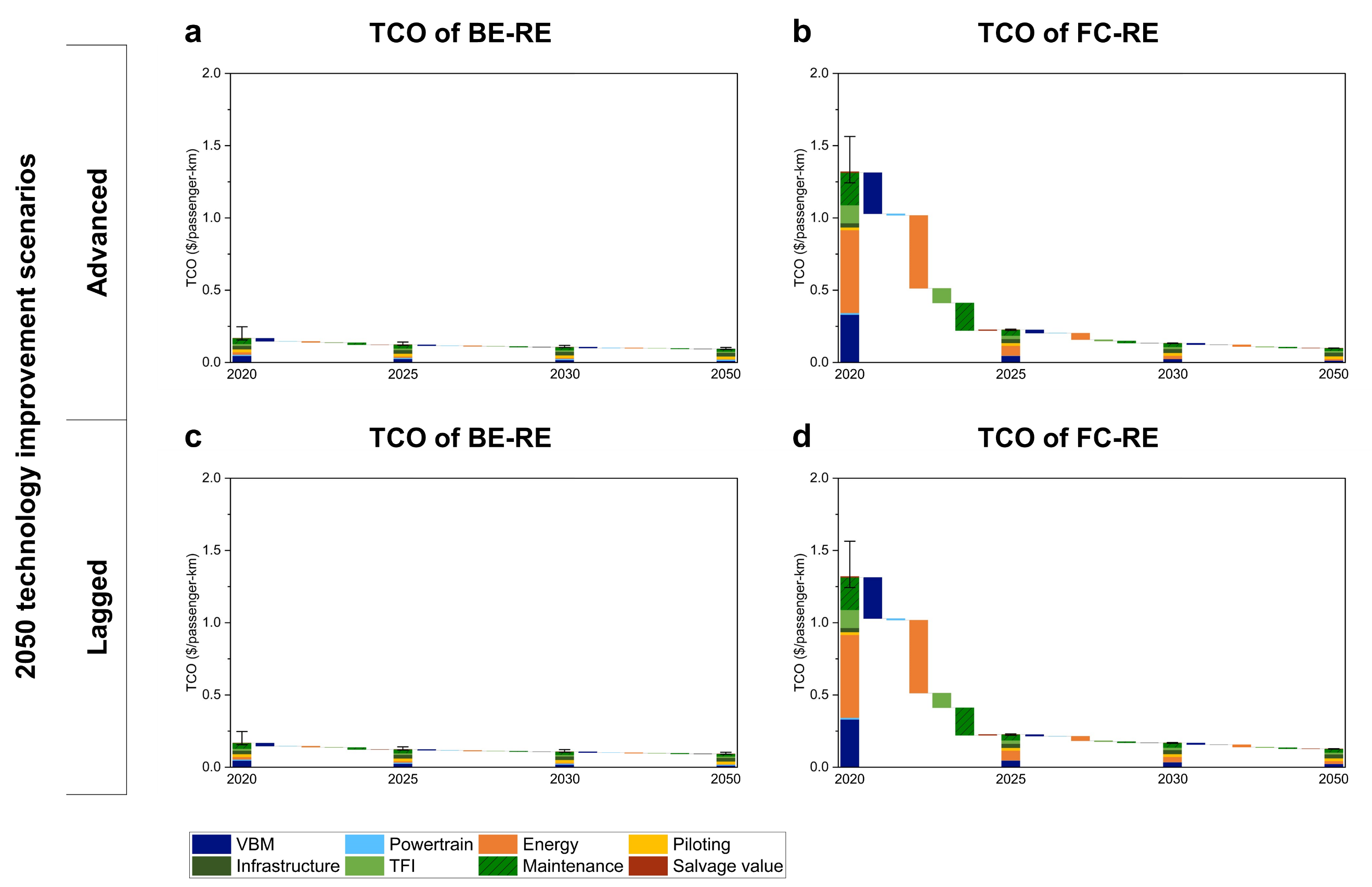


**Fig. S18.** TCO changes and the corresponding contributing factors under the alternative scenarios for airport shuttles based on RE technologies. Subfigures (a) and (c) show the changes in TCO of BE-RE with advanced and lagged battery technology development for 2050, with the changes broken down into contributions from cost components. Subfigures (b) and (d) show the changes in TCO of FC-RE with advanced and lagged fuel cell technology development for 2050, with the changes broken down into contributions from cost components. BE: Battery Electric; FC: Fuel Cell; RE: Renewable Energy-based; TCO: Total Cost of Ownership; VBM: VTOL Body Manufacturing; TFI: Taxes, Fees, and Insurance.


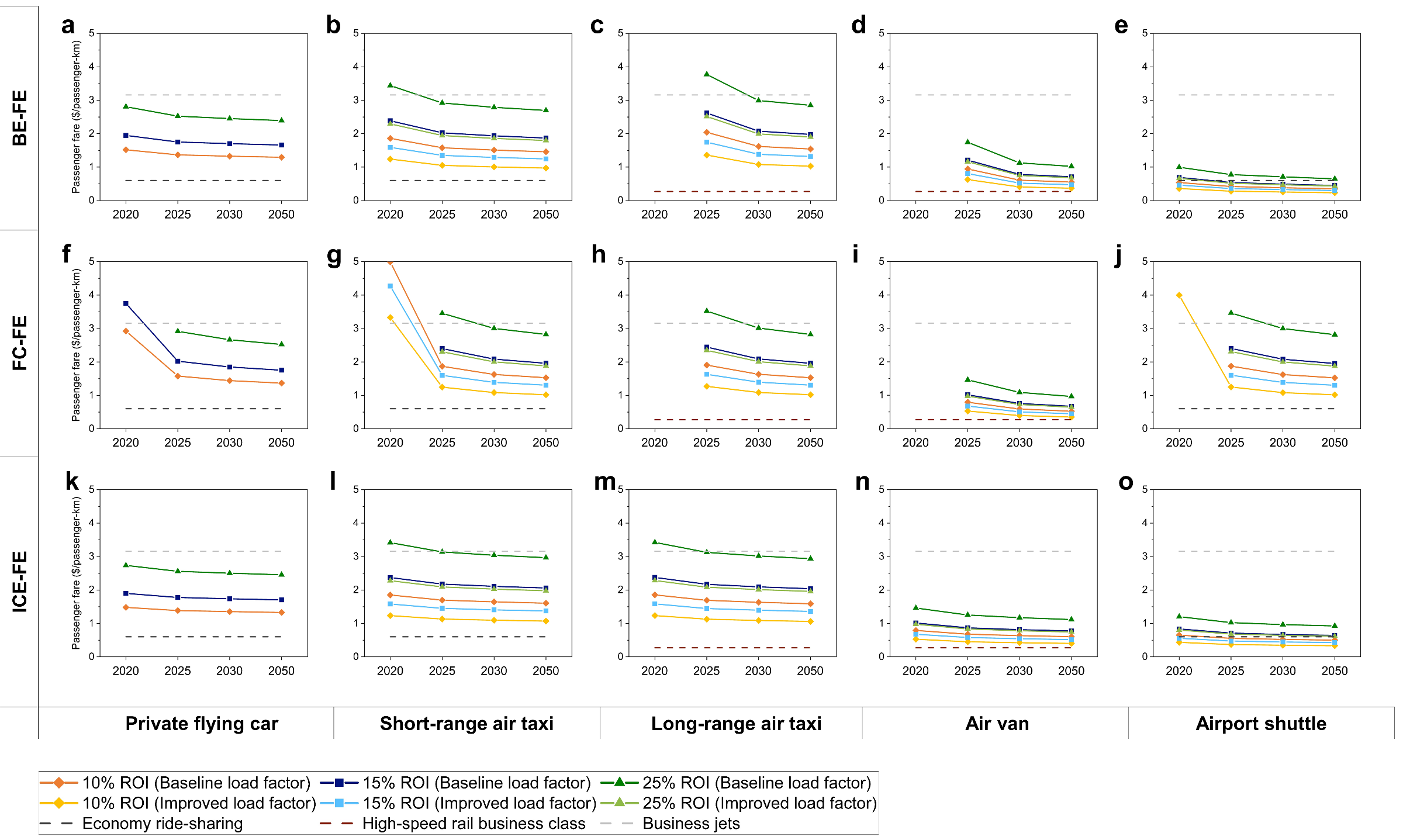


**Fig. S19.** Required passenger fares for achieving target ROIs under FE technologies. The subfigures represent results under different applications (horizontal dimension) and technologies (vertical dimension): BE-FE for private flying cars (a), short-range air taxis (b), long-range air taxis (c), air vans (d), and airport shuttles (e); FC-FE for private flying cars (f), short-range air taxis (g), long-range air taxis (h), air vans (i), and airport shuttles (j); ICE-FE for private flying cars (k), short-range air taxis (l), long-range air taxis (m), air vans (n), and airport shuttles (o). The required fares are provided for three ROIs (10%, 15%, and 25%) and two load factor cases (baseline vs. improved). For some cases, where required fares are too high to be realistic, the fares are not plotted. Average passenger fares for economy ride-sharing, high-speed rail business class, and business jets are provided as benchmarks to be compared with the results. BE: Battery Electric; FC: Fuel Cell; ICE: Internal Combustion Engine; FE: Fossil Energy-based; ROI: Return on Investment.


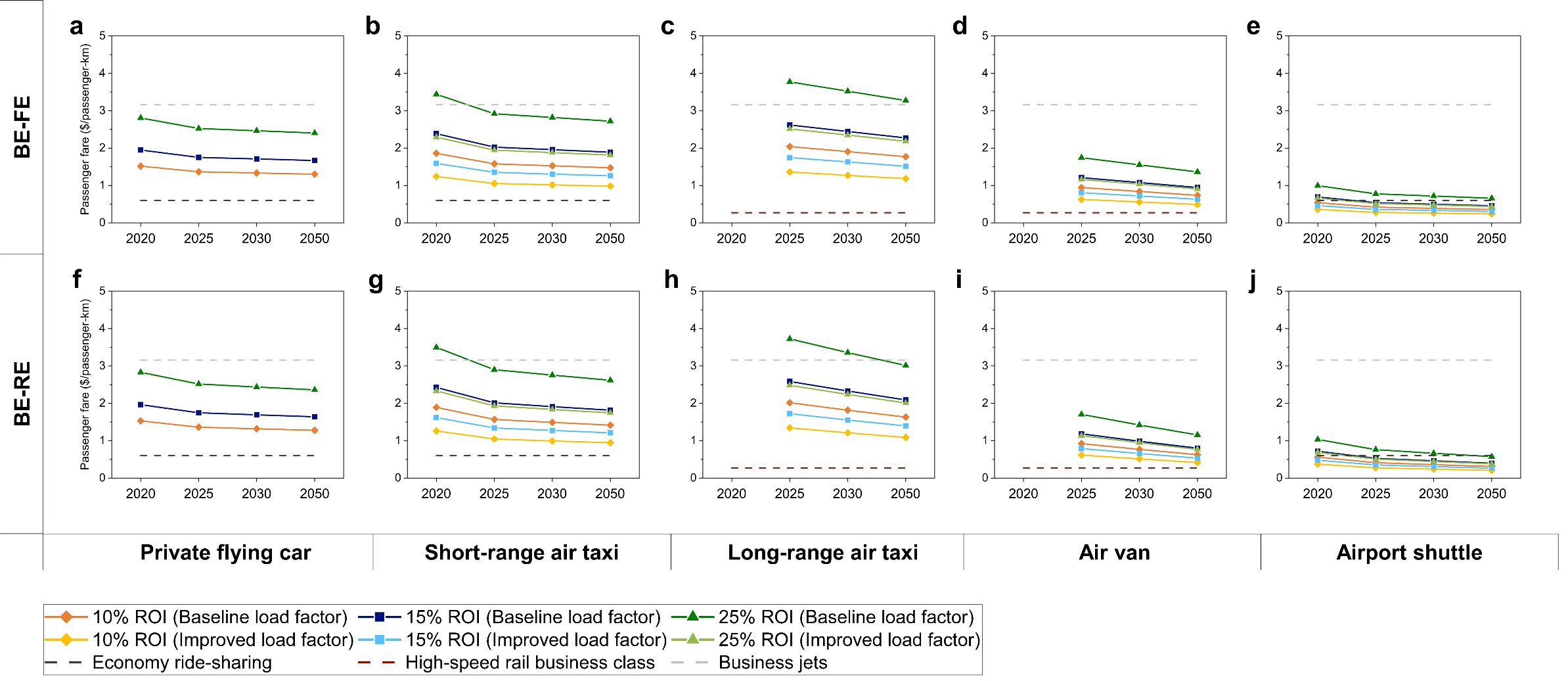


**Fig. S20.** Required passenger fares for achieving target ROIs under alternative scenario for 2050 (lagged battery technology development). The subfigures represent results for BE technologies (vertical dimension) with lagged battery technology development under different applications (horizontal dimension): BE-FE for private flying cars (a), short-range air taxis (b), long-range air taxis (c), air vans (d), and airport shuttles (e); BE-RE for private flying cars (f), short-range air taxis (g), long-range air taxis (h), air vans (i), and airport shuttles (j). The required fares are provided for three ROIs (10%, 15%, and 25%) and two load factor cases (baseline vs. improved). For some cases, where required fares are too high to be realistic, the fares are not plotted. Average passenger fares for economy ride-sharing, high-speed rail business class, and business jets are provided as benchmarks to be compared with the results. BE: Battery Electric; FE: Fossil Energy-based; RE: Renewable Energy-based; ROI: Return on Investment.


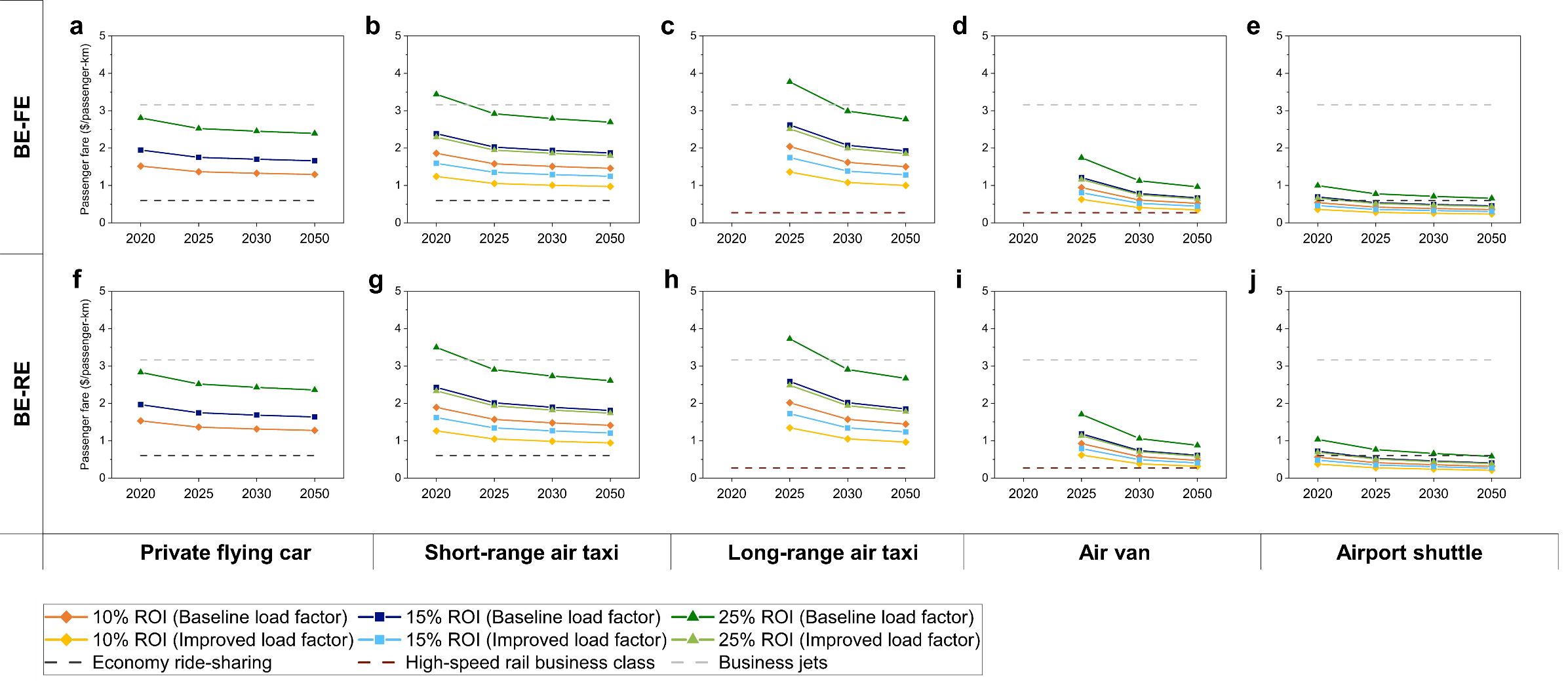


**Fig. S21.** Required passenger fares for achieving target ROIs under alternative scenario for 2050 (advanced battery technology development). The subfigures represent results for BE technologies (vertical dimension) with advanced battery technology development under different applications (horizontal dimension): BE-FE for private flying cars (a), short-range air taxis (b), long-range air taxis (c), air vans (d), and airport shuttles (e); BE-RE for private flying cars (f), short-range air taxis (g), long-range air taxis (h), air vans (i), and airport shuttles (j). The required fares are provided for three ROIs (10%, 15%, and 25%) and two load factor cases (baseline vs. improved). For some cases, where required fares are too high to be realistic, the fares are not plotted. Average passenger fares for economy ride-sharing, high-speed rail business class, and business jets are provided as benchmarks to be compared with the results. BE: Battery Electric; FE: Fossil Energy-based; RE: Renewable Energy-based; ROI: Return on Investment.


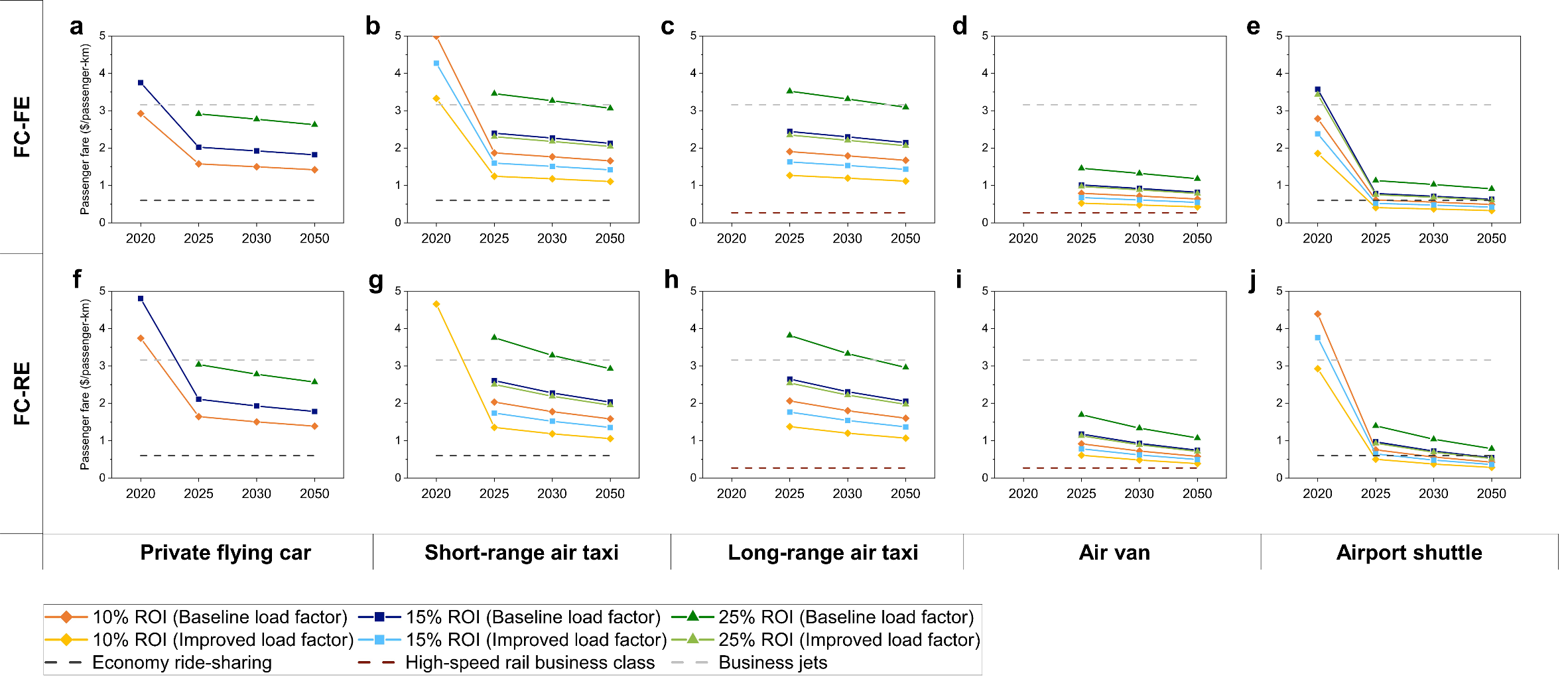


**Fig. S22.** Required passenger fares for achieving target ROIs under alternative scenario for 2050 (lagged fuel cell technology development). The subfigures represent results for FC technologies (vertical dimension) with lagged fuel cell technology development under different applications (horizontal dimension): FC-FE for private flying cars (a), short-range air taxis (b), long-range air taxis (c), air vans (d), and airport shuttles (e); FC-RE for private flying cars (f), short-range air taxis (g), long-range air taxis (h), air vans (i), and airport shuttles (j). The required fares are provided for three ROIs (10%, 15%, and 25%) and two load factor cases (baseline vs. improved). For some cases, where required fares are too high to be realistic, the fares are not plotted. Average passenger fares for economy ride-sharing, high-speed rail business class, and business jets are provided as benchmarks to be compared with the results. FC: Fuel Cell; FE: Fossil Energy-based; RE: Renewable Energy-based; ROI: Return on Investment.


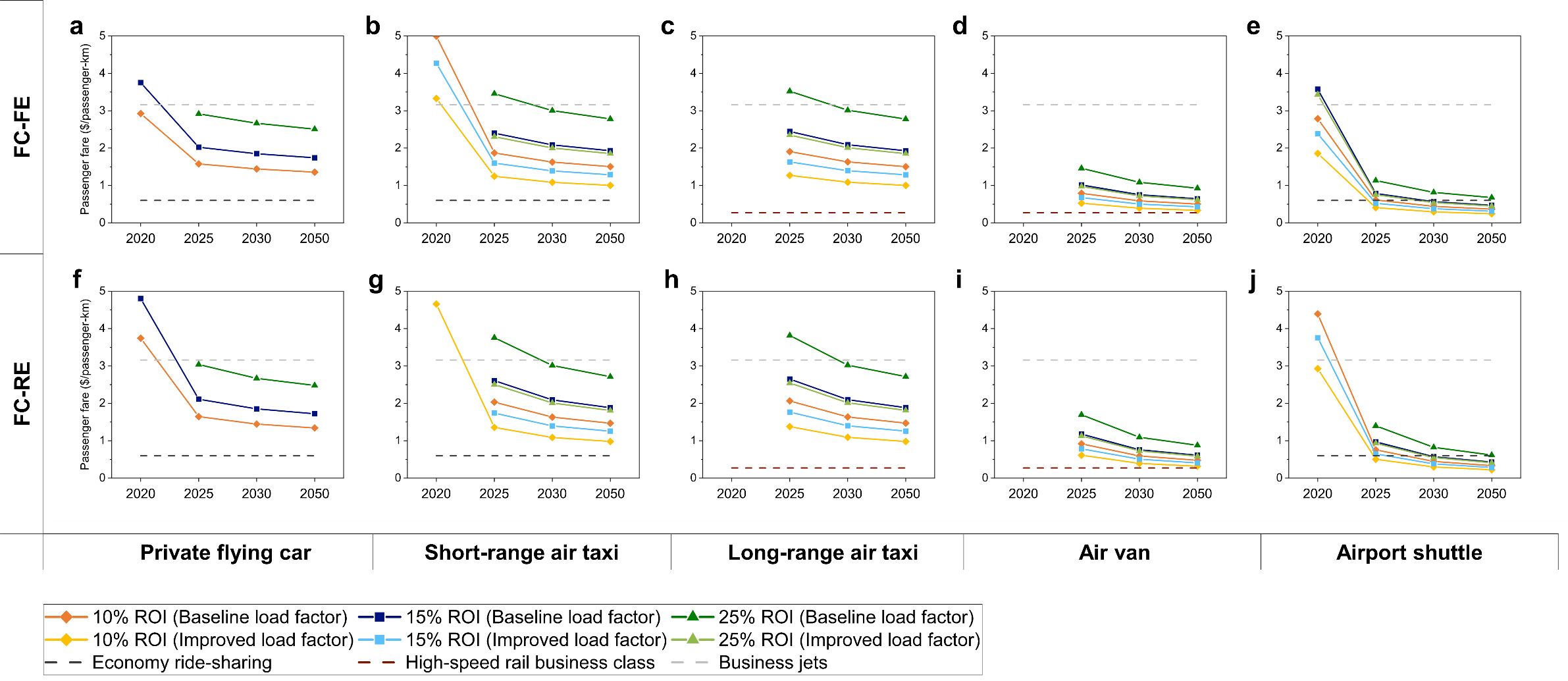


**Fig. S23.** Required passenger fares for achieving target ROIs under alternative scenario for 2050 (advanced fuel cell technology development). The subfigures represent results for FC technologies (vertical dimension) with advanced fuel cell technology development under different applications (horizontal dimension): FC-FE for private flying cars (a), short-range air taxis (b), long-range air taxis (c), air vans (d), and airport shuttles (e); FC-RE for private flying cars (f), short-range air taxis (g), long-range air taxis (h), air vans (i), and airport shuttles (j). The required fares are provided for three ROIs (10%, 15%, and 25%) and two load factor cases (baseline vs. improved). For some cases, where required fares are too high to be realistic, the fares are not plotted. Average passenger fares for economy ride-sharing, high-speed rail business class, and business jets are provided as benchmarks to be compared with the results. FC: Fuel Cell; FE: Fossil Energy-based; RE: Renewable Energy-based; ROI: Return on Investment.


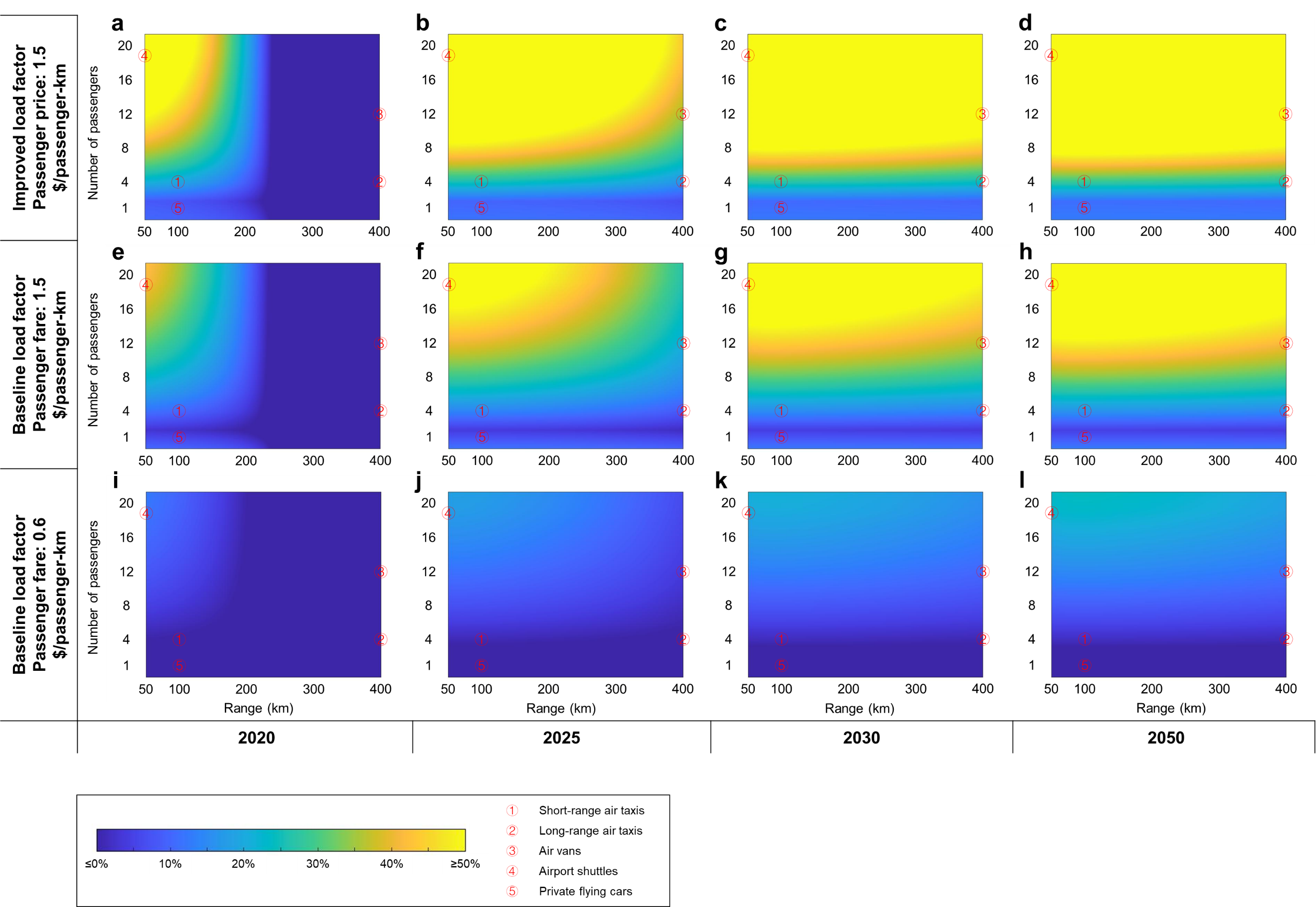


**Fig. S24.** The ROI of BE-FE VTOLs operation. The subfigures represent results under different load factors (baseline vs. improved) and passenger fares (baseline: $0.60/passenger-km vs. increased: $1.50/passenger-km) cases in 2020, 2025, 2030, and 2050: ROI under improved load factor and increased passenger fare in 2020 (a), 2025 (b), 2030 (c), and 2050 (d); ROI under baseline load factor and increased passenger fare in 2020 (e), 2025 (f), 2030 (g), and 2050 (h); ROI under baseline load factor and baseline passenger fare in 2020 (i), 2025 (j), 2030 (k), and 2050 (l). The ROI for a certain application domain is indicated by the color of the area, which can be linked to the corresponding color in the legend. In each subfigure, typical applications of VTOLs, including air taxis, air vans, airport shuttles, and private flying cars, are indicated using the circled numbers. BE: Battery Electric; FE: Fossil Energy-based; ROI: Return on Investment.


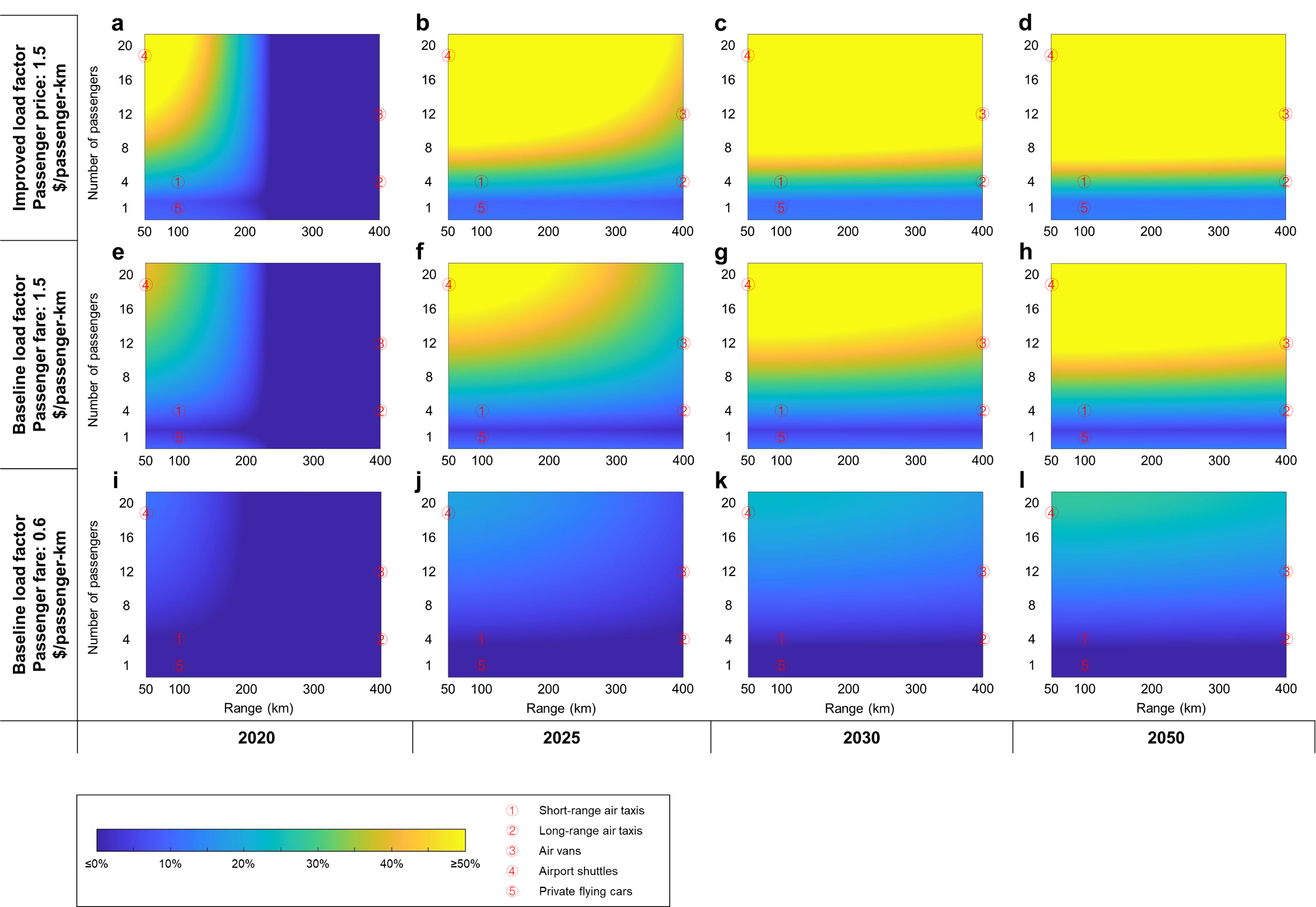


**Fig. S25.** The ROI of BE-RE VTOLs operation. The subfigures represent results under different load factors (baseline vs. improved) and passenger fares (baseline: $0.60/passenger-km vs. increased: $1.50/passenger-km) cases in 2020, 2025, 2030, and 2050: ROI under improved load factor and increased passenger fare in 2020 (a), 2025 (b), 2030 (c), and 2050 (d); ROI under baseline load factor and increased passenger fare in 2020 (e), 2025 (f), 2030 (g), and 2050 (h); ROI under baseline load factor and baseline passenger fare in 2020 (i), 2025 (j), 2030 (k), and 2050 (l). The ROI for a certain application domain is indicated by the color of the area, which can be linked to the corresponding color in the legend. In each subfigure, typical applications of VTOLs, including air taxis, air vans, airport shuttles, and private flying cars, are indicated using the circled numbers. BE: Battery Electric; RE: Renewable Energy-based; ROI: Return on Investment.


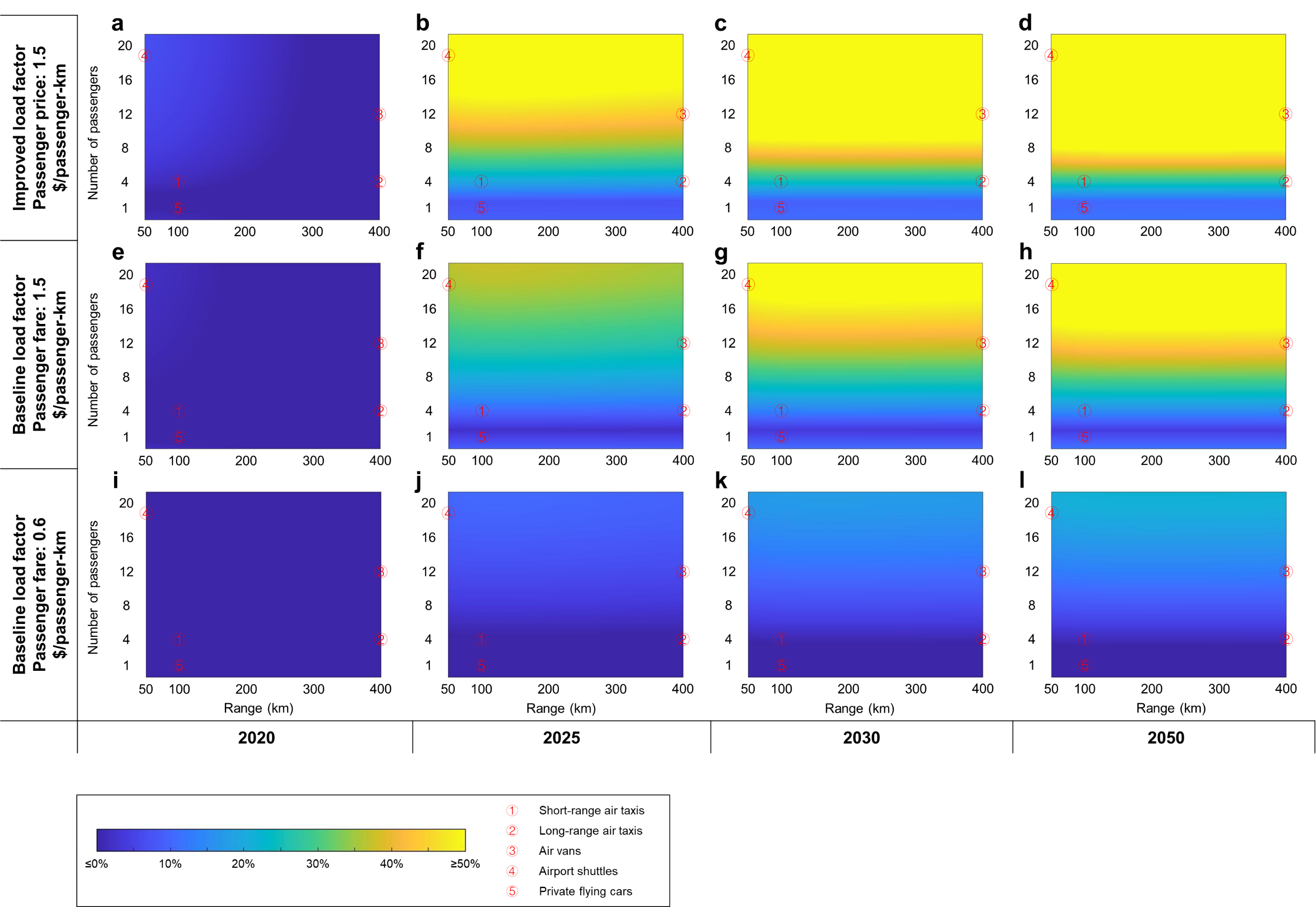


**Fig. S26.** The ROI of FC-FE VTOLs operation. The subfigures represent results under different load factors (baseline vs. improved) and passenger fares (baseline: $0.60/passenger-km vs. increased: $1.50/passenger-km) cases in 2020, 2025, 2030, and 2050: ROI under improved load factor and increased passenger fare in 2020 (a), 2025 (b), 2030 (c), and 2050 (d); ROI under baseline load factor and increased passenger fare in 2020 (e), 2025 (f), 2030 (g), and 2050 (h); ROI under baseline load factor and baseline passenger fare in 2020 (i), 2025 (j), 2030 (k), and 2050 (l). The ROI for a certain application domain is indicated by the color of the area, which can be linked to the corresponding color in the legend. In each subfigure, typical applications of VTOLs, including air taxis, air vans, airport shuttles, and private flying cars, are indicated using the circled numbers. FC: Fuel Cell; FE: Fossil Energy-based; ROI: Return on Investment.


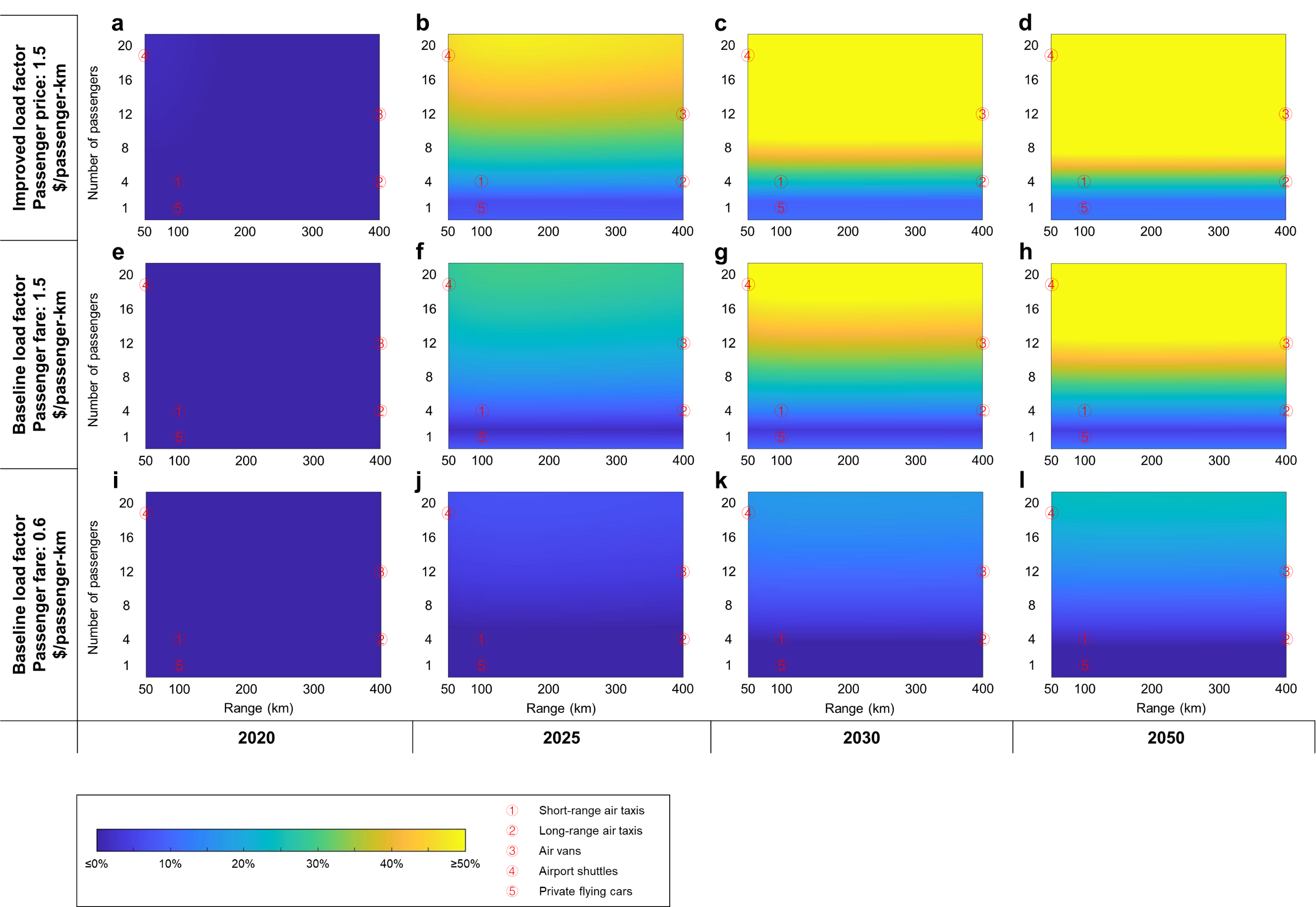


**Fig. S27.** The ROI of FC-RE VTOLs operation. The subfigures represent results under different load factors (baseline vs. improved) and passenger fares (baseline: $0.60/passenger-km vs. increased: $1.50/passenger-km) cases in 2020, 2025, 2030, and 2050: ROI under improved load factor and increased passenger fare in 2020 (a), 2025 (b), 2030 (c), and 2050 (d); ROI under baseline load factor and increased passenger fare in 2020 (e), 2025 (f), 2030 (g), and 2050 (h); ROI under baseline load factor and baseline passenger fare in 2020 (i), 2025 (j), 2030 (k), and 2050 (l). The ROI for a certain application domain is indicated by the color of the area, which can be linked to the corresponding color in the legend. In each subfigure, typical applications of VTOLs, including air taxis, air vans, airport shuttles, and private flying cars, are indicated using the circled numbers. FC: Fuel Cell; RE: Renewable Energy-based; ROI: Return on Investment.


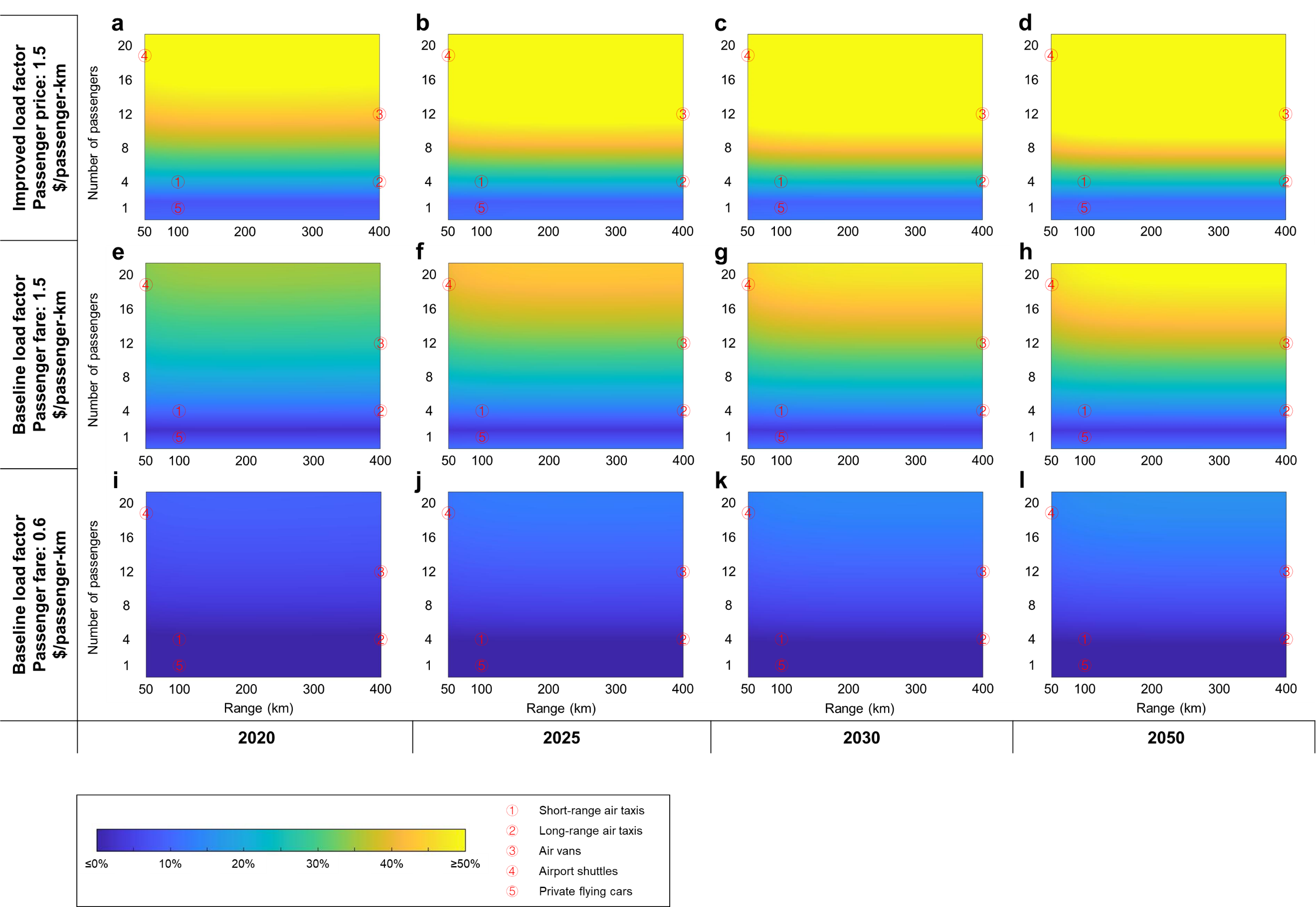


**Fig. S28.** The ROI of ICE-FE VTOLs operation. The subfigures represent results under different load factors (baseline vs. improved) and passenger fares (baseline: $0.60/passenger-km vs. increased: $1.50/passenger-km) cases in 2020, 2025, 2030, and 2050: ROI under improved load factor and increased passenger fare in 2020 (a), 2025 (b), 2030 (c), and 2050 (d); ROI under baseline load factor and increased passenger fare in 2020 (e), 2025 (f), 2030 (g), and 2050 (h); ROI under baseline load factor and baseline passenger fare in 2020 (i), 2025 (j), 2030 (k), and 2050 (l). The ROI for a certain application domain is indicated by the color of the area, which can be linked to the corresponding color in the legend. In each subfigure, typical applications of VTOLs, including air taxis, air vans, airport shuttles, and private flying cars, are indicated using the circled numbers. ICE: Internal Combustion Engine; FE: Fossil Energy-based; ROI: Return on Investment.


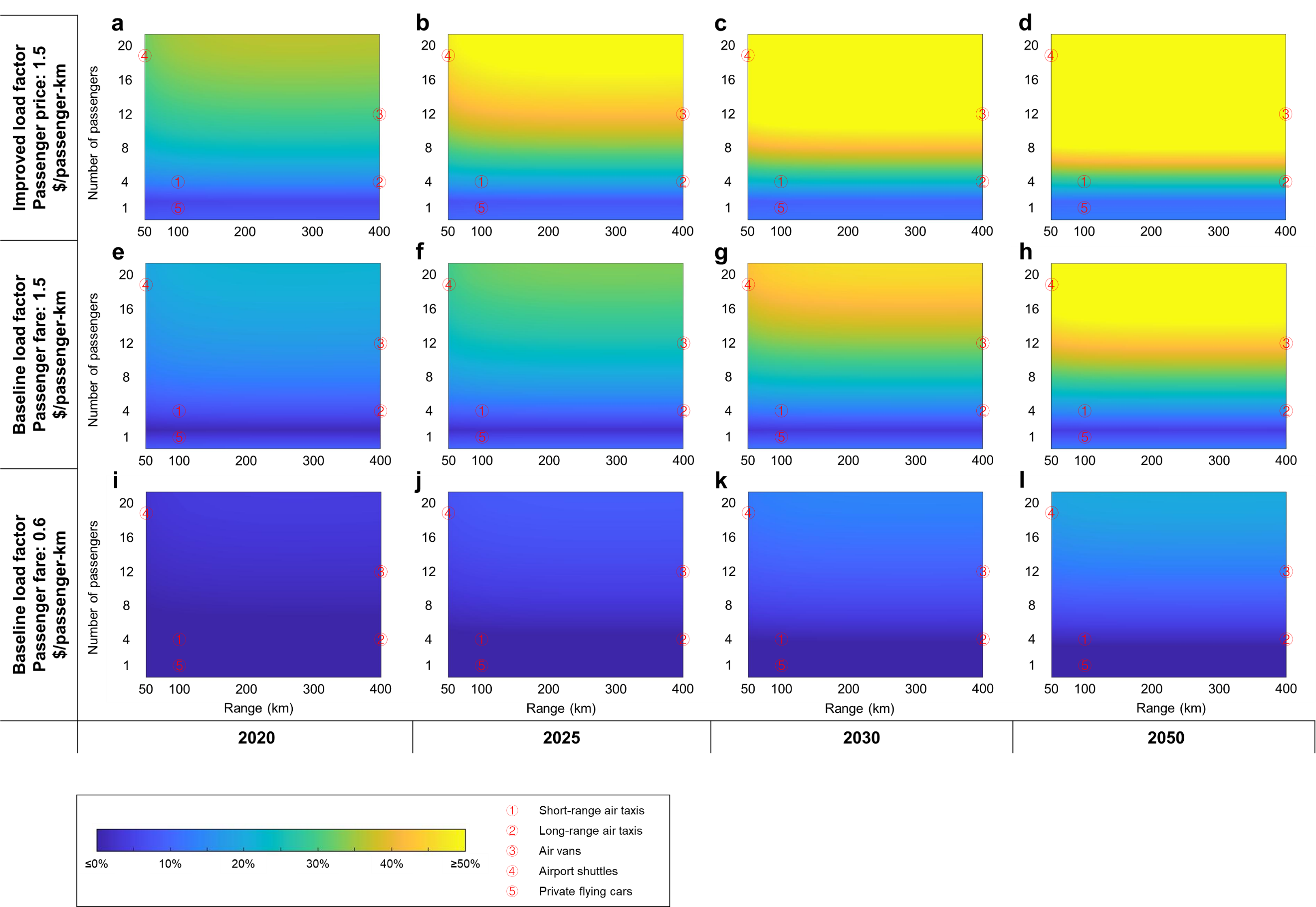


**Fig. S29.** The ROI of ICE-RE VTOLs operation. The subfigures represent results under different load factors (baseline vs. improved) and passenger fares (baseline: $0.60/passenger-km vs. increased: $1.50/passenger-km) cases in 2020, 2025, 2030, and 2050: ROI under improved load factor and increased passenger fare in 2020 (a), 2025 (b), 2030 (c), and 2050 (d); ROI under baseline load factor and increased passenger fare in 2020 (e), 2025 (f), 2030 (g), and 2050 (h); ROI under baseline load factor and baseline passenger fare in 2020 (i), 2025 (j), 2030 (k), and 2050 (l). The ROI for a certain application domain is indicated by the color of the area, which can be linked to the corresponding color in the legend. In each subfigure, typical applications of VTOLs, including air taxis, air vans, airport shuttles, and private flying cars, are indicated using the circled numbers. ICE: Internal Combustion Engine; RE: Renewable Energy-based; ROI: Return on Investment.


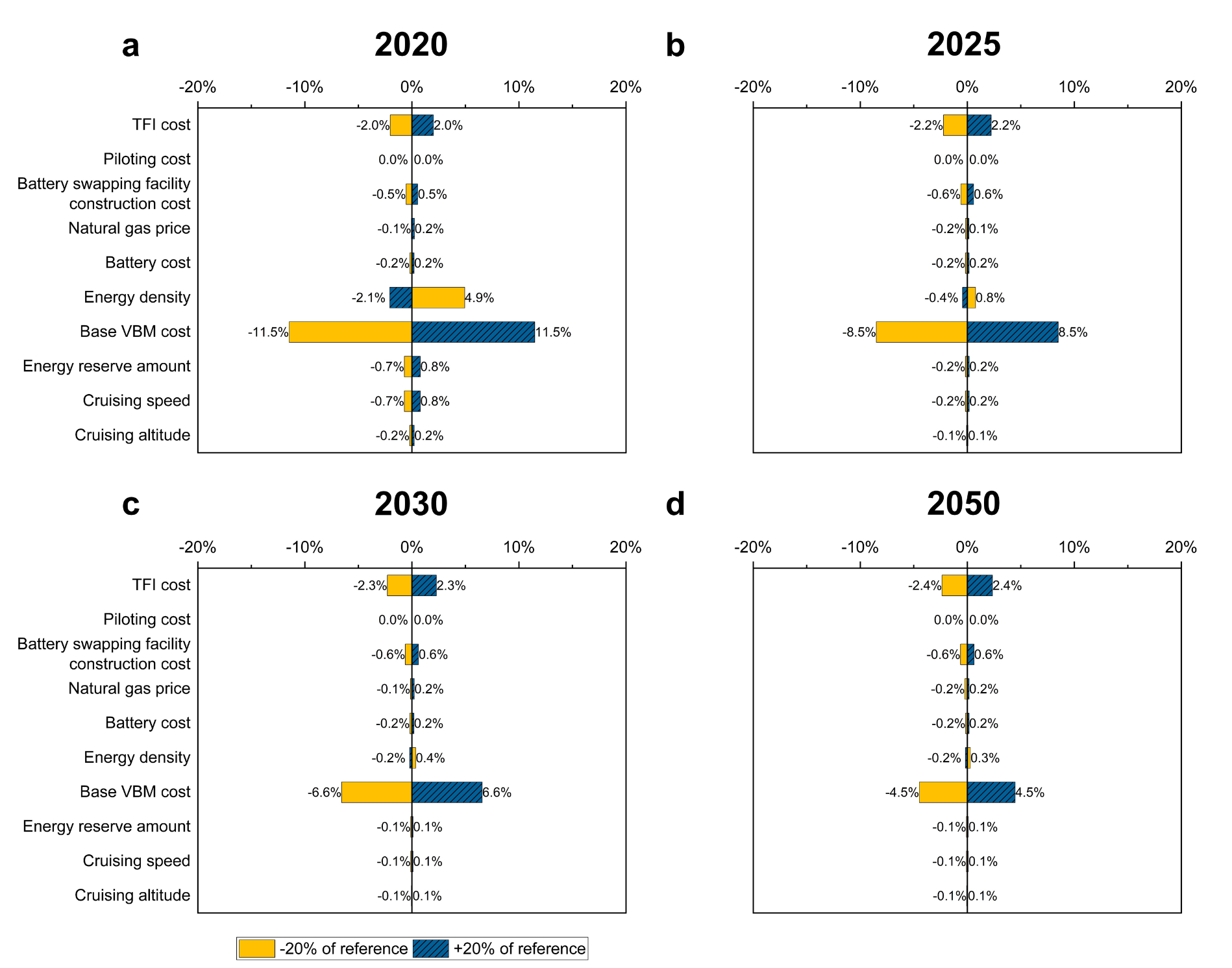


**Fig. S30.** Sensitivity analysis on the TCO of BE-FE private flying cars. The subfigures present the sensitivity of the TCO estimates to changes in VTOL technical parameters (*i.e.*, cruising speed, cruising altitude, energy reserve amount, and energy density) and cost parameters (*i.e.*, base VBM cost, battery cost, natural gas price, battery swapping facility construction cost, piloting cost, and TFI cost) in 2020 (a), 2025 (b), 2030 (c), and 2050 (d), respectively. The TCO changes under each parameter at -20% and +20% of its baseline value are shown. BE: Battery Electric; FE: Fossil Energy-based; TCO: Total Cost of Ownership; VTOL: Vertical Takeoff and Landing aircraft; VBM: VTOL Body Manufacturing; TFI: Taxes, Fees, and Insurance.


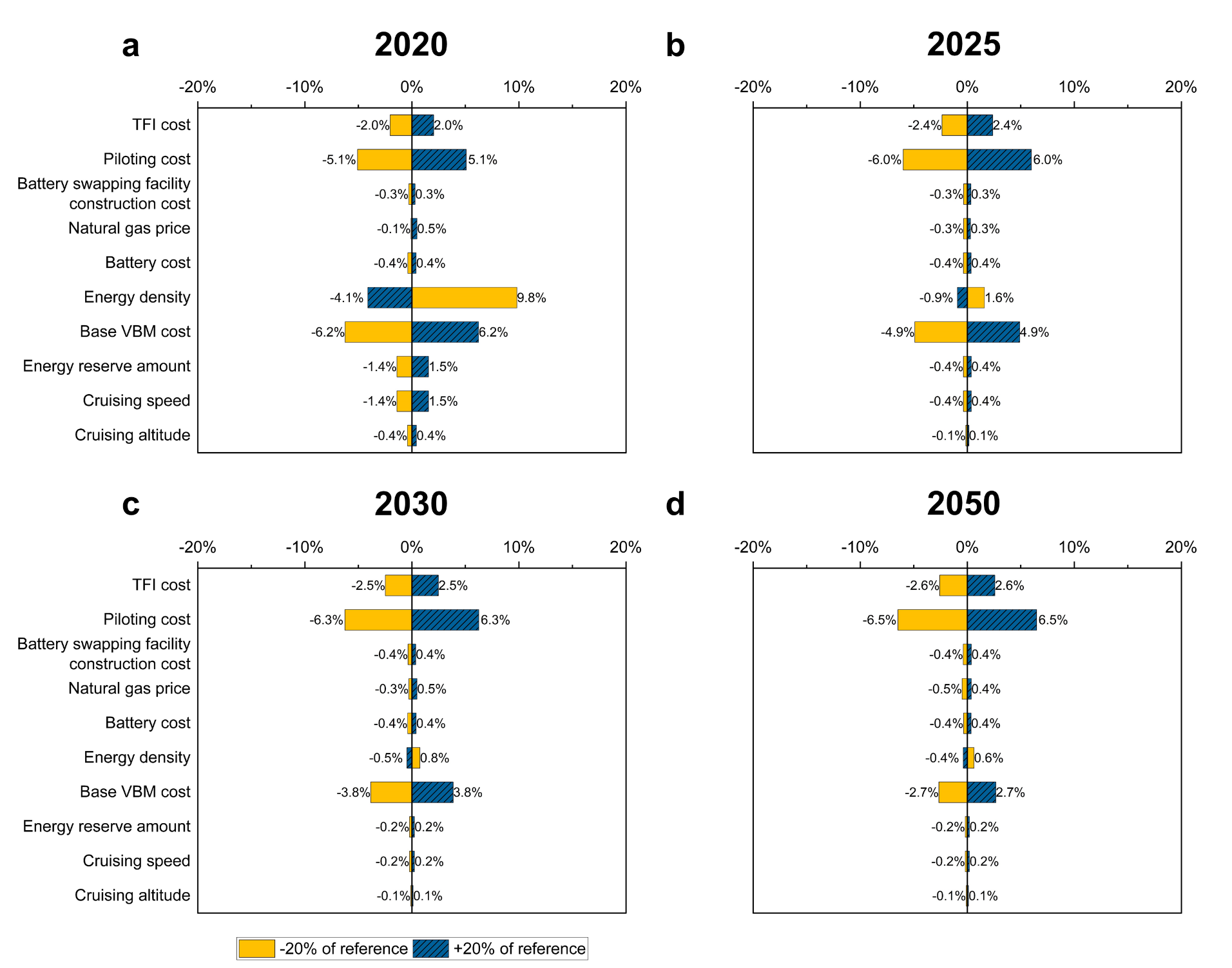


**Fig. S31.** Sensitivity analysis on the TCO of BE-FE short-range air taxis. The subfigures present the sensitivity of the TCO estimates to changes in VTOL technical parameters (*i.e.*, cruising speed, cruising altitude, energy reserve amount, and energy density) and cost parameters (*i.e.*, base VBM cost, battery cost, natural gas price, battery swapping facility construction cost, piloting cost, and TFI cost) in 2020 (a), 2025 (b), 2030 (c), and 2050 (d), respectively. The TCO changes under each parameter at -20% and +20% of its baseline value are shown. BE: Battery Electric; FE: Fossil Energy-based; TCO: Total Cost of Ownership; VTOL: Vertical Takeoff and Landing aircraft; VBM: VTOL Body Manufacturing; TFI: Taxes, Fees, and Insurance.


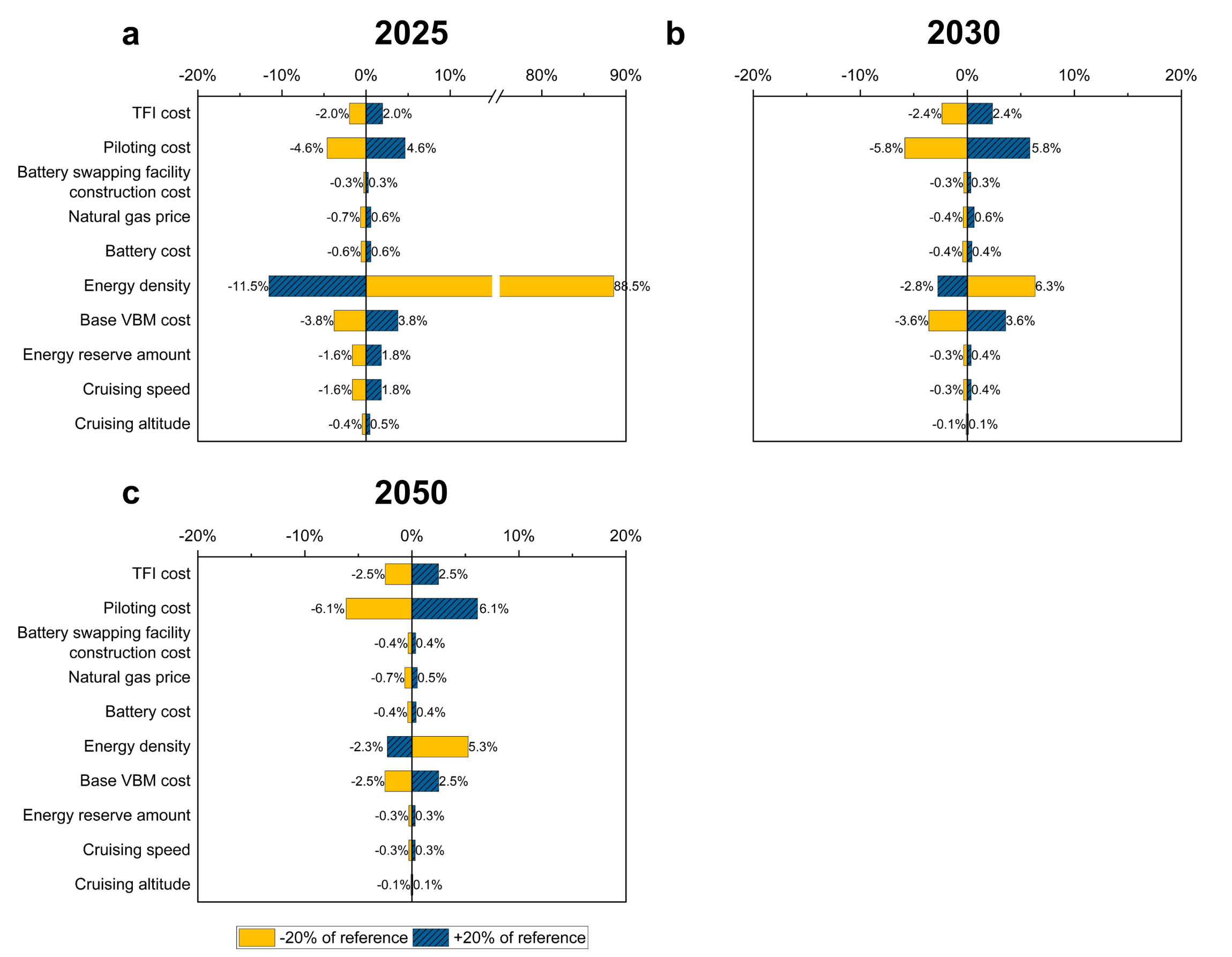


**Fig. S32.** Sensitivity analysis on the TCO of BE-FE long-range air taxis. The subfigures present the sensitivity of the TCO estimates to changes in VTOL technical parameters (*i.e.*, cruising speed, cruising altitude, energy reserve amount, and energy density) and cost parameters (*i.e.*, base VBM cost, battery cost, natural gas price, battery swapping facility construction cost, piloting cost, and TFI cost) in 2025 (a), 2030 (b), and 2050 (c), respectively. The TCO changes under each parameter at -20% and +20% of its baseline value are shown. The absence of analysis for certain years indicates technological infeasibility. BE: Battery Electric; FE: Fossil Energy-based; TCO: Total Cost of Ownership; VTOL: Vertical Takeoff and Landing aircraft; VBM: VTOL Body Manufacturing; TFI: Taxes, Fees, and Insurance.


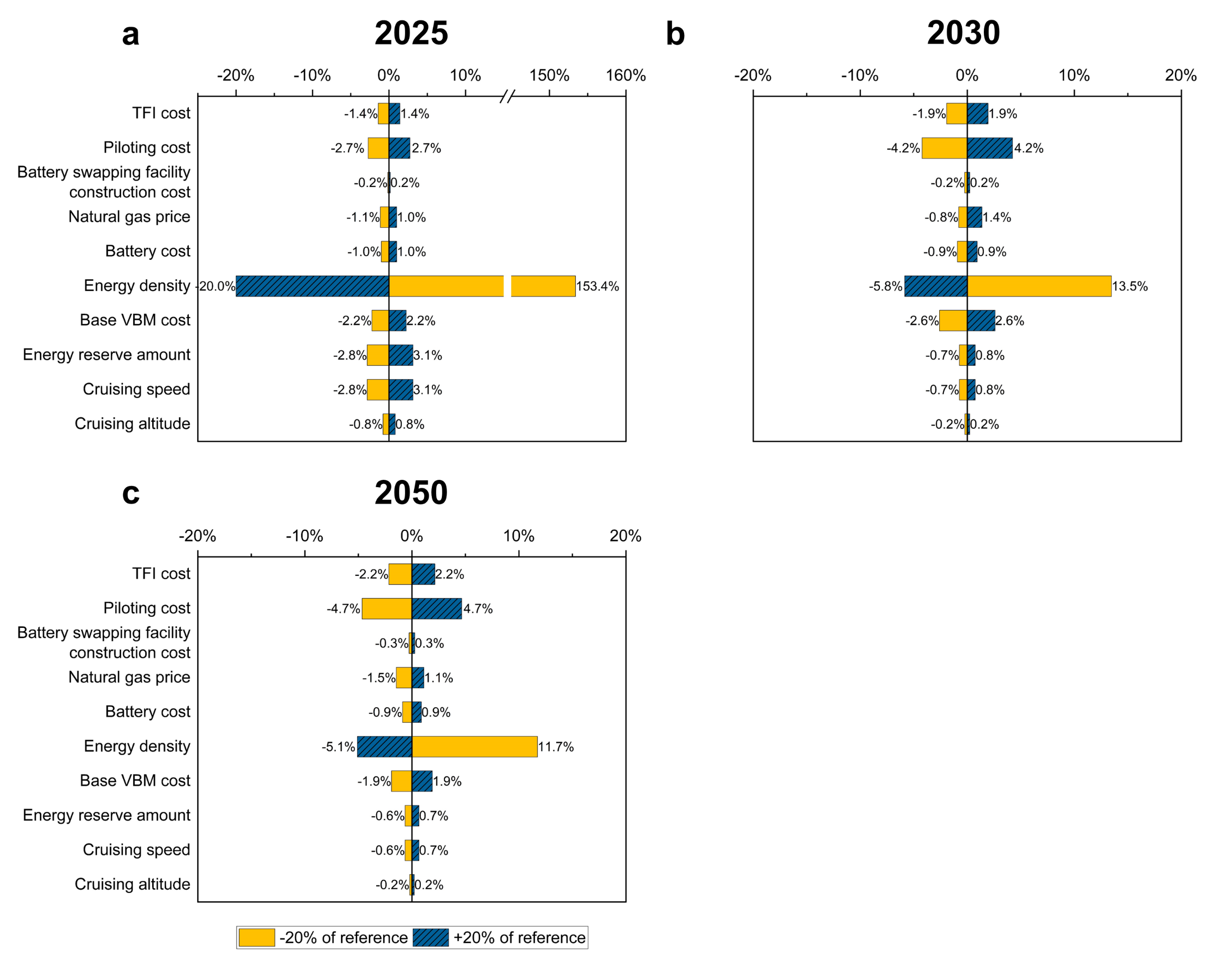


**Fig. S33.** Sensitivity analysis on the TCO of BE-FE air vans. The subfigures present the sensitivity of the TCO estimates to changes in VTOL technical parameters (*i.e.*, cruising speed, cruising altitude, energy reserve amount, and energy density) and cost parameters (*i.e.*, base VBM cost, battery cost, natural gas price, battery swapping facility construction cost, piloting cost, and TFI cost) in 2025 (a), 2030 (b), and 2050 (c), respectively. The TCO changes under each parameter at -20% and +20% of its baseline value are shown. The absence of analysis for certain years indicates technological infeasibility. BE: Battery Electric; FE: Fossil Energy-based; TCO: Total Cost of Ownership; VTOL: Vertical Takeoff and Landing aircraft; VBM: VTOL Body Manufacturing; TFI: Taxes, Fees, and Insurance.


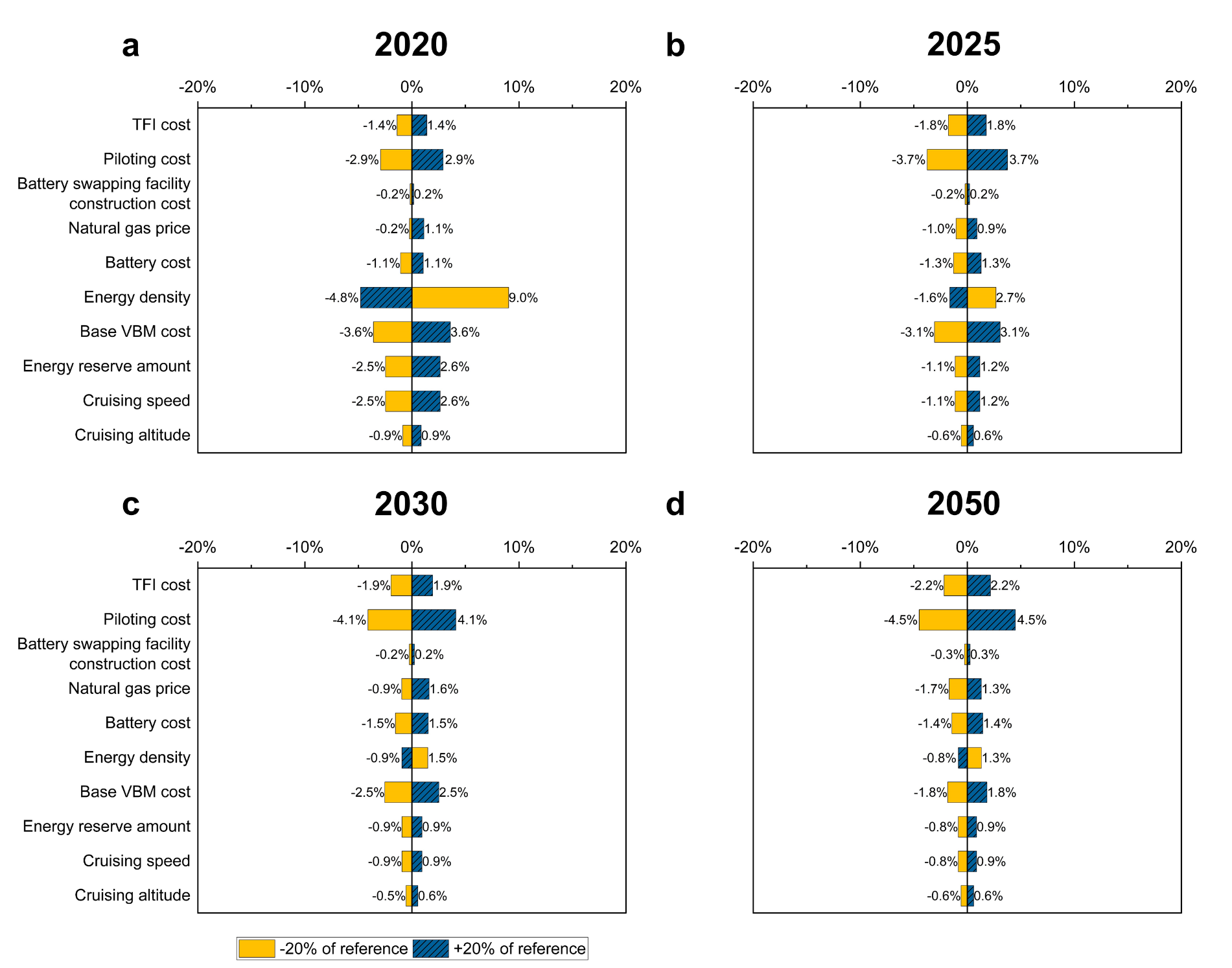


**Fig. S34.** Sensitivity analysis on the TCO of BE-FE airport shuttles. The subfigures present the sensitivity of the TCO estimates to changes in VTOL technical parameters (*i.e.*, cruising speed, cruising altitude, energy reserve amount, and energy density) and cost parameters (*i.e.*, base VBM cost, battery cost, natural gas price, battery swapping facility construction cost, piloting cost, and TFI cost) in 2020 (a), 2025 (b), 2030 (c), and 2050 (d), respectively. The TCO changes under each parameter at -20% and +20% of its baseline value are shown. BE: Battery Electric; FE: Fossil Energy-based; TCO: Total Cost of Ownership; VTOL: Vertical Takeoff and Landing aircraft; VBM: VTOL Body Manufacturing; TFI: Taxes, Fees, and Insurance.


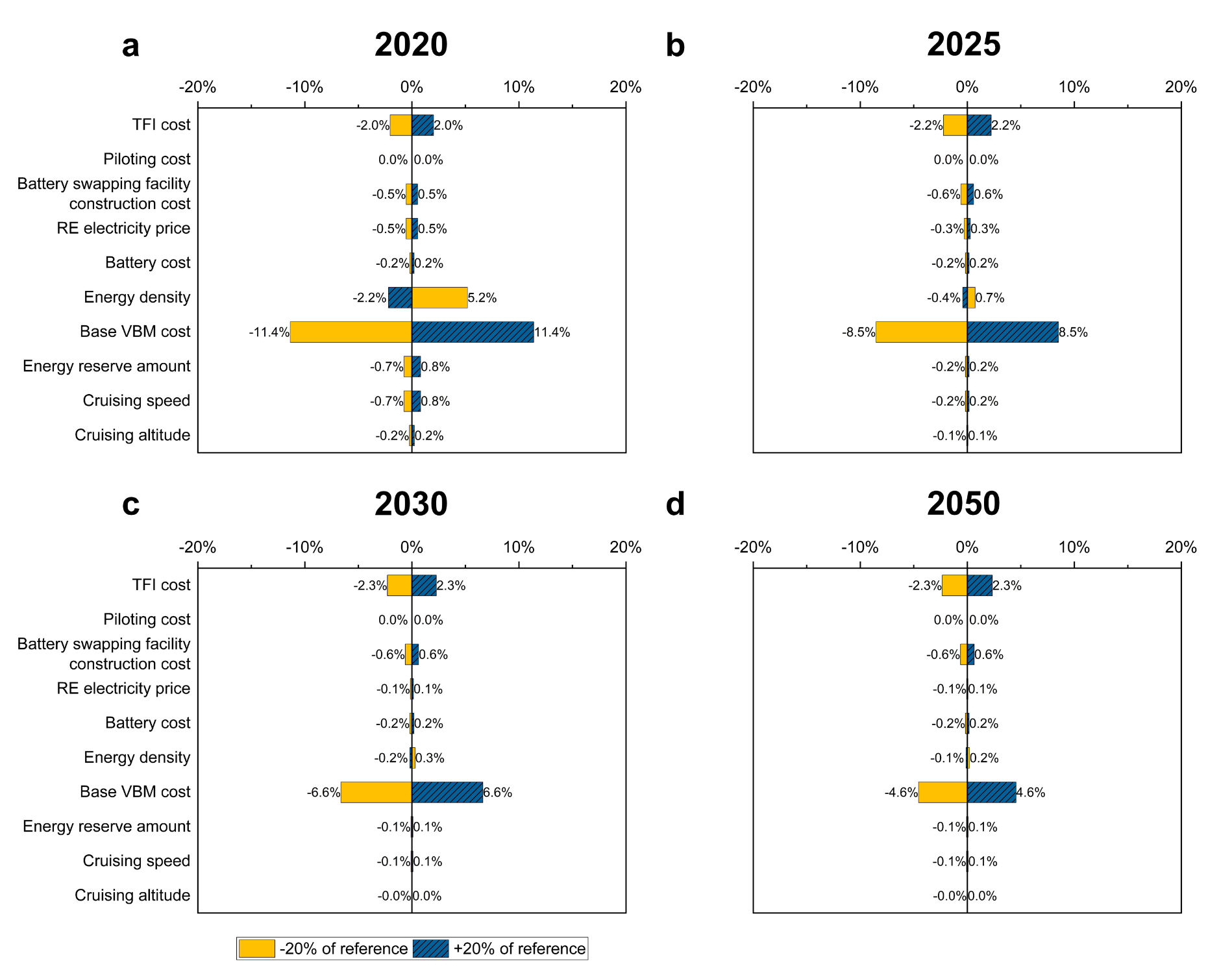


**Fig. S35.** Sensitivity analysis on the TCO of BE-RE private flying cars. The subfigures present the sensitivity of the TCO estimates to changes in VTOL technical parameters (*i.e.*, cruising speed, cruising altitude, energy reserve amount, and energy density) and cost parameters (*i.e.*, base VBM cost, battery cost, RE electricity price, battery swapping facility construction cost, piloting cost, and TFI cost) in 2020 (a), 2025 (b), 2030 (c), and 2050 (d), respectively. The TCO changes under each parameter at -20% and +20% of its baseline value are shown. BE: Battery Electric; RE: Renewable Energy-based; TCO: Total Cost of Ownership; VTOL: Vertical Takeoff and Landing aircraft; VBM: VTOL Body Manufacturing; TFI: Taxes, Fees, and Insurance.


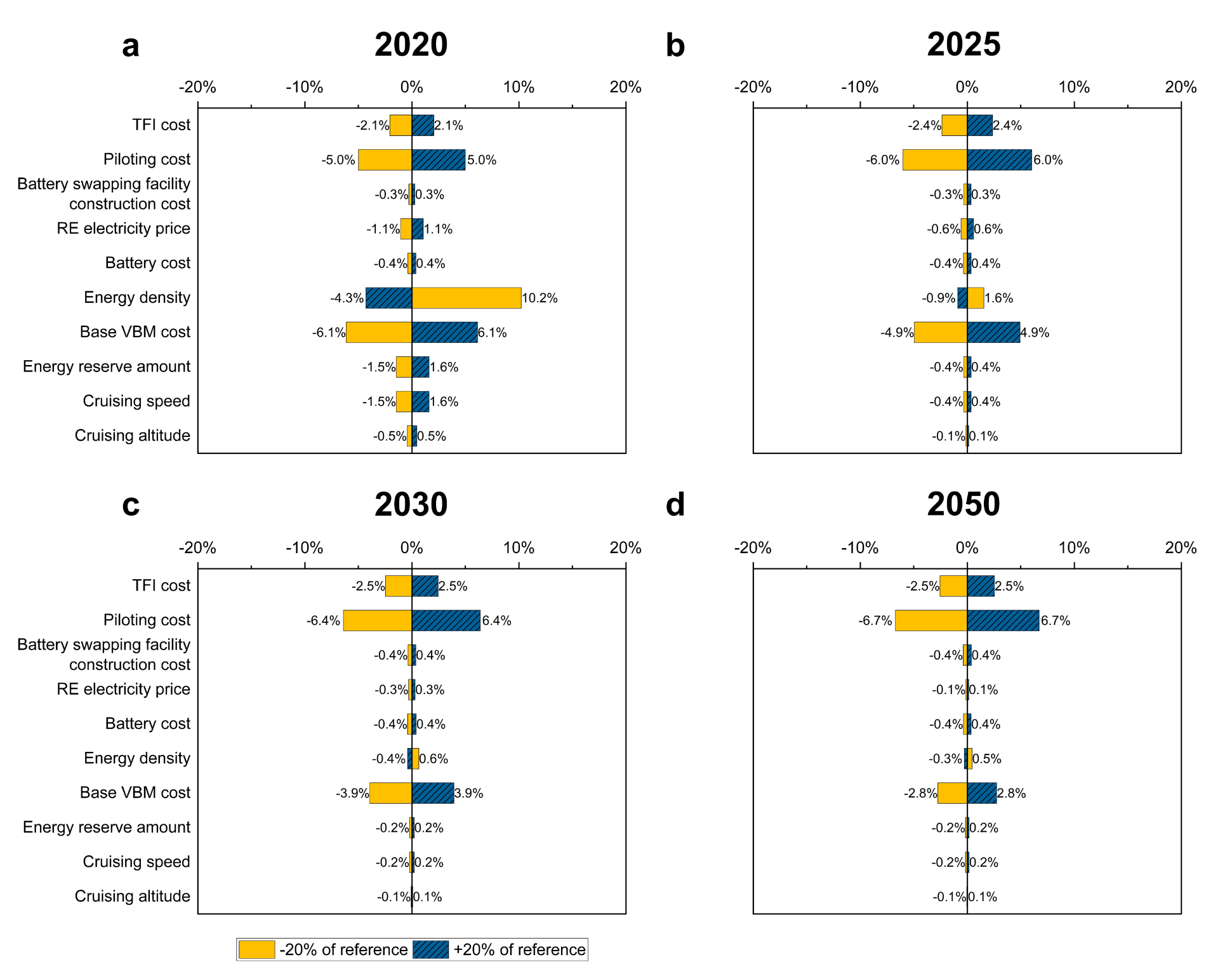


**Fig. S36.** Sensitivity analysis on the TCO of BE-RE short-range air taxis. The subfigures present the sensitivity of the TCO estimates to changes in VTOL technical parameters (*i.e.*, cruising speed, cruising altitude, energy reserve amount, and energy density) and cost parameters (*i.e.*, base VBM cost, battery cost, RE electricity price, battery swapping facility construction cost, piloting cost, and TFI cost) in 2020 (a), 2025 (b), 2030 (c), and 2050 (d), respectively. The TCO changes under each parameter at -20% and +20% of its baseline value are shown. BE: Battery Electric; RE: Renewable Energy-based; TCO: Total Cost of Ownership; VTOL: Vertical Takeoff and Landing aircraft; VBM: VTOL Body Manufacturing; TFI: Taxes, Fees, and Insurance.


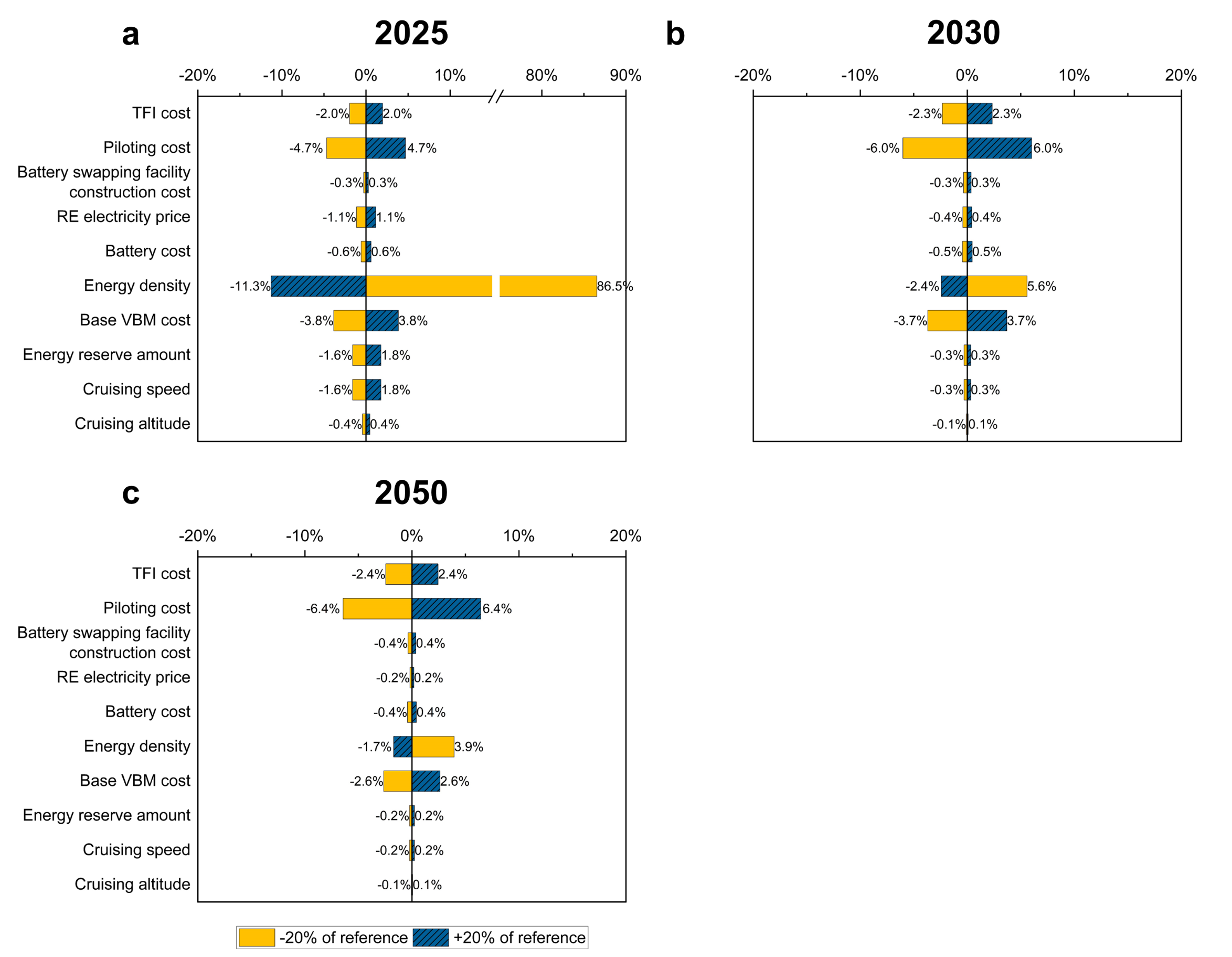


**Fig. S37.** Sensitivity analysis on the TCO of BE-RE long-range air taxis. The subfigures present the sensitivity of the TCO estimates to changes in VTOL technical parameters (*i.e.*, cruising speed, cruising altitude, energy reserve amount, and energy density) and cost parameters (*i.e.*, base VBM cost, battery cost, RE electricity price, battery swapping facility construction cost, piloting cost, and TFI cost) in 2025 (a), 2030 (b), and 2050 (c), respectively. The TCO changes under each parameter at -20% and +20% of its baseline value are shown. The absence of analysis for certain years indicates technological infeasibility. BE: Battery Electric; RE: Renewable Energy-based; TCO: Total Cost of Ownership; VTOL: Vertical Takeoff and Landing aircraft; VBM: VTOL Body Manufacturing; TFI: Taxes, Fees, and Insurance.


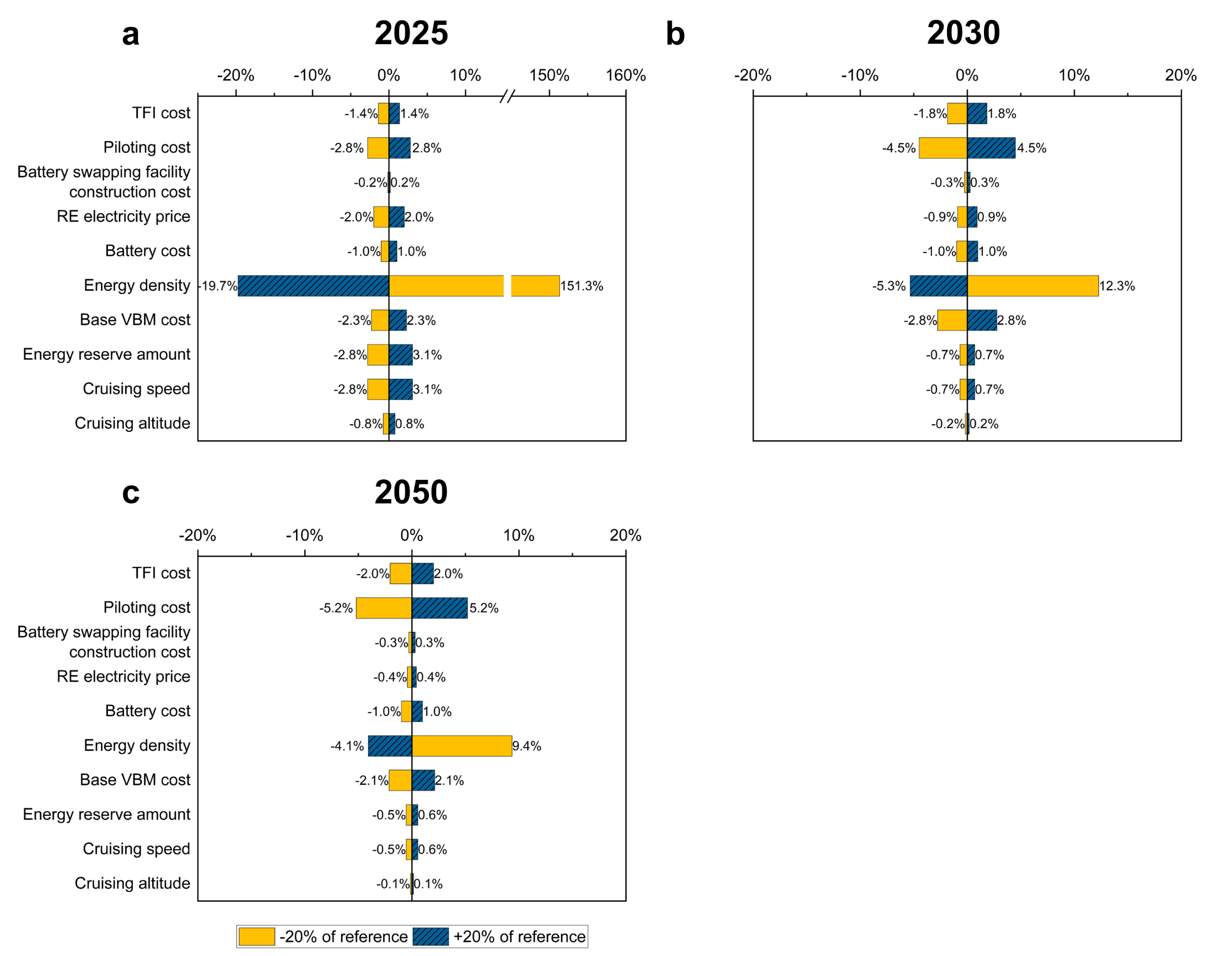


**Fig. S38.** Sensitivity analysis on the TCO of BE-RE air vans. The subfigures present the sensitivity of the TCO estimates to changes in VTOL technical parameters (*i.e.*, cruising speed, cruising altitude, energy reserve amount, and energy density) and cost parameters (*i.e.*, base VBM cost, battery cost, RE electricity price, battery swapping facility construction cost, piloting cost, and TFI cost) in 2025 (a), 2030 (b), and 2050 (c), respectively. The TCO changes under each parameter at -20% and +20% of its baseline value are shown. The absence of analysis for certain years indicates technological infeasibility. BE: Battery Electric; RE: Renewable Energy-based; TCO: Total Cost of Ownership; VTOL: Vertical Takeoff and Landing aircraft; VBM: VTOL Body Manufacturing; TFI: Taxes, Fees, and Insurance.


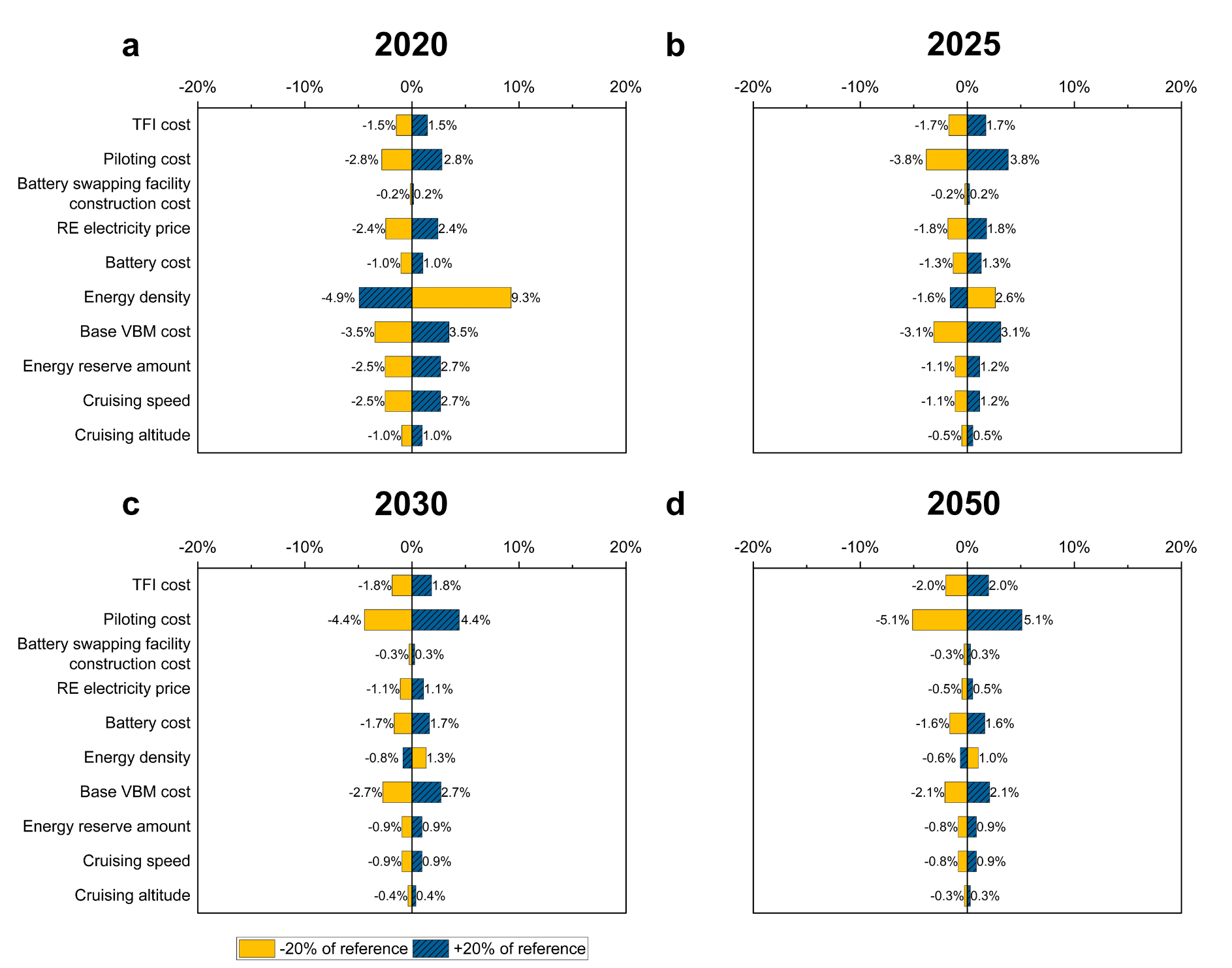


**Fig. S39.** Sensitivity analysis on the TCO of BE-RE airport shuttles. The subfigures present the sensitivity of the TCO estimates to changes in VTOL technical parameters (*i.e.*, cruising speed, cruising altitude, energy reserve amount, and energy density) and cost parameters (*i.e.*, base VBM cost, battery cost, RE electricity price, battery swapping facility construction cost, piloting cost, and TFI cost) in 2020 (a), 2025 (b), 2030 (c), and 2050 (d), respectively. The TCO changes under each parameter at -20% and +20% of its baseline value are shown. BE: Battery Electric; RE: Renewable Energy-based; TCO: Total Cost of Ownership; VTOL: Vertical Takeoff and Landing aircraft; VBM: VTOL Body Manufacturing; TFI: Taxes, Fees, and Insurance.


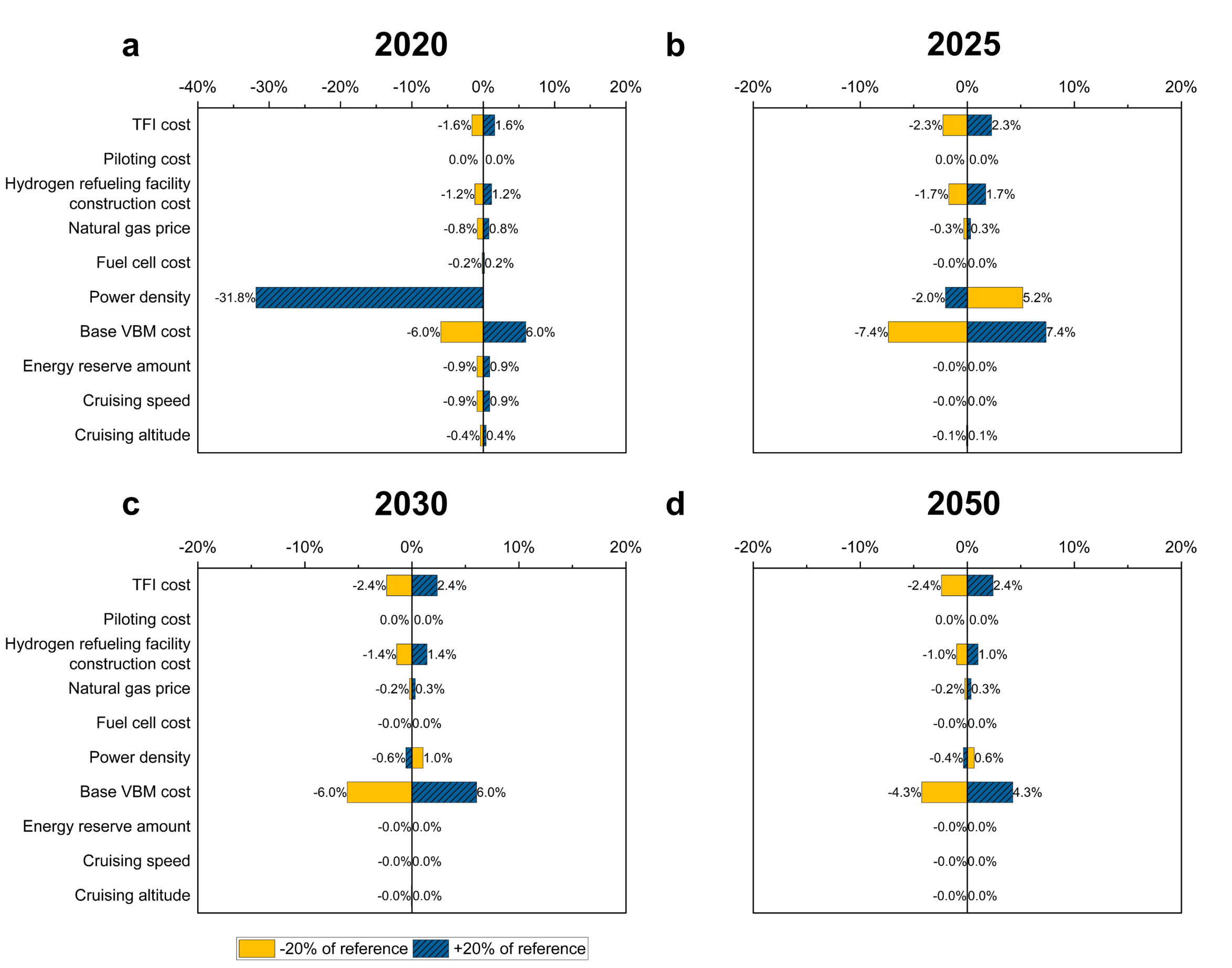


**Fig. S40.** Sensitivity analysis on the TCO of FC-FE private flying cars. The subfigures present the sensitivity of the TCO estimates to changes in VTOL technical parameters (*i.e.*, cruising speed, cruising altitude, energy reserve amount, and power density) and cost parameters (*i.e.*, base VBM cost, fuel cell cost, natural gas price, hydrogen refueling facility construction cost, piloting cost, and TFI cost) in 2020 (a), 2025 (b), 2030 (c), and 2050 (d), respectively. The TCO changes under each parameter at -20% and +20% of its baseline value are shown. FC: Fuel Cell; FE: Fossil Energy-based; TCO: Total Cost of Ownership; VTOL: Vertical Takeoff and Landing aircraft; VBM: VTOL Body Manufacturing; TFI: Taxes, Fees, and Insurance.


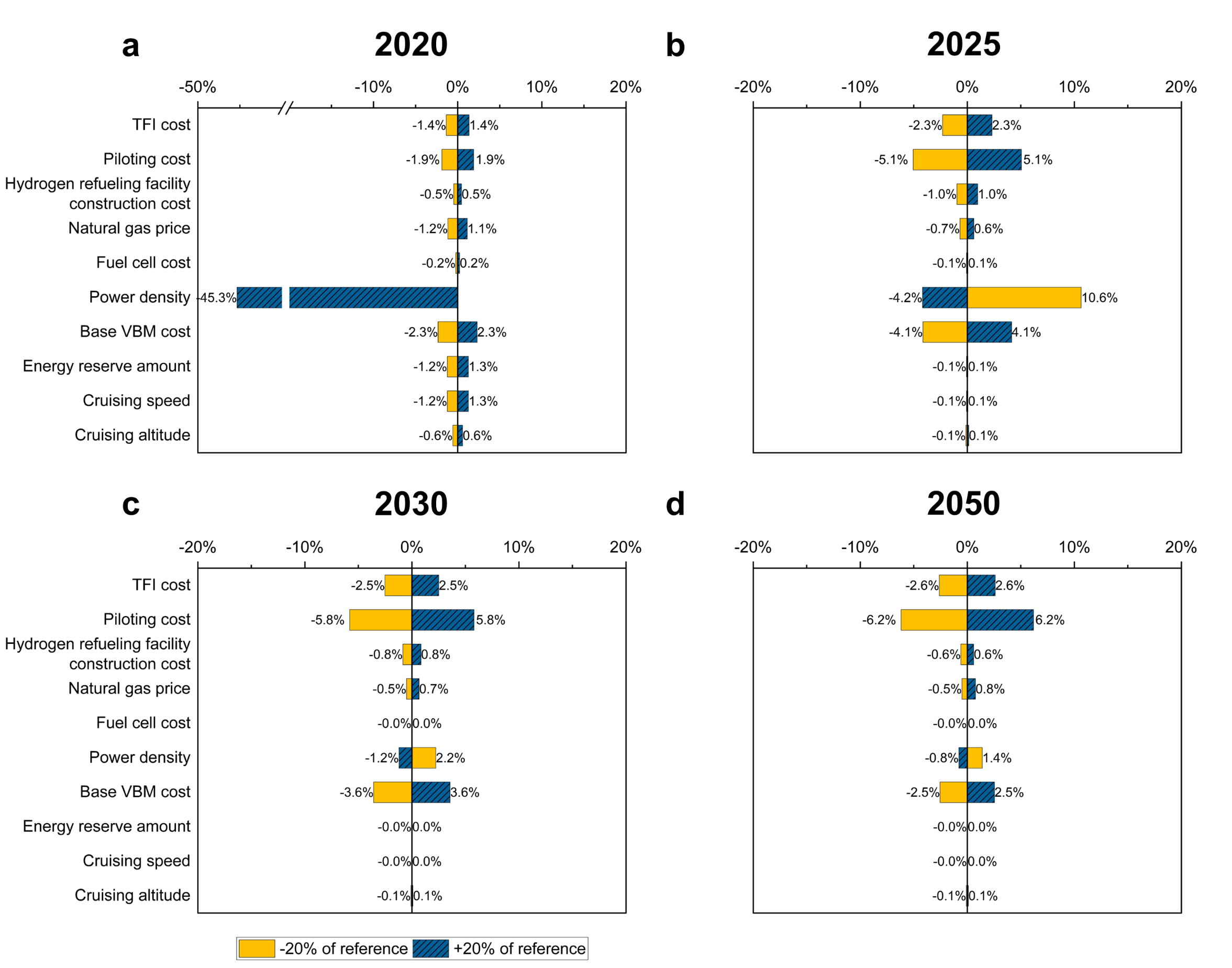


**Fig. S41.** Sensitivity analysis on the TCO of FC-FE short-range air taxis. The subfigures present the sensitivity of the TCO estimates to changes in VTOL technical parameters (*i.e.*, cruising speed, cruising altitude, energy reserve amount, and power density) and cost parameters (*i.e.*, base VBM cost, fuel cell cost, natural gas price, hydrogen refueling facility construction cost, piloting cost, and TFI cost) in 2020 (a), 2025 (b), 2030 (c), and 2050 (d), respectively. The TCO changes under each parameter at -20% and +20% of its baseline value are shown. FC: Fuel Cell; FE: Fossil Energy-based; TCO: Total Cost of Ownership; VTOL: Vertical Takeoff and Landing aircraft; VBM: VTOL Body Manufacturing; TFI: Taxes, Fees, and Insurance.


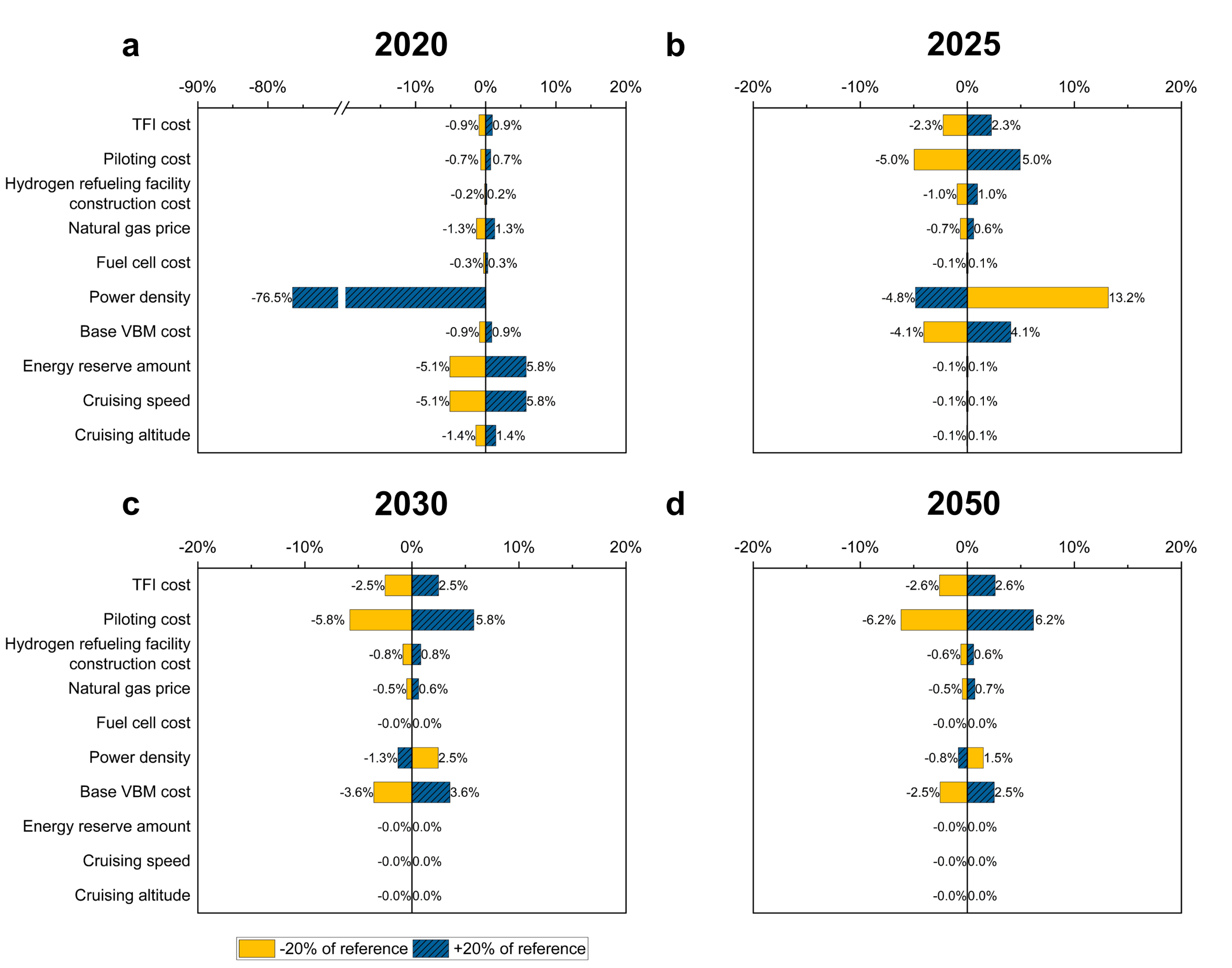


**Fig. S42.** Sensitivity analysis on the TCO of FC-FE long-range air taxis. The subfigures present the sensitivity of the TCO estimates to changes in VTOL technical parameters (*i.e.*, cruising speed, cruising altitude, energy reserve amount, and power density) and cost parameters (*i.e.*, base VBM cost, fuel cell cost, natural gas price, hydrogen refueling facility construction cost, piloting cost, and TFI cost) in 2020 (a), 2025 (b), 2030 (c), and 2050 (d), respectively. The TCO changes under each parameter at -20% and +20% of its baseline value are shown. FC: Fuel Cell; FE: Fossil Energy-based; TCO: Total Cost of Ownership; VTOL: Vertical Takeoff and Landing aircraft; VBM: VTOL Body Manufacturing; TFI: Taxes, Fees, and Insurance.


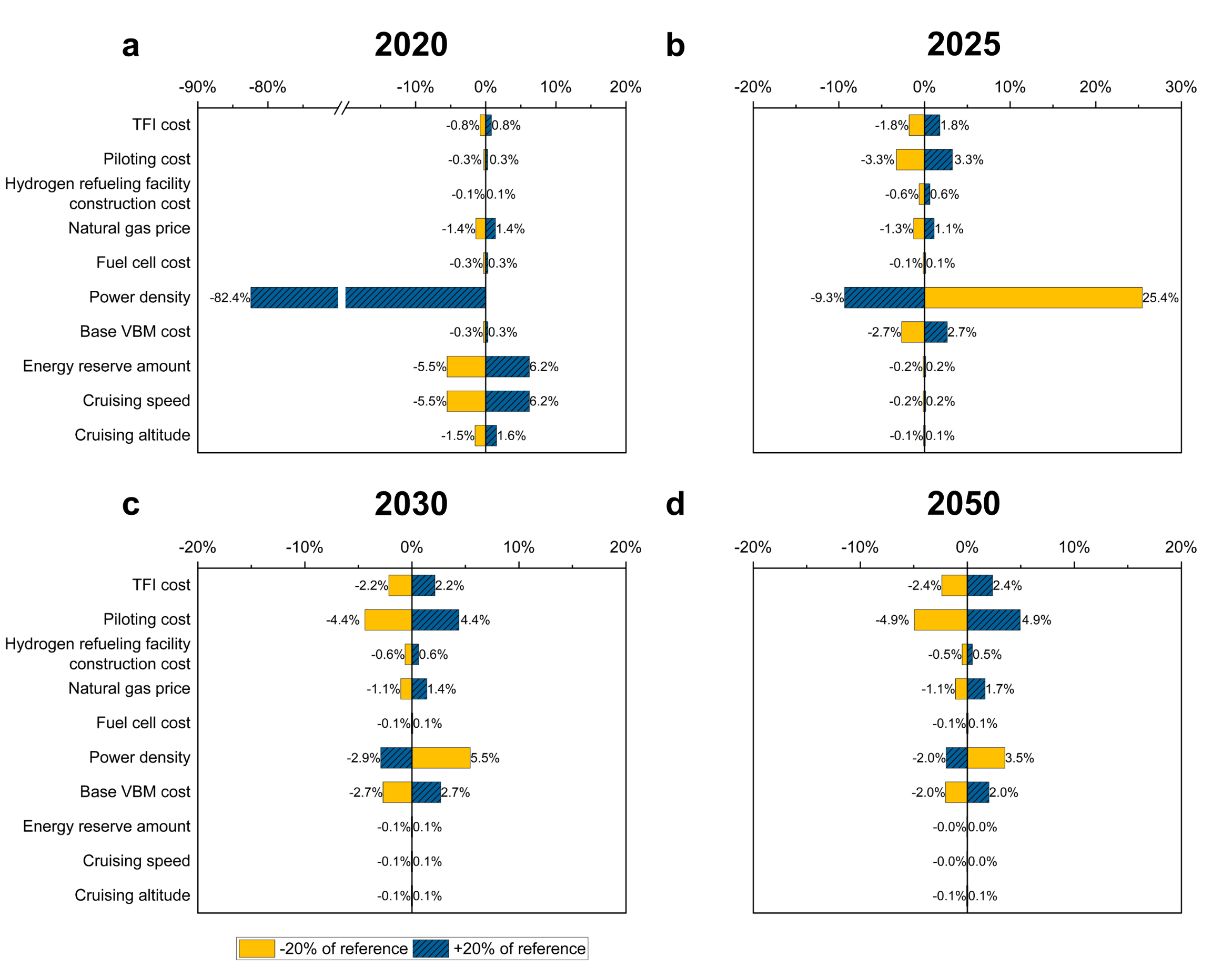


**Fig. S43.** Sensitivity analysis on the TCO of FC-FE air vans. The subfigures present the sensitivity of the TCO estimates to changes in VTOL technical parameters (*i.e.*, cruising speed, cruising altitude, energy reserve amount, and power density) and cost parameters (*i.e.*, base VBM cost, fuel cell cost, natural gas price, hydrogen refueling facility construction cost, piloting cost, and TFI cost) in 2020 (a), 2025 (b), 2030 (c), and 2050 (d), respectively. The TCO changes under each parameter at -20% and +20% of its baseline value are shown. FC: Fuel Cell; FE: Fossil Energy-based; TCO: Total Cost of Ownership; VTOL: Vertical Takeoff and Landing aircraft; VBM: VTOL Body Manufacturing; TFI: Taxes, Fees, and Insurance.


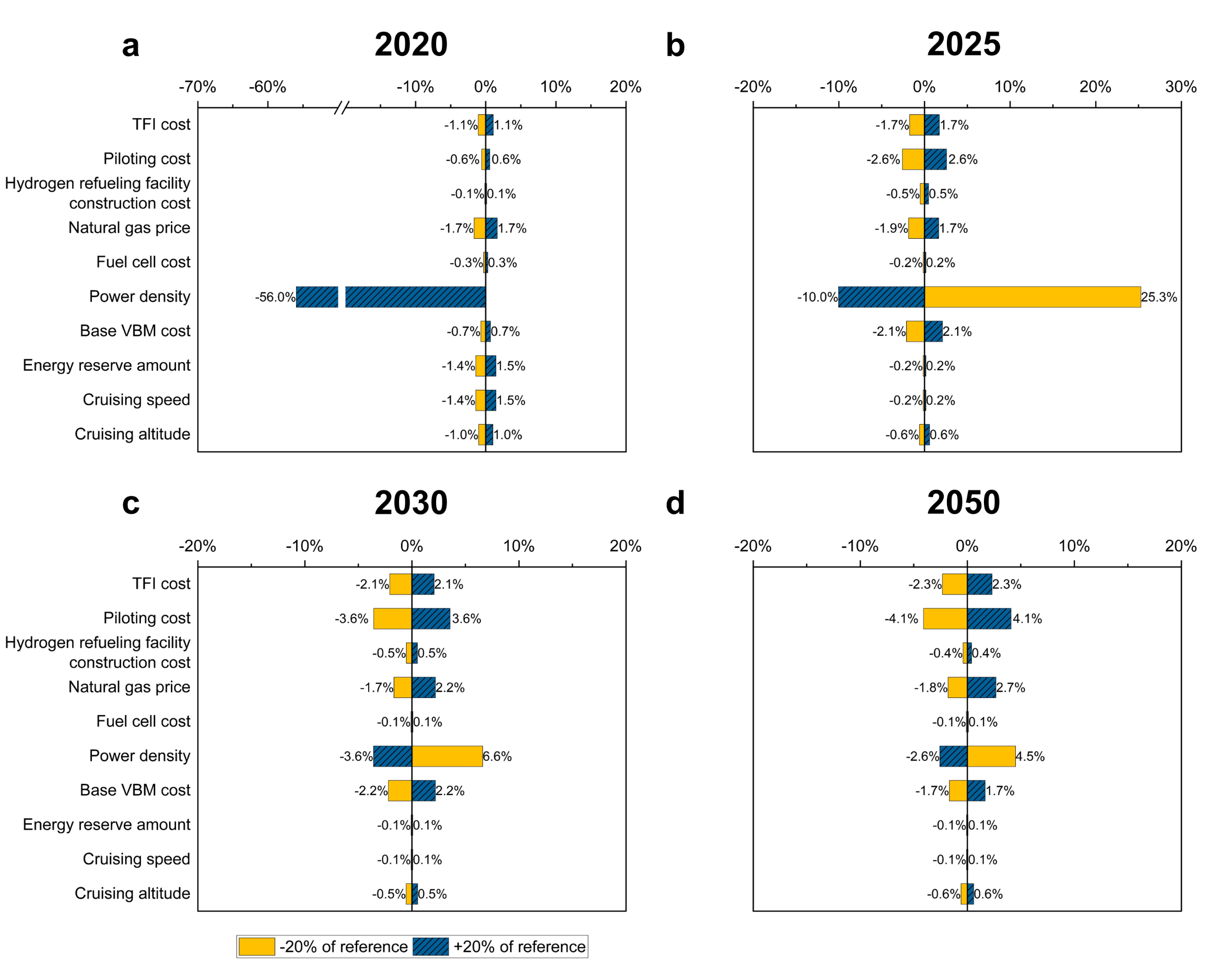


**Fig. S44.** Sensitivity analysis on the TCO of FC-FE airport shuttles. The subfigures present the sensitivity of the TCO estimates to changes in VTOL technical parameters (*i.e.*, cruising speed, cruising altitude, energy reserve amount, and power density) and cost parameters (*i.e.*, base VBM cost, fuel cell cost, natural gas price, hydrogen refueling facility construction cost, piloting cost, and TFI cost) in 2020 (a), 2025 (b), 2030 (c), and 2050 (d), respectively. The TCO changes under each parameter at -20% and +20% of its baseline value are shown. FC: Fuel Cell; FE: Fossil Energy-based; TCO: Total Cost of Ownership; VTOL: Vertical Takeoff and Landing aircraft; VBM: VTOL Body Manufacturing; TFI: Taxes, Fees, and Insurance.


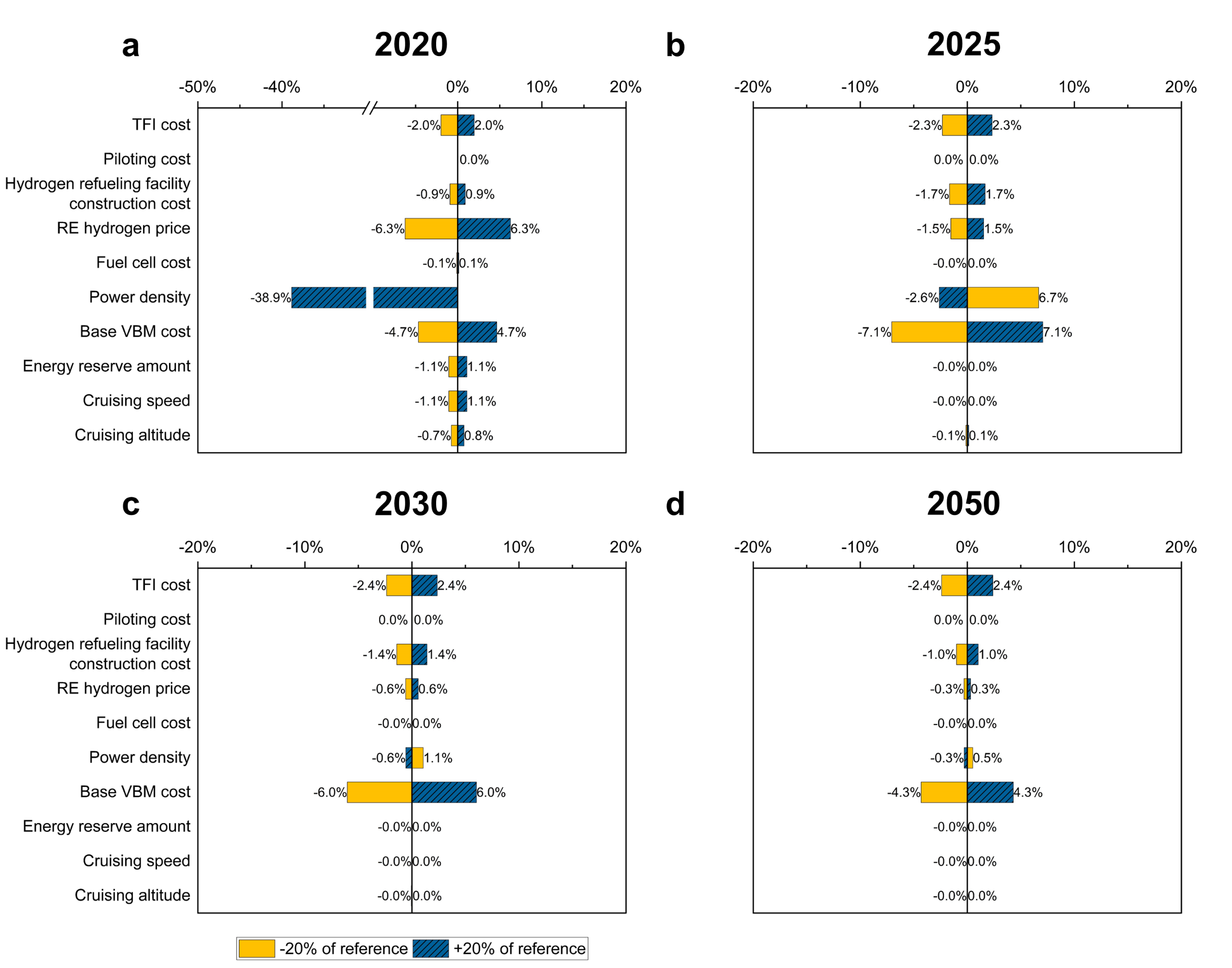


**Fig. S45.** Sensitivity analysis on the TCO of FC-RE private flying cars. The subfigures present the sensitivity of the TCO estimates to changes in VTOL technical parameters (*i.e.*, cruising speed, cruising altitude, energy reserve amount, and power density) and cost parameters (*i.e.*, base VBM cost, fuel cell cost, RE hydrogen price, hydrogen refueling facility construction cost, piloting cost, and TFI cost) in 2020 (a), 2025 (b), 2030 (c), and 2050 (d), respectively. The TCO changes under each parameter at -20% and +20% of its baseline value are shown. FC: Fuel Cell; RE: Renewable Energy-based; TCO: Total Cost of Ownership; VTOL: Vertical Takeoff and Landing aircraft; VBM: VTOL Body Manufacturing; TFI: Taxes, Fees, and Insurance.


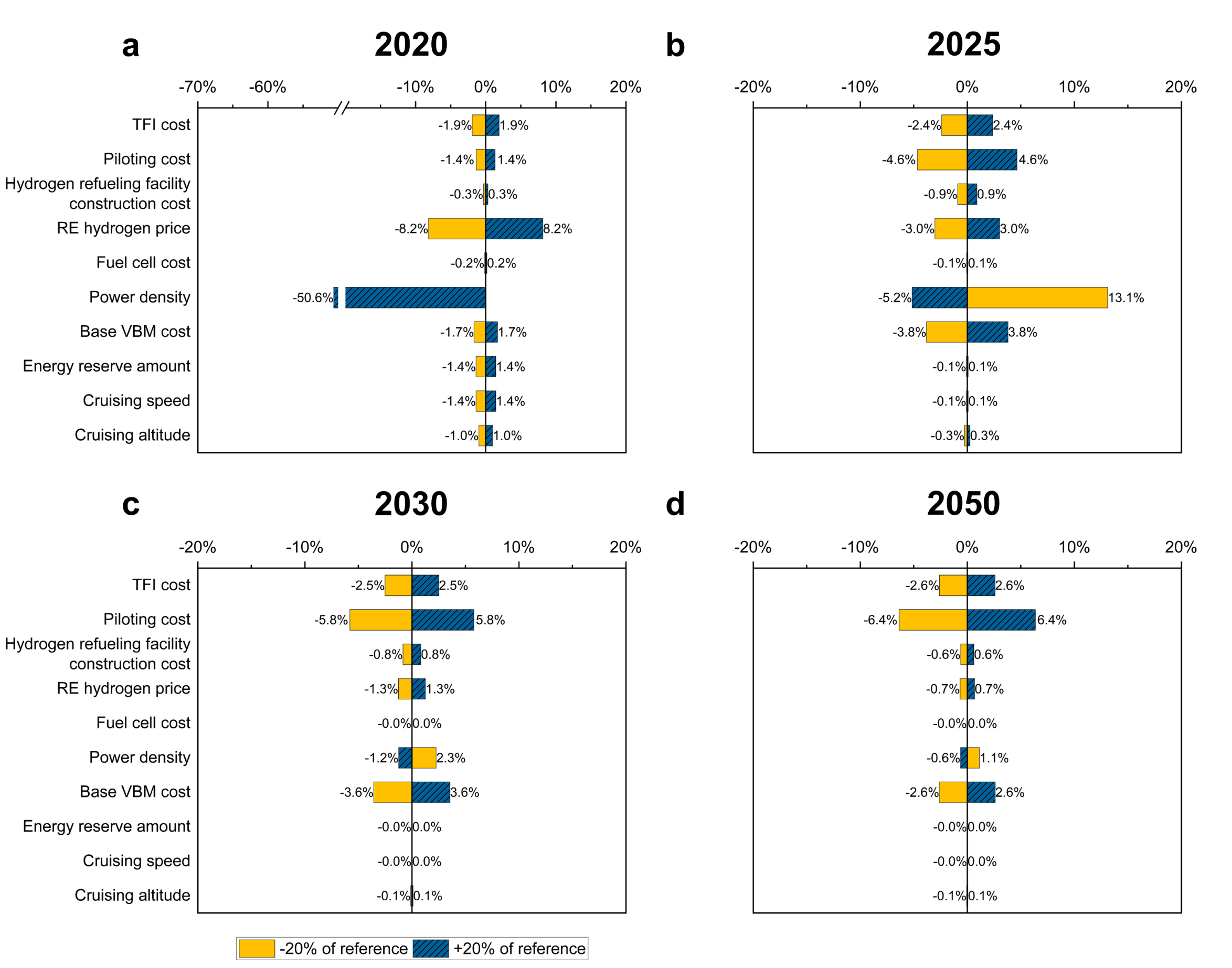


**Fig. S46.** Sensitivity analysis on the TCO of FC-RE short-range air taxis. The subfigures present the sensitivity of the TCO estimates to changes in VTOL technical parameters (*i.e.*, cruising speed, cruising altitude, energy reserve amount, and power density) and cost parameters (*i.e.*, base VBM cost, fuel cell cost, RE hydrogen price, hydrogen refueling facility construction cost, piloting cost, and TFI cost) in 2020 (a), 2025 (b), 2030 (c), and 2050 (d), respectively. The TCO changes under each parameter at -20% and +20% of its baseline value are shown. FC: Fuel Cell; RE: Renewable Energy-based; TCO: Total Cost of Ownership; VTOL: Vertical Takeoff and Landing aircraft; VBM: VTOL Body Manufacturing; TFI: Taxes, Fees, and Insurance.


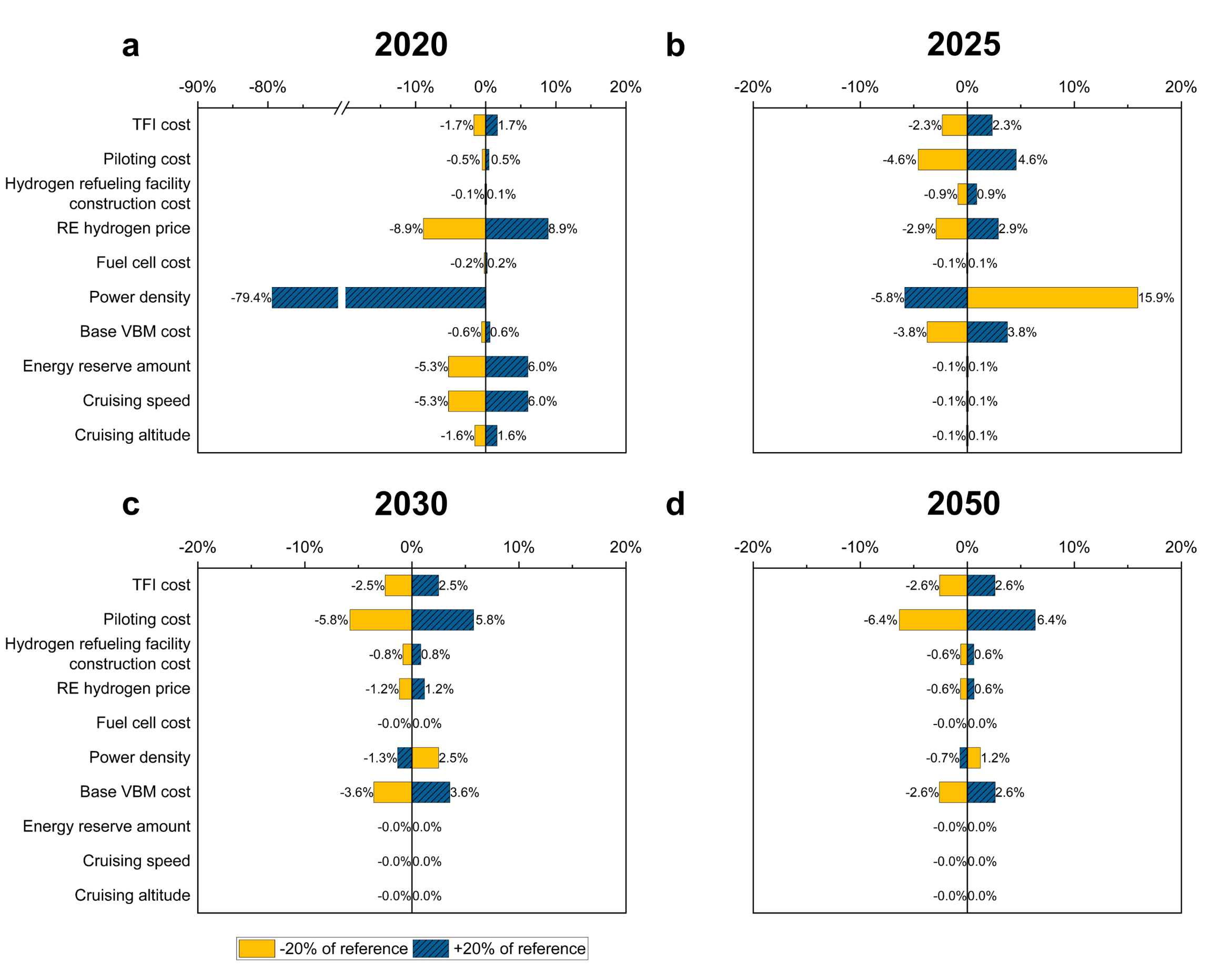


**Fig. S47.** Sensitivity analysis on the TCO of FC-RE long-range air taxis. The subfigures present the sensitivity of the TCO estimates to changes in VTOL technical parameters (*i.e.*, cruising speed, cruising altitude, energy reserve amount, and power density) and cost parameters (*i.e.*, base VBM cost, fuel cell cost, RE hydrogen price, hydrogen refueling facility construction cost, piloting cost, and TFI cost) in 2020 (a), 2025 (b), 2030 (c), and 2050 (d), respectively. The TCO changes under each parameter at -20% and +20% of its baseline value are shown. FC: Fuel Cell; RE: Renewable Energy-based; TCO: Total Cost of Ownership; VTOL: Vertical Takeoff and Landing aircraft; VBM: VTOL Body Manufacturing; TFI: Taxes, Fees, and Insurance.


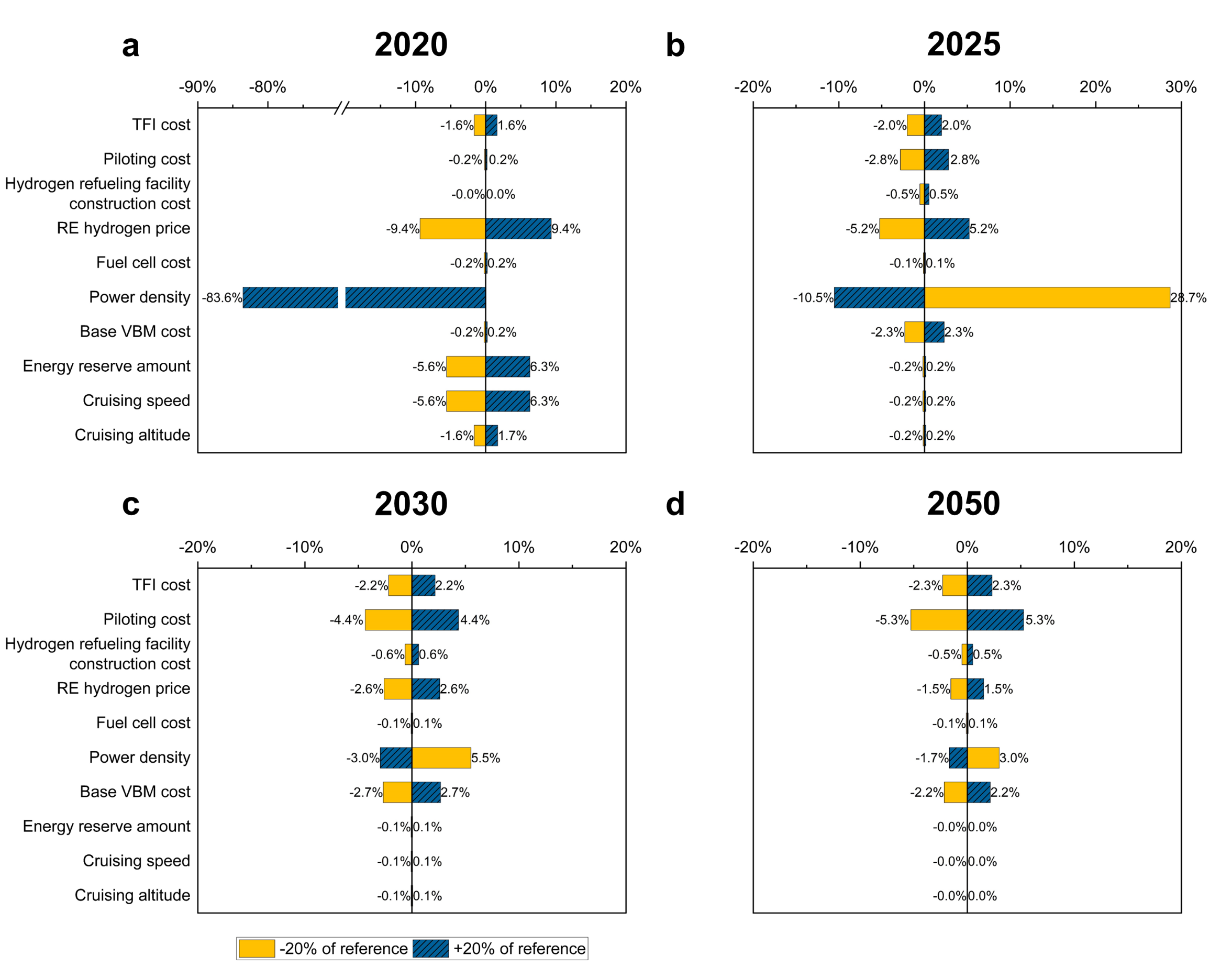


**Fig. S48.** Sensitivity analysis on the TCO of FC-RE air vans. The subfigures present the sensitivity of the TCO estimates to changes in VTOL technical parameters (*i.e.*, cruising speed, cruising altitude, energy reserve amount, and power density) and cost parameters (*i.e.*, base VBM cost, fuel cell cost, RE hydrogen price, hydrogen refueling facility construction cost, piloting cost, and TFI cost) in 2020 (a), 2025 (b), 2030 (c), and 2050 (d), respectively. The TCO changes under each parameter at -20% and +20% of its baseline value are shown. FC: Fuel Cell; RE: Renewable Energy-based; TCO: Total Cost of Ownership; VTOL: Vertical Takeoff and Landing aircraft; VBM: VTOL Body Manufacturing; TFI: Taxes, Fees, and Insurance.


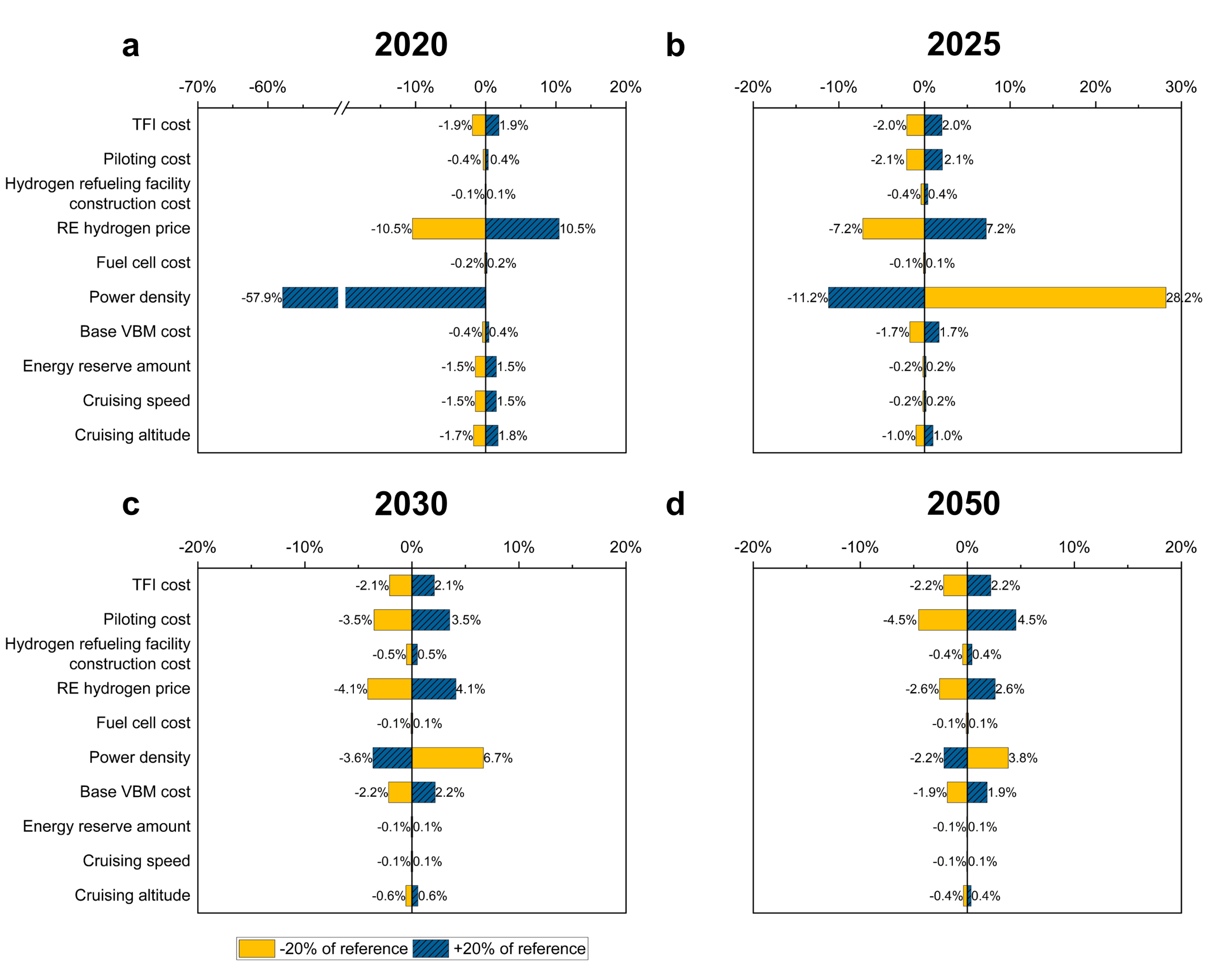


**Fig. S49.** Sensitivity analysis on the TCO of FC-RE airport shuttles. The subfigures present the sensitivity of the TCO estimates to changes in VTOL technical parameters (*i.e.*, cruising speed, cruising altitude, energy reserve amount, and power density) and cost parameters (*i.e.*, base VBM cost, fuel cell cost, RE hydrogen price, hydrogen refueling facility construction cost, piloting cost, and TFI cost) in 2020 (a), 2025 (b), 2030 (c), and 2050 (d), respectively. The TCO changes under each parameter at -20% and +20% of its baseline value are shown. FC: Fuel Cell; RE: Renewable Energy-based; TCO: Total Cost of Ownership; VTOL: Vertical Takeoff and Landing aircraft; VBM: VTOL Body Manufacturing; TFI: Taxes, Fees, and Insurance.


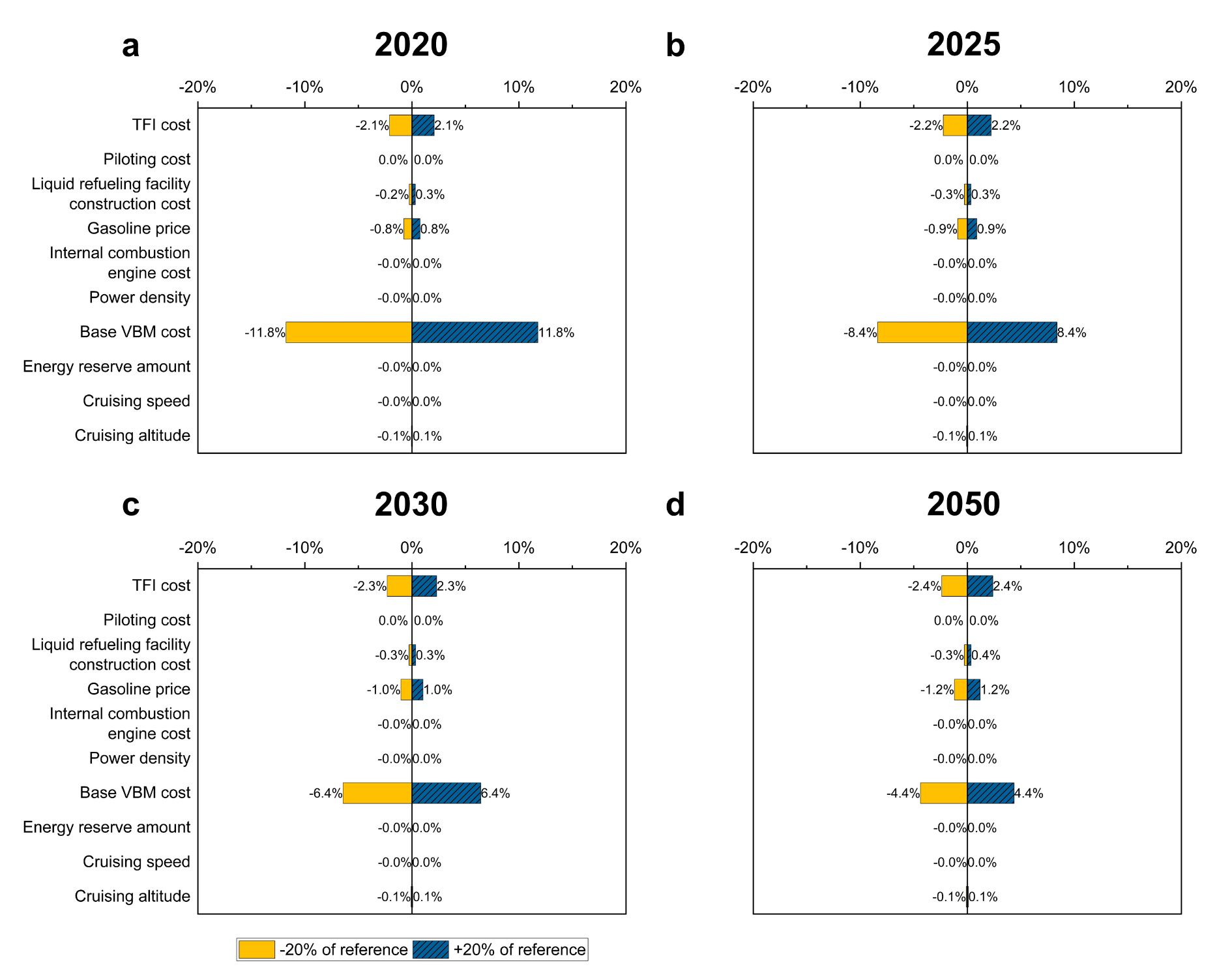


**Fig. S50.** Sensitivity analysis on the TCO of ICE-FE private flying cars. The subfigures present the sensitivity of the TCO estimates to changes in VTOL technical parameters (*i.e.*, cruising speed, cruising altitude, energy reserve amount, and power density) and cost parameters (*i.e.*, base VBM cost, internal combustion engine cost, gasoline price, liquid refueling facility construction cost, piloting cost, and TFI cost) in 2020 (a), 2025 (b), 2030 (c), and 2050 (d), respectively. The TCO changes under each parameter at -20% and +20% of its baseline value are shown. ICE: Internal Combustion Engine; FE: Fossil Energy-based; TCO: Total Cost of Ownership; VTOL: Vertical Takeoff and Landing aircraft; VBM: VTOL Body Manufacturing; TFI: Taxes, Fees, and Insurance.


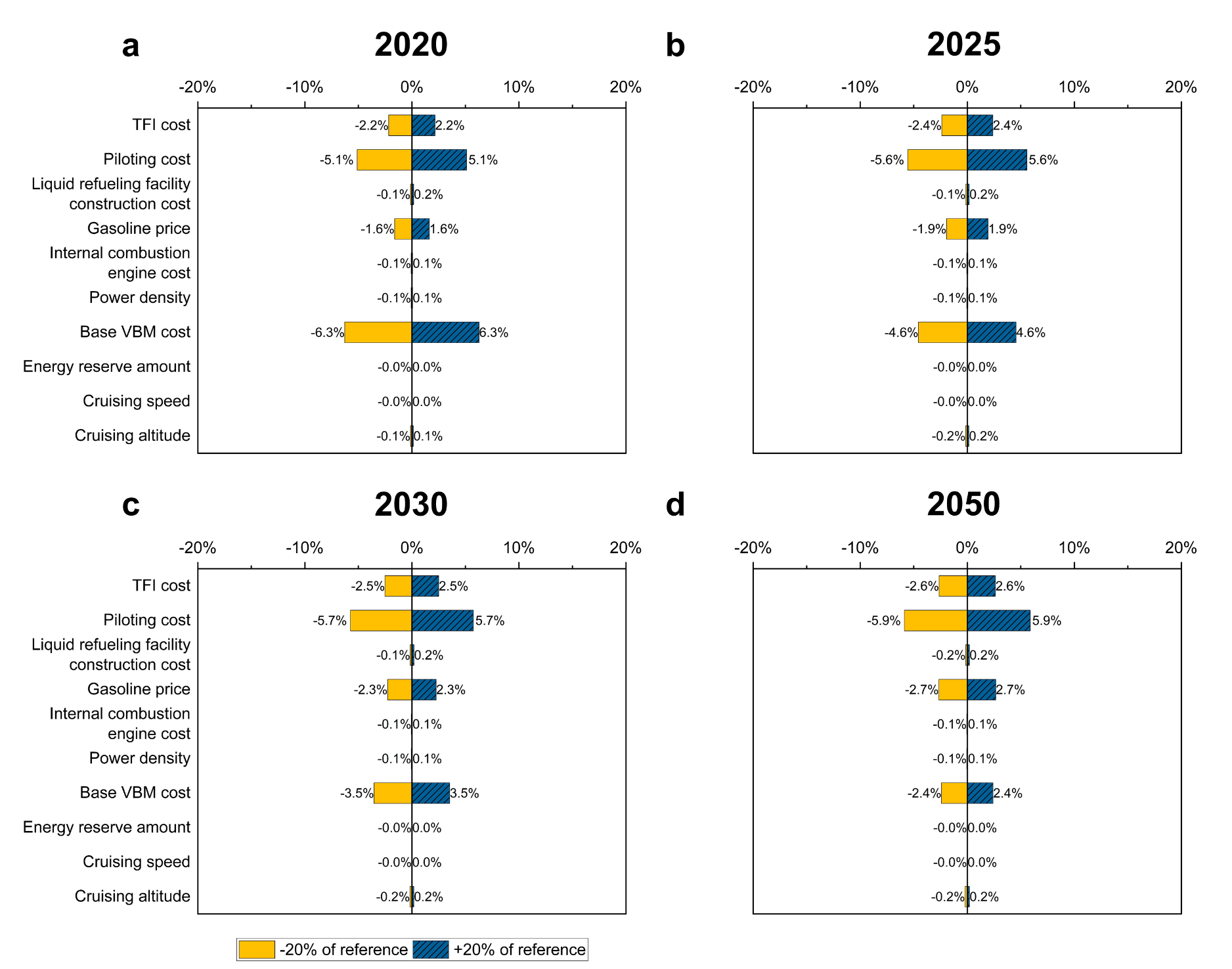


**Fig. S51.** Sensitivity analysis on the TCO of ICE-FE short-range air taxis. The subfigures present the sensitivity of the TCO estimates to changes in VTOL technical parameters (*i.e.*, cruising speed, cruising altitude, energy reserve amount, and power density) and cost parameters (*i.e.*, base VBM cost, internal combustion engine cost, gasoline price, liquid refueling facility construction cost, piloting cost, and TFI cost) in 2020 (a), 2025 (b), 2030 (c), and 2050 (d), respectively. The TCO changes under each parameter at -20% and +20% of its baseline value are shown. ICE: Internal Combustion Engine; FE: Fossil Energy-based; TCO: Total Cost of Ownership; VTOL: Vertical Takeoff and Landing aircraft; VBM: VTOL Body Manufacturing; TFI: Taxes, Fees, and Insurance.


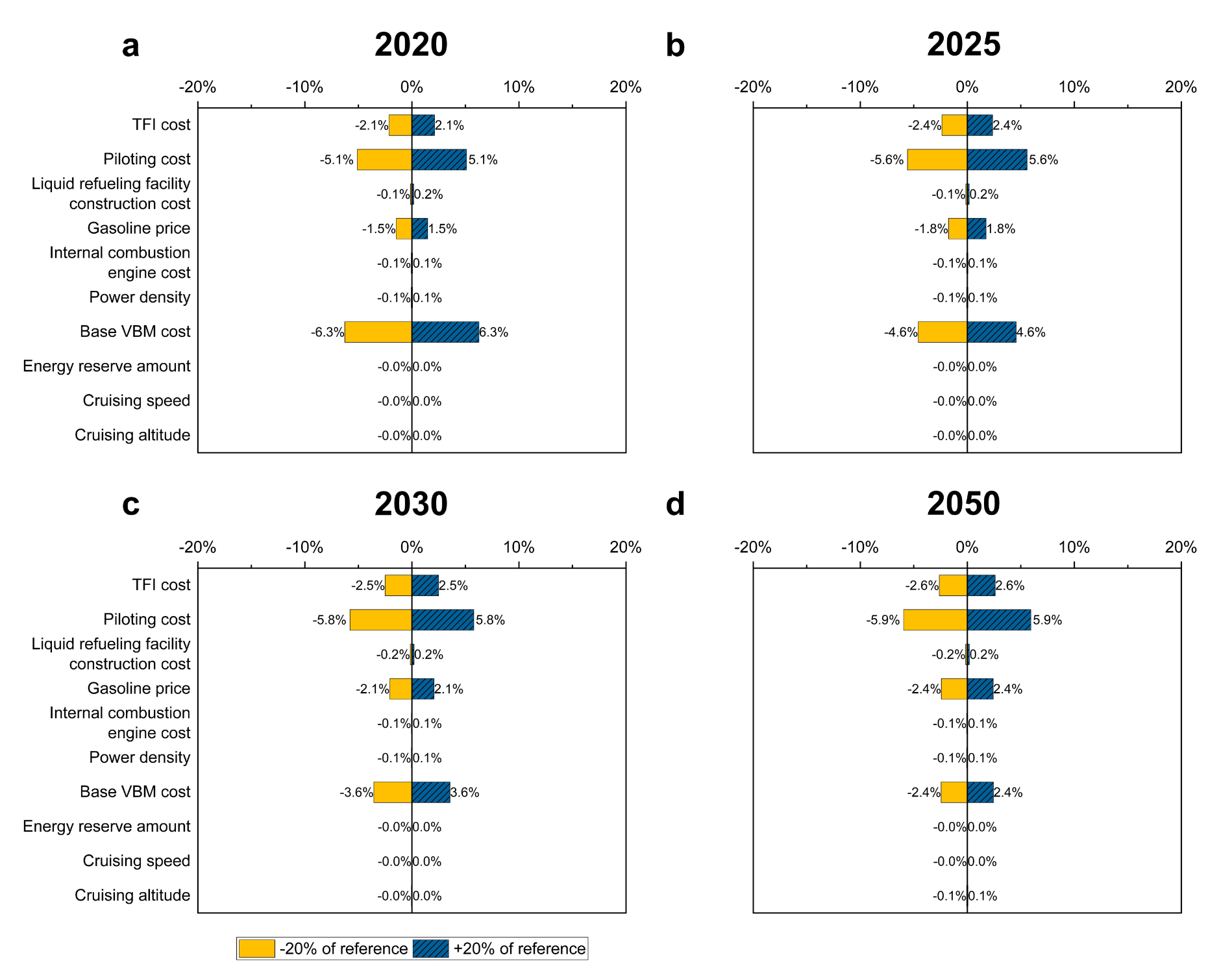


**Fig. S52.** Sensitivity analysis on the TCO of ICE-FE long-range air taxis. The subfigures present the sensitivity of the TCO estimates to changes in VTOL technical parameters (*i.e.*, cruising speed, cruising altitude, energy reserve amount, and power density) and cost parameters (*i.e.*, base VBM cost, internal combustion engine cost, gasoline price, liquid refueling facility construction cost, piloting cost, and TFI cost) in 2020 (a), 2025 (b), 2030 (c), and 2050 (d), respectively. The TCO changes under each parameter at -20% and +20% of its baseline value are shown. ICE: Internal Combustion Engine; FE: Fossil Energy-based; TCO: Total Cost of Ownership; VTOL: Vertical Takeoff and Landing aircraft; VBM: VTOL Body Manufacturing; TFI: Taxes, Fees, and Insurance.


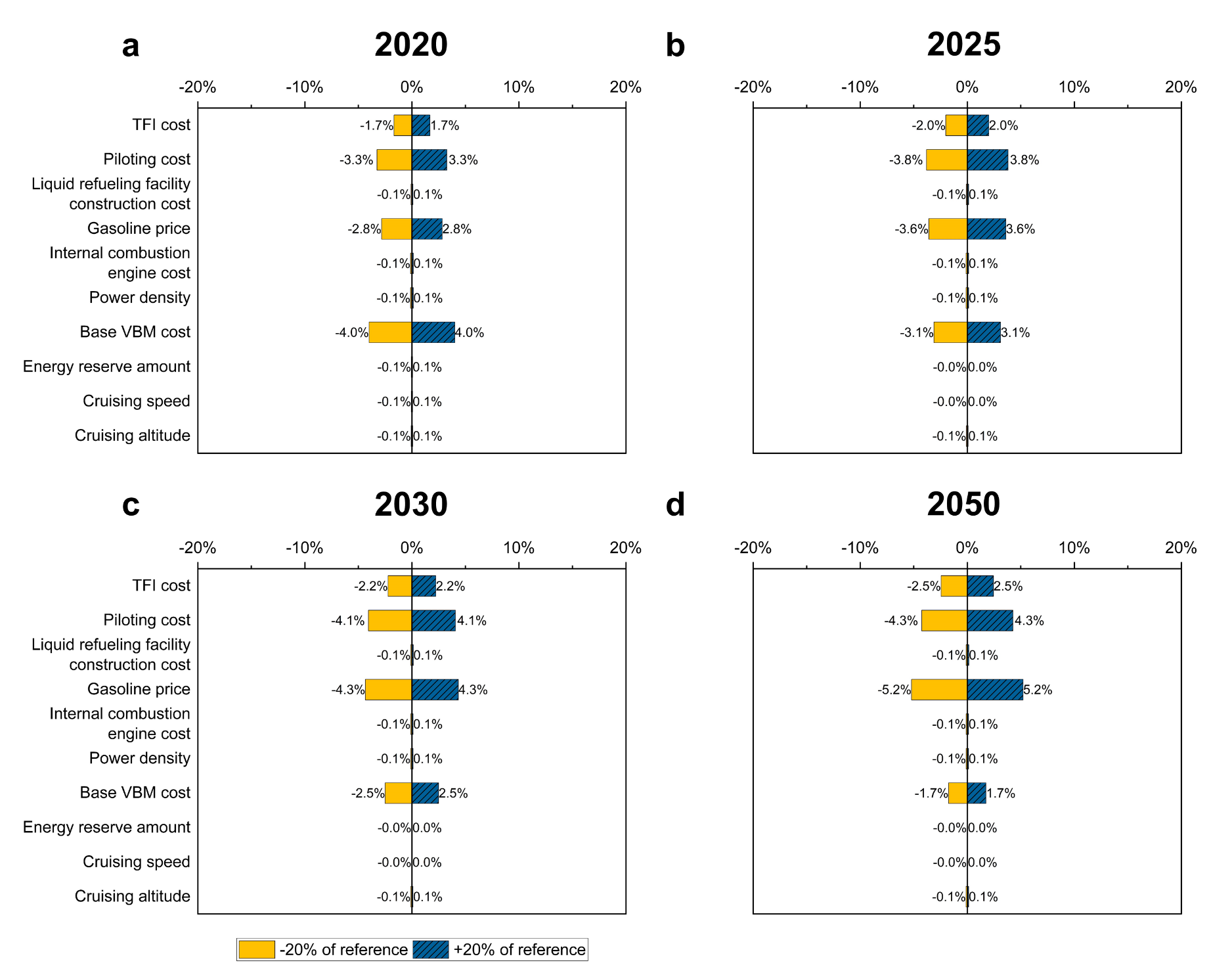


**Fig. S53.** Sensitivity analysis on the TCO of ICE-FE air vans. The subfigures present the sensitivity of the TCO estimates to changes in VTOL technical parameters (*i.e.*, cruising speed, cruising altitude, energy reserve amount, and power density) and cost parameters (*i.e.*, base VBM cost, internal combustion engine cost, gasoline price, liquid refueling facility construction cost, piloting cost, and TFI cost) in 2020 (a), 2025 (b), 2030 (c), and 2050 (d), respectively. The TCO changes under each parameter at -20% and +20% of its baseline value are shown. ICE: Internal Combustion Engine; FE: Fossil Energy-based; TCO: Total Cost of Ownership; VTOL: Vertical Takeoff and Landing aircraft; VBM: VTOL Body Manufacturing; TFI: Taxes, Fees, and Insurance.


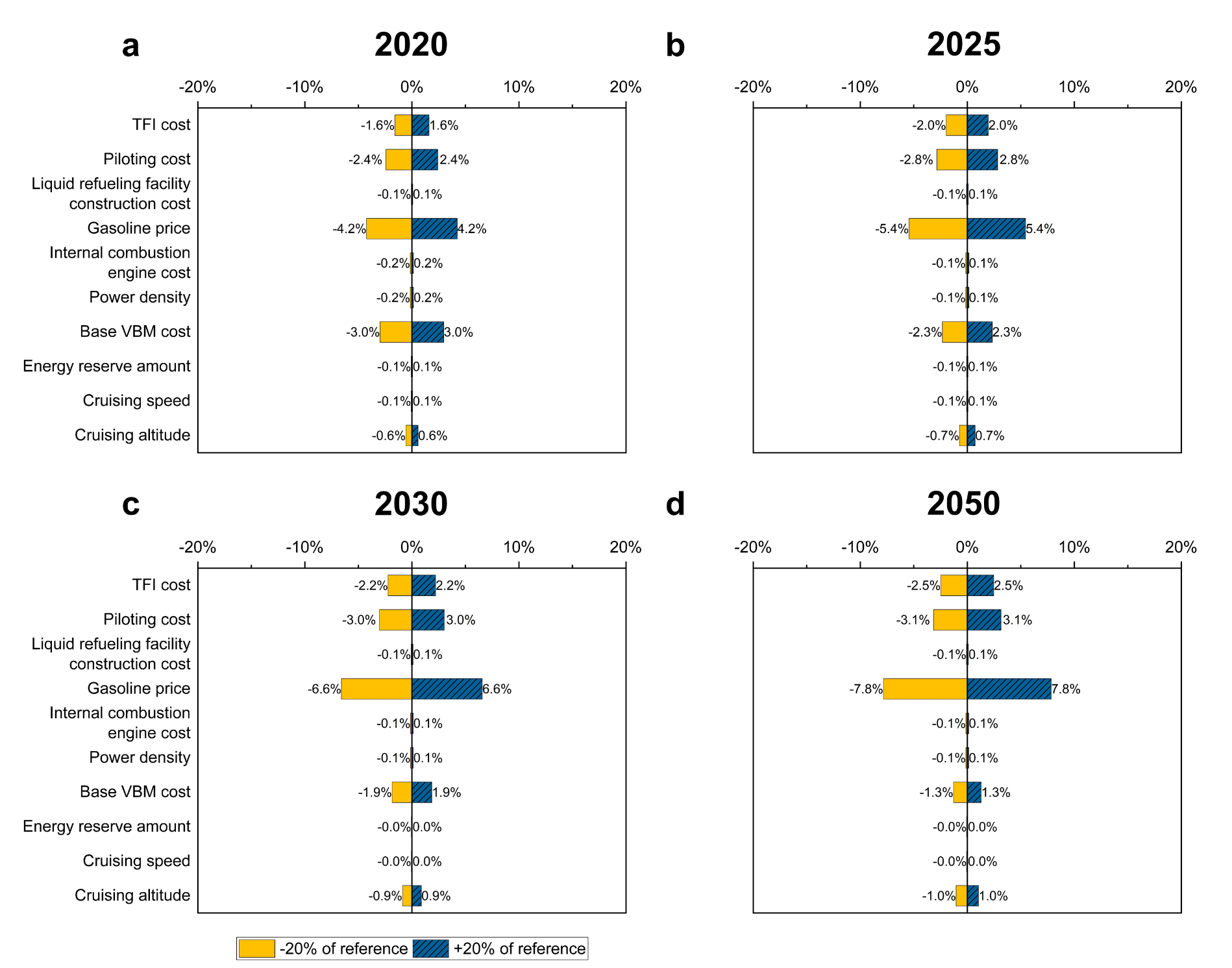


**Fig. S54.** Sensitivity analysis on the TCO of ICE-FE airport shuttles. The subfigures present the sensitivity of the TCO estimates to changes in VTOL technical parameters (*i.e.*, cruising speed, cruising altitude, energy reserve amount, and power density) and cost parameters (*i.e.*, base VBM cost, internal combustion engine cost, gasoline price, liquid refueling facility construction cost, piloting cost, and TFI cost) in 2020 (a), 2025 (b), 2030 (c), and 2050 (d), respectively. The TCO changes under each parameter at -20% and +20% of its baseline value are shown. ICE: Internal Combustion Engine; FE: Fossil Energy-based; TCO: Total Cost of Ownership; VTOL: Vertical Takeoff and Landing aircraft; VBM: VTOL Body Manufacturing; TFI: Taxes, Fees, and Insurance.


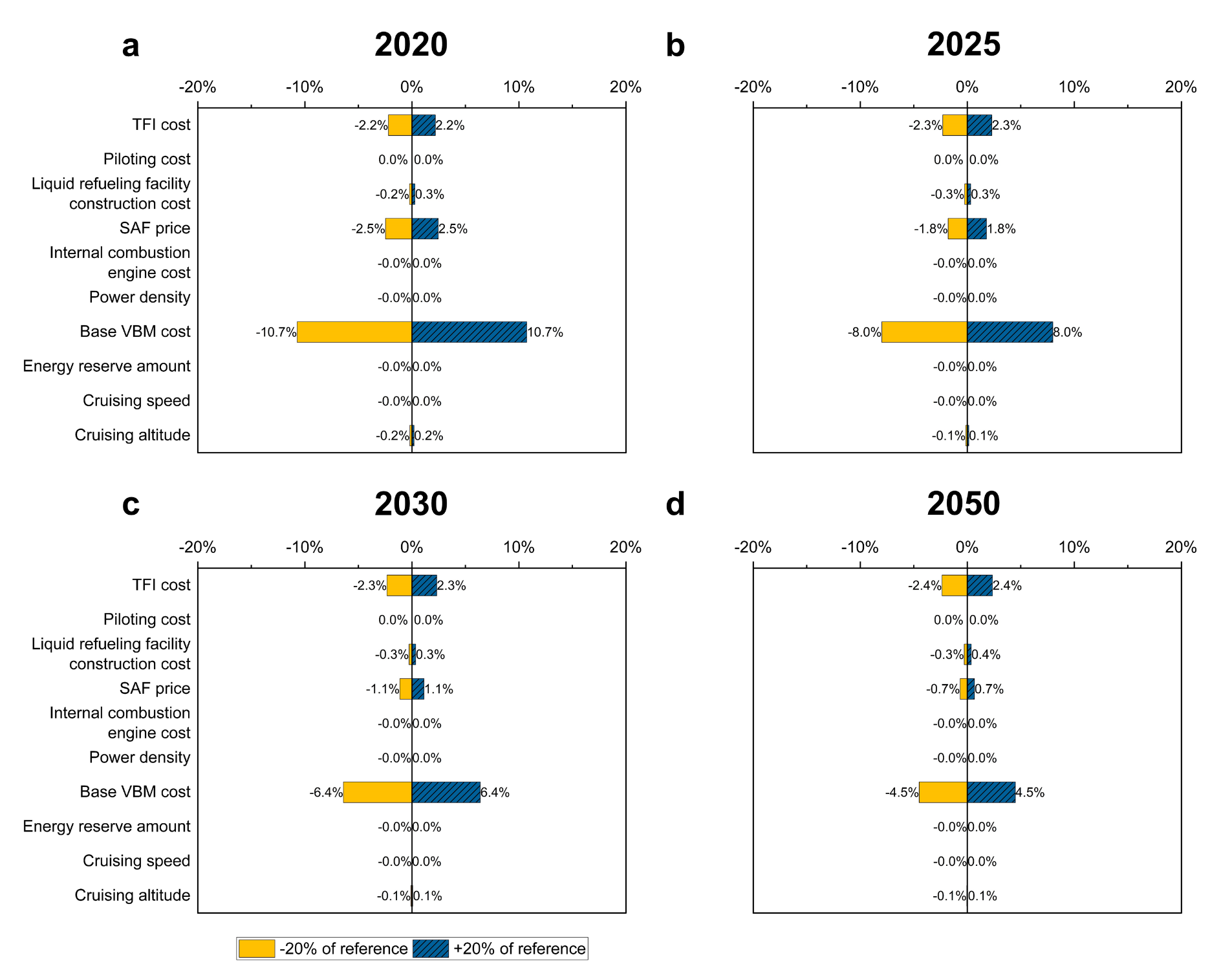


**Fig. S55.** Sensitivity analysis on the TCO of ICE-RE private flying cars. The subfigures present the sensitivity of the TCO estimates to changes in VTOL technical parameters (*i.e.*, cruising speed, cruising altitude, energy reserve amount, and power density) and cost parameters (*i.e.*, base VBM cost, internal combustion engine cost, SAF price, liquid refueling facility construction cost, piloting cost, and TFI cost) in 2020 (a), 2025 (b), 2030 (c), and 2050 (d), respectively. The TCO changes under each parameter at -20% and +20% of its baseline value are shown. ICE: Internal Combustion Engine; RE: Renewable Energy-based; TCO: Total Cost of Ownership; VTOL: Vertical Takeoff and Landing aircraft; VBM: VTOL Body Manufacturing; TFI: Taxes, Fees, and Insurance; SAF: Sustainable Aviation Fuel.


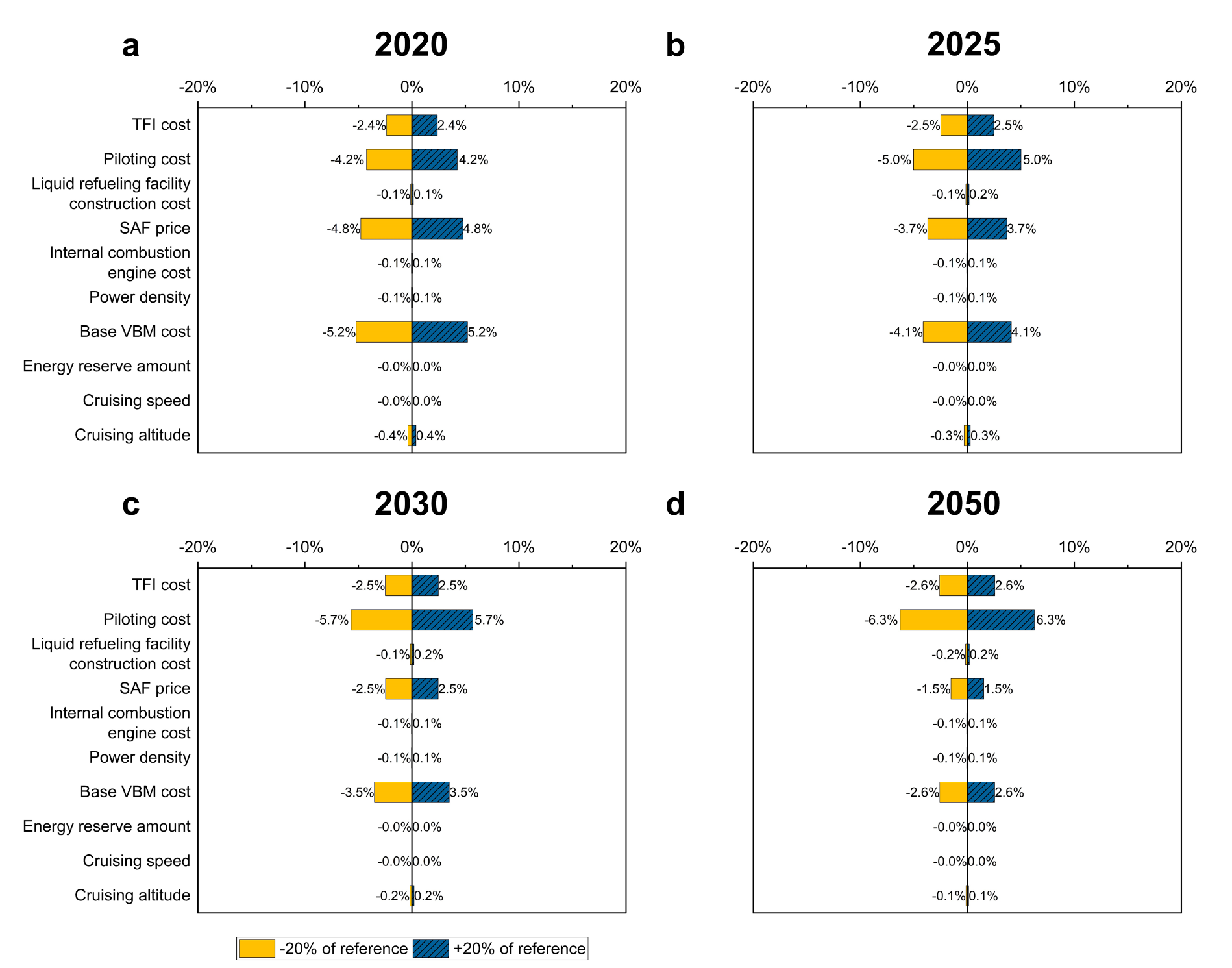


**Fig. S56.** Sensitivity analysis on the TCO of ICE-RE short-range air taxis. The subfigures present the sensitivity of the TCO estimates to changes in VTOL technical parameters (*i.e.*, cruising speed, cruising altitude, energy reserve amount, and power density) and cost parameters (*i.e.*, base VBM cost, internal combustion engine cost, SAF price, liquid refueling facility construction cost, piloting cost, and TFI cost) in 2020 (a), 2025 (b), 2030 (c), and 2050 (d), respectively. The TCO changes under each parameter at -20% and +20% of its baseline value are shown. ICE: Internal Combustion Engine; RE: Renewable Energy-based; TCO: Total Cost of Ownership; VTOL: Vertical Takeoff and Landing aircraft; VBM: VTOL Body Manufacturing; TFI: Taxes, Fees, and Insurance; SAF: Sustainable Aviation Fuel.


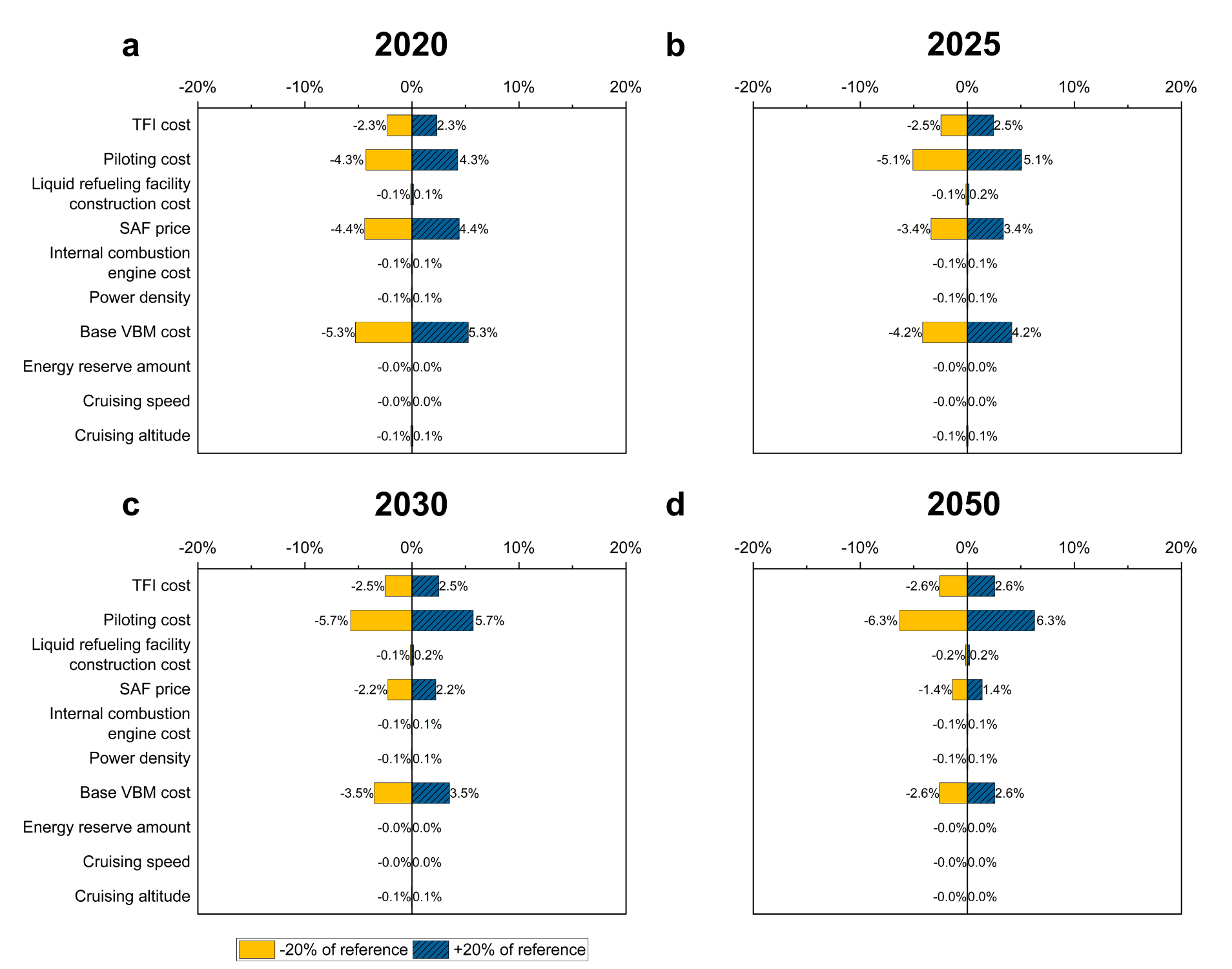


**Fig. S57.** Sensitivity analysis on the TCO of ICE-RE long-range air taxis. The subfigures present the sensitivity of the TCO estimates to changes in VTOL technical parameters (*i.e.*, cruising speed, cruising altitude, energy reserve amount, and power density) and cost parameters (*i.e.*, base VBM cost, internal combustion engine cost, SAF price, liquid refueling facility construction cost, piloting cost, and TFI cost) in 2020 (a), 2025 (b), 2030 (c), and 2050 (d), respectively. The TCO changes under each parameter at -20% and +20% of its baseline value are shown. ICE: Internal Combustion Engine; RE: Renewable Energy-based; TCO: Total Cost of Ownership; VTOL: Vertical Takeoff and Landing aircraft; VBM: VTOL Body Manufacturing; TFI: Taxes, Fees, and Insurance; SAF: Sustainable Aviation Fuel.


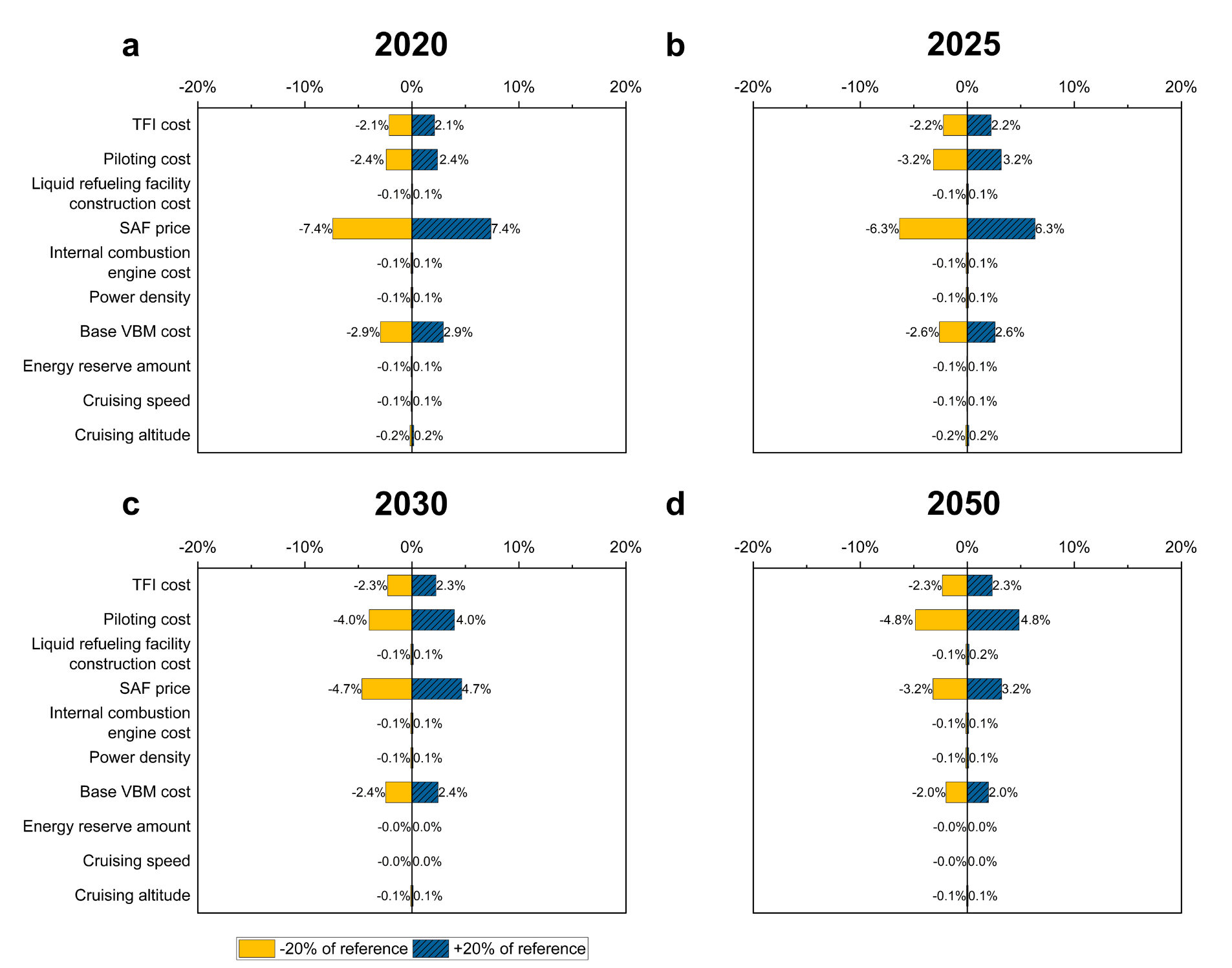


**Fig. S58.** Sensitivity analysis on the TCO of ICE-RE air vans. The subfigures present the sensitivity of the TCO estimates to changes in VTOL technical parameters (*i.e.*, cruising speed, cruising altitude, energy reserve amount, and power density) and cost parameters (*i.e.*, base VBM cost, internal combustion engine cost, SAF price, liquid refueling facility construction cost, piloting cost, and TFI cost) in 2020 (a), 2025 (b), 2030 (c), and 2050 (d), respectively. The TCO changes under each parameter at -20% and +20% of its baseline value are shown. ICE: Internal Combustion Engine; RE: Renewable Energy-based; TCO: Total Cost of Ownership; VTOL: Vertical Takeoff and Landing aircraft; VBM: VTOL Body Manufacturing; TFI: Taxes, Fees, and Insurance; SAF: Sustainable Aviation Fuel.


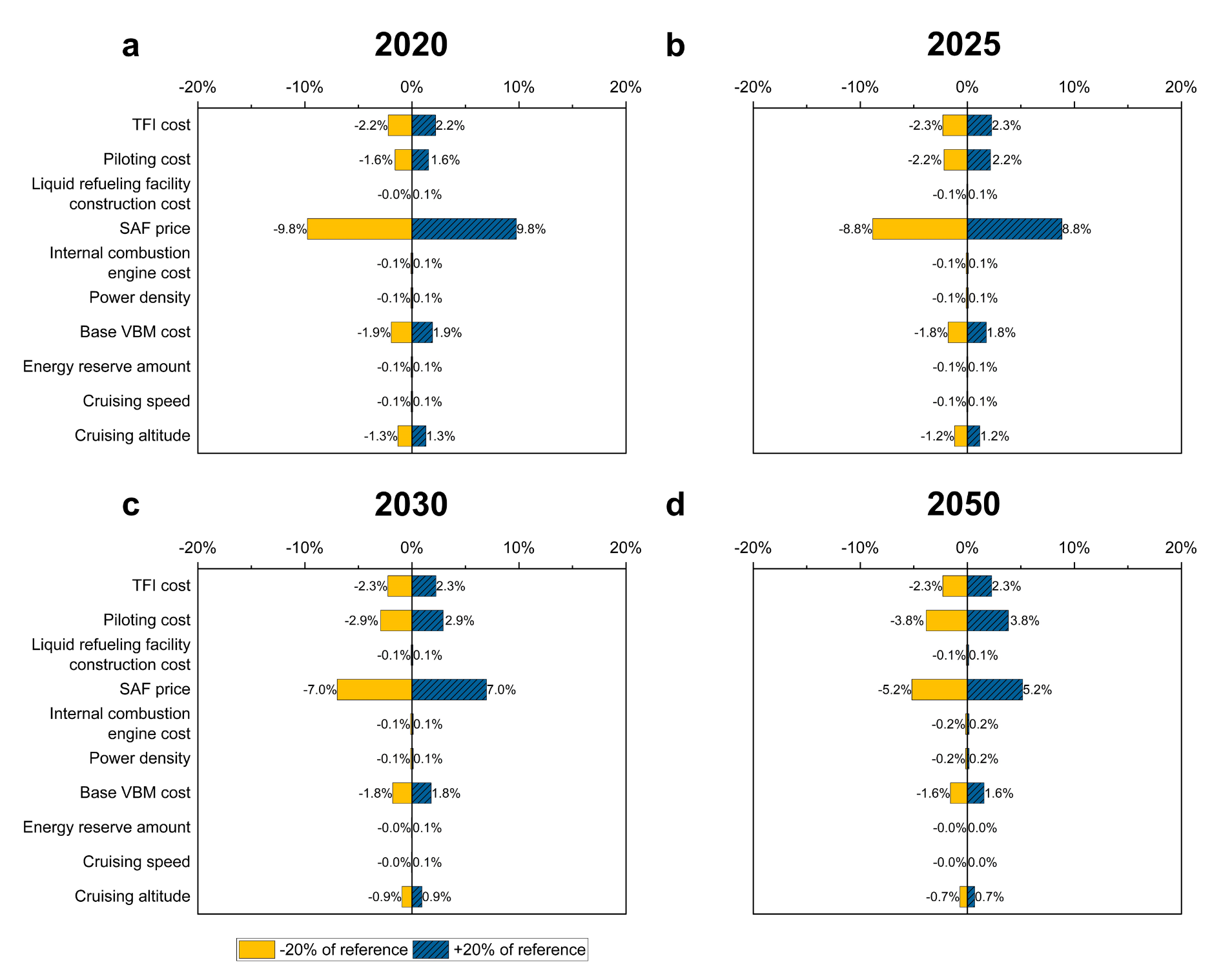


**Fig. S59.** Sensitivity analysis on the TCO of ICE-RE airport shuttles. The subfigures present the sensitivity of the TCO estimates to changes in VTOL technical parameters (*i.e.*, cruising speed, cruising altitude, energy reserve amount, and power density) and cost parameters (*i.e.*, base VBM cost, internal combustion engine cost, SAF price, liquid refueling facility construction cost, piloting cost, and TFI cost) in 2020 (a), 2025 (b), 2030 (c), and 2050 (d), respectively. The TCO changes under each parameter at -20% and +20% of its baseline value are shown. ICE: Internal Combustion Engine; RE: Renewable Energy-based; TCO: Total Cost of Ownership; VTOL: Vertical Takeoff and Landing aircraft; VBM: VTOL Body Manufacturing; TFI: Taxes, Fees, and Insurance; SAF: Sustainable Aviation Fuel.


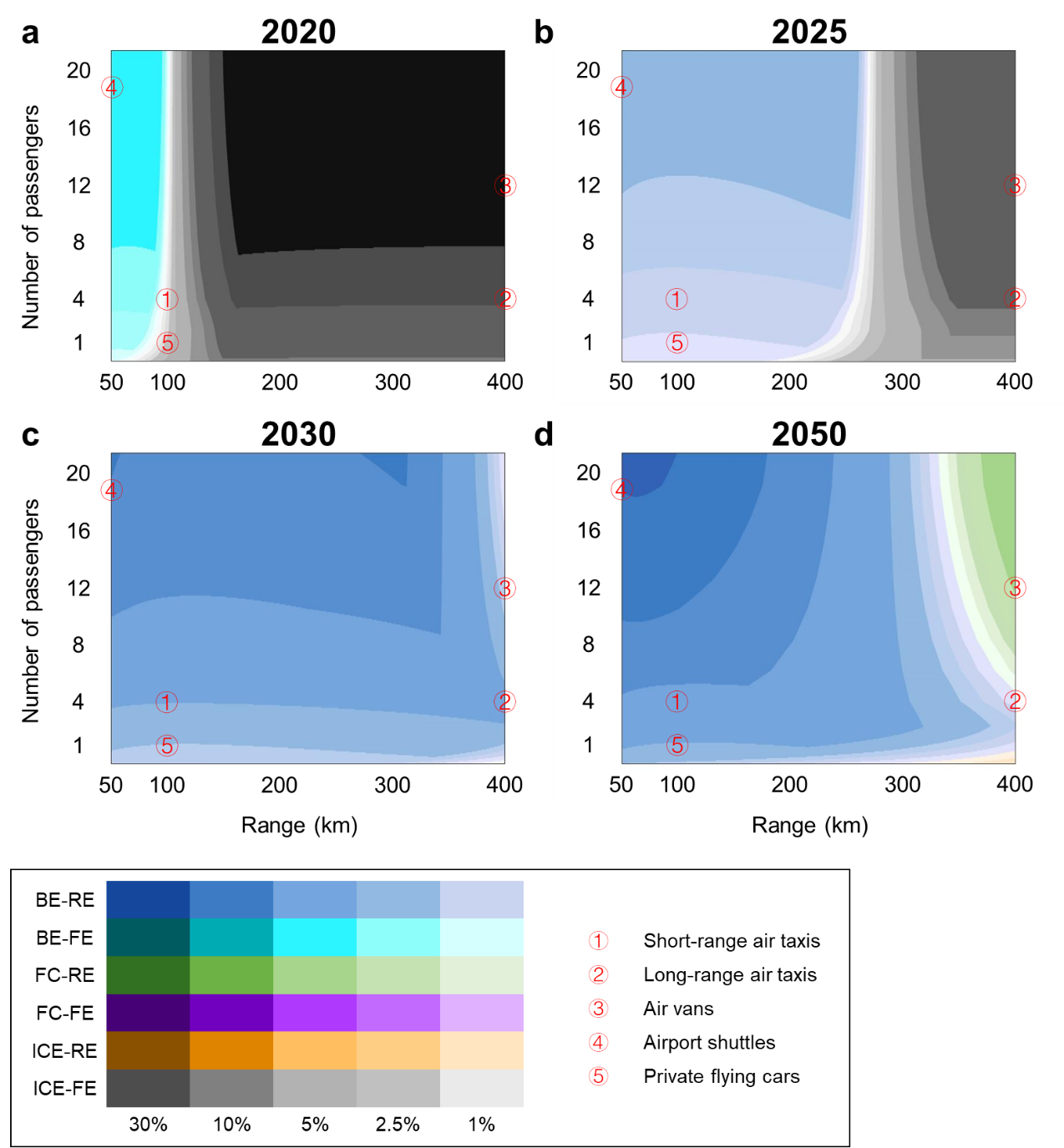


**Fig. S60.** Sensitivity analysis on TCO-advantageous technologies (battery life: -20%). The results are presented under assumed technology improvements for the years of 2020 (a), 2025 (b), 2030 (c), and 2050 (d), respectively. The TCO-advantageous technology for a given application domain is indicated by the color of the area. Color shading indicates the relative advantage of the leading technology against the second-best technology. A darker color indicates a stronger advantage and vice versa. In each subfigure, typical applications of VTOLs, including air taxis, air vans, airport shuttles, and private flying cars, are indicated using circled numbers. BE: Battery Electric; FC: Fuel Cell; ICE: Internal Combustion Engine; FE: Fossil Energy-based; RE: Renewable Energy-based.


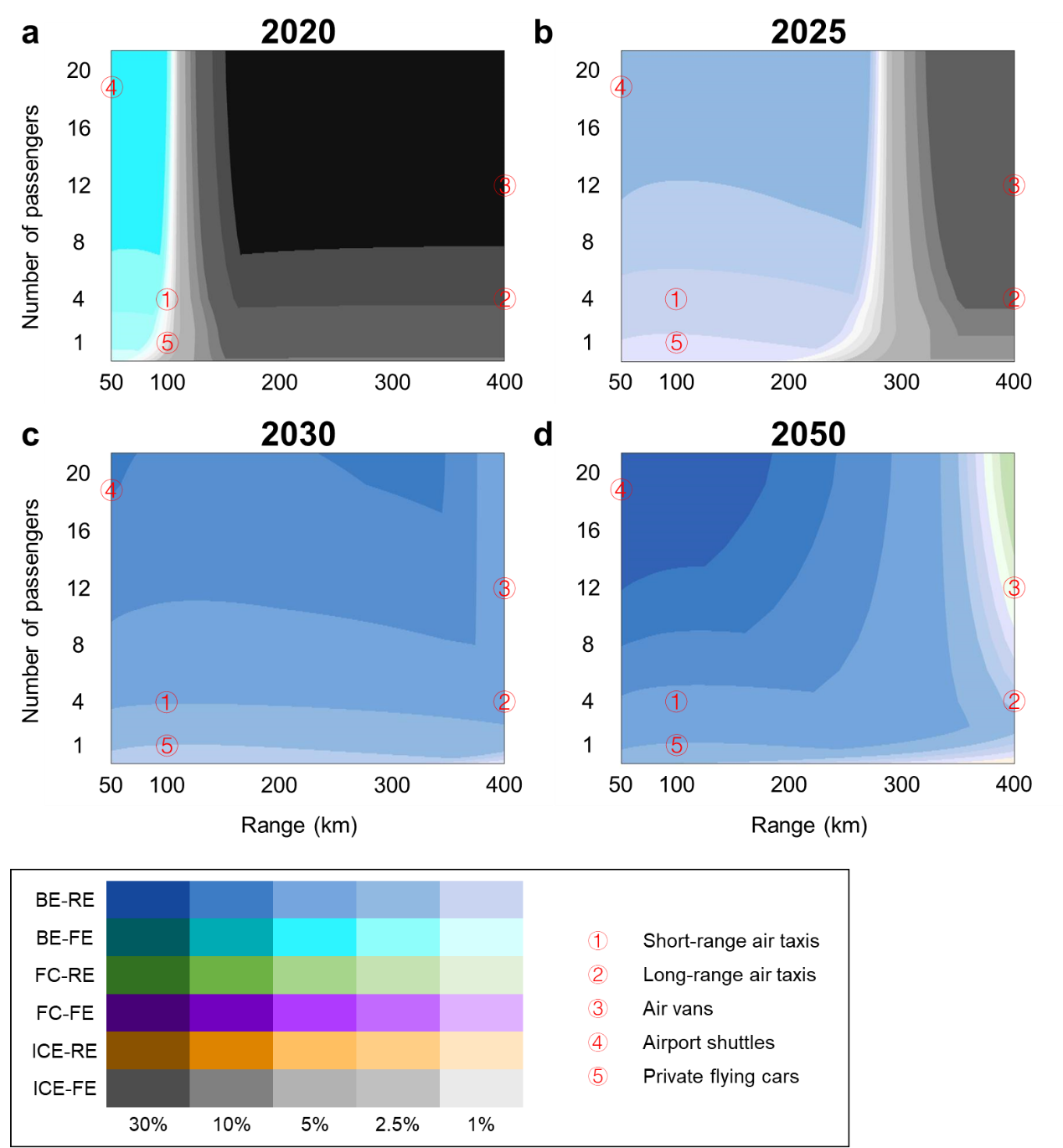


**Fig. S61.** Sensitivity analysis on TCO-advantageous technologies (battery life: +20%). The results are presented under assumed technology improvements for the years of 2020 (a), 2025 (b), 2030 (c), and 2050 (d), respectively. The TCO-advantageous technology for a given application domain is indicated by the color of the area. Color shading indicates the relative advantage of the leading technology against the second-best technology. A darker color indicates a stronger advantage and vice versa. In each subfigure, typical applications of VTOLs, including air taxis, air vans, airport shuttles, and private flying cars, are indicated using circled numbers. BE: Battery Electric; FC: Fuel Cell; ICE: Internal Combustion Engine; FE: Fossil Energy-based; RE: Renewable Energy-based.


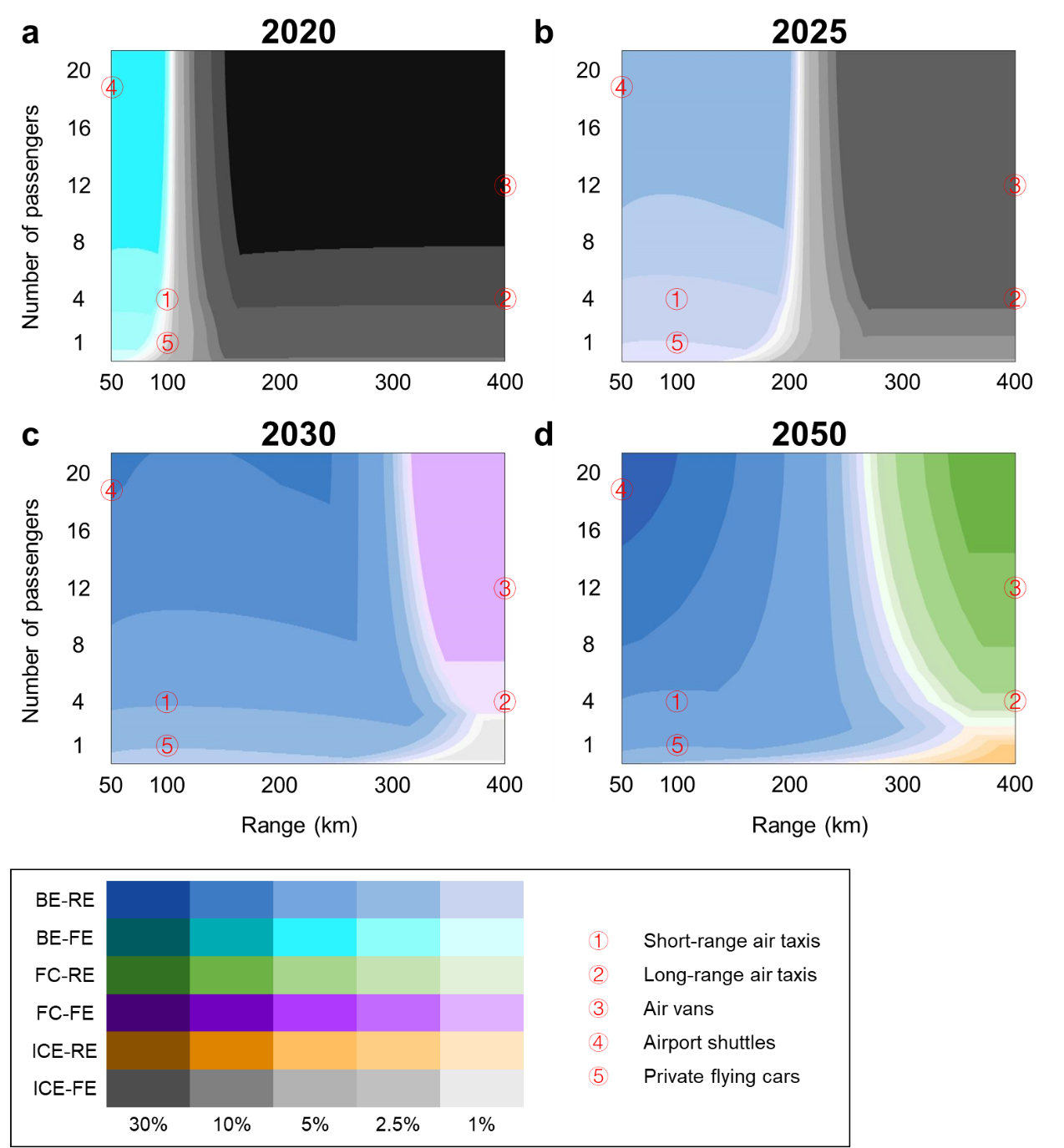


**Fig. S62.** Sensitivity analysis on TCO-advantageous technologies (battery energy density: -20%). The results are presented under assumed technology improvements for the years of 2020 (a), 2025 (b), 2030 (c), and 2050 (d), respectively. The TCO-advantageous technology for a given application domain is indicated by the color of the area. Color shading indicates the relative advantage of the leading technology against the second-best technology. A darker color indicates a stronger advantage and vice versa. In each subfigure, typical applications of VTOLs, including air taxis, air vans, airport shuttles, and private flying cars, are indicated using circled numbers. BE: Battery Electric; FC: Fuel Cell; ICE: Internal Combustion Engine; FE: Fossil Energy-based; RE: Renewable Energy-based.


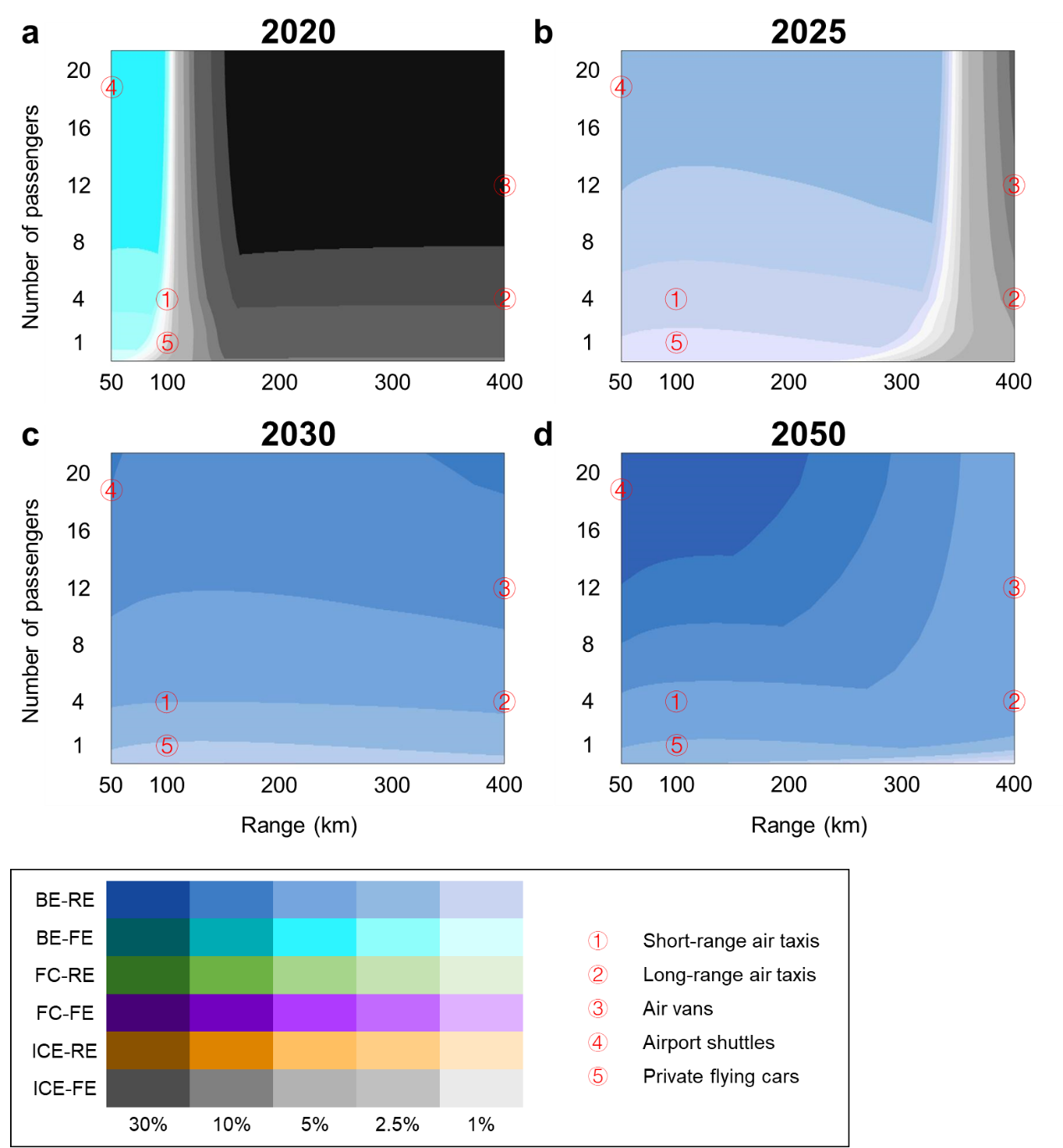


**Fig. S63.** Sensitivity analysis on TCO-advantageous technologies (battery energy density: +20%). The results are presented under assumed technology improvements for the years of 2020 (a), 2025 (b), 2030 (c), and 2050 (d), respectively. The TCO-advantageous technology for a given application domain is indicated by the color of the area. Color shading indicates the relative advantage of the leading technology against the second-best technology. A darker color indicates a stronger advantage and vice versa. In each subfigure, typical applications of VTOLs, including air taxis, air vans, airport shuttles, and private flying cars, are indicated using circled numbers. BE: Battery Electric; FC: Fuel Cell; ICE: Internal Combustion Engine; FE: Fossil Energy-based; RE: Renewable Energy-based.


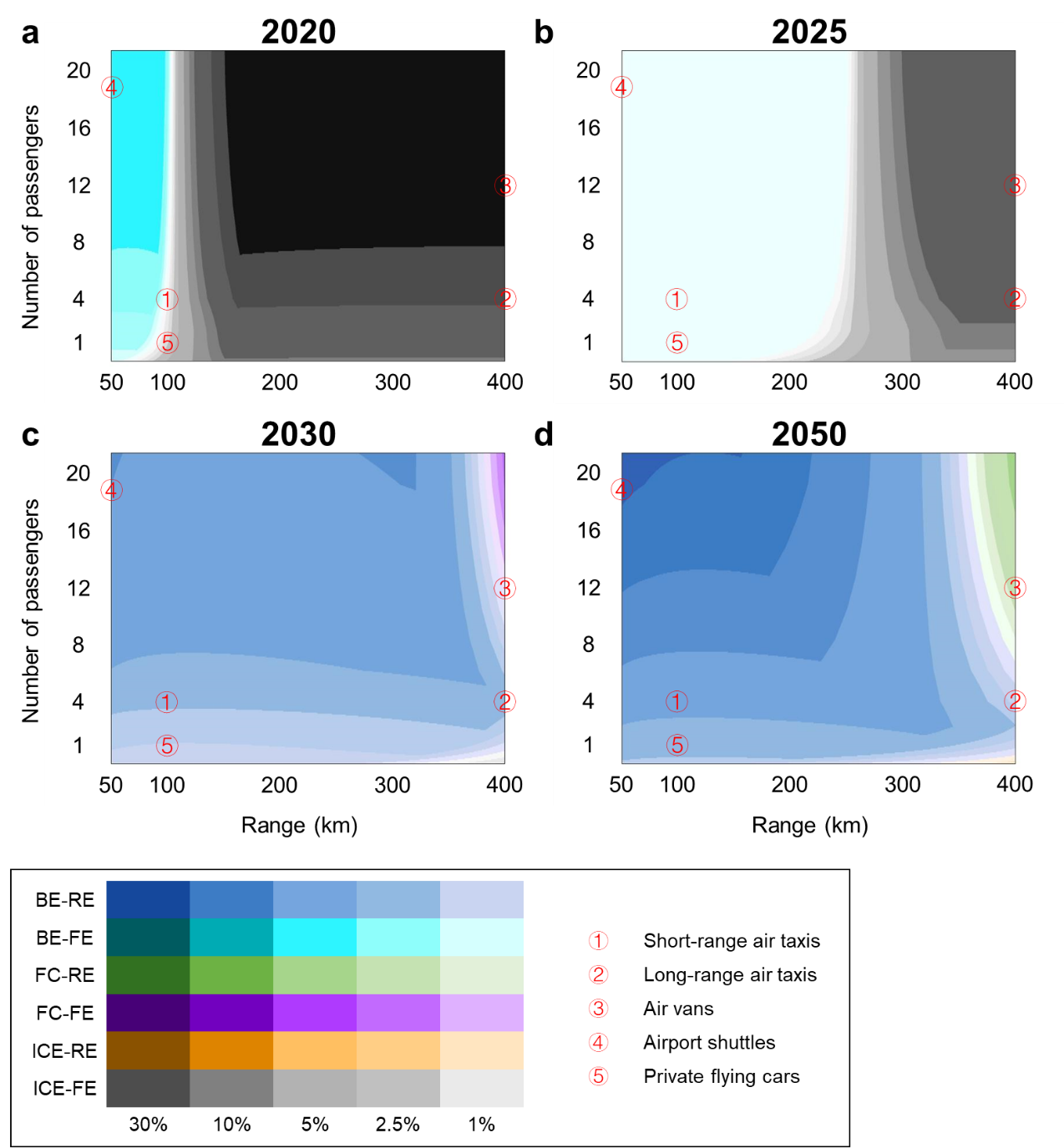


**Fig. S64.** Sensitivity analysis on TCO-advantageous technologies (FE energy price: -20%). The results are presented under assumed technology improvements for the years of 2020 (a), 2025 (b), 2030 (c), and 2050 (d), respectively. The TCO-advantageous technology for a given application domain is indicated by the color of the area. Color shading indicates the relative advantage of the leading technology against the second-best technology. A darker color indicates a stronger advantage and vice versa. In each subfigure, typical applications of VTOLs, including air taxis, air vans, airport shuttles, and private flying cars, are indicated using circled numbers. BE: Battery Electric; FC: Fuel Cell; ICE: Internal Combustion Engine; FE: Fossil Energy-based; RE: Renewable Energy-based.


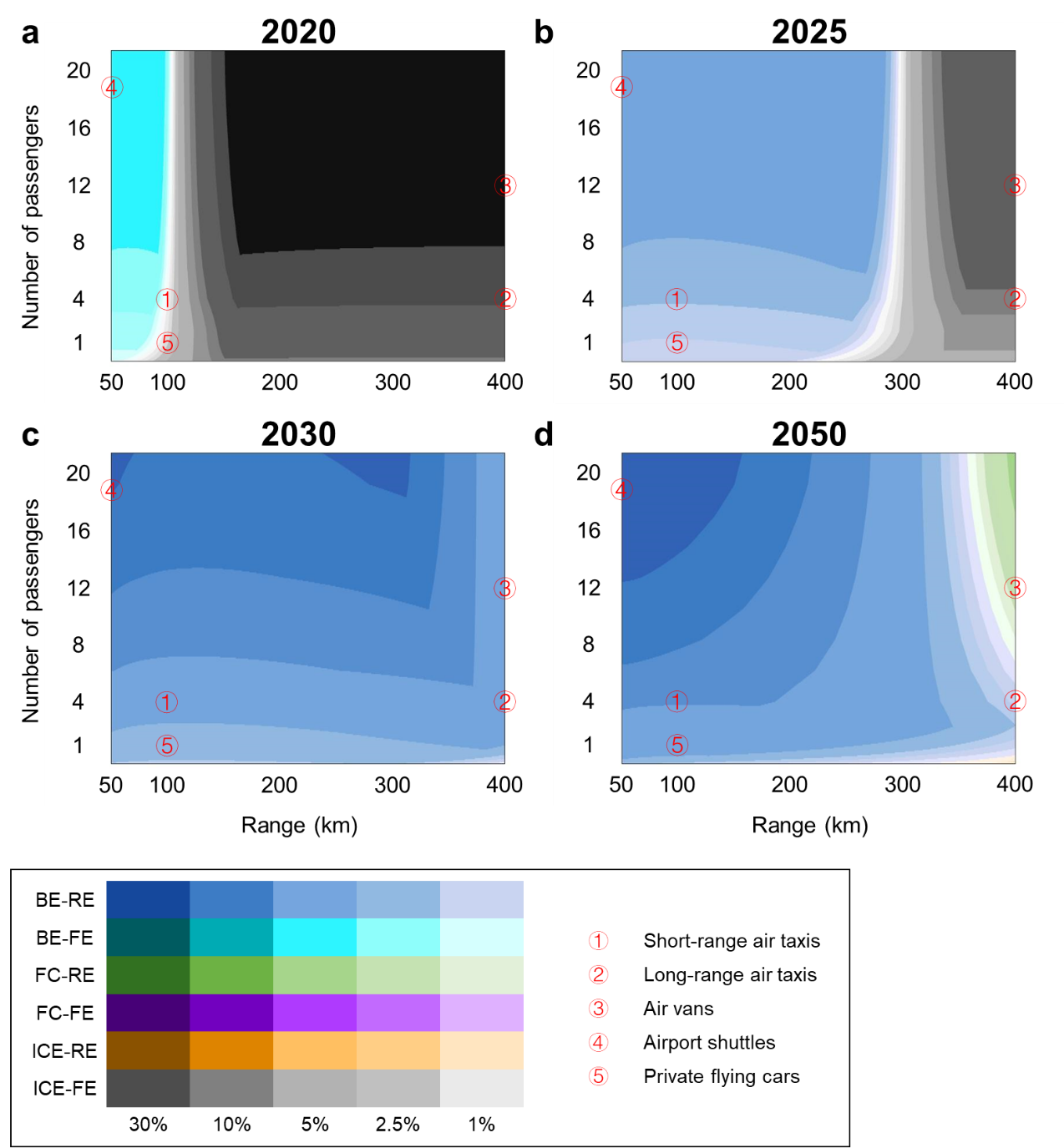


**Fig. S65.** Sensitivity analysis on TCO-advantageous technologies (FE energy price: +20%). The results are presented under assumed technology improvements for the years of 2020 (a), 2025 (b), 2030 (c), and 2050 (d), respectively. The TCO-advantageous technology for a given application domain is indicated by the color of the area. Color shading indicates the relative advantage of the leading technology against the second-best technology. A darker color indicates a stronger advantage and vice versa. In each subfigure, typical applications of VTOLs, including air taxis, air vans, airport shuttles, and private flying cars, are indicated using circled numbers. BE: Battery Electric; FC: Fuel Cell; ICE: Internal Combustion Engine; FE: Fossil Energy-based; RE: Renewable Energy-based.


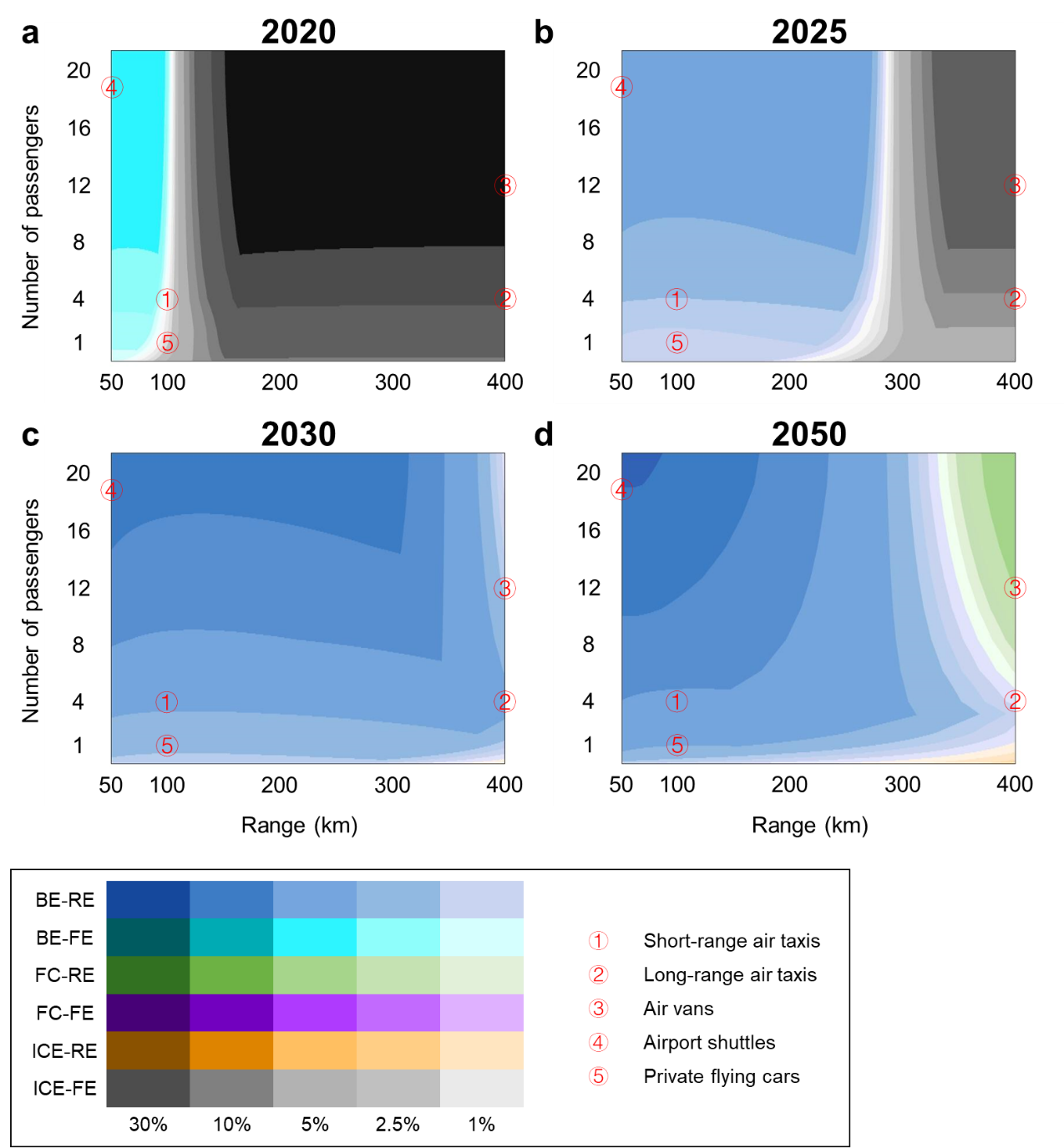


**Fig. S66.** Sensitivity analysis on TCO-advantageous technologies (RE energy price: -20%). The results are presented under assumed technology improvements for the years of 2020 (a), 2025 (b), 2030 (c), and 2050 (d), respectively. The TCO-advantageous technology for a given application domain is indicated by the color of the area. Color shading indicates the relative advantage of the leading technology against the second-best technology. A darker color indicates a stronger advantage and vice versa. In each subfigure, typical applications of VTOLs, including air taxis, air vans, airport shuttles, and private flying cars, are indicated using circled numbers. BE: Battery Electric; FC: Fuel Cell; ICE: Internal Combustion Engine; FE: Fossil Energy-based; RE: Renewable Energy-based.

**
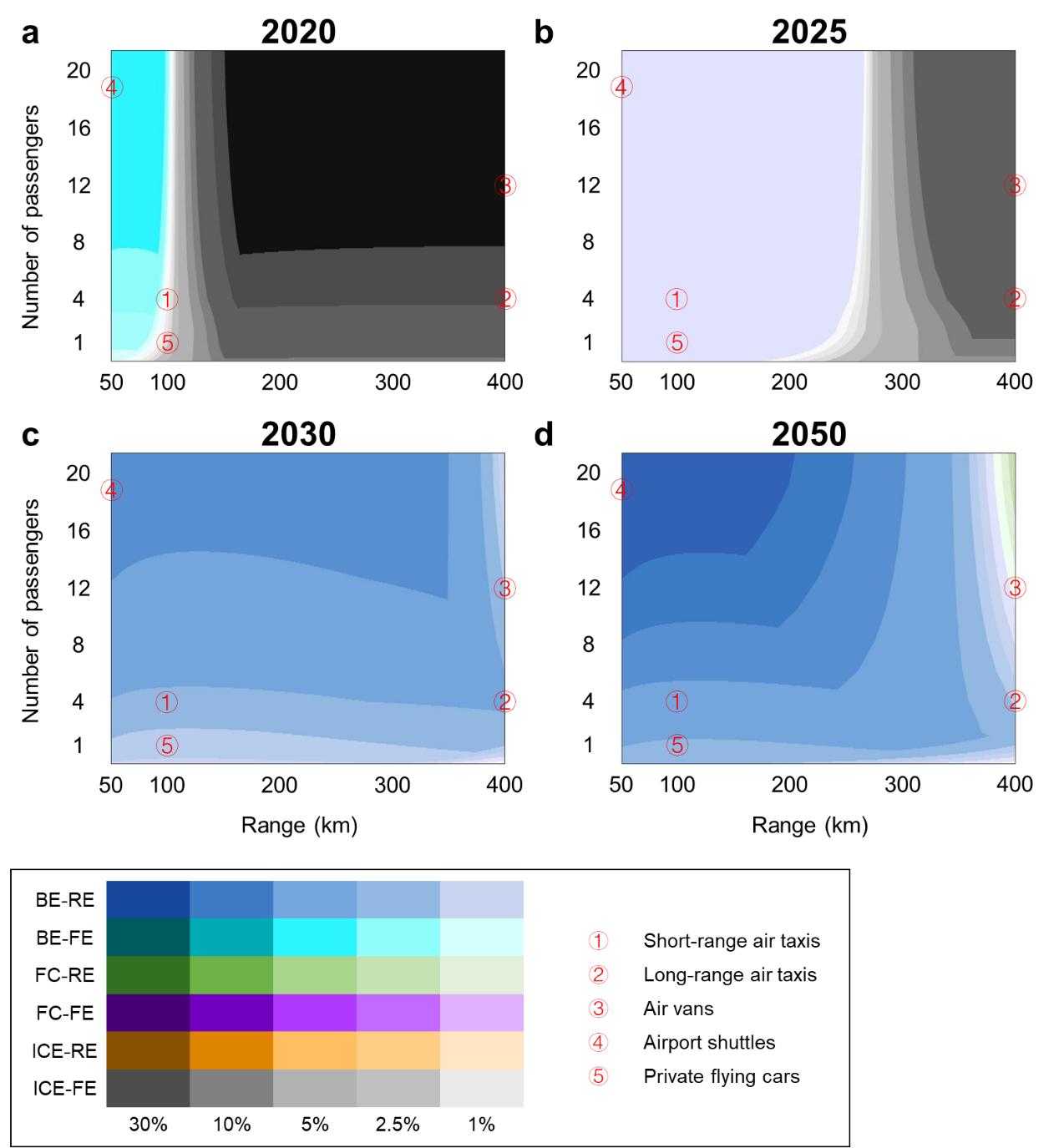
**

**Fig. S67.** Sensitivity analysis on TCO-advantageous technologies (RE energy price: +20%). The results are presented under assumed technology improvements for the years of 2020 (a), 2025 (b), 2030 (c), and 2050 (d), respectively. The TCO-advantageous technology for a given application domain is indicated by the color of the area. Color shading indicates the relative advantage of the leading technology against the second-best technology. A darker color indicates a stronger advantage and vice versa. In each subfigure, typical applications of VTOLs, including air taxis, air vans, airport shuttles, and private flying cars, are indicated using circled numbers. BE: Battery Electric; FC: Fuel Cell; ICE: Internal Combustion Engine; FE: Fossil Energy-based; RE: Renewable Energy-based.

**
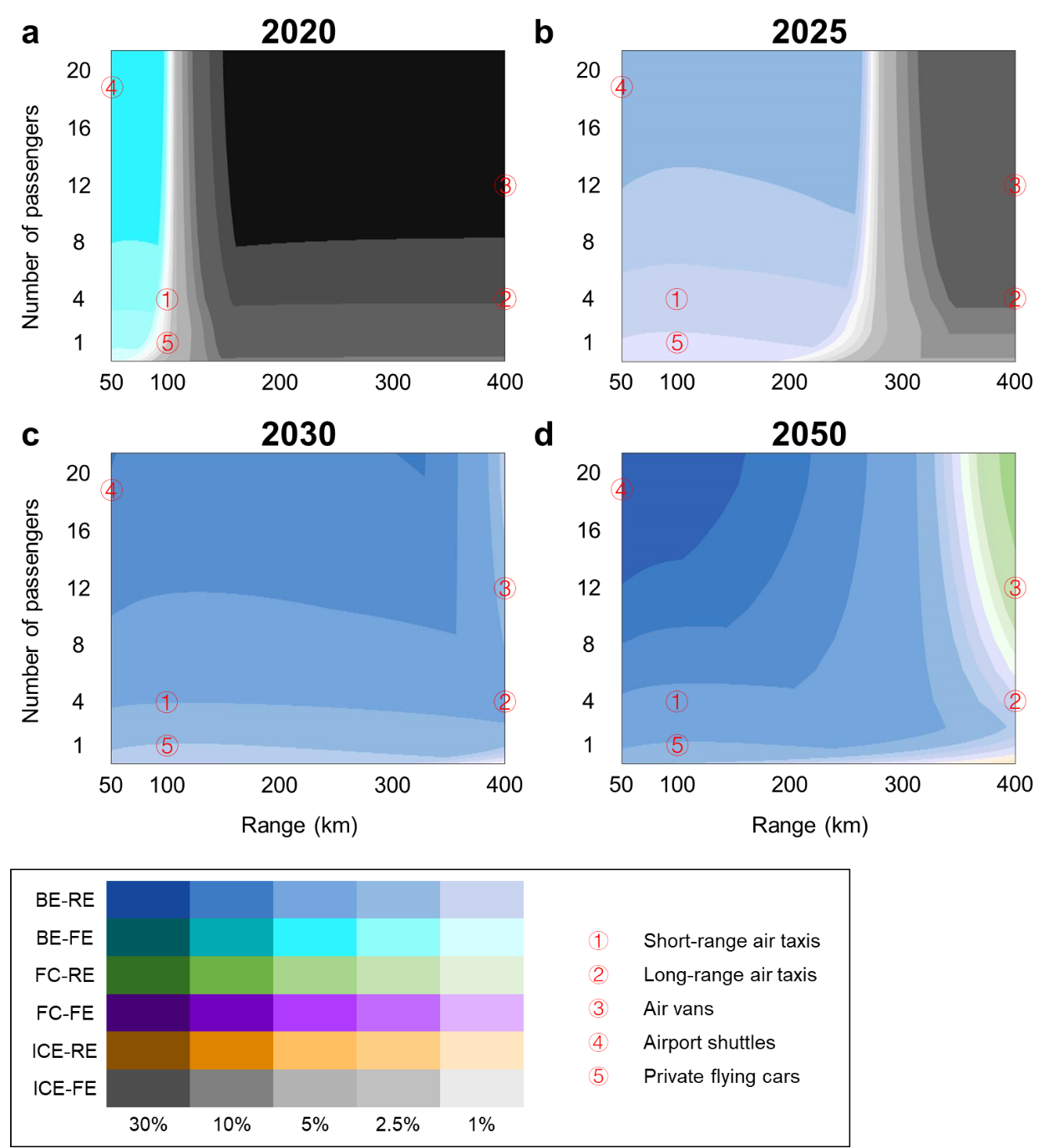
**

**Fig. S68.** Sensitivity analysis on TCO-advantageous technologies (VTOL life: 10 years). The results are presented under assumed technology improvements for the years of 2020 (a), 2025 (b), 2030 (c), and 2050 (d), respectively. The TCO-advantageous technology for a given application domain is indicated by the color of the area. Color shading indicates the relative advantage of the leading technology against the second-best technology. A darker color indicates a stronger advantage and vice versa. In each subfigure, typical applications of VTOLs, including air taxis, air vans, airport shuttles, and private flying cars, are indicated using circled numbers. BE: Battery Electric; FC: Fuel Cell; ICE: Internal Combustion Engine; FE: Fossil Energy-based; RE: Renewable Energy-based.

**
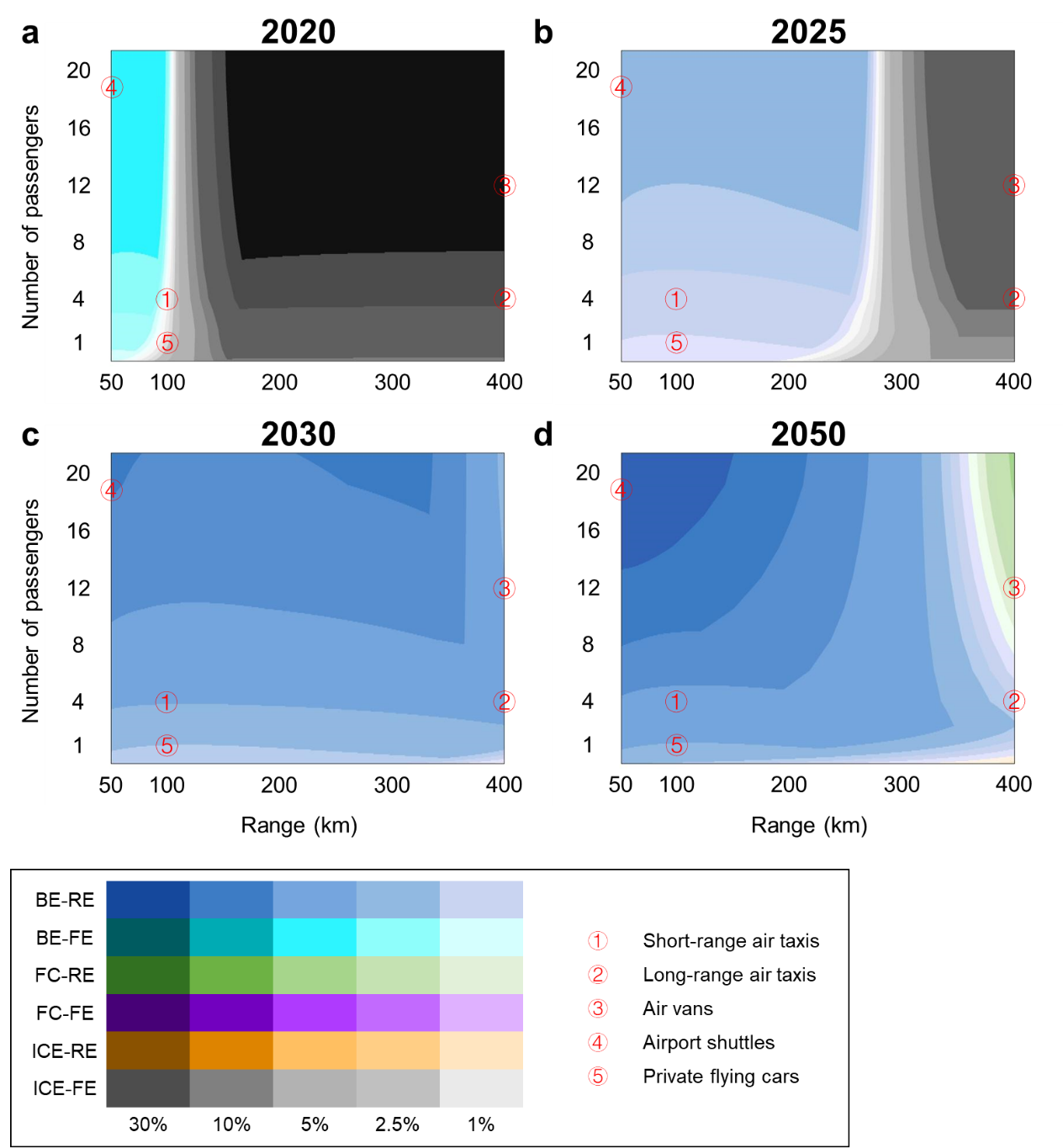
**

**Fig. S69.** Sensitivity analysis on TCO-advantageous technologies (VTOL life: 16 years). The results are presented under assumed technology improvements for the years of 2020 (a), 2025 (b), 2030 (c), and 2050 (d), respectively. The TCO-advantageous technology for a given application domain is indicated by the color of the area. Color shading indicates the relative advantage of the leading technology against the second-best technology. A darker color indicates a stronger advantage and vice versa. In each subfigure, typical applications of VTOLs, including air taxis, air vans, airport shuttles, and private flying cars, are indicated using circled numbers. BE: Battery Electric; FC: Fuel Cell; ICE: Internal Combustion Engine; FE: Fossil Energy-based; RE: Renewable Energy-based.

**
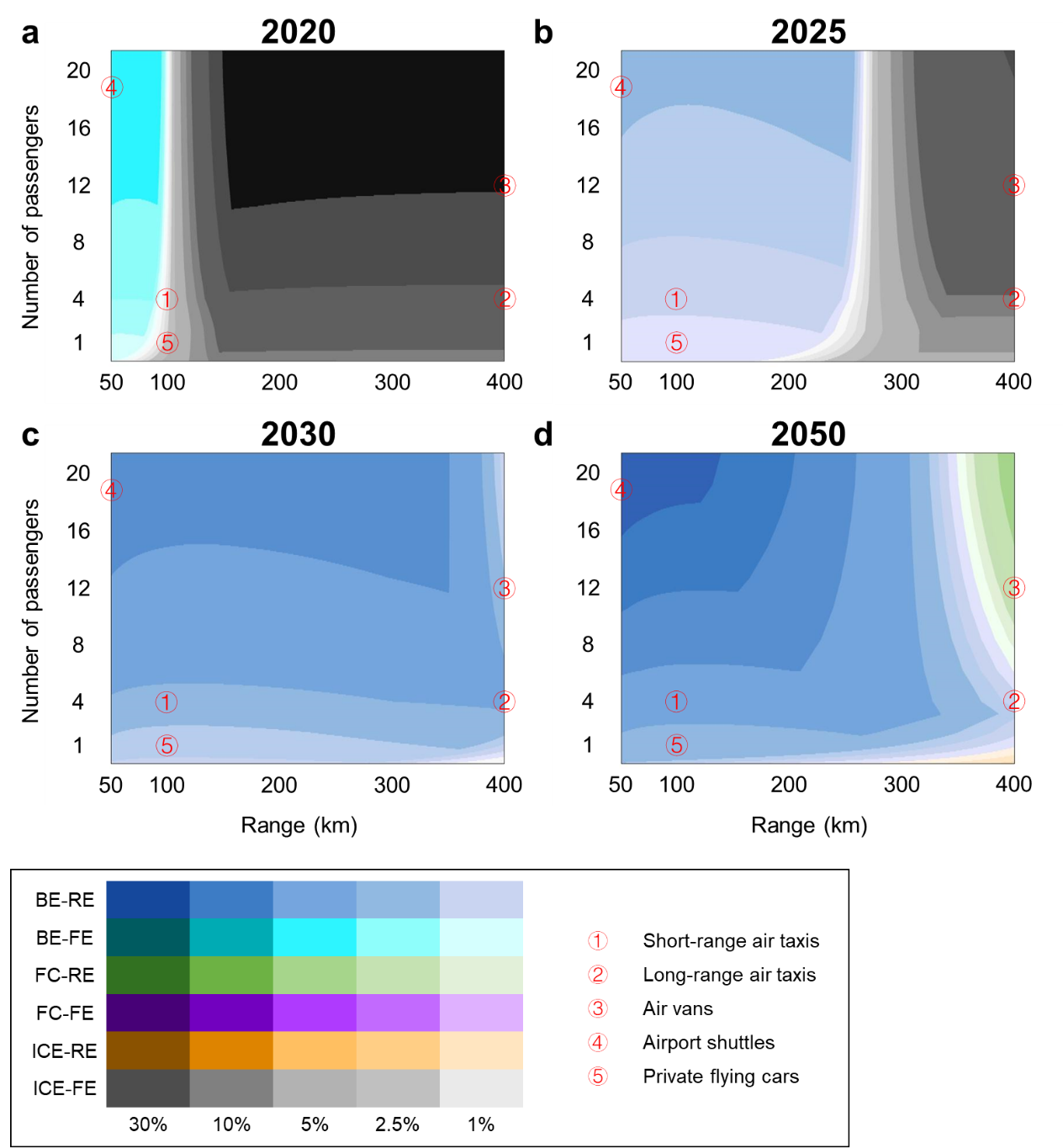
**

**Fig. S70.** Sensitivity analysis on TCO-advantageous technologies (Yearly operation time: -20%). The results are presented under assumed technology improvements for the years of 2020 (a), 2025 (b), 2030 (c), and 2050 (d), respectively. The TCO-advantageous technology for a given application domain is indicated by the color of the area. Color shading indicates the relative advantage of the leading technology against the second-best technology. A darker color indicates a stronger advantage and vice versa. In each subfigure, typical applications of VTOLs, including air taxis, air vans, airport shuttles, and private flying cars, are indicated using circled numbers. BE: Battery Electric; FC: Fuel Cell; ICE: Internal Combustion Engine; FE: Fossil Energy-based; RE: Renewable Energy-based.


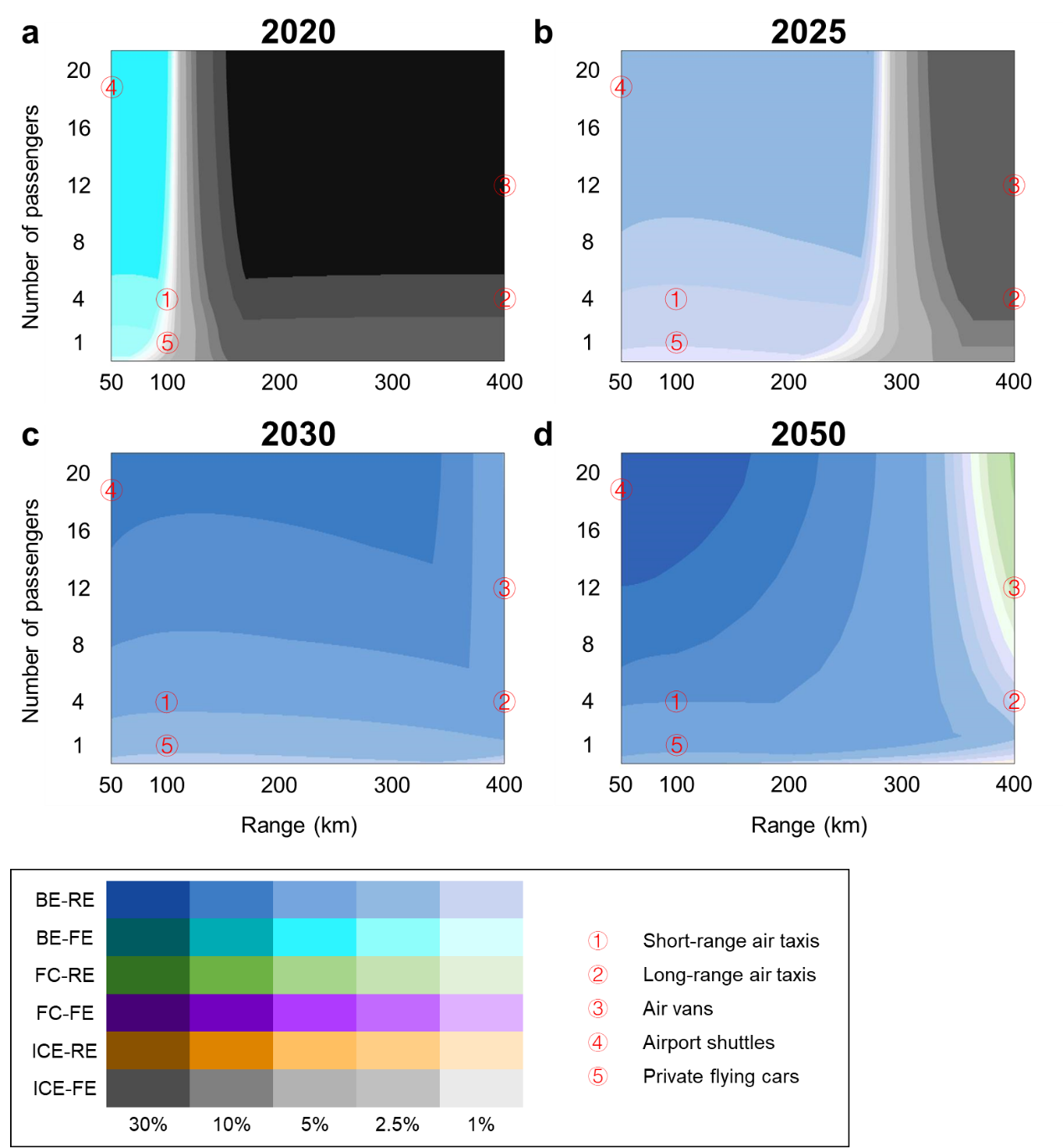


**Fig. S71.** Sensitivity analysis on TCO-advantageous technologies (Yearly operation time: +20%). The results are presented under assumed technology improvements for the years of 2020 (a), 2025 (b), 2030 (c), and 2050 (d), respectively. The TCO-advantageous technology for a given application domain is indicated by the color of the area. Color shading indicates the relative advantage of the leading technology against the second-best technology. A darker color indicates a stronger advantage and vice versa. In each subfigure, typical applications of VTOLs, including air taxis, air vans, airport shuttles, and private flying cars, are indicated using circled numbers. BE: Battery Electric; FC: Fuel Cell; ICE: Internal Combustion Engine; FE: Fossil Energy-based; RE: Renewable Energy-based.


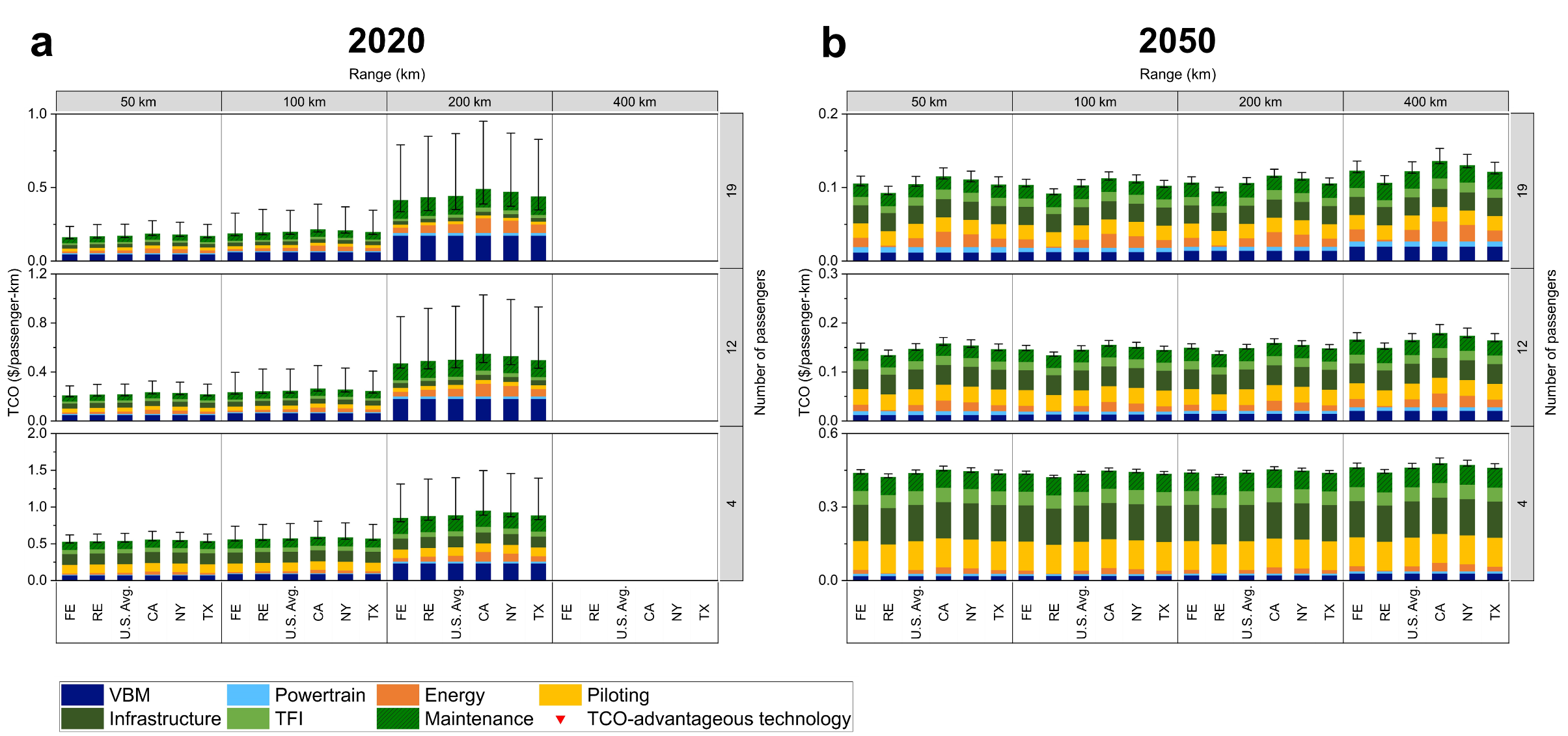


**Fig. S72.** TCO breakdown of BE technology under different electricity rate assumptions. The two subfigures are presented under baseline technology improvements in 2020 (a) and 2050 (b). Each subfigure is divided into twelve application domains distinguished by the number of passengers (vertical dimension) and the range (horizontal dimension). For each application domain, The TCO estimates of BE technology with six electricity rate scenarios (*i.e.*, FE, RE, U.S. grid average [U.S. Avg.], California grid [CA], New York grid [NY], and Texas grid [TX]) are shown, with different colors representing different cost components.(1, 2) Additional charges, such as fixed-charges and demand-charges, are also considered.(3) The absence of estimates for some circumstances indicates technological infeasibility. Salvage value is not shown due to its marginal influence on TCO. BE: Battery Electric; FC: Fuel Cell; ICE: Internal Combustion Engine; FE: Fossil Energy-based; RE: Renewable Energy-based; TCO: Total Cost of Ownership; VBM: VTOL Body Manufacturing; TFI: Taxes, Fees, and Insurance; U.S. Avg.: the United States average; CA: California; NY: New York; TX: Texas.


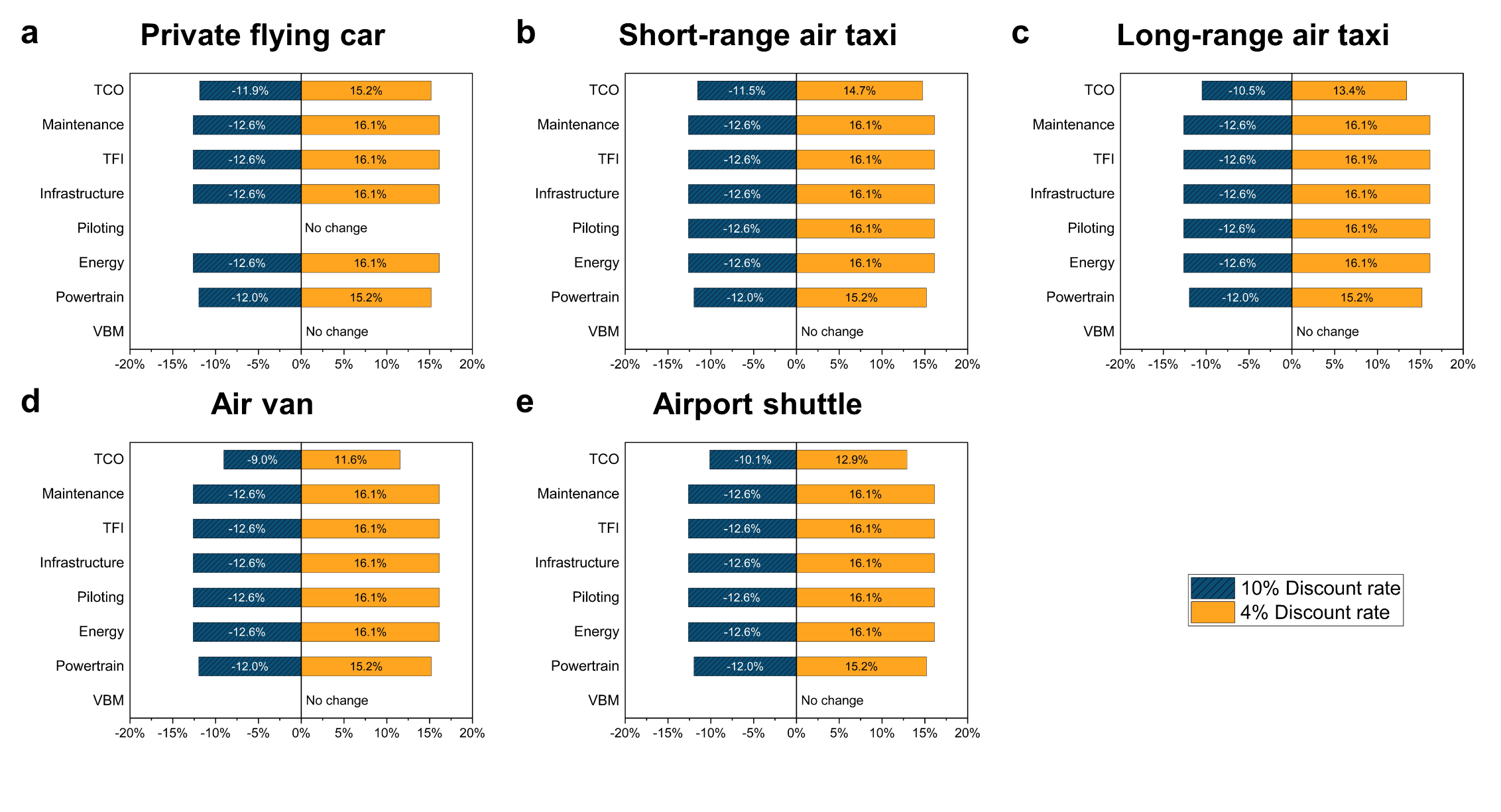


**Fig. S73.** Sensitivity analysis on cost results of BE-RE VTOLs with different discount rates in 2025 scenario. The subfigures represent results under different applications: private flying cars (a); short-range air taxis (b); long-range air taxis (c); air vans (d); and airport shuttles (e). The cost changes with high discount rate (10%) and low discount rate (4%) are shown, with 7% being the baseline discount rate. TCO: Total Cost of Ownership; VBM: VTOL Body Manufacturing; TFI: Taxes, Fees, and Insurance.


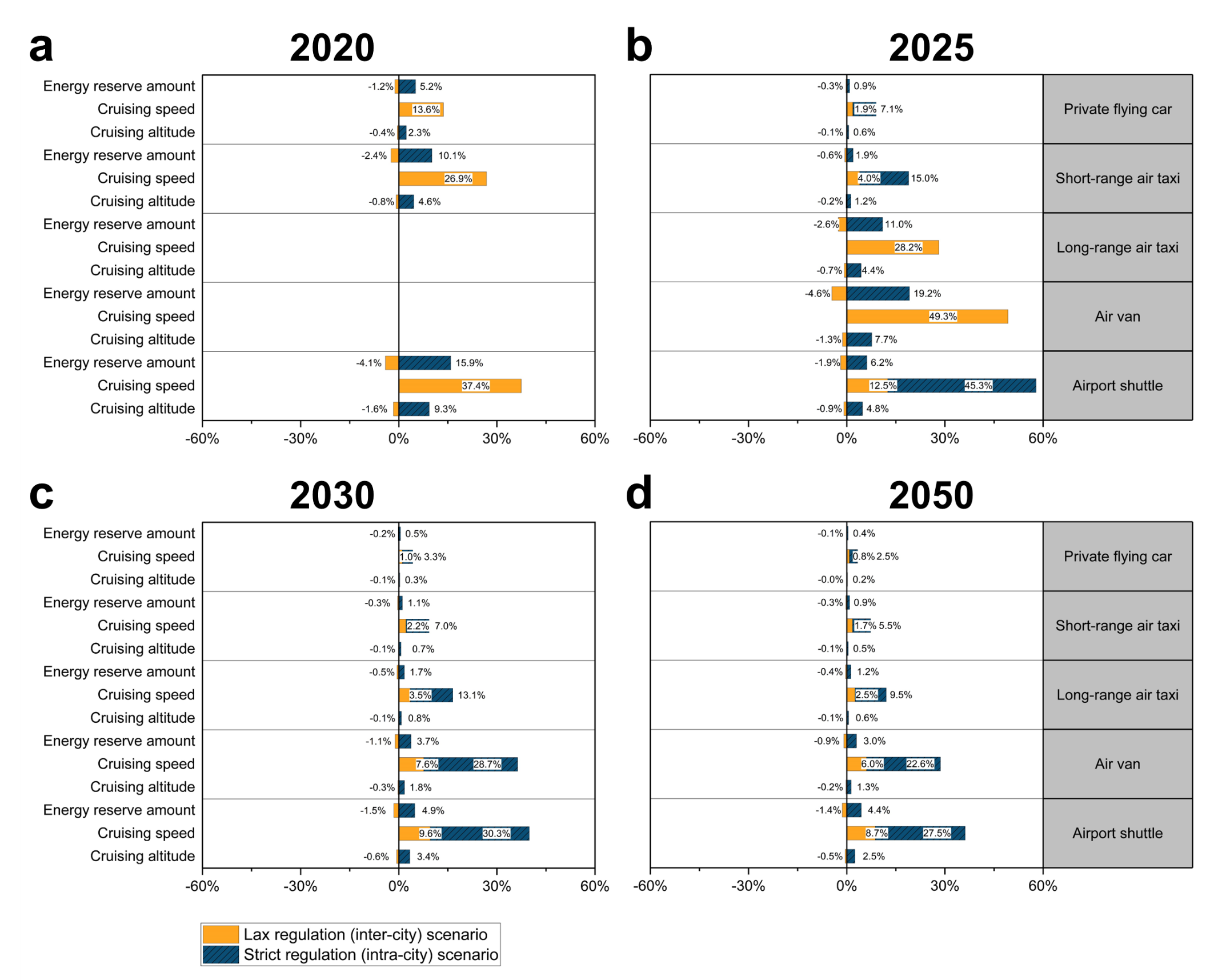


**Fig. S74.** Sensitivity analysis on the TCO of BE-RE VTOLs to the regulations. The subfigures present the sensitivity of the TCO estimates to changes in VTOL technical parameters (*i.e.*, cruising speed, cruising altitude, energy reserve amount, and power density), which is highly related to regulations, in 2020 (a), 2025 (b), 2030 (c), and 2050 (d), respectively. In each subfigure, the TCO changes of five typical VTOL applications (*i.e.*, private flying car, short-range air taxi, long-range air taxi, air van, and airport shuttle) under lax regulation (inter-city) and strict regulation (intra-city) scenarios are shown. The absence of TCO changes for some circumstances indicates technological infeasibility. BE: Battery Electric; RE: Renewable Energy-based; TCO: Total Cost of Ownership; VTOL: Vertical Takeoff and Landing aircraft.


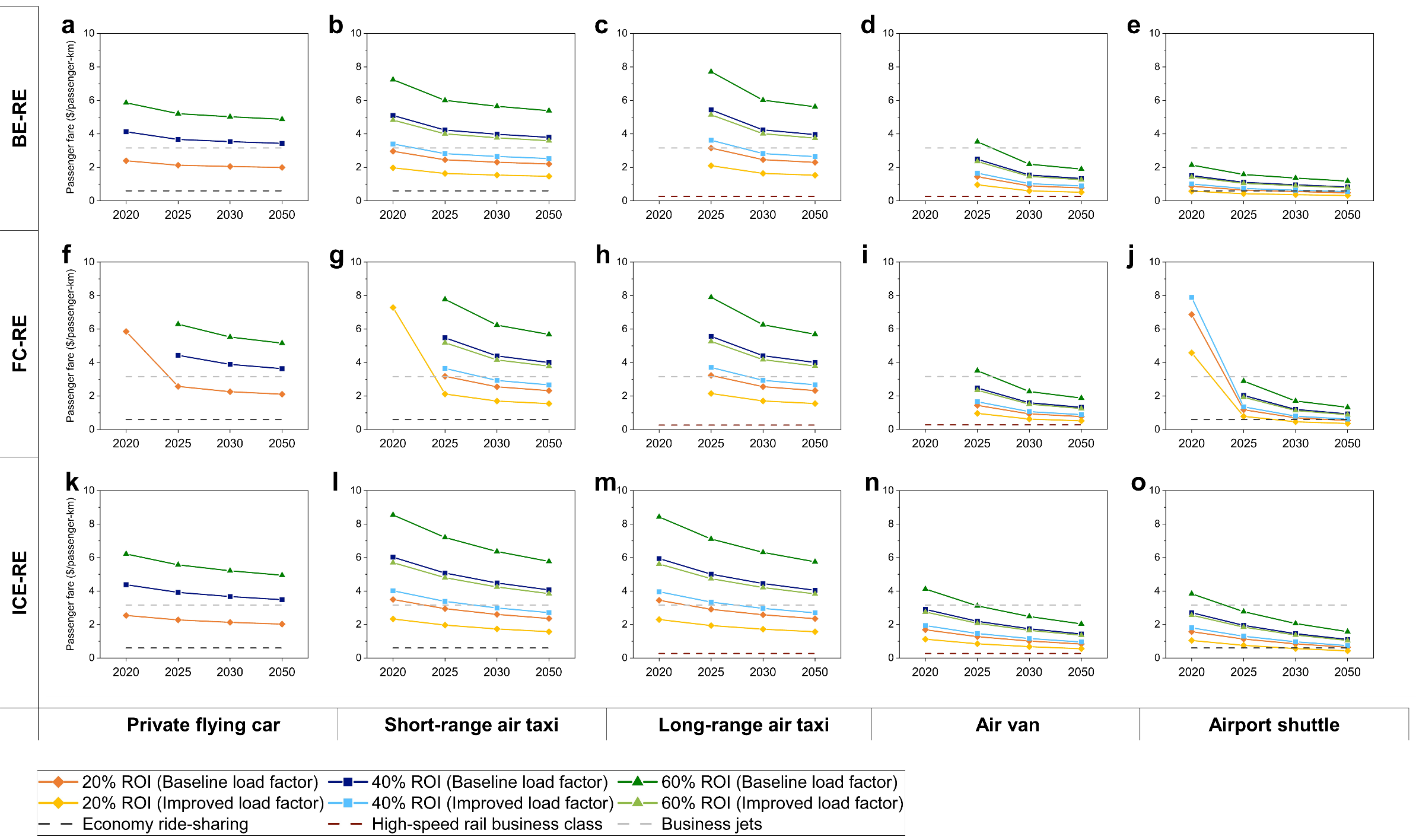


**Fig. S75.** Required passenger fares for achieving target ROIs under RE technologies. The subfigures represent results under different applications (horizontal dimension) and technologies (vertical dimension): BE-RE for private flying cars (a), short-range air taxis (b), long-range air taxis (c), air vans (d), and airport shuttles (e); FC-RE for private flying cars (f), short-range air taxis (g), long-range air taxis (h), air vans (i), and airport shuttles (j); ICE-RE for private flying cars (k), short-range air taxis (l), long-range air taxis (m), air vans (n), and airport shuttles (o). The required fares are provided for three ROIs (20% as a benchmark, 40%, and 60%) and two load factor cases (baseline vs. improved). For some cases, where required fares are too high to be realistic, the fares are not plotted. Average passenger fares for economy ride-sharing, high-speed rail business class, and business jets are provided as benchmarks to be compared with the results. BE: Battery Electric; FC: Fuel Cell; ICE: Internal Combustion Engine; RE: Renewable Energy-based; ROI: Return on Investment.


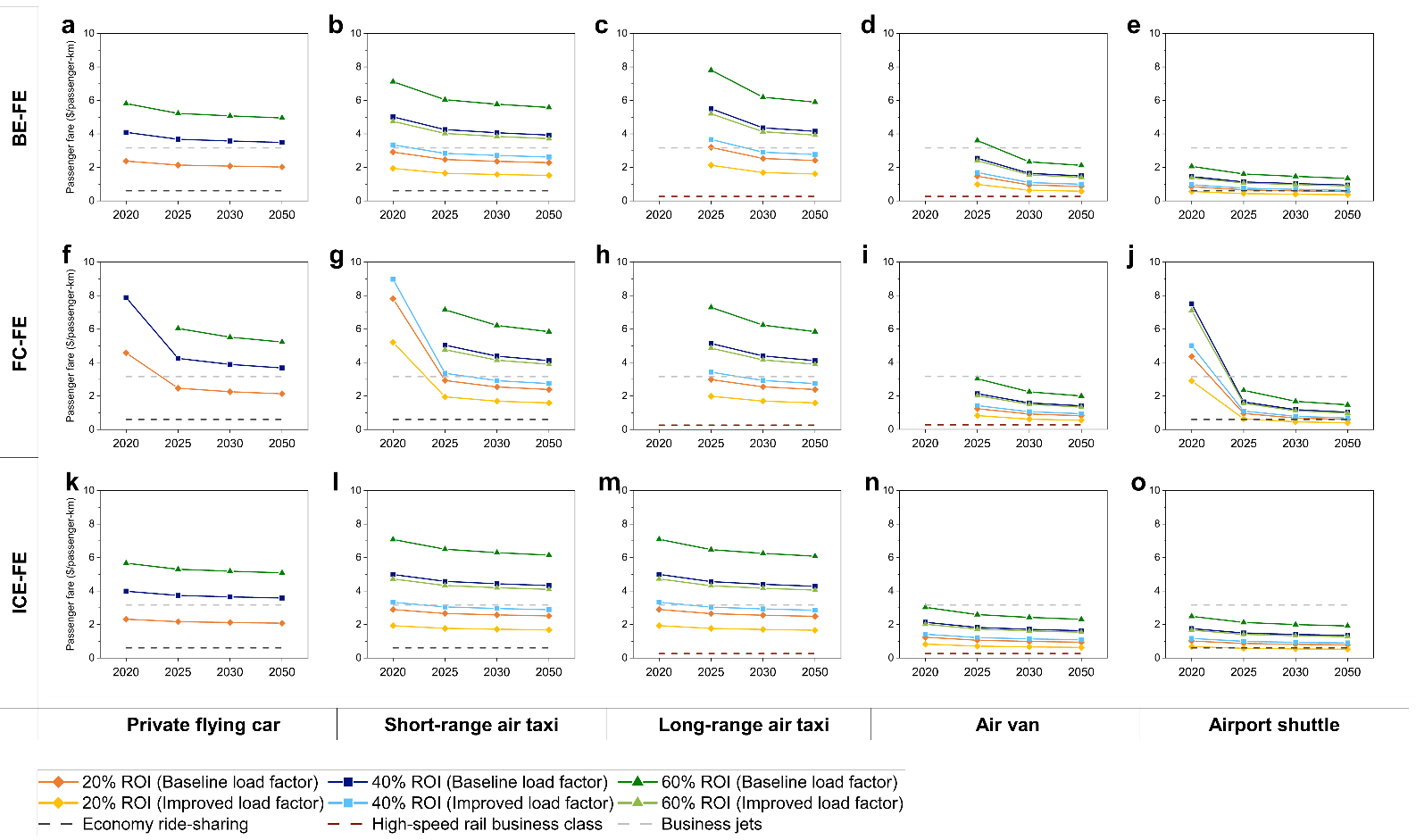


**Fig. S76.** Required passenger fares for achieving target ROIs under FE technologies. The subfigures represent results under different applications (horizontal dimension) and technologies (vertical dimension): BE-FE for private flying cars (a), short-range air taxis (b), long-range air taxis (c), air vans (d), and airport shuttles (e); FC-FE for private flying cars (f), short-range air taxis (g), long-range air taxis (h), air vans (i), and airport shuttles (j); ICE-FE for private flying cars (k), short-range air taxis (l), long-range air taxis (m), air vans (n), and airport shuttles (o). The required fares are provided for three ROIs (20% as a benchmark, 40%, and 60%) and two load factor cases (baseline vs. improved). For some cases, where required fares are too high to be realistic, the fares are not plotted. Average passenger fares for economy ride-sharing, high-speed rail business class, and business jets are provided as benchmarks to be compared with the results. BE: Battery Electric; FC: Fuel Cell; ICE: Internal Combustion Engine; FE: Fossil Energy-based; ROI: Return on Investment.

**Supplementary Tables**

**Table S1.** Key TCO modeling assumptions for VTOLs

| **Segment 1. Key assumptions for VTOL component sizing** | | | | | | | | | | | |
| --- | --- | --- | --- | --- | --- | --- | --- | --- | --- | --- | --- |
| **Parameter** | | **Notation** | | **Unit** | | | **Value** | | | **References** | |
| Disk loading | | 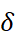 | | N/m^2^ | | | 450 | | | (4-6) | |
| Air density | | 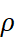 | | kg/m^3^ | | | 1.29 | | |  | |
| Cruising altitude | | 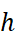 | | m | | | 305 | | | (7, 8) | |
| Cruising speed | | 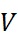 | | km/h | | | 150 | | | (9) | |
| Energy reserve | | 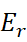 | | min cruising-range | | | 15 | | | (7) | |
| Angle of attack | | 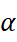 | | degree | | | 20 | | | (7) | |
| Lift-to-drag ratio while cruising | | 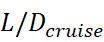 | | - | | | 14 | | | (5, 8) | |
| Lift-to-drag ratio while climbing | | 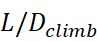 | | - | | | 10.5 | | | (5) | |
| Rate of climb | | 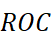 | | m/s | | | 5 | | | (7) | |
| Rate of descent | | 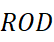 | | m/s | | | 5 | | | (7) | |
| Structural mass fraction considering takeoff mass | | 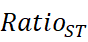 | | - | | | 44% | | | (5, 8) | |
| Ratio of structural mass to battery mass | | 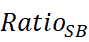 | | - | | | 40% | | | (10) | |
| Number of passengers | | 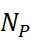 | | - | | | up to 19 | | |  | |
| Average passenger mass | | - | | kg | | | 75 | | |  | |
| Hover-time | | 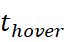 | | s | | | 30 | | | (7) | |
| **Segment 2. Key assumptions for VTOL operation** | | | | | | | | | | | |
| **Parameters** | | **Notation** | | **Unit** | | | **Value** | | | **References** | |
| VTOL life | | 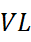 | | h | | | 26,000 | | | (11) | |
| Yearly operation time | | 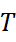 | | h | | | 2,000 | | | (11) | |
| Battery life | | - | | cycle | | | 2,000 | | | (11) | |
| Trip range per mission | | 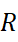 | | km | | | up to 400 | | |  | |
| Dead-end ratio | | 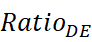 | | - | | | 20% | | | (11) | |
| Load factor | | 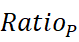 | | - | | | 50%-75% (100% for one-seater) | | | (12) | |
| Discount rate | | 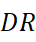 | | - | | | 7% | | | (13) | |
| **Segment 3. Key assumptions for VTOL powertrain components**  **(baseline scenario)** | | | | | | | | | | | |
| **Parameters** | | **Notation** | **Unit** | | **Value** | | | | | | **References** |
|  |  |  |  |  | **2020** | **2025** | | **2030** | **2050** | |  |
| Battery | Energy density | - | Wh/kg | | 200 | 400 | | 600 | 600 | | (14, 15) |
|  | Charging efficiency | 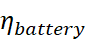 | - | | 90% | | | | | | (10) |
|  | Cost | 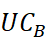 | $/kWh | | 100 | 120 | | 140 | 120 | | (15) |
| Fuel cell | Power density | - | kW/kg | | 0.75 | 1.0 | | 1.5 | 1.75 | | (16) |
|  | Energy efficiency | 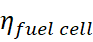 | - | | 45% | 55% | | 60% | 60% | | (16) |
|  | Cost |  | $/kW | | 40 | 30 | | 20 | 20 | | (17) |
| ICE | Power density | - | kW/kg | | 1.2 | | | | | | (10) |
|  | Energy efficiency |  | - | | 40% | 43% | | 45% | 45% | | * |
|  | Cost |  | $/kW | | 30 | 27.5 | | 25 | 25 | | (18) |
| Motor | Power density | - | kW/kg | | 4.0 | | | | | | (8, 9) |
|  | Energy efficiency |  | - | | 92% | | | | | | (8, 9) |
|  | Cost |  | $/kW | | 25 | | | | | | (19) |
| Transmission efficiency | |  | - | | 98% | | | | | | (8) |
| LH_2_ tank | Mass of LH_2_ in hydrogen tank | - | wt% | | 18% | | | | | | (16) |
|  | Cost |  | $/kg | | 40 | 30 | | 20 | 10 | | (16, 17) |
| VBM | Base |  | million $ | | 0.6 | 0.4 | | 0.3 | 0.2 | | (16) |
|  | Variable |  | $/kg | | 800 | 550 | | 400 | 270 | | (16) |
| Fuel lower heating value | Gasoline | - | MJ/kg | | 46.0 | | | | | |  |
|  | Hydrogen | - |  |  | 119.6 | | | | | |  |
| Energy price | Natural gas |  | $/MBtu | | 4.2 | 6.0 | | 7.7 | 8.3 | | (20) |
|  | Electricity (FE) |  | $/kWh | | 0.09 | 0.11 | | 0.12 | 0.13 | | (21) |
|  | Electricity  (RE) |  |  |  | 0.13 | 0.09 | | 0.05 | 0.02 | | (21, 22) |
|  | Hydrogen (FE) |  | $/kg | | 2.9 | 3.3 | | 3.6 | 3.7 | | (23) |
|  | Hydrogen (RE) |  |  |  | 9.6 | 7.0 | | 3.8 | 2.0 | | (23) |
|  | Gasoline |  | $/L | | 1.0 | 1.3 | | 1.6 | 1.8 | | (20, 24) |
|  | Sustainable aviation fuel |  |  |  | 3.6 | 2.7 | | 1.7 | 1.0 | | (25) |
| Vertiport | Construction | - | million $ | | 1.45 | | | | | | (11) |
|  | Operation | - | $/VTOL-yr | | 80,000 | | | | | | (11) |
| Battery swapping facility | Construction | - | million $ | | 0.6 | | | | | | (26) |
|  | Operation | - | $/yr | | 82,000 | | | | | | Calculated |
| Hydrogen refueling facility | Construction | - | million $ | | 2.5 | 2.0 | | 1.5 | 1.0 | | (27-29) |
|  | Operation | - | $/yr | | 160,000 | 150,000 | | 140,000 | 130,000 | | Calculated |
| Liquid refueling facility | Construction | - | million $ | | 0.5 | | | | | | (30) |
|  | Operation | - | $/yr | | 80,000 | | | | | | Calculated |
| Maintenance | Variable |  | $/flight hour | | 20 | | | | | | (16) |
|  | Fixed | - | $/yr | | 7.5% of VBM cost | | | | | | (11, 16) |
| Piloting | |  | $/yr | | 75000 | | | | | | (11) |
| **Segment 4. Key assumptions for VTOL powertrain components**  **(alternative scenarios for 2050)** | | | | | | | | | | | |
| **Parameters** | | **Notation** | **Unit** | | **Value** | | | | | | **References** |
|  |  |  |  |  | **Lagged** | | | **Advanced** | | |  |
| Battery | Energy density | - | Wh/kg | | 400 | | | 800 | | |  |
|  | Cost |  | $/kWh | | 100 | | | 160 | | |  |
| Fuel cell | Power density | - | kW/kg | | 1.0 | | | 2.0 | | |  |
|  | Energy efficiency |  | - | | 55% | | | 65% | | |  |

* The ICE efficiency is assumed considering that rotate speeds of the ICE and rotors are well matched, which enables ICE to achieve a higher energy efficiency.

**Table S2. TCO results for private flying cars (1-seater, 100-km-range) under the baseline scenario**

| Technology | TCO breakdown ($/passenger-km) | | | | | | | | Total |
| --- | --- | --- | --- | --- | --- | --- | --- | --- | --- |
|  | VBM | Powertrain | Energy | Piloting | Infrastructure | TFI | Maintenance | Salvage value |  |
| 2020 | | | | | | | | | |
| BE-FE | 0.0505 | 0.0044 | 0.0072 | 0 | 0.2218 | 0.0458 | 0.1256 | -0.0011 | 0.4542 |
| BE-RE | 0.0505 | 0.0044 | 0.0103 | 0 | 0.2218 | 0.0464 | 0.1256 | -0.0011 | 0.4580 |
| FC-FE | 0.2155 | 0.0074 | 0.0883 | 0 | 0.2620 | 0.0701 | 0.2362 | -0.0048 | 0.8747 |
| FC-RE | 0.2155 | 0.0074 | 0.2922 | 0 | 0.2620 | 0.1108 | 0.2362 | -0.0048 | 1.1193 |
| ICE-FE | 0.0446 | 0.0007 | 0.0140 | 0 | 0.2167 | 0.0462 | 0.1216 | -0.0010 | 0.4428 |
| ICE-RE | 0.0446 | 0.0007 | 0.0498 | 0 | 0.2167 | 0.0533 | 0.1216 | -0.0010 | 0.4858 |
| 2025 | | | | | | | | | |
| BE-FE | 0.0244 | 0.0035 | 0.0057 | 0 | 0.2218 | 0.0455 | 0.1081 | -0.0005 | 0.4086 |
| BE-RE | 0.0244 | 0.0035 | 0.0047 | 0 | 0.2218 | 0.0453 | 0.1081 | -0.0005 | 0.4073 |
| FC-FE | 0.0348 | 0.0008 | 0.0149 | 0 | 0.2535 | 0.0537 | 0.1151 | -0.0008 | 0.4719 |
| FC-RE | 0.0348 | 0.0008 | 0.0315 | 0 | 0.2535 | 0.0570 | 0.1151 | -0.0008 | 0.4919 |
| ICE-FE | 0.0264 | 0.0006 | 0.0153 | 0 | 0.2167 | 0.0464 | 0.1094 | -0.0006 | 0.4143 |
| ICE-RE | 0.0264 | 0.0006 | 0.0325 | 0 | 0.2167 | 0.0498 | 0.1094 | -0.0006 | 0.4349 |
| 2030 | | | | | | | | | |
| BE-FE | 0.0172 | 0.0037 | 0.0056 | 0 | 0.2218 | 0.0455 | 0.1033 | -0.0004 | 0.3968 |
| BE-RE | 0.0172 | 0.0037 | 0.0023 | 0 | 0.2218 | 0.0448 | 0.1033 | -0.0004 | 0.3928 |
| FC-FE | 0.0201 | 0.0003 | 0.0100 | 0 | 0.2450 | 0.0510 | 0.1052 | -0.0004 | 0.4312 |
| FC-RE | 0.0201 | 0.0003 | 0.0105 | 0 | 0.2450 | 0.0511 | 0.1052 | -0.0004 | 0.4319 |
| ICE-FE | 0.0194 | 0.0005 | 0.0174 | 0 | 0.2167 | 0.0468 | 0.1047 | -0.0004 | 0.4051 |
| ICE-RE | 0.0194 | 0.0005 | 0.0191 | 0 | 0.2167 | 0.0472 | 0.1047 | -0.0004 | 0.4071 |
| 2050 | | | | | | | | | |
| BE-FE | 0.0115 | 0.0031 | 0.0061 | 0 | 0.2218 | 0.0456 | 0.0994 | -0.0003 | 0.3873 |
| BE-RE | 0.0115 | 0.0031 | 0.0009 | 0 | 0.2218 | 0.0446 | 0.0994 | -0.0003 | 0.3811 |
| FC-FE | 0.0129 | 0.0003 | 0.0096 | 0 | 0.2365 | 0.0492 | 0.1004 | -0.0003 | 0.4086 |
| FC-RE | 0.0129 | 0.0003 | 0.0052 | 0 | 0.2365 | 0.0483 | 0.1004 | -0.0003 | 0.4033 |
| ICE-FE | 0.0129 | 0.0005 | 0.0199 | 0 | 0.2167 | 0.0473 | 0.1004 | -0.0003 | 0.3975 |
| ICE-RE | 0.0129 | 0.0005 | 0.0108 | 0 | 0.2167 | 0.0455 | 0.1004 | -0.0003 | 0.3865 |

**Table S3.** TCO results for short-range air taxis (4-seater, 100-km-range) under the baseline scenario

| Technology | TCO breakdown ($/passenger-km) | | | | | | | | Total |
| --- | --- | --- | --- | --- | --- | --- | --- | --- | --- |
|  | VBM | Powertrain | Energy | Piloting | Infrastructure | TFI | Maintenance | Salvage value |  |
| 2020 | | | | | | | | | |
| BE-FE | 0.0880 | 0.0108 | 0.0174 | 0.1178 | 0.1479 | 0.0566 | 0.1201 | -0.0020 | 0.5567 |
| BE-RE | 0.0880 | 0.0108 | 0.0251 | 0.1178 | 0.1479 | 0.0582 | 0.1201 | -0.0020 | 0.5659 |
| FC-FE | 0.4890 | 0.0180 | 0.2146 | 0.1178 | 0.1747 | 0.1014 | 0.3891 | -0.0109 | 1.4938 |
| FC-RE | 0.4890 | 0.0180 | 0.7103 | 0.1178 | 0.1747 | 0.2006 | 0.3891 | -0.0109 | 2.0887 |
| ICE-FE | 0.0793 | 0.0019 | 0.0374 | 0.1178 | 0.1445 | 0.0599 | 0.1143 | -0.0018 | 0.5534 |
| ICE-RE | 0.0793 | 0.0019 | 0.1329 | 0.1178 | 0.1445 | 0.0790 | 0.1143 | -0.0018 | 0.6680 |
| 2025 | | | | | | | | | |
| BE-FE | 0.0407 | 0.0085 | 0.0140 | 0.1178 | 0.1479 | 0.0559 | 0.0885 | -0.0009 | 0.4724 |
| BE-RE | 0.0407 | 0.0085 | 0.0114 | 0.1178 | 0.1479 | 0.0554 | 0.0885 | -0.0009 | 0.4694 |
| FC-FE | 0.0660 | 0.0020 | 0.0361 | 0.1178 | 0.1690 | 0.0646 | 0.1054 | -0.0015 | 0.5594 |
| FC-RE | 0.0660 | 0.0020 | 0.0766 | 0.1178 | 0.1690 | 0.0727 | 0.1054 | -0.0015 | 0.6080 |
| ICE-FE | 0.0494 | 0.0015 | 0.0409 | 0.1178 | 0.1445 | 0.0606 | 0.0943 | -0.0011 | 0.5079 |
| ICE-RE | 0.0494 | 0.0015 | 0.0866 | 0.1178 | 0.1445 | 0.0698 | 0.0943 | -0.0011 | 0.5628 |
| 2030 | | | | | | | | | |
| BE-FE | 0.0280 | 0.0089 | 0.0137 | 0.1178 | 0.1479 | 0.0559 | 0.0799 | -0.0006 | 0.4514 |
| BE-RE | 0.0280 | 0.0089 | 0.0057 | 0.1178 | 0.1479 | 0.0543 | 0.0799 | -0.0006 | 0.4418 |
| FC-FE | 0.0350 | 0.0008 | 0.0243 | 0.1178 | 0.1633 | 0.0611 | 0.0846 | -0.0008 | 0.4862 |
| FC-RE | 0.0350 | 0.0008 | 0.0256 | 0.1178 | 0.1633 | 0.0614 | 0.0846 | -0.0008 | 0.4878 |
| ICE-FE | 0.0359 | 0.0012 | 0.0465 | 0.1178 | 0.1445 | 0.0618 | 0.0852 | -0.0008 | 0.4920 |
| ICE-RE | 0.0359 | 0.0012 | 0.0509 | 0.1178 | 0.1445 | 0.0626 | 0.0852 | -0.0008 | 0.4973 |
| 2050 | | | | | | | | | |
| BE-FE | 0.0186 | 0.0076 | 0.0148 | 0.1178 | 0.1479 | 0.0561 | 0.0737 | -0.0004 | 0.4361 |
| BE-RE | 0.0186 | 0.0076 | 0.0023 | 0.1178 | 0.1479 | 0.0536 | 0.0737 | -0.0004 | 0.4211 |
| FC-FE | 0.0221 | 0.0008 | 0.0233 | 0.1178 | 0.1577 | 0.0598 | 0.0759 | -0.0005 | 0.4568 |
| FC-RE | 0.0221 | 0.0008 | 0.0126 | 0.1178 | 0.1577 | 0.0576 | 0.0759 | -0.0005 | 0.4439 |
| ICE-FE | 0.0239 | 0.0012 | 0.0531 | 0.1178 | 0.1445 | 0.0631 | 0.0772 | -0.0005 | 0.4803 |
| ICE-RE | 0.0239 | 0.0012 | 0.0288 | 0.1178 | 0.1445 | 0.0582 | 0.0772 | -0.0005 | 0.4511 |

**Table S4.** TCO results for long-range air taxis (4-seater, 400-km-range) under the baseline scenario

| Technology | TCO breakdown ($/passenger-km) | | | | | | | | Total |
| --- | --- | --- | --- | --- | --- | --- | --- | --- | --- |
|  | VBM | Powertrain | Energy | Piloting | Infrastructure | TFI | Maintenance | Salvage value |  |
| 2020 | | | | | | | | | |
| BE-FE* |  |  |  |  |  |  |  |  |  |
| BE-RE* |  |  |  |  |  |  |  |  |  |
| FC-FE | 1.6479 | 0.0665 | 0.6438 | 0.1178 | 0.1747 | 0.1873 | 1.1664 | -0.0366 | 3.9678 |
| FC-RE | 1.6479 | 0.0665 | 2.1313 | 0.1178 | 0.1747 | 0.4848 | 1.1664 | -0.0366 | 5.7528 |
| ICE-FE | 0.0822 | 0.0020 | 0.0341 | 0.1178 | 0.1445 | 0.0593 | 0.1163 | -0.0018 | 0.5542 |
| ICE-RE | 0.0822 | 0.0020 | 0.1211 | 0.1178 | 0.1445 | 0.0767 | 0.1163 | -0.0018 | 0.6587 |
| 2025 | | | | | | | | | |
| BE-FE | 0.1037 | 0.0177 | 0.0350 | 0.1178 | 0.1479 | 0.0601 | 0.1307 | -0.0023 | 0.6105 |
| BE-RE | 0.1037 | 0.0177 | 0.0286 | 0.1178 | 0.1479 | 0.0589 | 0.1307 | -0.0023 | 0.6029 |
| FC-FE | 0.0729 | 0.0024 | 0.0352 | 0.1178 | 0.1690 | 0.0644 | 0.1100 | -0.0016 | 0.5702 |
| FC-RE | 0.0729 | 0.0024 | 0.0747 | 0.1178 | 0.1690 | 0.0723 | 0.1100 | -0.0016 | 0.6176 |
| ICE-FE | 0.0510 | 0.0015 | 0.0371 | 0.1178 | 0.1445 | 0.0599 | 0.0954 | -0.0011 | 0.5060 |
| ICE-RE | 0.0510 | 0.0015 | 0.0785 | 0.1178 | 0.1445 | 0.0682 | 0.0954 | -0.0011 | 0.5557 |
| 2030 | | | | | | | | | |
| BE-FE | 0.0425 | 0.0106 | 0.0196 | 0.1178 | 0.1479 | 0.0571 | 0.0896 | -0.0009 | 0.4840 |
| BE-RE | 0.0425 | 0.0106 | 0.0082 | 0.1178 | 0.1479 | 0.0548 | 0.0896 | -0.0009 | 0.4703 |
| FC-FE | 0.0371 | 0.0009 | 0.0227 | 0.1178 | 0.1633 | 0.0608 | 0.0860 | -0.0008 | 0.4877 |
| FC-RE | 0.0371 | 0.0009 | 0.0239 | 0.1178 | 0.1633 | 0.0610 | 0.0860 | -0.0008 | 0.4893 |
| ICE-FE | 0.0369 | 0.0013 | 0.0420 | 0.1178 | 0.1445 | 0.0609 | 0.0859 | -0.0008 | 0.4884 |
| ICE-RE | 0.0369 | 0.0013 | 0.0460 | 0.1178 | 0.1445 | 0.0617 | 0.0859 | -0.0008 | 0.4932 |
| 2050 | | | | | | | | | |
| BE-FE | 0.0283 | 0.0091 | 0.0212 | 0.1178 | 0.1479 | 0.0574 | 0.0801 | -0.0006 | 0.4612 |
| BE-RE | 0.0283 | 0.0091 | 0.0033 | 0.1178 | 0.1479 | 0.0538 | 0.0801 | -0.0006 | 0.4396 |
| FC-FE | 0.0232 | 0.0008 | 0.0217 | 0.1178 | 0.1577 | 0.0594 | 0.0767 | -0.0005 | 0.4569 |
| FC-RE | 0.0232 | 0.0008 | 0.0117 | 0.1178 | 0.1577 | 0.0574 | 0.0767 | -0.0005 | 0.4449 |
| ICE-FE | 0.0246 | 0.0013 | 0.0480 | 0.1178 | 0.1445 | 0.0621 | 0.0777 | -0.0005 | 0.4753 |
| ICE-RE | 0.0246 | 0.0013 | 0.0260 | 0.1178 | 0.1445 | 0.0577 | 0.0777 | -0.0005 | 0.4489 |

*Technologically infeasible

**Table S5.** TCO results for air vans (12-seater, 400-km-range) under the baseline scenario

| Technology | TCO breakdown ($/passenger-km) | | | | | | | | Total |
| --- | --- | --- | --- | --- | --- | --- | --- | --- | --- |
|  | VBM | Powertrain | Energy | Piloting | Infrastructure | TFI | Maintenance | Salvage value |  |
| 2020 | | | | | | | | | |
| BE-FE* |  |  |  |  |  |  |  |  |  |
| BE-RE* |  |  |  |  |  |  |  |  |  |
| FC-FE | 1.3124 | 0.0532 | 0.5154 | 0.0321 | 0.0476 | 0.1190 | 0.8969 | -0.0291 | 2.9476 |
| FC-RE | 1.3124 | 0.0532 | 1.7063 | 0.0321 | 0.0476 | 0.3572 | 0.8969 | -0.0291 | 4.3766 |
| ICE-FE | 0.0601 | 0.0016 | 0.0279 | 0.0321 | 0.0394 | 0.0199 | 0.0570 | -0.0013 | 0.2366 |
| ICE-RE | 0.0601 | 0.0016 | 0.0991 | 0.0321 | 0.0394 | 0.0341 | 0.0570 | -0.0013 | 0.3221 |
| 2025 | | | | | | | | | |
| BE-FE | 0.0793 | 0.0142 | 0.0280 | 0.0321 | 0.0403 | 0.0201 | 0.0699 | -0.0018 | 0.2821 |
| BE-RE | 0.0793 | 0.0142 | 0.0229 | 0.0321 | 0.0403 | 0.0191 | 0.0699 | -0.0018 | 0.2760 |
| FC-FE | 0.0547 | 0.0019 | 0.0282 | 0.0321 | 0.0461 | 0.0213 | 0.0533 | -0.0012 | 0.2364 |
| FC-RE | 0.0547 | 0.0019 | 0.0598 | 0.0321 | 0.0461 | 0.0276 | 0.0533 | -0.0012 | 0.2743 |
| ICE-FE | 0.0379 | 0.0013 | 0.0303 | 0.0321 | 0.0394 | 0.0204 | 0.0421 | -0.0008 | 0.2027 |
| ICE-RE | 0.0379 | 0.0013 | 0.0642 | 0.0321 | 0.0394 | 0.0272 | 0.0421 | -0.0008 | 0.2433 |
| 2030 | | | | | | | | | |
| BE-FE | 0.0312 | 0.0085 | 0.0157 | 0.0321 | 0.0403 | 0.0176 | 0.0376 | -0.0007 | 0.1824 |
| BE-RE | 0.0312 | 0.0085 | 0.0065 | 0.0321 | 0.0403 | 0.0158 | 0.0376 | -0.0007 | 0.1714 |
| FC-FE | 0.0269 | 0.0008 | 0.0181 | 0.0321 | 0.0445 | 0.0190 | 0.0347 | -0.0006 | 0.1755 |
| FC-RE | 0.0269 | 0.0008 | 0.0192 | 0.0321 | 0.0445 | 0.0192 | 0.0347 | -0.0006 | 0.1768 |
| ICE-FE | 0.0273 | 0.0011 | 0.0344 | 0.0321 | 0.0394 | 0.0212 | 0.0350 | -0.0006 | 0.1899 |
| ICE-RE | 0.0273 | 0.0011 | 0.0376 | 0.0321 | 0.0394 | 0.0218 | 0.0350 | -0.0006 | 0.1938 |
| 2050 | | | | | | | | | |
| BE-FE | 0.0208 | 0.0073 | 0.0170 | 0.0321 | 0.0403 | 0.0179 | 0.0306 | -0.0005 | 0.1656 |
| BE-RE | 0.0208 | 0.0073 | 0.0026 | 0.0321 | 0.0403 | 0.0150 | 0.0306 | -0.0005 | 0.1483 |
| FC-FE | 0.0168 | 0.0007 | 0.0174 | 0.0321 | 0.0430 | 0.0185 | 0.0279 | -0.0004 | 0.1560 |
| FC-RE | 0.0168 | 0.0007 | 0.0094 | 0.0321 | 0.0430 | 0.0169 | 0.0279 | -0.0004 | 0.1464 |
| ICE-FE | 0.0182 | 0.0011 | 0.0393 | 0.0321 | 0.0394 | 0.0222 | 0.0289 | -0.0004 | 0.1807 |
| ICE-RE | 0.0182 | 0.0011 | 0.0213 | 0.0321 | 0.0394 | 0.0186 | 0.0289 | -0.0004 | 0.1591 |

*Technologically infeasible

**Table S6.** TCO results for airport shuttles (19-seater, 50-km-range) under the baseline scenario

| Technology | TCO breakdown ($/passenger-km) | | | | | | | | Total |
| --- | --- | --- | --- | --- | --- | --- | --- | --- | --- |
|  | VBM | Powertrain | Energy | Piloting | Infrastructure | TFI | Maintenance | Salvage value |  |
| 2020 | | | | | | | | | |
| BE-FE | 0.0456 | 0.0086 | 0.0118 | 0.0196 | 0.0246 | 0.0112 | 0.0408 | -0.0010 | 0.1612 |
| BE-RE | 0.0456 | 0.0086 | 0.0170 | 0.0196 | 0.0246 | 0.0123 | 0.0408 | -0.0010 | 0.1675 |
| FC-FE | 0.3305 | 0.0123 | 0.1731 | 0.0196 | 0.0291 | 0.0444 | 0.2319 | -0.0073 | 0.8335 |
| FC-RE | 0.3305 | 0.0123 | 0.5729 | 0.0196 | 0.0291 | 0.1243 | 0.2319 | -0.0073 | 1.3133 |
| ICE-FE | 0.0542 | 0.0015 | 0.0344 | 0.0196 | 0.0241 | 0.0156 | 0.0465 | -0.0012 | 0.1947 |
| ICE-RE | 0.0542 | 0.0015 | 0.1221 | 0.0196 | 0.0241 | 0.0332 | 0.0465 | -0.0012 | 0.3000 |
| 2025 | | | | | | | | | |
| BE-FE | 0.0246 | 0.0081 | 0.0114 | 0.0196 | 0.0246 | 0.0111 | 0.0267 | -0.0005 | 0.1257 |
| BE-RE | 0.0246 | 0.0081 | 0.0093 | 0.0196 | 0.0246 | 0.0107 | 0.0267 | -0.0005 | 0.1232 |
| FC-FE | 0.0459 | 0.0015 | 0.0320 | 0.0196 | 0.0282 | 0.0160 | 0.0410 | -0.0010 | 0.1831 |
| FC-RE | 0.0459 | 0.0015 | 0.0679 | 0.0196 | 0.0282 | 0.0231 | 0.0410 | -0.0010 | 0.2262 |
| ICE-FE | 0.0345 | 0.0012 | 0.0376 | 0.0196 | 0.0241 | 0.0163 | 0.0333 | -0.0008 | 0.1659 |
| ICE-RE | 0.0345 | 0.0012 | 0.0797 | 0.0196 | 0.0241 | 0.0247 | 0.0333 | -0.0008 | 0.2163 |
| 2030 | | | | | | | | | |
| BE-FE | 0.0173 | 0.0088 | 0.0116 | 0.0196 | 0.0246 | 0.0112 | 0.0218 | -0.0004 | 0.1146 |
| BE-RE | 0.0173 | 0.0088 | 0.0048 | 0.0196 | 0.0246 | 0.0098 | 0.0218 | -0.0004 | 0.1065 |
| FC-FE | 0.0236 | 0.0006 | 0.0217 | 0.0196 | 0.0272 | 0.0137 | 0.0260 | -0.0005 | 0.1319 |
| FC-RE | 0.0236 | 0.0006 | 0.0229 | 0.0196 | 0.0272 | 0.0139 | 0.0260 | -0.0005 | 0.1333 |
| ICE-FE | 0.0250 | 0.0010 | 0.0428 | 0.0196 | 0.0241 | 0.0173 | 0.0269 | -0.0006 | 0.1561 |
| ICE-RE | 0.0250 | 0.0010 | 0.0468 | 0.0196 | 0.0241 | 0.0181 | 0.0269 | -0.0006 | 0.1610 |
| 2050 | | | | | | | | | |
| BE-FE | 0.0116 | 0.0076 | 0.0126 | 0.0196 | 0.0246 | 0.0114 | 0.0179 | -0.0003 | 0.1050 |
| BE-RE | 0.0116 | 0.0076 | 0.0019 | 0.0196 | 0.0246 | 0.0092 | 0.0179 | -0.0003 | 0.0922 |
| FC-FE | 0.0147 | 0.0006 | 0.0208 | 0.0196 | 0.0263 | 0.0133 | 0.0201 | -0.0003 | 0.1152 |
| FC-RE | 0.0147 | 0.0006 | 0.0113 | 0.0196 | 0.0263 | 0.0114 | 0.0201 | -0.0003 | 0.1037 |
| ICE-FE | 0.0166 | 0.0010 | 0.0489 | 0.0196 | 0.0241 | 0.0185 | 0.0214 | -0.0004 | 0.1497 |
| ICE-RE | 0.0166 | 0.0010 | 0.0265 | 0.0196 | 0.0241 | 0.0140 | 0.0214 | -0.0004 | 0.1228 |

**Table S7.** TCO results for private flying cars (1-seater, 100-km-range) under the alternative scenarios for 2050

| Technology | TCO breakdown ($/passenger-km) | | | | | | | | Total |
| --- | --- | --- | --- | --- | --- | --- | --- | --- | --- |
|  | VBM | Powertrain | Energy | Piloting | Infrastructure | TFI | Maintenance | Salvage value |  |
| Lagged battery technology development | | | | | | | | | |
| BE-FE | 0.0122 | 0.0029 | 0.0068 | 0 | 0.2218 | 0.0457 | 0.0999 | -0.0003 | 0.3891 |
| BE-RE | 0.0122 | 0.0029 | 0.0010 | 0 | 0.2218 | 0.0446 | 0.0999 | -0.0003 | 0.3822 |
| Advanced battery technology development | | | | | | | | | |
| BE-FE | 0.0112 | 0.0040 | 0.0058 | 0 | 0.2218 | 0.0455 | 0.0992 | -0.0002 | 0.3873 |
| BE-RE | 0.0112 | 0.0040 | 0.0009 | 0 | 0.2218 | 0.0445 | 0.0992 | -0.0002 | 0.3814 |
| Lagged fuel cell technology development | | | | | | | | | |
| FC-FE | 0.0174 | 0.0005 | 0.0167 | 0 | 0.2365 | 0.0506 | 0.1034 | -0.0004 | 0.4247 |
| FC-RE | 0.0174 | 0.0005 | 0.0090 | 0 | 0.2365 | 0.0491 | 0.1034 | -0.0004 | 0.4155 |
| Advanced fuel cell technology development | | | | | | | | | |
| FC-FE | 0.0123 | 0.0003 | 0.0081 | 0 | 0.2365 | 0.0489 | 0.0999 | -0.0003 | 0.4058 |
| FC-RE | 0.0123 | 0.0003 | 0.0044 | 0 | 0.2365 | 0.0482 | 0.0999 | -0.0003 | 0.4013 |

**Table S8.** TCO results for short-range air taxis (4-seater, 100-km-range) under the alternative scenarios for 2050

| Technology | TCO breakdown ($/passenger-km) | | | | | | | | Total |
| --- | --- | --- | --- | --- | --- | --- | --- | --- | --- |
|  | VBM | Powertrain | Energy | Piloting | Infrastructure | TFI | Maintenance | Salvage value |  |
| Lagged battery technology development | | | | | | | | | |
| BE-FE | 0.0204 | 0.0071 | 0.0165 | 0.1178 | 0.1479 | 0.0564 | 0.0748 | -0.0005 | 0.4405 |
| BE-RE | 0.0204 | 0.0071 | 0.0025 | 0.1178 | 0.1479 | 0.0536 | 0.0748 | -0.0005 | 0.4237 |
| Advanced battery technology development | | | | | | | | | |
| BE-FE | 0.0179 | 0.0097 | 0.0141 | 0.1178 | 0.1479 | 0.0560 | 0.0732 | -0.0004 | 0.4361 |
| BE-RE | 0.0179 | 0.0097 | 0.0022 | 0.1178 | 0.1479 | 0.0536 | 0.0732 | -0.0004 | 0.4218 |
| Lagged fuel cell technology development | | | | | | | | | |
| FC-FE | 0.0330 | 0.0013 | 0.0405 | 0.1178 | 0.1577 | 0.0632 | 0.0833 | -0.0007 | 0.4960 |
| FC-RE | 0.0330 | 0.0013 | 0.0219 | 0.1178 | 0.1577 | 0.0595 | 0.0833 | -0.0007 | 0.4737 |
| Advanced fuel cell technology development | | | | | | | | | |
| FC-FE | 0.0205 | 0.0006 | 0.0198 | 0.1178 | 0.1577 | 0.0590 | 0.0749 | -0.0005 | 0.4499 |
| FC-RE | 0.0205 | 0.0006 | 0.0107 | 0.1178 | 0.1577 | 0.0572 | 0.0749 | -0.0005 | 0.4390 |

**Table S9.** TCO results for long-range air taxis (4-seater, 400-km-range) under the alternative scenarios for 2050

| Technology | TCO breakdown ($/passenger-km) | | | | | | | | Total |
| --- | --- | --- | --- | --- | --- | --- | --- | --- | --- |
|  | VBM | Powertrain | Energy | Piloting | Infrastructure | TFI | Maintenance | Salvage value |  |
| Lagged battery technology development | | | | | | | | | |
| BE-FE | 0.0518 | 0.0147 | 0.0413 | 0.1178 | 0.1479 | 0.0614 | 0.0959 | -0.0012 | 0.5298 |
| BE-RE | 0.0518 | 0.0147 | 0.0064 | 0.1178 | 0.1479 | 0.0544 | 0.0959 | -0.0012 | 0.4878 |
| Advanced battery technology development | | | | | | | | | |
| BE-FE | 0.0235 | 0.0097 | 0.0171 | 0.1178 | 0.1479 | 0.0565 | 0.0769 | -0.0005 | 0.4488 |
| BE-RE | 0.0235 | 0.0097 | 0.0026 | 0.1178 | 0.1479 | 0.0537 | 0.0769 | -0.0005 | 0.4315 |
| Lagged fuel cell technology development | | | | | | | | | |
| FC-FE | 0.0365 | 0.0015 | 0.0395 | 0.1178 | 0.1577 | 0.0630 | 0.0856 | -0.0008 | 0.5007 |
| FC-RE | 0.0365 | 0.0015 | 0.0214 | 0.1178 | 0.1577 | 0.0594 | 0.0856 | -0.0008 | 0.4789 |
| Advanced fuel cell technology development | | | | | | | | | |
| FC-FE | 0.0214 | 0.0007 | 0.0182 | 0.1178 | 0.1577 | 0.0587 | 0.0755 | -0.0005 | 0.4496 |
| FC-RE | 0.0214 | 0.0007 | 0.0098 | 0.1178 | 0.1577 | 0.0571 | 0.0755 | -0.0005 | 0.4395 |

**Table S10.** TCO results for air vans (12-seater, 400-km-range) under the alternative scenarios for 2050

| Technology | TCO breakdown ($/passenger-km) | | | | | | | | Total |
| --- | --- | --- | --- | --- | --- | --- | --- | --- | --- |
|  | VBM | Powertrain | Energy | Piloting | Infrastructure | TFI | Maintenance | Salvage value |  |
| Lagged battery technology development | | | | | | | | | |
| BE-FE | 0.0396 | 0.0118 | 0.0331 | 0.0321 | 0.0403 | 0.0211 | 0.0433 | -0.0009 | 0.2205 |
| BE-RE | 0.0396 | 0.0118 | 0.0051 | 0.0321 | 0.0403 | 0.0155 | 0.0433 | -0.0009 | 0.1869 |
| Advanced battery technology development | | | | | | | | | |
| BE-FE | 0.0169 | 0.0078 | 0.0137 | 0.0321 | 0.0403 | 0.0172 | 0.0280 | -0.0004 | 0.1557 |
| BE-RE | 0.0169 | 0.0078 | 0.0021 | 0.0321 | 0.0403 | 0.0149 | 0.0280 | -0.0004 | 0.1418 |
| Lagged fuel cell technology development | | | | | | | | | |
| FC-FE | 0.0273 | 0.0012 | 0.0316 | 0.0321 | 0.0430 | 0.0213 | 0.0350 | -0.0006 | 0.1910 |
| FC-RE | 0.0273 | 0.0012 | 0.0171 | 0.0321 | 0.0430 | 0.0184 | 0.0350 | -0.0006 | 0.1736 |
| Advanced fuel cell technology development | | | | | | | | | |
| FC-FE | 0.0153 | 0.0006 | 0.0146 | 0.0321 | 0.0430 | 0.0179 | 0.0269 | -0.0003 | 0.1501 |
| FC-RE | 0.0153 | 0.0006 | 0.0079 | 0.0321 | 0.0430 | 0.0166 | 0.0269 | -0.0003 | 0.1421 |

**Table S11.** TCO results for airport shuttles (19-seater, 50-km-range) under the alternative scenarios for 2050

| Technology | TCO breakdown ($/passenger-km) | | | | | | | | Total |
| --- | --- | --- | --- | --- | --- | --- | --- | --- | --- |
|  | VBM | Powertrain | Energy | Piloting | Infrastructure | TFI | Maintenance | Salvage value |  |
| Lagged battery technology development | | | | | | | | | |
| BE-FE | 0.0123 | 0.0067 | 0.0134 | 0.0196 | 0.0246 | 0.0115 | 0.0185 | -0.0003 | 0.1065 |
| BE-RE | 0.0123 | 0.0067 | 0.0021 | 0.0196 | 0.0246 | 0.0093 | 0.0185 | -0.0003 | 0.0929 |
| Advanced battery technology development | | | | | | | | | |
| BE-FE | 0.0112 | 0.0098 | 0.0122 | 0.0196 | 0.0246 | 0.0113 | 0.0177 | -0.0002 | 0.1061 |
| BE-RE | 0.0112 | 0.0098 | 0.0019 | 0.0196 | 0.0246 | 0.0092 | 0.0177 | -0.0002 | 0.0938 |
| Lagged fuel cell technology development | | | | | | | | | |
| FC-FE | 0.0229 | 0.0010 | 0.0359 | 0.0196 | 0.0263 | 0.0164 | 0.0256 | -0.0005 | 0.1471 |
| FC-RE | 0.0229 | 0.0010 | 0.0194 | 0.0196 | 0.0263 | 0.0131 | 0.0256 | -0.0005 | 0.1274 |
| Advanced fuel cell technology development | | | | | | | | | |
| FC-FE | 0.0136 | 0.0005 | 0.0177 | 0.0196 | 0.0263 | 0.0127 | 0.0193 | -0.0003 | 0.1094 |
| FC-RE | 0.0136 | 0.0005 | 0.0096 | 0.0196 | 0.0263 | 0.0111 | 0.0193 | -0.0003 | 0.0996 |

**Table S12.** Required passenger fares for ROI targets for private flying cars (1-seater, 100-km-range) under the baseline scenario

| Technology | ROI target | Load factor | Required passenger fare ($/passenger-km) | | | |
| --- | --- | --- | --- | --- | --- | --- |
|  |  |  | 2020 | 2025 | 2030 | 2050 |
| BE-FE | 10% | 100% | 1.52 | 1.37 | 1.33 | 1.30 |
|  | 15% | 100% | 1.95 | 1.75 | 1.70 | 1.66 |
|  | 25% | 100% | 2.81 | 2.52 | 2.45 | 2.39 |
| BE-RE | 10% | 100% | 1.53 | 1.36 | 1.31 | 1.27 |
|  | 15% | 100% | 1.96 | 1.75 | 1.68 | 1.63 |
|  | 25% | 100% | 2.83 | 2.52 | 2.43 | 2.35 |
| FC-FE | 10% | 100% | 2.92 | 1.58 | 1.44 | 1.37 |
|  | 15% | 100% | 3.75 | 2.02 | 1.85 | 1.75 |
|  | 25% | 100% | 5.40 | 2.92 | 2.66 | 2.52 |
| FC-RE | 10% | 100% | 3.74 | 1.64 | 1.44 | 1.35 |
|  | 15% | 100% | 4.80 | 2.11 | 1.85 | 1.73 |
|  | 25% | 100% | 6.92 | 3.04 | 2.67 | 2.49 |
| ICE-FE | 10% | 100% | 1.48 | 1.39 | 1.35 | 1.33 |
|  | 15% | 100% | 1.90 | 1.78 | 1.74 | 1.70 |
|  | 25% | 100% | 2.74 | 2.56 | 2.50 | 2.46 |
| ICE-RE | 10% | 100% | 1.62 | 1.45 | 1.36 | 1.29 |
|  | 15% | 100% | 2.08 | 1.87 | 1.75 | 1.66 |
|  | 25% | 100% | 3.00 | 2.69 | 2.52 | 2.39 |

**Table S13.** Required passenger fares for ROI targets for short-range air taxis (4-seater, 100-km-range) under the baseline scenario

| Technology | ROI target | Load factor | Required passenger fare ($/passenger-km) | | | |
| --- | --- | --- | --- | --- | --- | --- |
|  |  |  | 2020 | 2025 | 2030 | 2050 |
| BE-FE | 10% | 50% | 1.86 | 1.58 | 1.51 | 1.46 |
|  |  | 75% | 1.24 | 1.05 | 1.01 | 0.97 |
|  | 15% | 50% | 2.39 | 2.03 | 1.94 | 1.87 |
|  |  | 75% | 1.59 | 1.35 | 1.29 | 1.25 |
|  | 25% | 50% | 3.44 | 2.92 | 2.79 | 2.69 |
|  |  | 75% | 2.29 | 1.95 | 1.86 | 1.80 |
| BE-RE | 10% | 50% | 1.89 | 1.57 | 1.48 | 1.41 |
|  |  | 75% | 1.26 | 1.05 | 0.98 | 0.94 |
|  | 15% | 50% | 2.43 | 2.01 | 1.89 | 1.81 |
|  |  | 75% | 1.62 | 1.34 | 1.26 | 1.20 |
|  | 25% | 50% | 3.50 | 2.90 | 2.73 | 2.60 |
|  |  | 75% | 2.33 | 1.93 | 1.82 | 1.73 |
| FC-FE | 10% | 50% | 4.99 | 1.87 | 1.63 | 1.53 |
|  |  | 75% | 3.33 | 1.25 | 1.08 | 1.02 |
|  | 15% | 50% | 6.41 | 2.40 | 2.08 | 1.96 |
|  |  | 75% | 4.27 | 1.60 | 1.39 | 1.31 |
|  | 25% | 50% | 9.23 | 3.46 | 3.00 | 2.82 |
|  |  | 75% | 6.15 | 2.30 | 2.00 | 1.88 |
| FC-RE | 10% | 50% | 6.98 | 2.03 | 1.63 | 1.48 |
|  |  | 75% | 4.66 | 1.36 | 1.09 | 0.99 |
|  | 15% | 50% | 8.96 | 2.61 | 2.09 | 1.90 |
|  |  | 75% | 5.97 | 1.74 | 1.39 | 1.27 |
|  | 25% | 50% | 12.90 | 3.76 | 3.01 | 2.74 |
|  |  | 75% | 8.60 | 2.50 | 2.01 | 1.83 |
| ICE-FE | 10% | 50% | 1.85 | 1.70 | 1.65 | 1.61 |
|  |  | 75% | 1.23 | 1.13 | 1.10 | 1.07 |
|  | 15% | 50% | 2.37 | 2.18 | 2.11 | 2.06 |
|  |  | 75% | 1.58 | 1.45 | 1.41 | 1.37 |
|  | 25% | 50% | 3.42 | 3.14 | 3.04 | 2.97 |
|  |  | 75% | 2.28 | 2.09 | 2.03 | 1.98 |
| ICE-RE | 10% | 50% | 2.23 | 1.88 | 1.66 | 1.51 |
|  |  | 75% | 1.49 | 1.25 | 1.11 | 1.01 |
|  | 15% | 50% | 2.86 | 2.41 | 2.13 | 1.93 |
|  |  | 75% | 1.91 | 1.61 | 1.42 | 1.29 |
|  | 25% | 50% | 4.13 | 3.48 | 3.07 | 2.79 |
|  |  | 75% | 2.75 | 2.32 | 2.05 | 1.86 |

**Table S14.** Required passenger fares for ROI targets for long-range air taxis (4-seater, 400-km-range) under the baseline scenario

| Technology | ROI target | Load factor | Required passenger fare ($/passenger-km) | | | |
| --- | --- | --- | --- | --- | --- | --- |
|  |  |  | 2020 | 2025 | 2030 | 2050 |
| BE-FE* | 10% | 50% |  | 2.04 | 1.62 | 1.54 |
|  |  | 75% |  | 1.36 | 1.08 | 1.03 |
|  | 15% | 50% |  | 2.62 | 2.08 | 1.98 |
|  |  | 75% |  | 1.75 | 1.38 | 1.32 |
|  | 25% | 50% |  | 3.77 | 2.99 | 2.85 |
|  |  | 75% |  | 2.51 | 1.99 | 1.90 |
| BE-RE* | 10% | 50% |  | 2.02 | 1.57 | 1.47 |
|  |  | 75% |  | 1.34 | 1.05 | 0.98 |
|  | 15% | 50% |  | 2.59 | 2.02 | 1.89 |
|  |  | 75% |  | 1.72 | 1.34 | 1.26 |
|  | 25% | 50% |  | 3.72 | 2.91 | 2.72 |
|  |  | 75% |  | 2.48 | 1.94 | 1.81 |
| FC-FE | 10% | 50% | 13.27 | 1.91 | 1.63 | 1.53 |
|  |  | 75% | 8.84 | 1.27 | 1.09 | 1.02 |
|  | 15% | 50% | 17.02 | 2.45 | 2.09 | 1.96 |
|  |  | 75% | 11.34 | 1.63 | 1.39 | 1.31 |
|  | 25% | 50% | 24.51 | 3.52 | 3.01 | 2.82 |
|  |  | 75% | 16.34 | 2.35 | 2.01 | 1.88 |
| FC-RE | 10% | 50% | 19.23 | 2.06 | 1.64 | 1.49 |
|  |  | 75% | 12.82 | 1.38 | 1.09 | 0.99 |
|  | 15% | 50% | 24.67 | 2.65 | 2.10 | 1.91 |
|  |  | 75% | 16.45 | 1.77 | 1.40 | 1.27 |
|  | 25% | 50% | 35.54 | 3.82 | 3.02 | 2.75 |
|  |  | 75% | 23.69 | 2.54 | 2.02 | 1.83 |
| ICE-FE | 10% | 50% | 1.85 | 1.69 | 1.63 | 1.59 |
|  |  | 75% | 1.24 | 1.13 | 1.09 | 1.06 |
|  | 15% | 50% | 2.38 | 2.17 | 2.09 | 2.04 |
|  |  | 75% | 1.58 | 1.45 | 1.40 | 1.36 |
|  | 25% | 50% | 3.42 | 3.13 | 3.02 | 2.94 |
|  |  | 75% | 2.28 | 2.08 | 2.01 | 1.96 |
| ICE-RE | 10% | 50% | 2.20 | 1.86 | 1.65 | 1.50 |
|  |  | 75% | 1.47 | 1.24 | 1.10 | 1.00 |
|  | 15% | 50% | 2.82 | 2.38 | 2.12 | 1.93 |
|  |  | 75% | 1.88 | 1.59 | 1.41 | 1.28 |
|  | 25% | 50% | 4.07 | 3.43 | 3.05 | 2.77 |
|  |  | 75% | 2.71 | 2.29 | 2.03 | 1.85 |

*Technologically infeasible in 2020

**Table S15.** Required passenger fares for ROI targets for air vans (12-seater, 400-km-range) under the baseline scenario

| Technology | ROI target | Load factor | Required passenger fare ($/passenger-km) | | | |
| --- | --- | --- | --- | --- | --- | --- |
|  |  |  | 2020 | 2025 | 2030 | 2050 |
| BE-FE* | 10% | 50% |  | 0.94 | 0.61 | 0.55 |
|  |  | 75% |  | 0.63 | 0.41 | 0.37 |
|  | 15% | 50% |  | 1.21 | 0.78 | 0.71 |
|  |  | 75% |  | 0.81 | 0.52 | 0.47 |
|  | 25% | 50% |  | 1.74 | 1.13 | 1.02 |
|  |  | 75% |  | 1.16 | 0.75 | 0.68 |
| BE-RE* | 10% | 50% |  | 0.92 | 0.57 | 0.50 |
|  |  | 75% |  | 0.62 | 0.38 | 0.33 |
|  | 15% | 50% |  | 1.18 | 0.74 | 0.64 |
|  |  | 75% |  | 0.79 | 0.49 | 0.42 |
|  | 25% | 50% |  | 1.71 | 1.06 | 0.92 |
|  |  | 75% |  | 1.14 | 0.71 | 0.61 |
| FC-FE | 10% | 50% | 9.86 | 0.79 | 0.59 | 0.52 |
|  |  | 75% | 6.57 | 0.53 | 0.39 | 0.35 |
|  | 15% | 50% | 12.64 | 1.01 | 0.75 | 0.67 |
|  |  | 75% | 8.43 | 0.68 | 0.50 | 0.45 |
|  | 25% | 50% | 18.21 | 1.46 | 1.08 | 0.96 |
|  |  | 75% | 12.14 | 0.97 | 0.72 | 0.64 |
| FC-RE | 10% | 50% | 14.63 | 0.92 | 0.59 | 0.49 |
|  |  | 75% | 9.76 | 0.61 | 0.39 | 0.33 |
|  | 15% | 50% | 18.77 | 1.18 | 0.76 | 0.63 |
|  |  | 75% | 12.51 | 0.78 | 0.51 | 0.42 |
|  | 25% | 50% | 27.04 | 1.69 | 1.09 | 0.90 |
|  |  | 75% | 18.03 | 1.13 | 0.73 | 0.60 |
| ICE-FE | 10% | 50% | 0.79 | 0.68 | 0.63 | 0.60 |
|  |  | 75% | 0.53 | 0.45 | 0.42 | 0.40 |
|  | 15% | 50% | 1.01 | 0.87 | 0.81 | 0.78 |
|  |  | 75% | 0.68 | 0.58 | 0.54 | 0.52 |
|  | 25% | 50% | 1.46 | 1.25 | 1.17 | 1.12 |
|  |  | 75% | 0.97 | 0.83 | 0.78 | 0.74 |
| ICE-RE | 10% | 50% | 1.08 | 0.81 | 0.65 | 0.53 |
|  |  | 75% | 0.72 | 0.54 | 0.43 | 0.35 |
|  | 15% | 50% | 1.38 | 1.04 | 0.83 | 0.68 |
|  |  | 75% | 0.92 | 0.70 | 0.55 | 0.45 |
|  | 25% | 50% | 1.99 | 1.50 | 1.20 | 0.98 |
|  |  | 75% | 1.33 | 1.00 | 0.80 | 0.66 |

*Technologically infeasible in 2020

**Table S16.** Required passenger fares for ROI targets for airport shuttles (19-seater, 50-km-range) under the baseline scenario

| Technology | ROI target | Load factor | Required passenger fare ($/passenger-km) | | | |
| --- | --- | --- | --- | --- | --- | --- |
|  |  |  | 2020 | 2025 | 2030 | 2050 |
| BE-FE | 10% | 50% | 0.54 | 0.42 | 0.38 | 0.35 |
|  |  | 75% | 0.36 | 0.28 | 0.26 | 0.23 |
|  | 15% | 50% | 0.69 | 0.54 | 0.49 | 0.45 |
|  |  | 75% | 0.46 | 0.36 | 0.33 | 0.30 |
|  | 25% | 50% | 1.00 | 0.78 | 0.71 | 0.65 |
|  |  | 75% | 0.66 | 0.52 | 0.47 | 0.43 |
| BE-RE | 10% | 50% | 0.56 | 0.41 | 0.36 | 0.31 |
|  |  | 75% | 0.37 | 0.27 | 0.24 | 0.21 |
|  | 15% | 50% | 0.72 | 0.53 | 0.46 | 0.40 |
|  |  | 75% | 0.48 | 0.35 | 0.30 | 0.26 |
|  | 25% | 50% | 1.03 | 0.76 | 0.66 | 0.57 |
|  |  | 75% | 0.69 | 0.51 | 0.44 | 0.38 |
| FC-FE | 10% | 50% | 2.79 | 0.61 | 0.44 | 0.39 |
|  |  | 75% | 1.86 | 0.41 | 0.29 | 0.26 |
|  | 15% | 50% | 3.57 | 0.79 | 0.57 | 0.49 |
|  |  | 75% | 2.38 | 0.52 | 0.38 | 0.33 |
|  | 25% | 50% | 5.15 | 1.13 | 0.81 | 0.71 |
|  |  | 75% | 3.43 | 0.75 | 0.54 | 0.47 |
| FC-RE | 10% | 50% | 4.39 | 0.76 | 0.45 | 0.35 |
|  |  | 75% | 2.93 | 0.50 | 0.30 | 0.23 |
|  | 15% | 50% | 5.63 | 0.97 | 0.57 | 0.44 |
|  |  | 75% | 3.75 | 0.65 | 0.38 | 0.30 |
|  | 25% | 50% | 8.11 | 1.40 | 0.82 | 0.64 |
|  |  | 75% | 5.41 | 0.93 | 0.55 | 0.43 |
| ICE-FE | 10% | 50% | 0.65 | 0.55 | 0.52 | 0.50 |
|  |  | 75% | 0.43 | 0.37 | 0.35 | 0.33 |
|  | 15% | 50% | 0.83 | 0.71 | 0.67 | 0.64 |
|  |  | 75% | 0.56 | 0.47 | 0.45 | 0.43 |
|  | 25% | 50% | 1.20 | 1.02 | 0.96 | 0.93 |
|  |  | 75% | 0.80 | 0.68 | 0.64 | 0.62 |
| ICE-RE | 10% | 50% | 1.00 | 0.72 | 0.54 | 0.41 |
|  |  | 75% | 0.67 | 0.48 | 0.36 | 0.27 |
|  | 15% | 50% | 1.29 | 0.93 | 0.69 | 0.53 |
|  |  | 75% | 0.86 | 0.62 | 0.46 | 0.35 |
|  | 25% | 50% | 1.85 | 1.34 | 0.99 | 0.76 |
|  |  | 75% | 1.24 | 0.89 | 0.66 | 0.51 |

**Table S17.** Required passenger fares for ROI targets for private flying cars (1-seater, 100-km-range) under the alternative scenario for 2050

| Technology | ROI target | Load factor | Required passenger fare ($/passenger-km) | | | |
| --- | --- | --- | --- | --- | --- | --- |
|  |  |  | Lagged battery | Advanced battery | Lagged fuel cell | Advanced fuel cell |
| BE-FE | 10% | 100% | 1.30 | 1.30 |  |  |
|  | 15% | 100% | 1.67 | 1.66 |  |  |
|  | 25% | 100% | 2.40 | 2.39 |  |  |
| BE-RE | 10% | 100% | 1.28 | 1.28 |  |  |
|  | 15% | 100% | 1.64 | 1.64 |  |  |
|  | 25% | 100% | 2.36 | 2.36 |  |  |
| FC-FE | 10% | 100% |  |  | 1.42 | 1.36 |
|  | 15% | 100% |  |  | 1.82 | 1.74 |
|  | 25% | 100% |  |  | 2.62 | 2.51 |
| FC-RE | 10% | 100% |  |  | 1.39 | 1.34 |
|  | 15% | 100% |  |  | 1.78 | 1.72 |
|  | 25% | 100% |  |  | 2.57 | 2.48 |

**Table S18.** Required passenger fares for ROI targets for short-range air taxis (4-seater, 100-km-range) under the alternative scenario for 2050

| Technology | ROI target | Load factor | Required passenger fare ($/passenger-km) | | | |
| --- | --- | --- | --- | --- | --- | --- |
|  |  |  | Lagged battery | Advanced battery | Lagged fuel cell | Advanced fuel cell |
| BE-FE | 10% | 50% | 1.47 | 1.46 |  |  |
|  |  | 75% | 0.98 | 0.97 |  |  |
|  | 15% | 50% | 1.89 | 1.87 |  |  |
|  |  | 75% | 1.26 | 1.25 |  |  |
|  | 25% | 50% | 2.72 | 2.69 |  |  |
|  |  | 75% | 1.81 | 1.80 |  |  |
| BE-RE | 10% | 50% | 1.42 | 1.41 |  |  |
|  |  | 75% | 0.94 | 0.94 |  |  |
|  | 15% | 50% | 1.82 | 1.81 |  |  |
|  |  | 75% | 1.21 | 1.21 |  |  |
|  | 25% | 50% | 2.62 | 2.61 |  |  |
|  |  | 75% | 1.75 | 1.74 |  |  |
| FC-FE | 10% | 50% |  |  | 1.66 | 1.50 |
|  |  | 75% |  |  | 1.11 | 1.00 |
|  | 15% | 50% |  |  | 2.13 | 1.93 |
|  |  | 75% |  |  | 1.42 | 1.29 |
|  | 25% | 50% |  |  | 3.06 | 2.78 |
|  |  | 75% |  |  | 2.04 | 1.85 |
| FC-RE | 10% | 50% |  |  | 1.58 | 1.47 |
|  |  | 75% |  |  | 1.06 | 0.98 |
|  | 15% | 50% |  |  | 2.03 | 1.88 |
|  |  | 75% |  |  | 1.35 | 1.26 |
|  | 25% | 50% |  |  | 2.93 | 2.71 |
|  |  | 75% |  |  | 1.95 | 1.81 |

**Table S19.** Required passenger fares for ROI targets for long-range air taxis (4-seater, 400-km-range) under the alternative scenario for 2050

| Technology | ROI target | Load factor | Required passenger fare ($/passenger-km) | | | |
| --- | --- | --- | --- | --- | --- | --- |
|  |  |  | Lagged battery | Advanced battery | Lagged fuel cell | Advanced fuel cell |
| BE-FE | 10% | 50% | 1.77 | 1.50 |  |  |
|  |  | 75% | 1.18 | 1.00 |  |  |
|  | 15% | 50% | 2.27 | 1.92 |  |  |
|  |  | 75% | 1.51 | 1.28 |  |  |
|  | 25% | 50% | 3.27 | 2.77 |  |  |
|  |  | 75% | 2.18 | 1.85 |  |  |
| BE-RE | 10% | 50% | 1.63 | 1.44 |  |  |
|  |  | 75% | 1.09 | 0.96 |  |  |
|  | 15% | 50% | 2.09 | 1.85 |  |  |
|  |  | 75% | 1.39 | 1.23 |  |  |
|  | 25% | 50% | 3.01 | 2.67 |  |  |
|  |  | 75% | 2.01 | 1.78 |  |  |
| FC-FE | 10% | 50% |  |  | 1.67 | 1.50 |
|  |  | 75% |  |  | 1.12 | 1.00 |
|  | 15% | 50% |  |  | 2.15 | 1.93 |
|  |  | 75% |  |  | 1.43 | 1.29 |
|  | 25% | 50% |  |  | 3.09 | 2.78 |
|  |  | 75% |  |  | 2.06 | 1.85 |
| FC-RE | 10% | 50% |  |  | 1.60 | 1.47 |
|  |  | 75% |  |  | 1.07 | 0.98 |
|  | 15% | 50% |  |  | 2.05 | 1.88 |
|  |  | 75% |  |  | 1.37 | 1.26 |
|  | 25% | 50% |  |  | 2.96 | 2.72 |
|  |  | 75% |  |  | 1.97 | 1.81 |

**Table S20.** Required passenger fares for ROI targets for air vans (12-seater, 400-km-range) under the alternative scenario for 2050

| Technology | ROI target | Load factor | Required passenger fare ($/passenger-km) | | | |
| --- | --- | --- | --- | --- | --- | --- |
|  |  |  | Lagged battery | Advanced battery | Lagged fuel cell | Advanced fuel cell |
| BE-FE | 10% | 50% | 0.74 | 0.52 |  |  |
|  |  | 75% | 0.49 | 0.35 |  |  |
|  | 15% | 50% | 0.95 | 0.67 |  |  |
|  |  | 75% | 0.63 | 0.45 |  |  |
|  | 25% | 50% | 1.36 | 0.96 |  |  |
|  |  | 75% | 0.91 | 0.64 |  |  |
| BE-RE | 10% | 50% | 0.62 | 0.47 |  |  |
|  |  | 75% | 0.42 | 0.32 |  |  |
|  | 15% | 50% | 0.80 | 0.61 |  |  |
|  |  | 75% | 0.53 | 0.41 |  |  |
|  | 25% | 50% | 1.15 | 0.88 |  |  |
|  |  | 75% | 0.77 | 0.58 |  |  |
| FC-FE | 10% | 50% |  |  | 0.64 | 0.50 |
|  |  | 75% |  |  | 0.43 | 0.33 |
|  | 15% | 50% |  |  | 0.82 | 0.64 |
|  |  | 75% |  |  | 0.55 | 0.43 |
|  | 25% | 50% |  |  | 1.18 | 0.93 |
|  |  | 75% |  |  | 0.79 | 0.62 |
| FC-RE | 10% | 50% |  |  | 0.58 | 0.48 |
|  |  | 75% |  |  | 0.39 | 0.32 |
|  | 15% | 50% |  |  | 0.74 | 0.61 |
|  |  | 75% |  |  | 0.50 | 0.41 |
|  | 25% | 50% |  |  | 1.07 | 0.88 |
|  |  | 75% |  |  | 0.72 | 0.59 |

**Table S21.** Required passenger fares for ROI targets for airport shuttles (19-seater, 50-km-range) under the alternative scenario for 2050

| Technology | ROI target | Load factor | Required passenger fare ($/passenger-km) | | | |
| --- | --- | --- | --- | --- | --- | --- |
|  |  |  | Lagged battery | Advanced battery | Lagged fuel cell | Advanced fuel cell |
| BE-FE | 10% | 50% | 0.36 | 0.35 |  |  |
|  |  | 75% | 0.24 | 0.24 |  |  |
|  | 15% | 50% | 0.46 | 0.46 |  |  |
|  |  | 75% | 0.30 | 0.30 |  |  |
|  | 25% | 50% | 0.66 | 0.66 |  |  |
|  |  | 75% | 0.44 | 0.44 |  |  |
| BE-RE | 10% | 50% | 0.31 | 0.31 |  |  |
|  |  | 75% | 0.21 | 0.21 |  |  |
|  | 15% | 50% | 0.40 | 0.40 |  |  |
|  |  | 75% | 0.27 | 0.27 |  |  |
|  | 25% | 50% | 0.57 | 0.58 |  |  |
|  |  | 75% | 0.38 | 0.39 |  |  |
| FC-FE | 10% | 50% |  |  | 0.49 | 0.37 |
|  |  | 75% |  |  | 0.33 | 0.24 |
|  | 15% | 50% |  |  | 0.63 | 0.47 |
|  |  | 75% |  |  | 0.42 | 0.31 |
|  | 25% | 50% |  |  | 0.91 | 0.68 |
|  |  | 75% |  |  | 0.61 | 0.45 |
| FC-RE | 10% | 50% |  |  | 0.43 | 0.33 |
|  |  | 75% |  |  | 0.28 | 0.22 |
|  | 15% | 50% |  |  | 0.55 | 0.43 |
|  |  | 75% |  |  | 0.36 | 0.28 |
|  | 25% | 50% |  |  | 0.79 | 0.62 |
|  |  | 75% |  |  | 0.52 | 0.41 |

**SI References**

1. P. R. Brown, P. J. Gagnon, J. S. Corcoran, W. J. Cole (2022) Retail Rate Projections for Long-Term Electricity System Models.

2. U.S. Energy Information Administration (2022) Levelized Costs of New Generation Resources in the Annual Energy Outlook 2022.

3. M. Muratori, E. Kontou, J. Eichman, Electricity rates for electric vehicle direct current fast charging in the United States. *Renewable and Sustainable Energy Reviews* **113** (2019).

4. A. Stoll (2015) Analysis and Full Scale Testing of the Joby S4 Propulsion System. (Joby Aviation).

5. D. G. Ullman (2017) Comparing Electric Sky Taxi Visions.

6. A. Bacchini, E. Cestino, Electric VTOL Configurations Comparison. *Aerospace* **6** (2019).

7. A. Kasliwal *et al.*, Role of flying cars in sustainable mobility. *Nat Commun* **10**, 1555 (2019).

8. Y. Luo, Y. Qian, Z. Zeng, Y. Zhang, Simulation and analysis of operating characteristics of power battery for flying car utilization. *eTransportation* **8** (2021).

9. Y. Zhang *et al.*, Progress and key technologies of flying cars. *Journal of Automotive Safety and Energy* **11**, 1-16 (2020).

10. M. Liu *et al.*, Lifecycle greenhouse gas emissions and energy cost analysis of flying cars with three different propulsion systems. *Journal of Cleaner Production* **331** (2022).

11. Uber (2016) Fast-Forwarding to a Future of On-Demand Urban Air Transportation.

12. R. Goyal (2018) Urban Air Mobility (UAM) Market Study.

13. International Air Transport Association (2021) Annual Review 2021.

14. X. Sun, M. Ouyang, H. Hao, Surging lithium price will not impede the electric vehicle boom. *Joule* **6**, 1738-1742 (2022).

15. R. Schmuch, R. Wagner, G. Hörpel, T. Placke, M. Winter, Performance and cost of materials for lithium-based rechargeable automotive batteries. *Nature Energy* **3**, 267-278 (2018).

16. R. K. Ahluwalia, J. K. Peng, X. Wang, D. Papadias, J. Kopasz, Performance and cost of fuel cells for urban air mobility. *International Journal of Hydrogen Energy* **46**, 36917-36929 (2021).

17. Y. Chen, M. Melaina, Model-based techno-economic evaluation of fuel cell vehicles considering technology uncertainties. *Transportation Research Part D: Transport and Environment* **74**, 234-244 (2019).

18. Z. Liu *et al.*, Comparing total cost of ownership of battery electric vehicles and internal combustion engine vehicles. *Energy Policy* **158** (2021).

19. W. Cai, X. Wu, M. Zhou, Y. Liang, Y. Wang, Review and Development of Electric Motor Systems and Electric Powertrains for New Energy Vehicles. *Automotive Innovation* **4**, 3-22 (2021).

20. International Energy Agency (2021) World Energy Outlook 2021.

21. International Energy Agency (2020) Projected Costs of Generating Electricity (2020 Edition).

22. International Renewable Energy Agency (2020) Global Renewables Outlook 2020.

23. Hydrogen Council (2020) Path to Hydrogen Competitiveness: A Cost Perspective.

24. U.S. Energy Information Administration (2021) International Energy Outlook 2021.

25. McKinsey & Company (2020) Clean Skies for Tomorrow: Sustainable Aviation Fuels as a Pathway to Net-Zero Aviation.

26. R. Lu, M. Li, G. Gu, J. Shi, Y. Lin (2021) Battery Swapping Station, the Next 100 Billion Market.

27. International Energy Agency (2019) The Future of Hydrogen.

28. D. Apostolou, G. Xydis, A literature review on hydrogen refuelling stations and infrastructure. Current status and future prospects. *Renewable and Sustainable Energy Reviews* **113** (2019).

29. Y. Wang *et al.*, Polymer electrolyte membrane fuel cell and hydrogen station networks for automobiles: Status, technology, and perspectives. *Advances in Applied Energy* **2** (2021).

30. W. Yang (2020) Research on Feasibility of Gas Station Renting Project of Company C. (Nanchang University).
